# Inbred Strain Variant Database (ISVdb): A Repository for Probabilistically Informed Sequence Differences Among the Collaborative Cross Strains and Their Founders

**DOI:** 10.1534/g3.117.041491

**Published:** 2017-06-05

**Authors:** Daniel Oreper, Yanwei Cai, Lisa M. Tarantino, Fernando Pardo-Manuel de Villena, William Valdar

**Affiliations:** * Curriculum in Bioinformatics and Computational Biology, University of North Carolina, Chapel Hill, North Carolina 27599-7265; † Department of Genetics, University of North Carolina, Chapel Hill, North Carolina 27599-7265; ‡ Lineberger Comprehensive Cancer Center,; § Division of Pharmacotherapy and Experimental Therapeutics, Eshelman School of Pharmacy University of North Carolina, Chapel Hill, North Carolina 27599-7265

**Keywords:** Collaborative Cross, inbred strain, online GUI, variant imputation, haplotype, multiparental populations, MPP

## Abstract

The Collaborative Cross (CC) is a panel of recently established multiparental recombinant inbred mouse strains. For the CC, as for any multiparental population (MPP), effective experimental design and analysis benefit from detailed knowledge of the genetic differences between strains. Such differences can be directly determined by sequencing, but until now whole-genome sequencing was not publicly available for individual CC strains. An alternative and complementary approach is to infer genetic differences by combining two pieces of information: probabilistic estimates of the CC haplotype mosaic from a custom genotyping array, and probabilistic variant calls from sequencing of the CC founders. The computation for this inference, especially when performed genome-wide, can be intricate and time-consuming, requiring the researcher to generate nontrivial and potentially error-prone scripts. To provide standardized, easy-to-access CC sequence information, we have developed the Inbred Strain Variant Database (ISVdb). The ISVdb provides, for all the exonic variants from the Sanger Institute mouse sequencing dataset, direct sequence information for CC founders and, critically, the imputed sequence information for CC strains. Notably, the ISVdb also: (1) provides predicted variant consequence metadata; (2) allows rapid simulation of F1 populations; and (3) preserves imputation uncertainty, which will allow imputed data to be refined in the future as additional sequencing and genotyping data are collected. The ISVdb information is housed in an SQL database and is easily accessible through a custom online interface (http://isvdb.unc.edu), reducing the analytic burden on any researcher using the CC.

The Collaborative Cross (CC) is a large panel of recombinant inbred mouse strains derived from a genetically diverse set of eight inbred founder strains: A/J (AJ), C57BL/6J (B6), 129S1Sv/ImJ (129), NOD/ShiLtJ (NOD), NZO/HlLtJ (NZO), CAST/EiJ (CAST), PWK/PhJ (PWK), and WSB/EiJ (WSB). These eight founder strains were first outcrossed for three generations to produce mice with contributions from all eight founder strains. These outcrosses were initiated, with different founder orderings, in over 1000 independent breeding funnels (Shorter *et al.* 2017). Mice within each funnel were subsequently inbred for multiple generations until two or more animals were identified by MegaMUGA genotyping as collectively having over 90% of the genome fixed (*i.e.*, homozygous and consistent for a founder haplotype). These animals, hereafter termed the most recent common ancestors (MRCAs), were then chosen to become the obligate ancestors of all subsequent generations and bred to produce a distinct CC strain. The set of MRCAs from all strains composes the CC’s obligate ancestors; that is, the set of individuals that together circumscribes the initial genetic material that can be passed on to subsequent CC mice. As a result of this breeding scheme, the inbred CC strain genomes are random and independent mosaics of the eight founder haplotypes Collaborative Cross Consortium (2012); (Srivastava *et al.* 2017) (Figure 1; more details at http://csbio.unc.edu/CCstatus).

**Figure 1:**
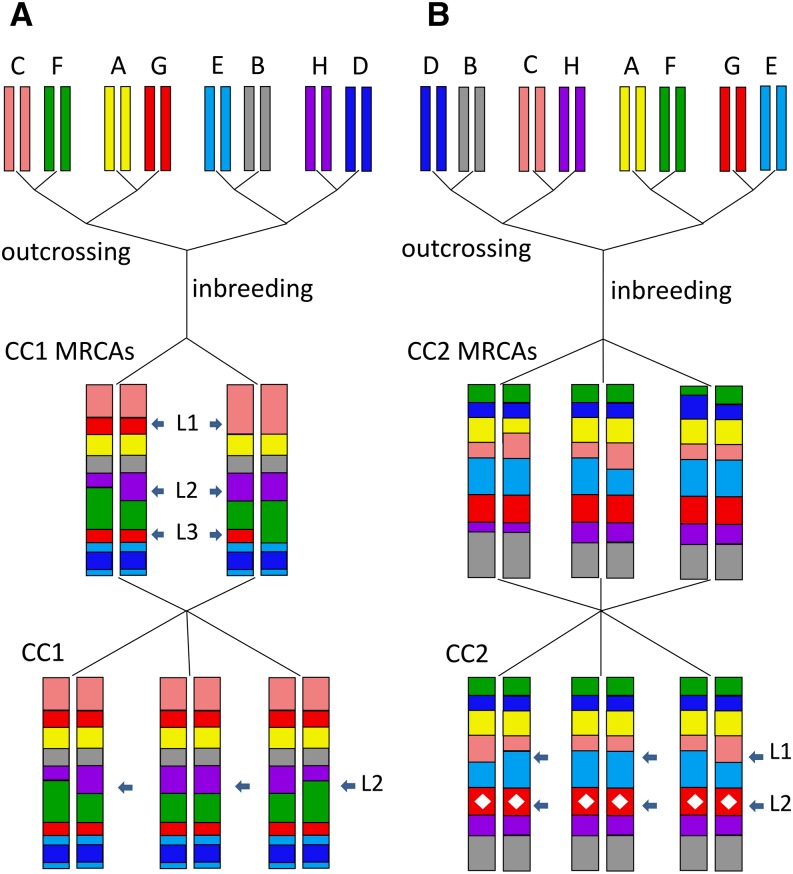
Breeding process for two Collaborative Cross (CC) strains. Both funnels begin by outcrossing the same eight founders, but the initial outcrossing order differs, resulting in completely independent populations per funnel. Animals are outcrossed for three generations, then inbred until genotyping reveals at least two animals with >90% consistent homozygosity by haploytpe. These homozygous animals [a.k.a., the most recent common ancestors (MRCAs)] are chosen to become the obligate ancestors for the CC strains; all subsequent generations of a CC strain descend from a subset of the MRCAs. In (A), arrows show CC1 MRCA regions of inconsistent homozygosity (L1) and residual heterozygosity (L2 and L3). After further inbreeding, only L2 continues to segregate. In (B), the CC2 MRCA set includes three animals rather than two. After further inbreeding, only L1 continues to segregate, but a *de novo* mutation has become fixed at L2.

This combination of independent genomes and high genetic diversity, along with the reproducibility of inbred strains, has made the CC a unique resource in mammalian genetics, and early studies on the CC have begun to exploit these features (Aylor *et al.* 2011; Ferris *et al.* 2013; Phillippi *et al.* 2014; Rasmussen *et al.* 2014; Mosedale *et al.* 2017; Green *et al.* 2017; Gralinski *et al.* 2017). To make optimal use of the CC strains, it is desirable to have an accurate catalog of the genetic differences between them—specifically, the positions and other characteristics of all known CC strain genetic variants—and to be able to predict which variants will be polymorphic in future hypothetical crosses of CC strains and of CC strains with other laboratory strains.

Such information could be determined directly by sequencing, but CC sequencing, soon to be released for a single male per strain (Srivastava *et al.* 2017), is not yet easily accessible. Furthermore, sequencing from a single animal will not resolve uncertainty arising from residual heterozygosity, since two animals from the same strain could easily differ at residually heterozygous loci. More generally, whole-genome sequencing in most organisms is expensive and inconvenient.

A commonly used alternative is haplotype-based variant imputation, whereby comparatively sparse and cheap genotyping data are combined with more complete information about allelic state in (even extremely distant) relatives to infer allelic state at ungenotyped positions in the target sample. This typically involves inferring shared haplotype blocks and, by assuming that individuals sharing a haplotype block also share the corresponding allelic state, using this to impute genotype (Li *et al.* 2009; Marchini and Howie 2010). A broad array of such imputation methods have been developed for use in humans (*e.g.*, Hawley and Kidd 1995; Scheet and Stephens 2006; Marchini *et al.* 2007; Browning and Browning 2009; Howie *et al.* 2009; Li *et al.* 2010) and livestock (*e.g.*, VanRaden *et al.* 2011; Hickey *et al.* 2012; Sargolzaei *et al.* 2014); these typically either start by inferring haplotypes from the genotype data or by approximating the pool of extant haplotypes via a large reference panel, and then use those haplotypes as a means to impute variant genotypes, which are assumed to be the primary interest.

In MPPs of model organisms, where the founder haplotypes are usually known, it is more common for primary interest to focus on reconstructing the haplotype mosaic itself, *e.g.*, for the purpose of linkage disequilibrium mapping (Mott *et al.* 2000; Liu *et al.* 2010; King *et al.* 2012; Fu *et al.* 2012; Gatti *et al.* 2014; Verbyla *et al.* 2014; Zheng *et al.* 2015). In such cases, haplotype-based imputation of specific variants may proceed as a second, refinement step to inform fine-mapping and candidate prioritization (Yalcin *et al.* 2005; Tian *et al.* 2011). In any analyses using imputed variants, it important to note that the haplotype-based variant imputation is inherently probabilistic. A failure to account for variant imputation uncertainty can negatively affect the robustness of downstream decisions (*e.g.*, overconfidence in a functional assignment), and can also produce misleading estimations of association significance and/or variant effects (*e.g.*, Marchini *et al.* 2007; Guan and Stephens 2008; Kutalik *et al.* 2011; Zheng *et al.* 2011; Zhang *et al.* 2014).

Haplotype-based variant imputation lends itself particularly well to MPPs because the haplotype blocks and the variants within are drawn from a known and relatively limited number of founders that can be (more) affordably deeply sequenced and genotyped. This in turn reduces variant imputation uncertainty. For MPP RI strains in particular, once an animal’s variants are imputed, the need for even sparse genotyping is largely obviated in its inbred descendants; they are effectively genotyped as well. Haplotypes can, and have been, similarly imputed for the entire CC population based on the CC MRCAs. In particular, a hidden Markov model (HMM)-based method (Fu *et al.* 2012) has previously been applied to MegaMUGA genotyping of the CC MRCA animals coupled with MegaMUGA genotyping from founder animals, (Welsh *et al.* 2012; Morgan *et al.* 2015; Srivastava *et al.* 2017) to impute a probabilistic estimate of each CC strain’s haplotype mosaic. Sequencing of the founders by the Sanger Institute has provided a catalog of the sequence variants within the founder haplotypes (Keane *et al.* 2011) as well as their predicted functional consequences (McLaren *et al.* 2010), allowing for CC variant imputation.

However, although probabilistic imputed descriptions of CC haplotypes are already available, the final step of imputing probabilistic CC variant state using these haplotypes is currently left up to the researcher. This imputation step can be time-consuming, especially genome-wide, and it typically requires the researcher to develop their own ad-hoc, nontrivial scripts to parse and process input files. We sought to ease this burden by creating the ISVdb. This database computes and stores imputed probabilistic CC variant information once, and then provides efficient, uniform, and convenient access through a publicly accessible webtool.

## ISVdb stored data and functionality

For all Sanger Institute sequencing variant positions that are exonic (or 100 bp upstream or downstream), polymorphic between CC founders, and correspond to SNP/indels, the ISVdb provides conveniently accessible information on the following:

- unphased genotypes for CC strains/founders, including the functional consequences per genotype, per transcript;
- unphased haplotype pairs (hereafter, “diplotypes”) for CC strains, derived probabilistically from MegaMUGA genotyping;
- unphased genotypes for hypothetical F1 crosses among and between CC and founder strains;
- unphased diplotypes for hypothetical F1 crosses among and between CC and founder strains.

All information in the ISVdb is associated with a probability, to reflect the uncertainty of inference (discussed in the next section). The ISVdb interface is designed to be practical and oriented toward concrete tasks. For example, the ISVdb could be used to answer the following questions:

- Given microarray measurements of CC expression, which probes ought to be masked from analysis to minimize the effect of differential hybridization due to variants within the corresponding probed regions?
- Where should PCR primers be designed to bind so as to avoid differential hybridization, while still amplifying informative regions?
- What are the alleles per variant per CC strain in a given region, in order to perform association mapping?
- Given a pair of CC strains, or set of pairs of CC strains, where would the resulting F1 offspring be heterozygous? Which CC strains could be crossed with one another, or against a founder strain, to ensure that a certain region is heterozygous in the offspring?
- Which CC strains contain a stop-gain codon in a particular gene?
- What is the ratio of missense to synonymous mutations on a particular chromosome?
- Which regions are fixed across all CC strains? Which regions are still segregating in a subset of CC strains?
- Which regions are most uncertain, either in haplotype or in genotype, across CC strains?

## ISVdb preserves uncertainty

The primary purpose of the ISVdb is to provide genome-wide, inferred CC genotypes. However, this inference depends on several processes and measurements that are themselves imprecise: (1) sequencing of founder strains was imperfect so some founder variant calls are ambiguous or incorrect; (2) uncertainty in the sparse, genotyping-based estimates of CC diplotypes; and (3) the CC strains themselves are still segregating in some regions.

Properly representing and accounting for such sources of uncertainty is essential to avoid inaccuracies in downstream inference. Consider the effect of diplotype uncertainty on predicting the functional consequences of the alleles in an F1 hybrid from two CC mice.

Suppose one of the CC parents had a 40:30:30 probability of AJ/AJ *vs.* 129/129 *vs.* NOD/NOD diplotype, where AJ carries a synonymous mutation, whereas 129 and NOD both carry a stop-gain mutation. Assuming the most likely diplotype, AJ/AJ, would imply a synonymous mutation in the F1, even though there is a greater (60%) probability of a stop-gain mutation.

The closest similar resource to the ISVdb, the CC “pseudogenomes” set, (Morgan and Welsh 2015; Huang *et al.* 2013) was designed primarily for sequence alignment: it employs most-likely point estimates of genotype and assumes that all alleles are fixed. Therefore, a key secondary goal of the ISVdb is to provide a resource that retains all of the aforementioned uncertainty in CC and F1 genotype inference. The ISVdb achieves this by storing multiple records per variant, in which each record includes a probability of that variant state.

## Methods

### Inputs for database construction

All inputs are based on the GRCm38 mouse reference assembly. Probabilistic estimates of CC unphased diplotypes were computed as of March 24, 2016. These diplotype estimates were derived from a HMM applied to MegaMUGA microarray measurements (Morgan and Welsh 2015).

Founder variants were determined using the Sanger Institute Mouse Genomes Project mouse variant VCF files, REL-1410 (from October 2014), corresponding to Ensembl release 75 of GRCm38 (Keane *et al.* 2011). These VCF files included SNPs and indels (1–100 bp). Exon boundaries were drawn from Ensembl release 75 as well (Ramasamy *et al.* 2013).

### CC genotype and diplotype inference: derivation

By tracing the potential transmission path of alleles from founders to CC strains, an expression can be derived for the probabilistic distribution of the unphased CC genotype, entirely in terms of known quantities. That is, we can derive an expression for the unphased CC genotype at each founder variant position, in terms of known unphased founder genotype probabilities from sequencing, and known unphased MegaMUGA haplotyping at markers. Along the way, we can also derive the probability distributions of the CC diplotypes. The equations below are specific to a given variant; they need to be recalculated for every founder variant and CC strain combination.

First, note that the unphased CC genotype probability distribution can be defined in terms of that of the phased CC genotype:

$$p\left(UG_{c}=\left\{a,a^{\prime }\right\}\right)=p\left(G_{c}=\left(a,a^{\prime }\right)\right)+p\left(G_{c}=\left(a\prime ,a\right)\right),$$ (1)

where $UG_{c}$ is the unphased genotype of CC strain *c*, $\left\{a,a^{\prime }\right\}$ is an unphased genotype with (possibly identical) alleles *a* and $a^{\prime },$ $G_{c}$ is the phased genotype of CC strain *c*, and $\left(a,a^{\prime }\right)$ is a phased genotype such that *a* is inherited maternally and $a^{\prime }$ is inherited paternally.

The probability of the (typically unobservable) phased CC genotype can be expressed in terms of phased founder diplotype as:

$$p\left(G_{c}=\left(a,a^{\prime }\right)\right)=\sum_{\left(h,h^{\prime }\right)\in \;H\;\times \;H}p\left(G_{c}=\left(a,a^{\prime }\right)|D_{c}=\left(h,h^{\prime }\right)\right)\cdot p\left(D_{c}=\left(h,h^{\prime }\right)\right),$$ (2)

where $D_{c}$ is the phased diplotype of *c* at the variant, $\left(h,h^{\prime }\right)$ describes the phased diplotype composed of (possibly identical) haplotypes *h* and $h^{\prime },$ which transmit alleles *a* and $a^{\prime }$ respectively, and H is the set of all haplotypes.

Assuming maternal and paternal alleles are transmitted independently, the first term in the product of the right hand side of (2) can be expressed as:

$$p\left(G_{c}=\left(a,a^{\prime }\right)|D_{c}=\left(h,h^{\prime }\right)\right)=p\left(T_{h}=a\right)\cdot p\left(T_{h^{\prime }}=a^{\prime }\right),$$ (3)

where $T_{h}$ is the allele transmitted from parental haplotype *h*. $T_{h}$ in turn depends on the *founder h* genotype, which is uncertain due to potential sequencing error, and on the number of *a* alleles in that founder genotype, which could be zero (homozygous for the minor allele), two (homozygous for the major allele), or even one (heterozygous) as some loci are not fully inbred in the founders. Assuming transmission of either copy is equally likely,

$$p\left(T_{h}=a\right)=\sum_{g\in G}\left(\frac{1}{2}\text{number}\;\text{of}\;a\;\text{alleles}\;\text{in}\;g\right)\cdot p\left(UG_{h}=g\right),$$ (4)

where $UG_{h}$ is the unphased genotype for the founder of haplotype *h*, G = the set of all possible genotypes and *g* is a genotype.

The second term in the product within Equation (2), $p\left(D_{c}=\left(h,h^{\prime }\right)\right),$ also needs to be derived in terms of known values: we do not know the probability of a given diplotype at any arbitrary variant position. Rather, we *do* know diplotype probabilities of the markers to the left and right of each variant position. As such, we can linearly interpolate between the two markers:

$$p\left(D_{c}=\left(h,h^{\prime }\right)\right)=\frac{w_{l}\cdot p\left(D_{\left(c,l\right)}=\left(h,h^{\prime }\right)\right)+w_{r}\cdot p\left(D_{\left(c,r\right)}=\left(h,h^{\prime }\right)\right)}{w_{l}+w_{r}},$$ (5)

where $w_{l}$ and $w_{r}$ are the distances from the variant position to the left-nearest and right-nearest haplotyping markers, respectively. $D_{\left(c,l\right)}$ and $D_{\left(c,r\right)}$ are the phased diplotypes of the cc strain at the left-nearest and right-nearest haplotyping markers, respectively.

**Figure 2:**
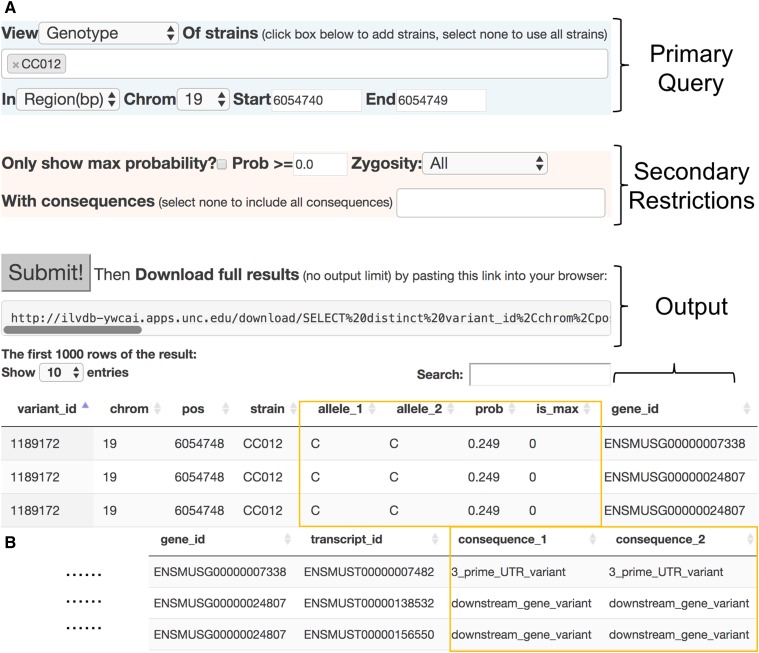
(A) Example workflow of the Inbred Strain Variant Database (ISVdb) online graphical user interface (GUI). A user has queried the genotype of CC012, on chromosome 19, from 6054740:6054749. The “Primary Query” panel also allows additional strains, and/or specification of the region by genes instead. The user is interested in all zygosity variants, of all consequences, and all probabilities, and thus has applied no secondary restriction. After the user clicked “Submit!,” a URL to download the resulting table was generated, as well as an online version of the table. The first three rows of the table are shown here: noticeably they all represent the same variant in the same strain. The difference between the rows is highlighted in the yellow box: the genotype per row and its associated probability. Collectively, the three rows represent that there is a 25% probability of a C/C genotype, 25% of T/T, and 50% of T/C at this variant in CC012. (B) The remaining wrapped columns of output from part (A) (A was too wide). Note that each genotype has a different consequence, accentuating that only accounting for the most likely genotype would cause a nonnegligible loss of information. Also, note that this figure was pieced together from a screen capture to fit on a single page.

Equation (5) is expressed in terms of phased diplotypes, but our observed MegaMUGA marker diplotype probabilities are unphased. If we assume that both phasings are equally likely, then for the left marker (similarly for the right),

$$p\left(D_{\left(c,l\right)}\right)=\frac{1}{2}p\left(UD_{\left(c,l\right)}\right)$$ (6)

where UD is the unphased diplotype. The probabilities of each unphased diplotype are known from microarray assays of the CC strains.

This concludes the derivation: using (1)–(6), the unphased CC genotype distribution, $p\left(UG_{c}\right),$ can be fully expressed from known unphased founder genotype distribution $p\left(UG_{h}\right)$ and known unphased CC marker diplotype distributions $p\left(UD_{\left(c,m\right)}\right).$

### Genotype and diplotype inference for simulated F1 offspring

Once CC genotype/diplotype probabilities have been inferred, unphased genotype/diplotype probabilities for F1 offspring between CC strains and/or founder strains can be inferred as well. As in the previous derivation, we can begin by expressing unphased genotype in terms of phased genotype:

$$p\left(UG_{12}=\left\{a,a^{\prime }\right\}\right)=p\left(G_{12}=\left(a,a^{\prime }\right)\right)+p\left(G_{12}=\left(a^{\prime },a\right)\right),$$ (7)

Where $UG_{12}$ is the unphased genotype of the offspring of inbred strain 1 and inbred strain 2. $G_{12}$ is the phased genotype.

Each phased genotype can then be rewritten in terms of the alleles transmitted from its parent strains. Assuming transmission of an allele from strain 1 is independent of transmission of an allele from strain 2,

$$p\left(G_{12}=\left(a,a^{\prime }\right)\right)=\left[p\left(T_{1}=a\right)\cdot p\left(T_{2}=a^{\prime }\right)+p\left(T_{1}=a^{\prime }\right)\cdot p\left(T_{2}=a\right)\right]/2$$ (8)

Combining (7), (8), and (4), the unphased offspring genotype distribution can be expressed in terms of known quantities. Diplotype probabilities for F1 offspring can be derived nearly identically.

### Functional consequence inference

Functional consequences per variant, and per transcript, have been predicted in the founder strains by the variant effect predictor (McLaren *et al.* 2010). We assume that any CC strain inheriting a founder’s haplotype inherits the same transcripts and functional consequences as were in the founder in that haplotype region. This assumption, that the genetic background will not affect functional consequence, is not necessarily true; if a recombination event were to occur within a gene (joining the sequence of two different founders into the same gene in a CC strain) and was upstream of some variant, that variant might no longer have the same effect. Nonetheless, our assumption is mostly reasonable: given the relatively small number of recombinations per CC line—on average 135 (Srivastava *et al.* 2017)—the number of midgene recombinations is necessarily small, and an even smaller number of these recombinations will actually have an effect on downstream variants. What might in fact be problematic to functional prediction within the CC, more so than within-gene recombination, is the original process by which functional consequences were predicted in the founder strains: predictions were made a single variant at a time, without accounting for other, potentially compensatory variants within the same gene.

Of note, functional consequences in the ISVdb are represented as uncertain; *i.e.*, the probability of a given CC genotype is also applied to the functional consequence of that genotype.

### Database and GUI implementation

Scripts to parse VCF and haplotype files, perform genotype and diplotype inference, and store the resulting processed information in a MariaDB database, were implemented using a combination of Python, VCFtools (Danecek *et al.* 2011), and R (R Core Team 2015). The ISVdb online GUI was implemented using the Python Flask library. The GUI was deployed online on the Carolina Cloud Apps managed platform, provided by UNC Information Technology Services.

Two sets of tables are stored within the MariaDB database: (1) an almost completely normalized set of tables with minimal redundancy and (2) a set of prejoined, nonnormalized tables derived from the normalized table that are designed to allow the GUI (which provides for typical ISVdb use cases) to return results efficiently, using a minimum number of joins. This second set of tables was an intentional trade-off of space for time.

A few additional database optimizations of note were necessary, especially to rapidly generate probabilistic variant states for F1 crosses. In particular, the ISVdb.v1.1 “database” is actually implemented (using R code) as a collection of smaller databases, in which each smaller database represents a single chromosome. Where applicable, most tables were indexed by variant and strain. Tables sizes were reduced by dropping those variant diplotypes (and corresponding genotypes) whose probability was < 0.001. Consequently, probability distributions at some variants may not sum exactly to 1.

### Data availability

The ISVdb version corresponding to this publication is ISVdb.v1.1, uploaded on March 15, 2017. A frozen snapshot of ISVdb.v1.1, including the v1.1 website interface and the variant information it provides, will be maintained at http://isvdb.unc.edu/archive. Subsequent ISVdb versions will be housed in the archive as well. The frozen ISVdb.v1.1 contents, and the inputs used to generate those contents, are also permanently stored on Zenodo at https://doi.org/10.5281/zenodo.399474 (Oreper *et al.* 2017b). All ISVdb.v1.1 results can be recreated from Supplemental Material, File S8 and File S9, which are briefly described below, and in further detail in File S1. As subsequent versions of ISVdb are developed, their contents will also be archived (at another location) on Zenodo.

For ISVdb.v1.1: File S1 contains detailed descriptions of all supplemental files; File S2 contains marker diplotype data for CC strains; File S3 contains nonmitochondrial SNPs for founders; File S4 contains indels for founders; File S5 contains mitochondrial SNPs for founders; File S6 contains the custom format specification for these VCF files; File S7 contains BL37.75 exons (along with other genomic features); File S8 contains an ISVdb.v1.1 dump of the imputed CC diplotypes per strain and per chromosome, in CSV format; and File S9 contains an ISVdb.v1.1 dump of the imputed CC genotypes per strain and per chromosome, in CSV format.

Code used to generate the ISVdb and its GUI is available at https://github.com/danoreper/ISVdb.git (Oreper *et al.* 2017a).

## Results and Discussion

The ISVdb houses and provides GUI access to imputed probabilistic genotype and diplotype data, for all eight founders and all (as of March 24, 2016) 72 CC strains. CC allelic state is imputed at all exonic (± 100 bp) founder SNPs and indels (which can be as long 100 bp), but not at founder large structural variant positions (Morgan *et al.* 2017).

According to the ISVdb estimates of allelic state across strains, the genotype in most variants is known with high certainty (Figure S1 in File S10). Variants with uncertain genotype appear widely and evenly distributed across the genome (Figures S2–S103 in File S10). Residual heterozygosity, a key driver of uncertainty, is estimated to affect 3.1% of exonic (± 100 bp) variants overall, but can vary dramatically between strains and chromosomes; *e.g.*, the proportion of variants affected by residual heterozygosity ranges from 0 in CC003 on chr 2 to 0.38 in CC056 on chr 8 (Table S1). Note that heterozygous variants are defined as those with at least a 25% chance of continuing to segregate.

Approximately 72.6% of (the polymorphic) CC strain variants are identical to the B6 mouse reference genome (Table S1). Intronic (8.8%), downstream (4.3%), noncoding transcript (3.4%), and upstream variants (2.9%) differing with respect to B6 are the next most common mutations, while alleles expected to have a large effect, such as stop-gain (0.003%) or stop-loss (0.001%) mutations, are extremely rare (Table S2).

### Database accessibility/usability: ISVdb GUI

The intended interface to ISVdb data is through the publicly accessible ISVdb GUI, hosted at http://isvdb.unc.edu; the GUI allows what we believe to be the most common types of queries.

The ISVdb GUI can be broken up into roughly three panels:

- A primary query panel that allows the user to query the ISVdb for: (i) inbred strain genotypes, diplotypes, and F1 genotypes and diplotypes; (ii) specify strains of interest; and to (iii) specify the genomic region(s) of interest; by basepair window, by genes, or by (internal ISVdb) variant IDs.
- A secondary restriction panel that allows the user to limit results: (i) to the maximum probability estimate of variant state; (ii) and/or only to that variant state which is more likely than a user-specified probability threshold; (iii) to variants of a particular zygosity; and (iv) for genotype and genotype cross queries, to variants having particular functional consequences.
- An output panel that allows the user to: (i) submit the primary query with secondary restrictions; (ii) save the full results by opening a download URL in a browser; and (iii) examine the (first 1000) results in a sortable and searchable table displayed online.

Additionally, the GUI provides: (i) a link to complete archived versions of the ISVdb and (ii) a link to CSV dump files of genotype and diplotype, per strain, per chromosome.

### GUI-based genotype query

The ISVdb is most typically used to determine the genotype of a set of CC strains in some region. When the ISVdb GUI is queried for genotype, it produces a table with the following columns:

variant_id: an internal ISVdb variant ID per variant.
chrom: chromosome of variant.
pos: variant start position, in mm10 coordinates.
strain: the inbred strain– either a CC or a founder strain.

The unphased genotype (allele_1 and allele_2 are arbitrary):

allele_1: the sequence of one allele at the variant.
allele_2: the sequence of the other allele at the variant.
prob: the probability that at this variant, this is the actual genotype. Note that at any given variant position there is a distribution of possible genotypes; as such there will be multiple rows representing each variant position, each with its own probability.
is_max: whether this is the maximum likelihood genotype at this variant.
gene_id: the Ensembl ID of a gene enclosing the variant. There may be multiple overlapping genes enclosing a variant, resulting in a separate row per gene for the same variant.
transcript_id: the Ensembl ID of a transcript enclosing a variant. A single variant will usually be enclosed by multiple transcripts, each of which is affected differently by the variant; *i.e.*, there will different consequences per transcript, at the same variant, necessitating a separate row per transcript for the same variant.
consequence_1: the predicted consequence of allele_1 on the transcript, with respect to B6. If allele_1 is the B6 allele, the consequence is “reference”. Note that a consequence is always with respect to some transcript.
consequence_2: the predicted consequence of allele_2 on the transcript.

### Example workflow for a genotype query

We provide an example of a genotype query to illustrate a partial ISVdb workflow, and also to demonstrate how uncertainty is represented in the ISVdb by storing multiple rows for a single variant in a single strain. Details are provided in the Figure 2 caption.

### Genotype queries are similar to other ISVdb queries

Genotype queries are just one of the four types of queries enabled by the ISVdb GUI. The remaining types of ISVdb queries are almost identical to a genotype query, and all of them represent uncertainty in the same manner as a genotype query. Rather than describing them exhaustively, we will emphasize how each differs from a genotype query.

- Genotype cross query: rather than accepting a list of strains, this query only accepts two strains, simulates their F1 offspring, and returns data nearly identical in structure to genotype query data, except for a second strain per record.
- Diplotype query: there is no notion of a functional consequence with regard to a diplotype, thus, diplotype queries return neither functional consequences nor transcript IDs, which are closely tied to functional consequence in the ISVdb. Additionally, instead of records with (allele1, allele2) genotype, a diplotype query returns records with (haplotype1, haplotype2) diplotypes.
- Diplotype cross query: just like the genotype cross query, this query accepts two strains as input and simulates their F1. It returns diplotype data that is nearly identical in structure to that from a diplotype query.

### Incorporation of CC Sequencing and up-to-date CC genotyping

The basis for the current version of ISVdb variant calls is integration of MegaMUGA genotyping data from the MRCAs of each CC strain with the sequencing data from a single mouse per founder strain (Keane *et al.* 2011; Fu *et al.* 2012; Srivastava *et al.* 2017) Consequently, the ISVdb currently has important limitations regarding completeness, heterozygosity, and imputation precision. With regard to completeness, in the generations since the MRCAs, additional mutations have accumulated and, thanks to the extremely small effective population size within each CC strain, rapidly become fixed. Sequencing results suggest that the number of variants now segregating in the CC has increased by 2% since the MRCA generation. (Srivastava *et al.* 2017). Similarly, many of the regions harboring residual heterozygosity in the MRCA animals have by now become fixed. Regarding the precision of the imputation itself, the ability to construct the underlying haplotype mosaic is limited by, among other things, the resolution of the MegaMUGA genotyping array (or of any array), and particularly how well that array can mitigate the inherent difficulties arising with inferring haplotype state at recombination breakpoints and regions of identity-by-descent between founders. Our imputed variants thus reflect an incomplete and uncertain view of the current generation’s CC genomes.

To address these limitations and gain a deeper understanding of the CC population, finer resolution data from a more recent breeding generation has been collected: the genome of a single male per CC strain has been sequenced (Srivastava *et al.* 2017), and this will not only identify the *de novo* CC mutations but also reduce the uncertainty in CC strain genotypes at known variants. Nonetheless, since sequencing is limited to a single animal per strain, it does not by itself provide a definitive answer genome-wide, largely due to residual heterozygosity. In the near future, a set of three other males per strain from the sequencing generation will be genotyped on the MUGA platform (Fernando Pardo-Manuel de Villena, personal communication).

The ISVdb will progressively incorporate these new sets of data into its variant representation according to the following release schedule:

IDVdb.v1.1 (March 15, 2017): the current ISVdb version, corresponding to this publication.
ISVdb.v1.2 (∼June 2017): inclusion of the whole genome (rather than exons ± 100 bp as in ISVdb.v1.1), and the incorporation of the latest MGP sequencing of the founder panel (REL-15.04).
ISVdb.v2.0 (∼July 2017): inclusion of sequenced *de novo* variants as new records in the ISVdb. This depends on projected availability of the sequencing data, which is expected in April 2017.
ISVdb.v2.1 (∼Sep 2017): inclusion of a first pass at integration of sequencing and same-generation MUGA genotyping, to better impute state at known (rather than *de novo*) variants: where sequencing and MUGA variant calls are inconsistent, ISVdb probabilities will be some sort of weighted sum of the data sources. Depends on projected availability of MUGA genotyping data, expected in July 2017.
ISVdb.v3.0 (∼December 2017): (a) a more sophisticated method of modeling the inconsistencies between and among genotyping and sequencing data to arrive at better estimates of genotype uncertainty and residual heterozygosity; (b) this may be coupled with reestimation of the founder genotypes themselves using CC sequencing data, which indirectly provide us with ultradeep founder sequencing and could be thought of as ancestral variant imputation; and (c) inclusion of indels/SNPs for the whole genome rather than only for exons.
ISVdb.v3.1 (∼February 2018): inclusion of structural variants (> 100 bp) and submission of a follow-up publication describing the latest developments.

As the reference genome and its exon annotations changes, the ISVdb will be updated as well.

In addition to updating the ISVdb with more accurate state, incorporation of the newest high-resolution data will allow a useful comparison between generations. In particular, detection of loci that have become fixed since the MRCA generation will open a new line of inquiry as to the possible selective advantage of the newly fixed alleles.

In summary, we have developed a database that stores imputed probabilistic variant state for CC strains and founder strains, and can rapidly generate probabilistic variants states for F1 populations. Imputed state includes alleles as well as predicted functional consequences of those alleles. This resource is a useful complement to sequencing data, and is easily accessible at http://isvdb.unc.edu.

## Supplementary Material

Supplemental material is available online at http://www.g3journal.org/lookup/suppl/doi:10.1534/g3.117.041491/-/DC1.
