# Supplementary material for: Inbred Strain Variant Database (ISVdb): A Repository for Probabilistically Informed Sequence Differences Among the Collaborative Cross Strains and Their Founders
Source: G3 (Bethesda). 2017 Jun 5;7(6):1623–30. doi: 10.1534/g3.117.041491 (PMC5473744; doi:10.1534/g3.117.041491)
Supplement: Supplementary file 1 [file 1623FileS1.docx]

**Inbred Strain Variant Database (ISVdb): A repository for probabilistically informed sequence differences among the Collaborative Cross strains and their founders**

Daniel G Oreper, Yanwei Cai, Lisa M Tarantino, Fernando Pardo Manuel de Villena, and William Valdar

All files/figures/tables except for the github code repository are in Zenodo, at https://doi.org/10.5281/zenodo.399474

File_S1 (this file): ISVdb data documentation.

**Raw genotype/marker information**

File_S2.tar.gz:contains marker diplotype data for each CC line, as downloaded 2016-03-24.

Each file is named according to the represented CC line: CCXXX_[Geni]Uncb38V01.csv,

where XXX is a 3 digit CC line number. These marker files were generated from MegaMUGA microarray data, in an HMM based analysis by Leonard McMillan's group within UNC Systems Genetics. The latest versions of these marker files (i.e., 36-founder-state probabilities) are also available at: <http://csbio.unc.edu/CCstatus/CCGenomes/>

Each row in a CC marker file represents a marker.

The fields as follows.

-marker: the marker name.

-chromosome: the marker chromosome.

-position(B38): the marker basepair position in BL38 coordinates.

-AA, BB, ..., HH, ..., AB, AC, ..., GH: A set of columns describing the probability of each diplotype at the marker. There are 28 potential diplotypes.

Each letter A-H is short for a CC founder haplotype:

A=A/J, B=C57BL/6J, C=129S1Sv/ImJ, D=NOD/ShiLtJ, E=NZO/H1LtJ, F=CAST/EiJ, G=PWK/PhJ, and H=WSB/EiJ.

File_S3.gz: VCF file containing the non-mitochondrial snps for all the CC founders, A-H.

Originally downloaded from ftp://ftp-mouse.sanger.ac.uk/REL-1410-SNPs_Indels/mgp.v4.snps.dbSNP.vcf.gz

File_S4.gz: VCF file containing the indel variants for all the CC founders, A-H

Originally downloaded from ftp://ftp-mouse.sanger.ac.uk/REL-1410-SNPs_Indels/mgp.v4.snps.MT.dbSNP.vcf.gz

File_S5.gz: VCF file containing the MT snps for all the CC founders, A-H

ftp://ftp-mouse.sanger.ac.uk/REL-1410-SNPs_Indels/mgp.v4.indels.dbSNP.vcf.gz.md5

File_S3, File_S4 and File_S5 VCF files were all generated by the Mouse Genome Project (MGP) in their analysis of sequencing data.

File_S6.txt: contains the MGP specification of the MGP custom VCF format. Originally downloaded from ftp://ftp-mouse.sanger.ac.uk/REL-1410-SNPs_Indels/README

File_S7.gtf.gz: Exon data and other genomic features for BL38.75 in gtf format. Originally downloaded from <ftp://ftp.ensembl.org/pub/release-75/gtf/mus_musculus/Mus_musculus.GRCm38.75.gtf.gz>

**Results files**

File_S8.tar.gz: A dump of imputed diplotypes in CC founder strains, as generated by ISVdb code.

Within this tar.gz, are a set of folders in which each folder represents a strain, and within that strain folder is a collection of chromosome information. I.e.,

---StrainName1

------1.txt.tar.gz

------2.txt.tar.gz

------3.txt.tar.gz

------...

------MT.txt.tar.gz

------X.txt.tar.gz

------Y.txt.tar.gz

Each such txt.tar.gz is a zipped csv with 2 header rows:

1) A row specifying the strain and chromosome of the format: strain:strainname,chr:chrname

2) The fields stored in the csv file.

The fields consist of the following:

-variant_id: a positive integer ID specifying the variant. Unique to every variant; chromosomes don't share variant_id. However, this ID is shared across strains, and is consistent between the diplotype dump and the genotype dump files.

-pos: positive integer specifying variant position in bp along chromosome.

-founder_1: one of the founder haplotypes at this variant for the file-specified strain and chromosome. This is part of an unphased diplotype.

-founder_2: the second founder haplotypes at this variant for the file-specified strain and chromosome. This is part of an unphased diplotype.

-prob: the probability on [0,1] that the unphased diplotype of the variant at this position is (founder_1, founder_2)

-gene_name: the name of a gene enclosing the variant.

Note that there may multiple records per variant if the diplotype of the variant is uncertain; in such a case there is one row per non-zero diplotype probability, and the probabilities add approximately to 1. There also may be multiple rows per variant if a variant is enclosed by more than one gene.

File_S9.tar.gz: A dump of imputed genotypes in CC founder strains, as generated by ISVdb code.

Within this tar.gz are a set of folders in which each folder represents a strain, and within that strain folder is a collection of chromosome information. I.e.,

---StrainName1

------1.txt.tar.gz

------2.txt.tar.gz

------3.txt.tar.gz

------...

------MT.txt.tar.gz

------X.txt.tar.gz

------Y.txt.tar.gz

Each such txt.tar.gz is a zipped csv with 2 header rows:

1) A row specifying the strain and chromosome of the format: strain:strainname,chr:chrname

2) The fields stored in the csv file.

The fields consist of the following:

-variant_id: a positive integer ID specifying the variant. Unique to every variant; chromosomes don't share variant_id. However, this ID is shared across strains, and is consistent between the diplotype dump and the genotype dump files.

-pos: positive integer specifying variant position in bp along chromosome.

-allele_1: one of the alleles at this variant for the file-specified strain. This is part of an unphased genotype.

-allele_2: the second alelle at this variant for the file-specified strain. This is part of an unphased diplotype.

-prob: the probability that the unphased diplotype of the variant at this position is (allele_1, allele_2)

-is_max: whether this is the max likelihood genotype for this variant in this strain. Redundant with prob, but available for convenience.

-gene_name: the name of a gene enclosing the variant.

Note that there may multiple records per variant if the diplotype of the variant is uncertain; in such a case there is one row per non-zero diplotype probability, and the probabilities add approximately to 1. There also may be multiple rows per variant if a variant is enclosed by more than one gene.

-transcript_name: the name of a transcript enclosing the variant.

-consequence_1: the functional consequence of allele_1 on transcript_name, with respect to the B6 reference allele. If the allele_2 is the reference allele, the consequence is "reference".

-consequence_2: the functional consequence of allele_2 on transcript_name, with respect to the B6 reference allele. If allele_2 is the reference allele, the consequence is "reference".

Note that there may multiple records per variant if the genotype of the variant is uncertain; in such a case there is one row per non-zero genotype probability, and the probabilities add approximately to 1. There also may be multiple rows per variant if a variant is enclosed by more than one gene and/or more than one transcript, as the consequence changes depending on the transcript.

Table_S1.csv: a table describing the pct heterozygosity per chromosome, per strain

Table_S2.csv: a table describing the number of variants per functional consequence (and fraction), split per strain.

File_S10.pdf: supplemental figures.

**Software**

Github Code repository: https://github.com/danoreper/ISVdb.git
