# Supplementary material for: Inbred Strain Variant Database (ISVdb): A Repository for Probabilistically Informed Sequence Differences Among the Collaborative Cross Strains and Their Founders
Source: G3 (Bethesda). 2017 Jun 5;7(6):1623–30. doi: 10.1534/g3.117.041491 (PMC5473744; doi:10.1534/g3.117.041491)
Supplement: Supplementary file 2 [file 1623FileS10.pdf]

File S10 supplemental figures and tables

Daniel Oreper

March 28, 2017

Contents

1 Genotype probabilities **4**

2 Entropy distributions across strains and chromosomes **5**

2.1 Entropies per chromosome . . . 5

2.2 Entropies per strain . . . 28

3 Residual heterozygosity **109**

4 Predicted functional consequences counts **109**

List of Figures

1 Genotype probabilities for all strains. . . . 4
2 Chr 1, non-zero entropies . . . 6
3 Chr 2, non-zero entropies . . . 7
4 Chr 3, non-zero entropies . . . 8
5 Chr 4, non-zero entropies . . . 9
6 Chr 5, non-zero entropies . . . 10
7 Chr 6, non-zero entropies . . . 11
8 Chr 7, non-zero entropies . . . 12
9 Chr 8, non-zero entropies . . . 13
10 Chr 9, non-zero entropies . . . 14
11 Chr 10, non-zero entropies . . . 15
12 Chr 11, non-zero entropies . . . 16
13 Chr 12, non-zero entropies . . . 17
14 Chr 13, non-zero entropies . . . 18
15 Chr 14, non-zero entropies . . . 19
16 Chr 15, non-zero entropies . . . 20
17 Chr 16, non-zero entropies . . . 21
18 Chr 17, non-zero entropies . . . 22
19 Chr 18, non-zero entropies . . . 23
20 Chr 19, non-zero entropies . . . 24
21 Chr X, non-zero entropies . . . 25
22 Chr Y, non-zero entropies . . . 26
23 Chr MT, non-zero entropies . . . 27
24 Strain AJ, non-zero entropies . . . 29
25 Strain B6, non-zero entropies . . . 30
26 Strain 129, non-zero entropies . . . 31
27 Strain NOD, non-zero entropies . . . 32
28 Strain NZO, non-zero entropies . . . 33
29 Strain CAST, non-zero entropies . . . 34
30 Strain PWK, non-zero entropies . . . 35
31 Strain WSB, non-zero entropies . . . 36
32 Strain CC001, non-zero entropies . . . 37
33 Strain CC002, non-zero entropies . . . 38
34 Strain CC003, non-zero entropies . . . 39
35 Strain CC004, non-zero entropies . . . 40
36 Strain CC005, non-zero entropies . . . 41

37 Strain CC006, non-zero entropies . . . 42
38 Strain CC007, non-zero entropies . . . 43
39 Strain CC008, non-zero entropies . . . 44
40 Strain CC009, non-zero entropies . . . 45
41 Strain CC010, non-zero entropies . . . 46
42 Strain CC011, non-zero entropies . . . 47
43 Strain CC012, non-zero entropies . . . 48
44 Strain CC013, non-zero entropies . . . 49
45 Strain CC014, non-zero entropies . . . 50
46 Strain CC015, non-zero entropies . . . 51
47 Strain CC016, non-zero entropies . . . 52
48 Strain CC017, non-zero entropies . . . 53
49 Strain CC018, non-zero entropies . . . 54
50 Strain CC019, non-zero entropies . . . 55
51 Strain CC020, non-zero entropies . . . 56
52 Strain CC021, non-zero entropies . . . 57
53 Strain CC022, non-zero entropies . . . 58
54 Strain CC023, non-zero entropies . . . 59
55 Strain CC024, non-zero entropies . . . 60
56 Strain CC025, non-zero entropies . . . 61
57 Strain CC026, non-zero entropies . . . 62
58 Strain CC027, non-zero entropies . . . 63
59 Strain CC028, non-zero entropies . . . 64
60 Strain CC029, non-zero entropies . . . 65
61 Strain CC030, non-zero entropies . . . 66
62 Strain CC031, non-zero entropies . . . 67
63 Strain CC032, non-zero entropies . . . 68
64 Strain CC033, non-zero entropies . . . 69
65 Strain CC034, non-zero entropies . . . 70
66 Strain CC035, non-zero entropies . . . 71
67 Strain CC036, non-zero entropies . . . 72
68 Strain CC037, non-zero entropies . . . 73
69 Strain CC038, non-zero entropies . . . 74
70 Strain CC039, non-zero entropies . . . 75
71 Strain CC040, non-zero entropies . . . 76
72 Strain CC041, non-zero entropies . . . 77
73 Strain CC042, non-zero entropies . . . 78
74 Strain CC043, non-zero entropies . . . 79
75 Strain CC044, non-zero entropies . . . 80
76 Strain CC045, non-zero entropies . . . 81
77 Strain CC046, non-zero entropies . . . 82
78 Strain CC047, non-zero entropies . . . 83
79 Strain CC048, non-zero entropies . . . 84
80 Strain CC049, non-zero entropies . . . 85
81 Strain CC050, non-zero entropies . . . 86
82 Strain CC051, non-zero entropies . . . 87
83 Strain CC052, non-zero entropies . . . 88
84 Strain CC053, non-zero entropies . . . 89
85 Strain CC054, non-zero entropies . . . 90
86 Strain CC055, non-zero entropies . . . 91
87 Strain CC056, non-zero entropies . . . 92
88 Strain CC057, non-zero entropies . . . 93
89 Strain CC058, non-zero entropies . . . 94
90 Strain CC059, non-zero entropies . . . 95
91 Strain CC060, non-zero entropies . . . 96
92 Strain CC061, non-zero entropies . . . 97
93 Strain CC062, non-zero entropies . . . 98
94 Strain CC063, non-zero entropies . . . 99
95 Strain CC065, non-zero entropies . . . 100
96 Strain CC068, non-zero entropies . . . 101
97 Strain CC070, non-zero entropies . . . 102

98 Strain CC071, non-zero entropies . . . 103
99 Strain CC072, non-zero entropies . . . 104
100 Strain CC073, non-zero entropies . . . 105
101 Strain CC074, non-zero entropies . . . 106
102 Strain CC075, non-zero entropies . . . 107
103 Strain CC076, non-zero entropies . . . 108

1 Genotype probabilities

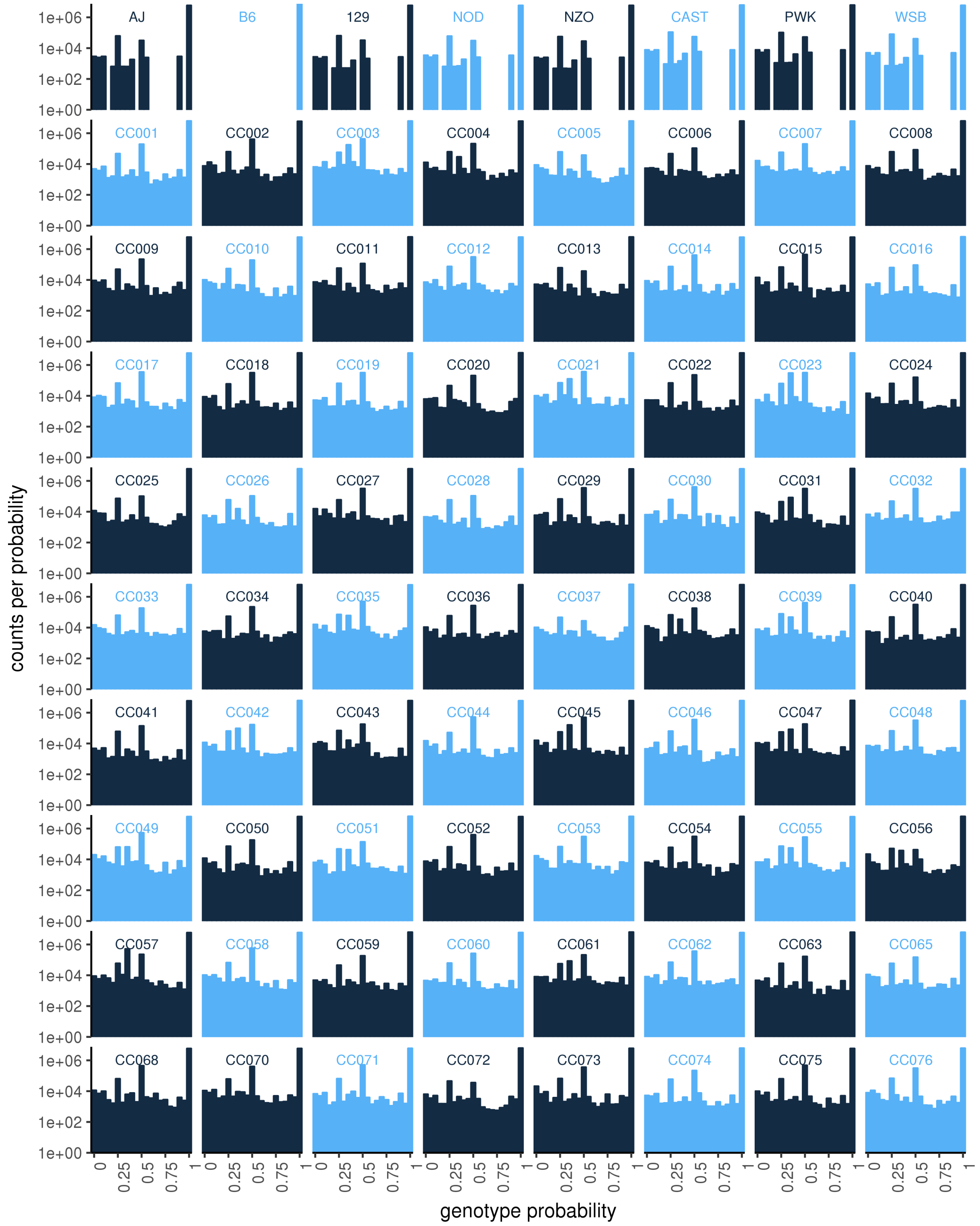

Figure S1 Each panel is the histogram of genotype probabilities imputed for a particular strain, on log scale. Most genotypes are known with full certainty, though there are spikes in occurrence of genotype probability at .25 and .5. For founders, probabilities <1 correspond to variants called as heterozygous by Mouse Genome Project sequencing, likely due to uncertain sequencing results rather than actual residual heterozygosity in the founders.

2 Entropy distributions across strains and chromosomes

Entropies of 0 are not plotted, as 0 represents perfect certainty, and exists throughout. High entropy regions correspond to low certainty. Entropy plots are generated per-strain, summarizing over all chromosomes for that single strain, and per-chromosome, summarizing over all strains for that single chromosome. Each point corresponds to the entropy in a particular strain for a particular variant.

2.1 Entropies per chromosome

Each figure corresponds to a chromosome. Each panel depicts the non-zero entropies for a strain, along that chromosome.

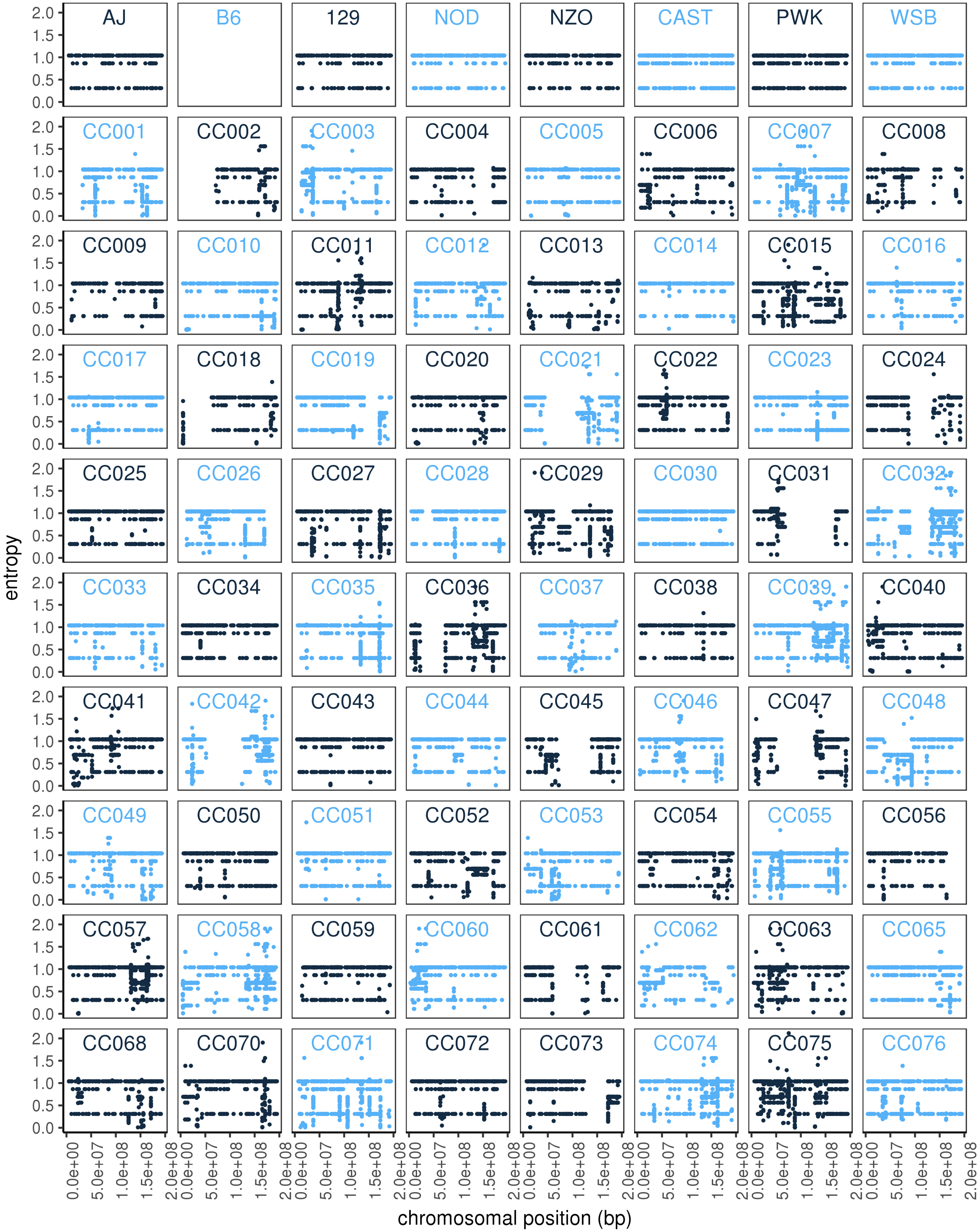

Figure S2 chr 1, non-zero entropies in exons (+/-100bp) in all strains. Each point corresponds to the entropy of a variant at that position along the chromosome

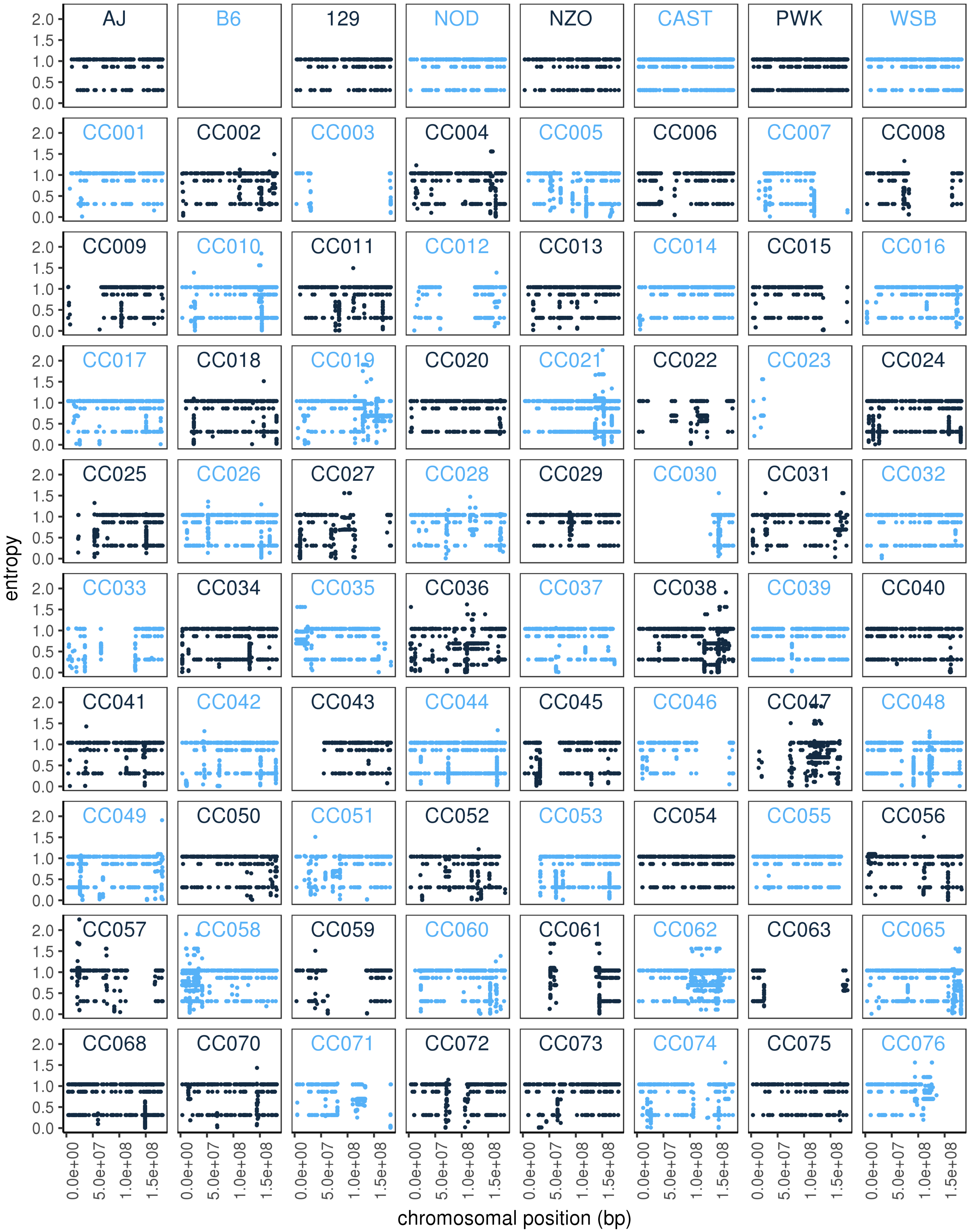

Figure S3 chr 2, non-zero entropies in exons (+/-100bp) in all strains. Each point corresponds to the entropy of a variant at that position along the chromosome

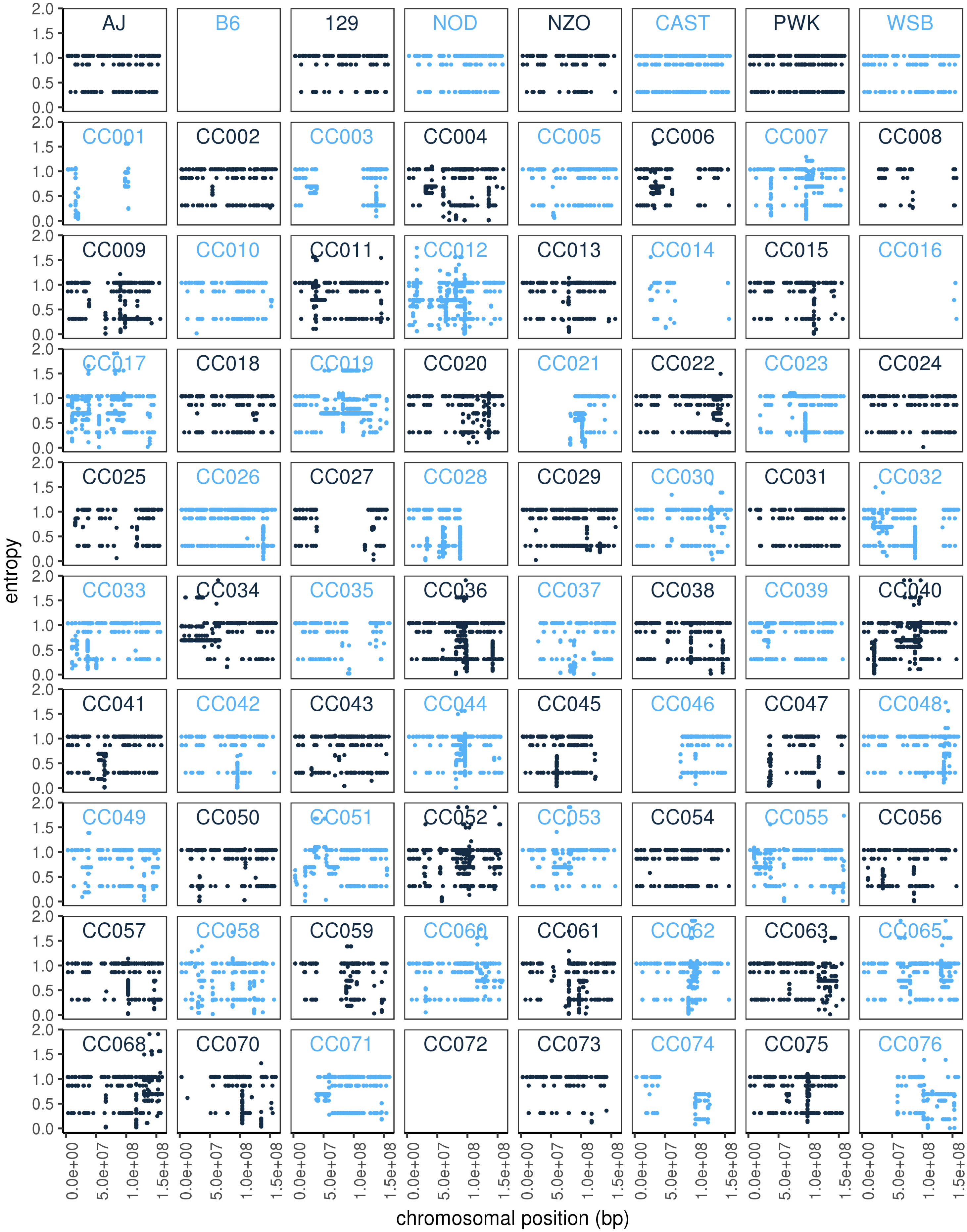

Figure S4 chr 3, non-zero entropies in exons (+/-100bp) in all strains. Each point corresponds to the entropy of a variant at that position along the chromosome

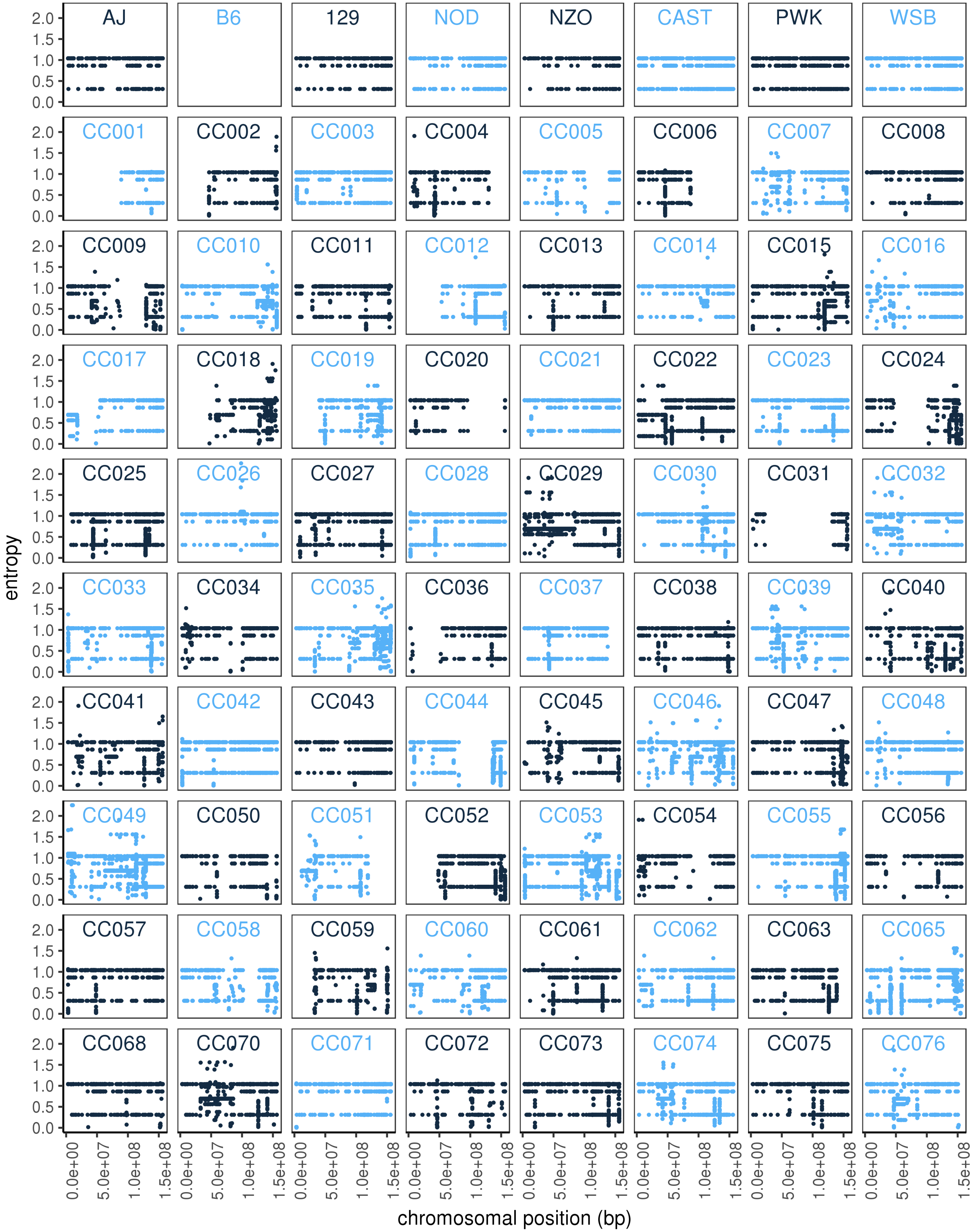

Figure S5 chr 4, non-zero entropies in exons (+/-100bp) in all strains. Each point corresponds to the entropy of a variant at that position along the chromosome

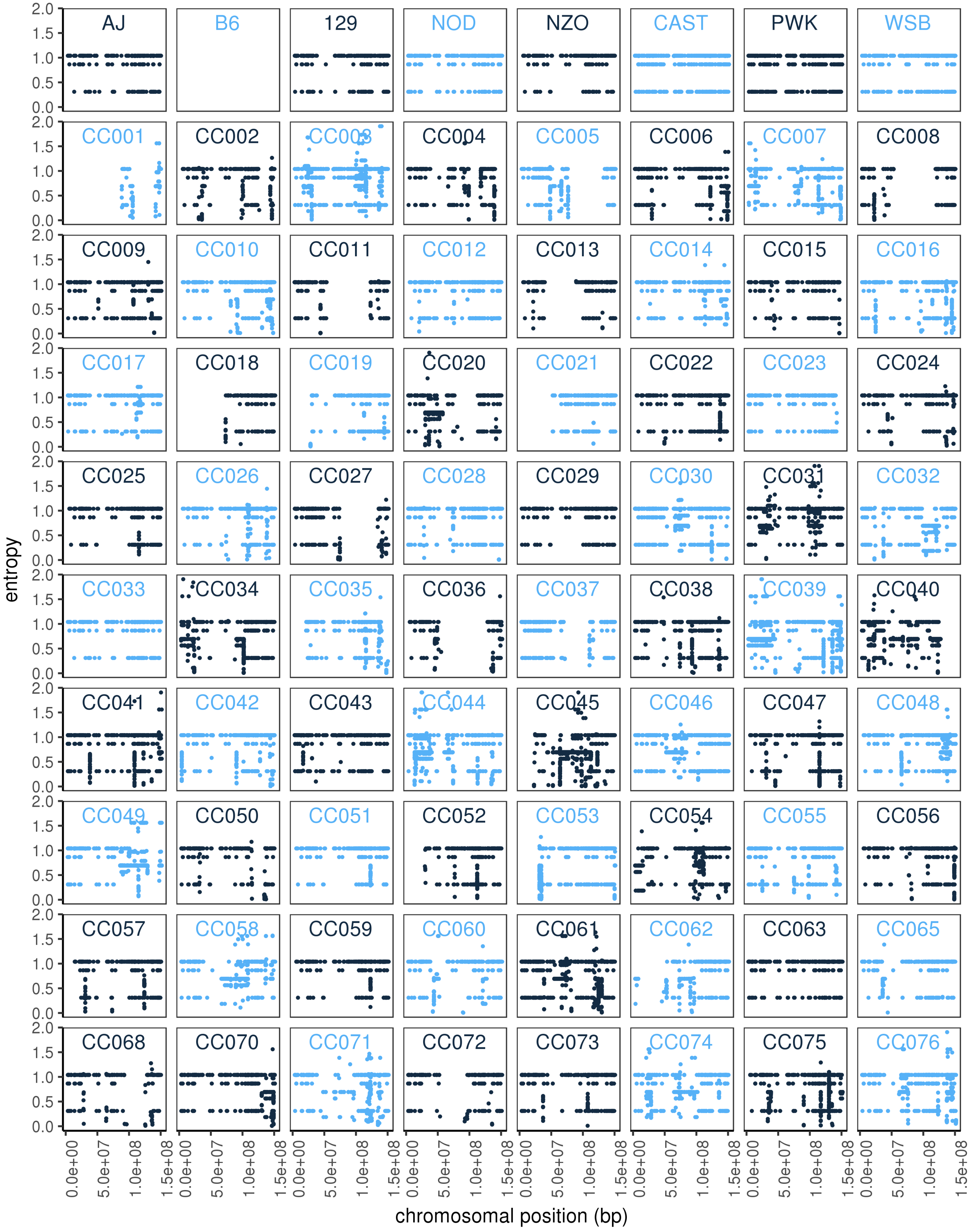

Figure S6 chr 5, non-zero entropies in exons (+/-100bp) in all strains. Each point corresponds to the entropy of a variant at that position along the chromosome

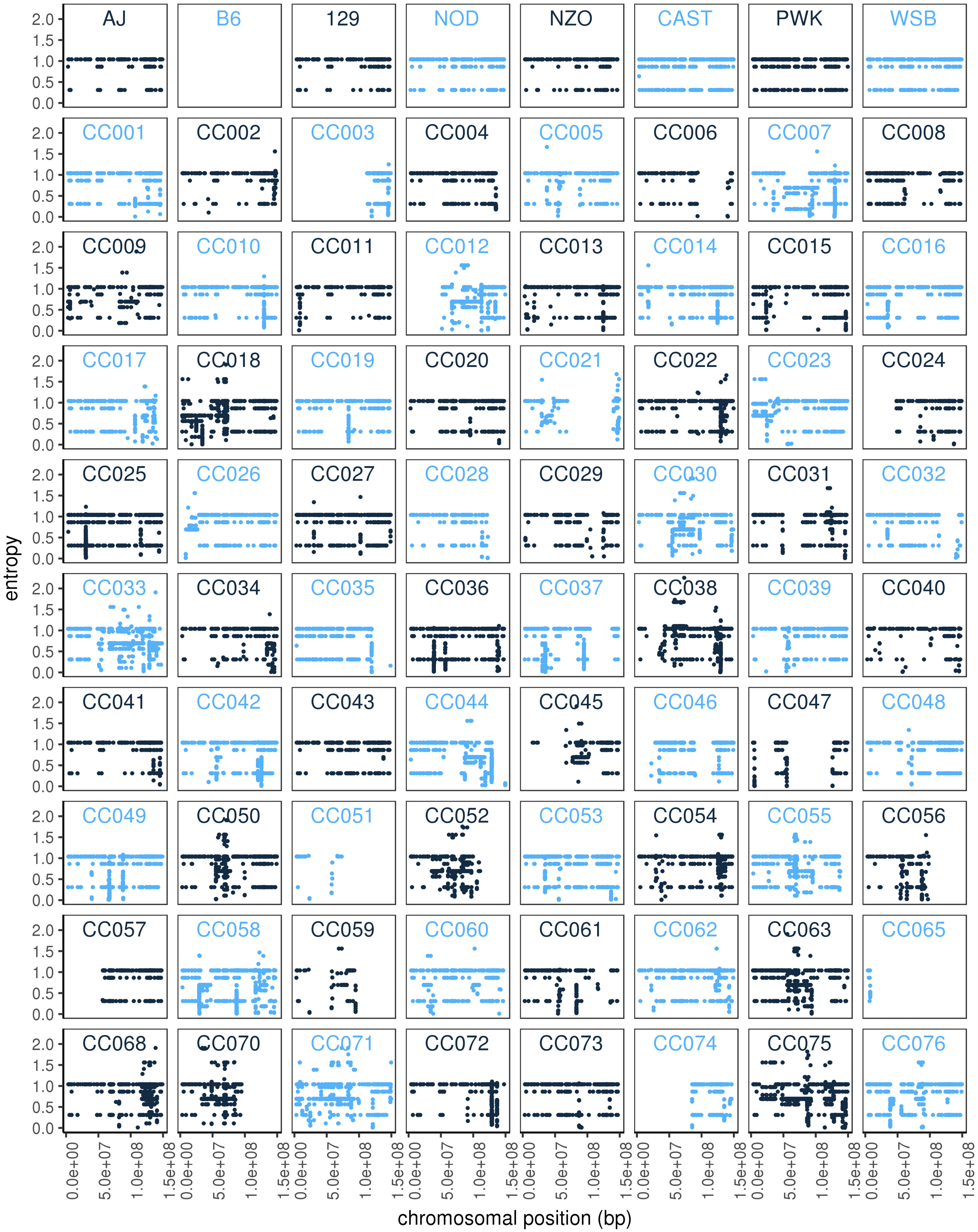

Figure S7 chr 6, non-zero entropies in exons (+/-100bp) in all strains. Each point corresponds to the entropy of a variant at that position along the chromosome

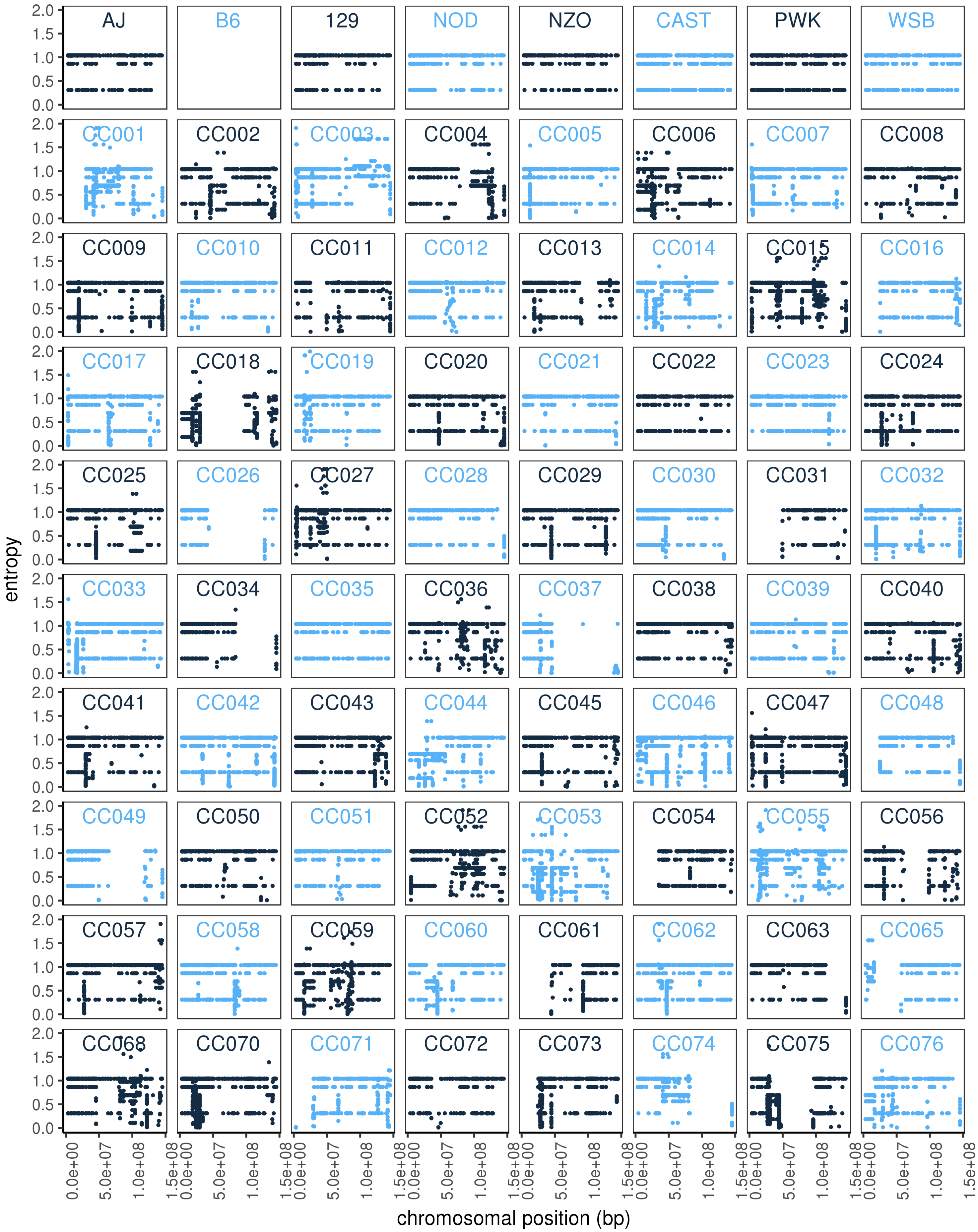

Figure S8 chr 7, non-zero entropies in exons (+/-100bp) in all strains. Each point corresponds to the entropy of a variant at that position along the chromosome

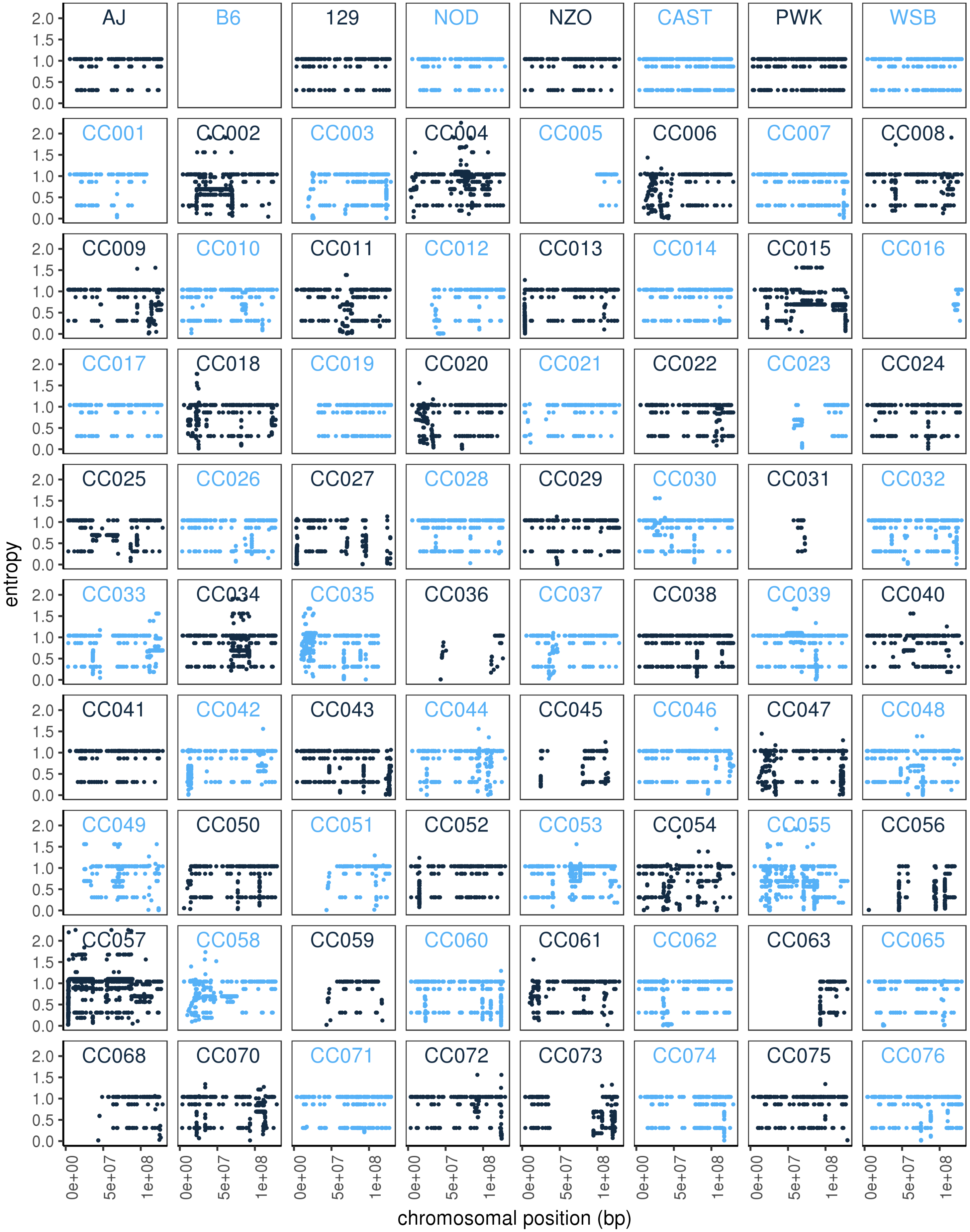

Figure S9 chr 8, non-zero entropies in exons (+/-100bp) in all strains. Each point corresponds to the entropy of a variant at that position along the chromosome

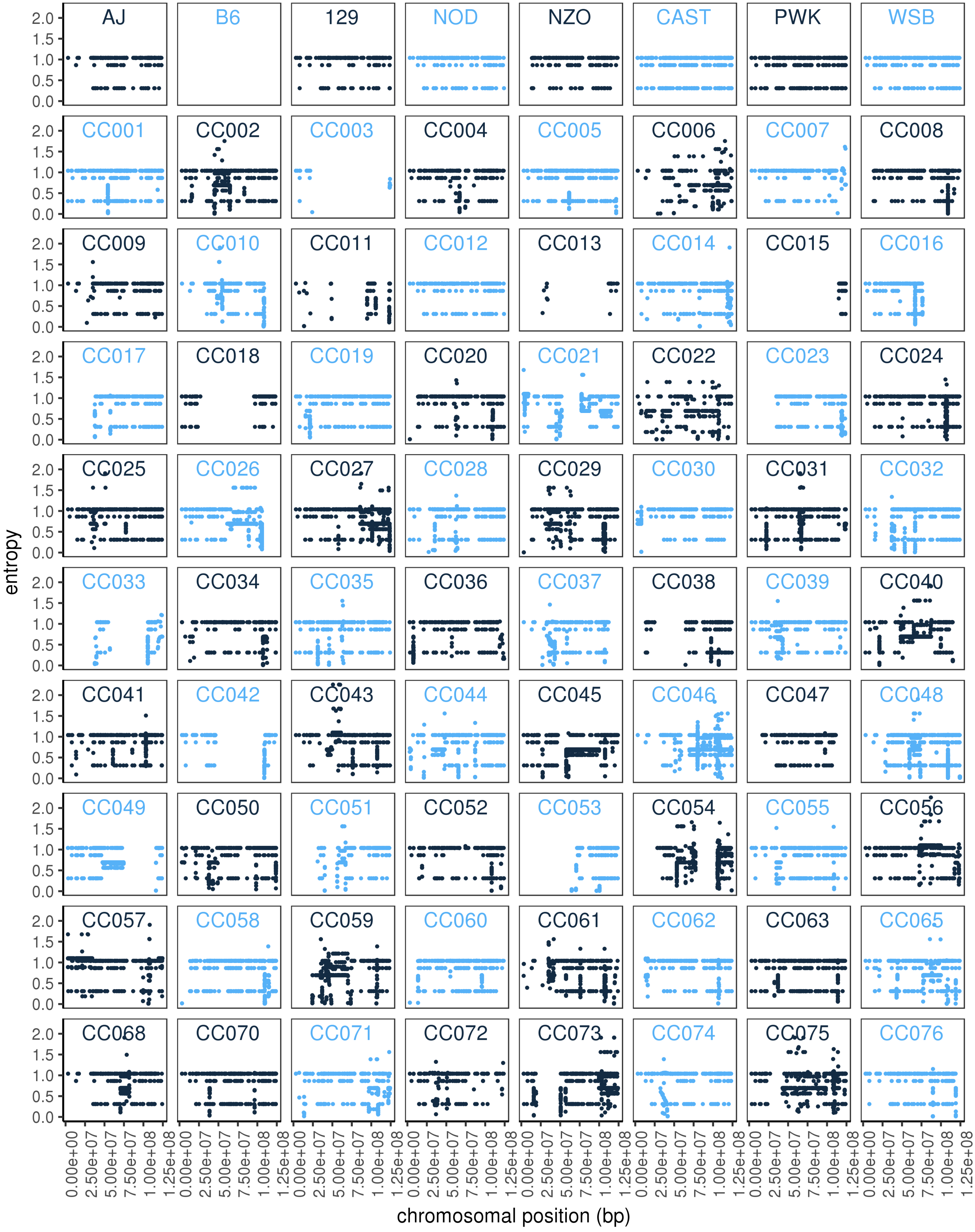

Figure S10 chr 9, non-zero entropies in exons (+/-100bp) in all strains. Each point corresponds to the entropy of a variant at that position along the chromosome

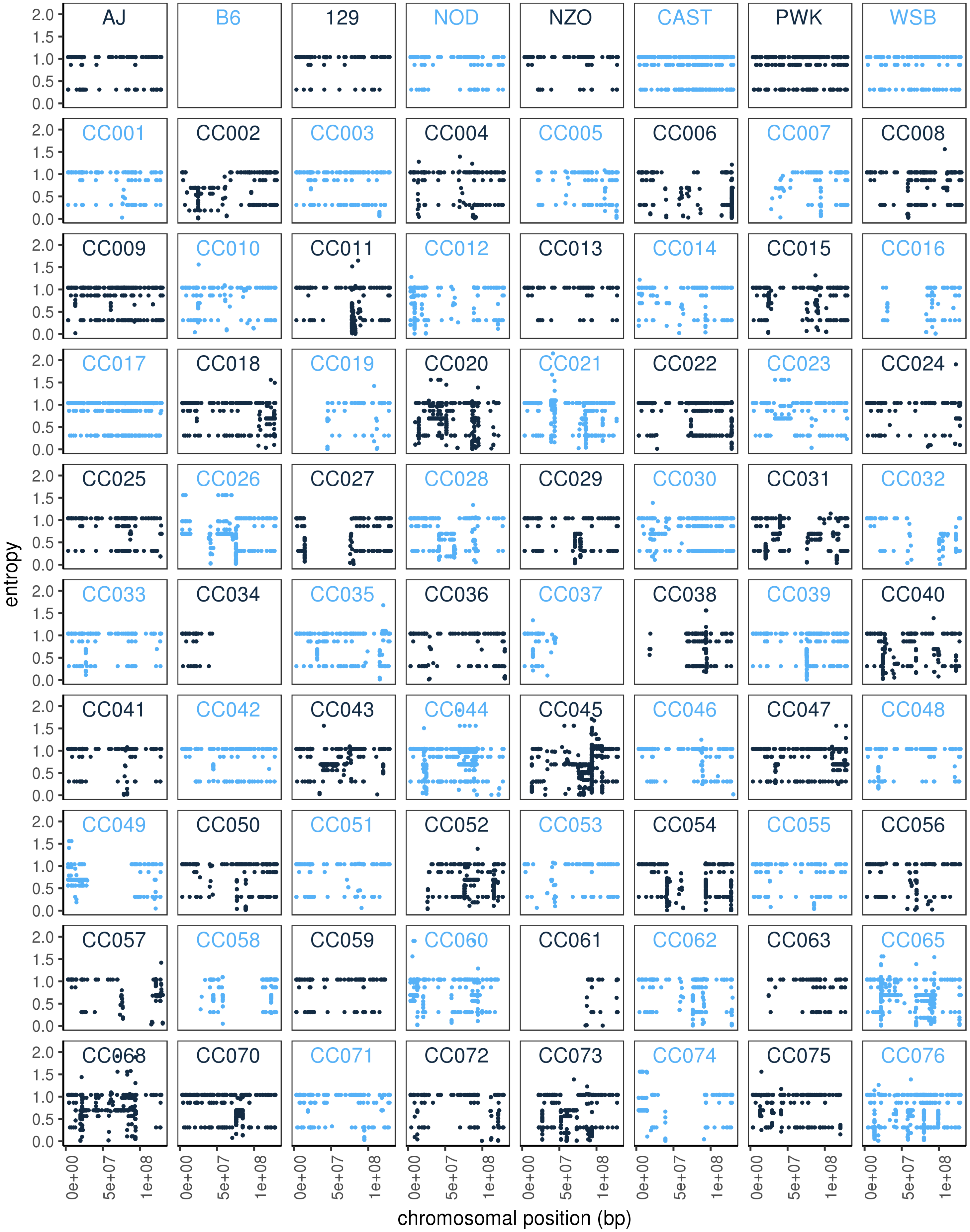

Figure S11 chr 10, non-zero entropies in exons (+/-100bp) in all strains. Each point corresponds to the entropy of a variant at that position along the chromosome

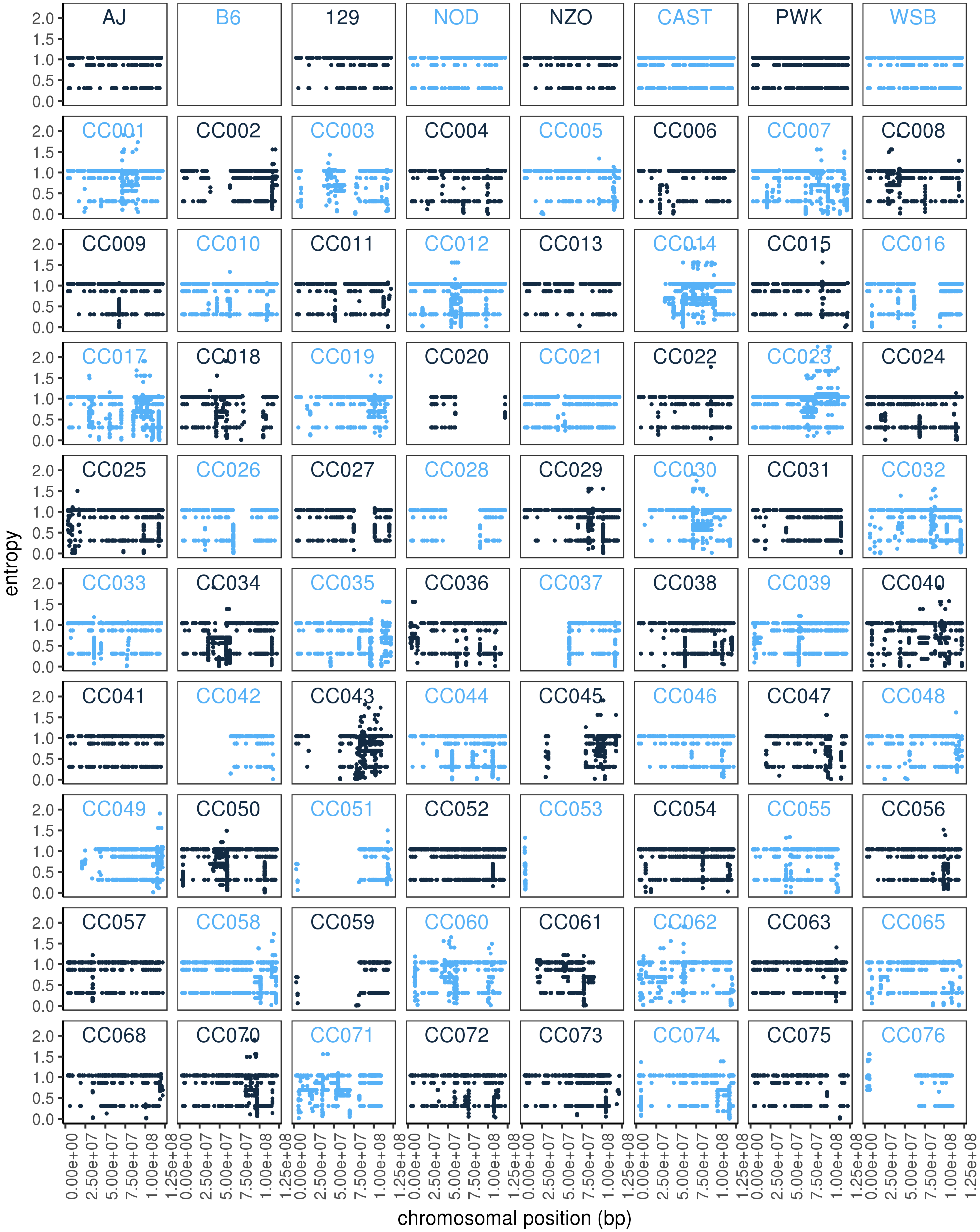

Figure S12 chr 11, non-zero entropies in exons (+/-100bp) in all strains. Each point corresponds to the entropy of a variant at that position along the chromosome

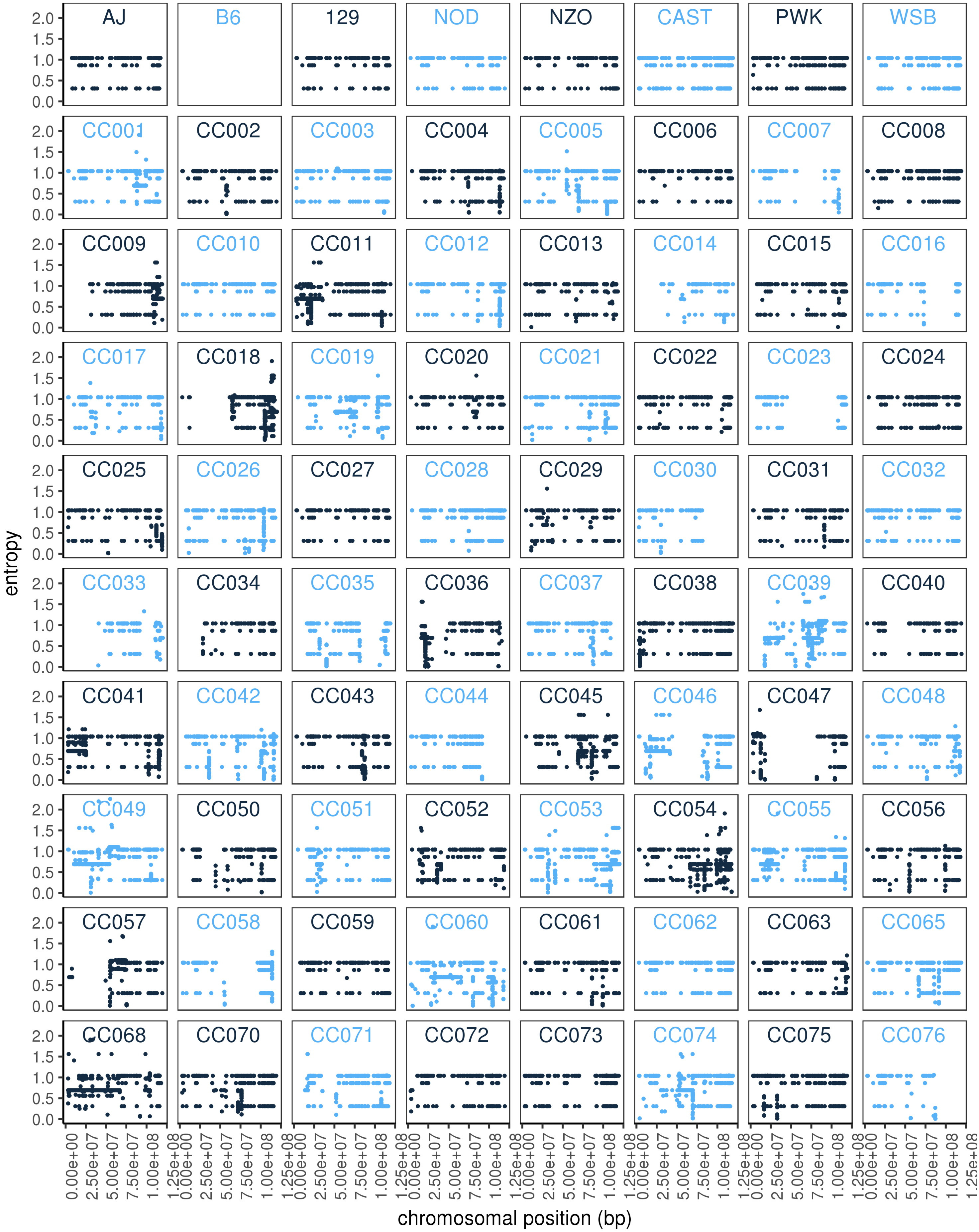

Figure S13 chr 12, non-zero entropies in exons (+/-100bp) in all strains. Each point corresponds to the entropy of a variant at that position along the chromosome

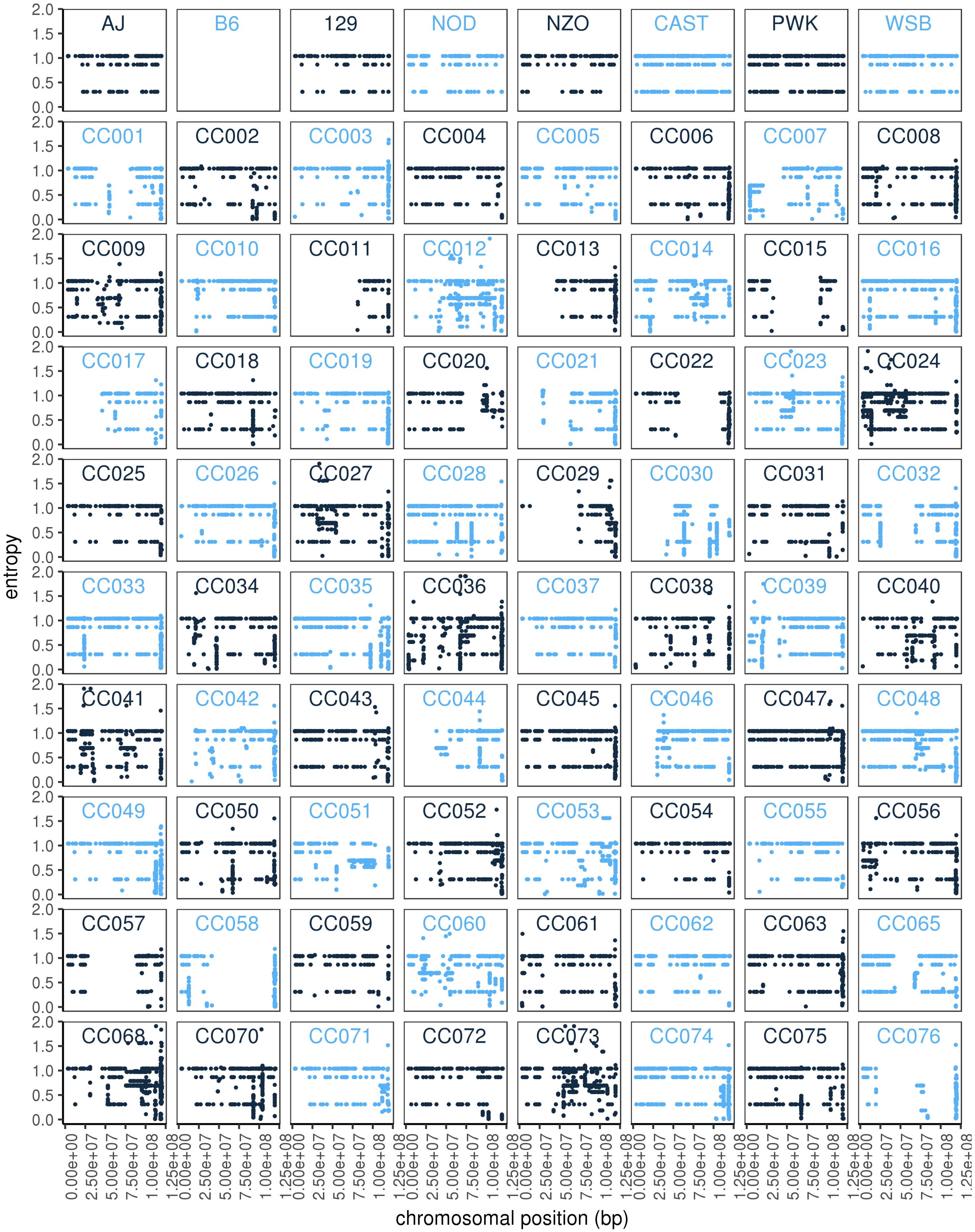

Figure S14 chr 13, non-zero entropies in exons (+/-100bp) in all strains. Each point corresponds to the entropy of a variant at that position along the chromosome

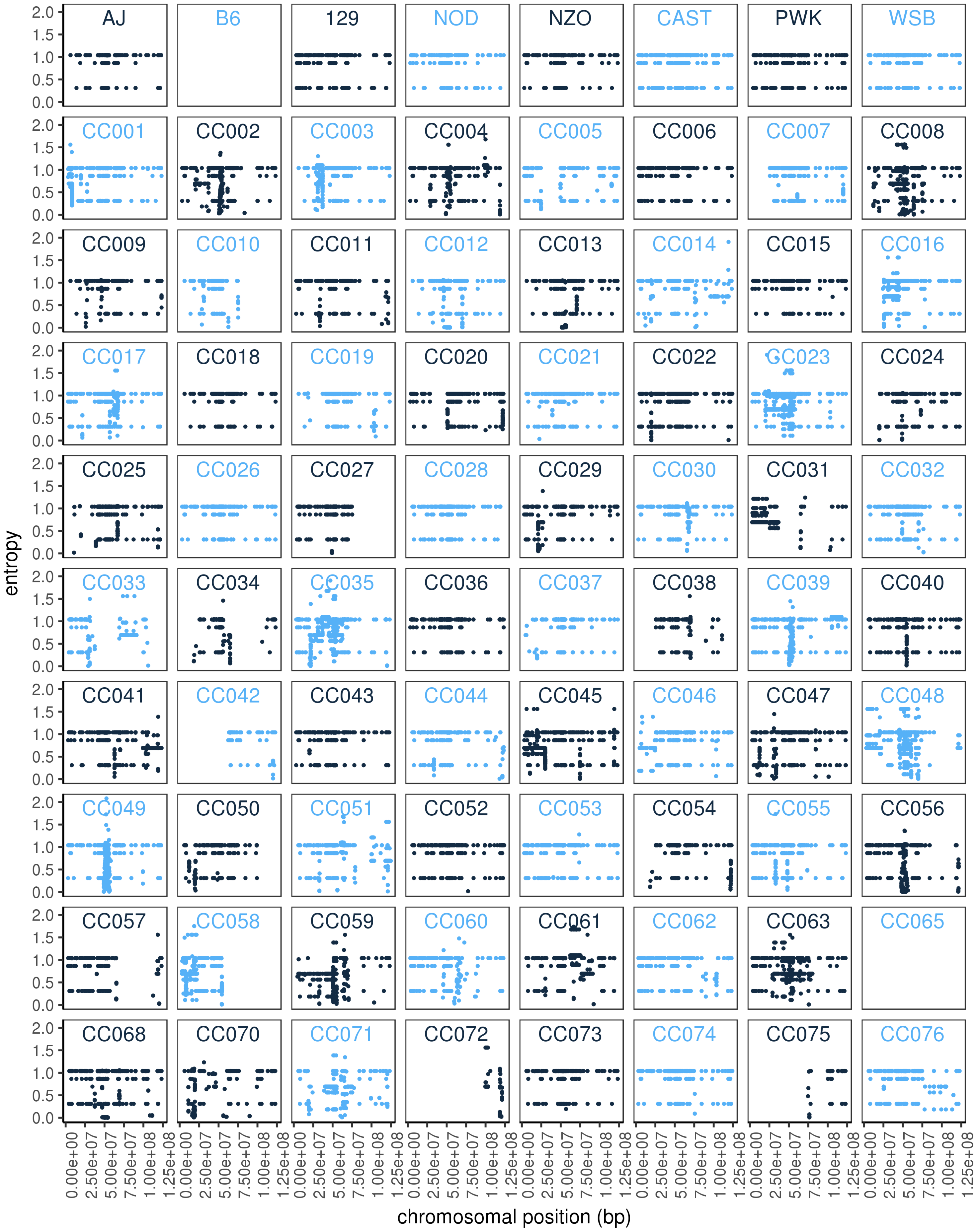

Figure S15 chr 14, non-zero entropies in exons (+/-100bp) in all strains. Each point corresponds to the entropy of a variant at that position along the chromosome

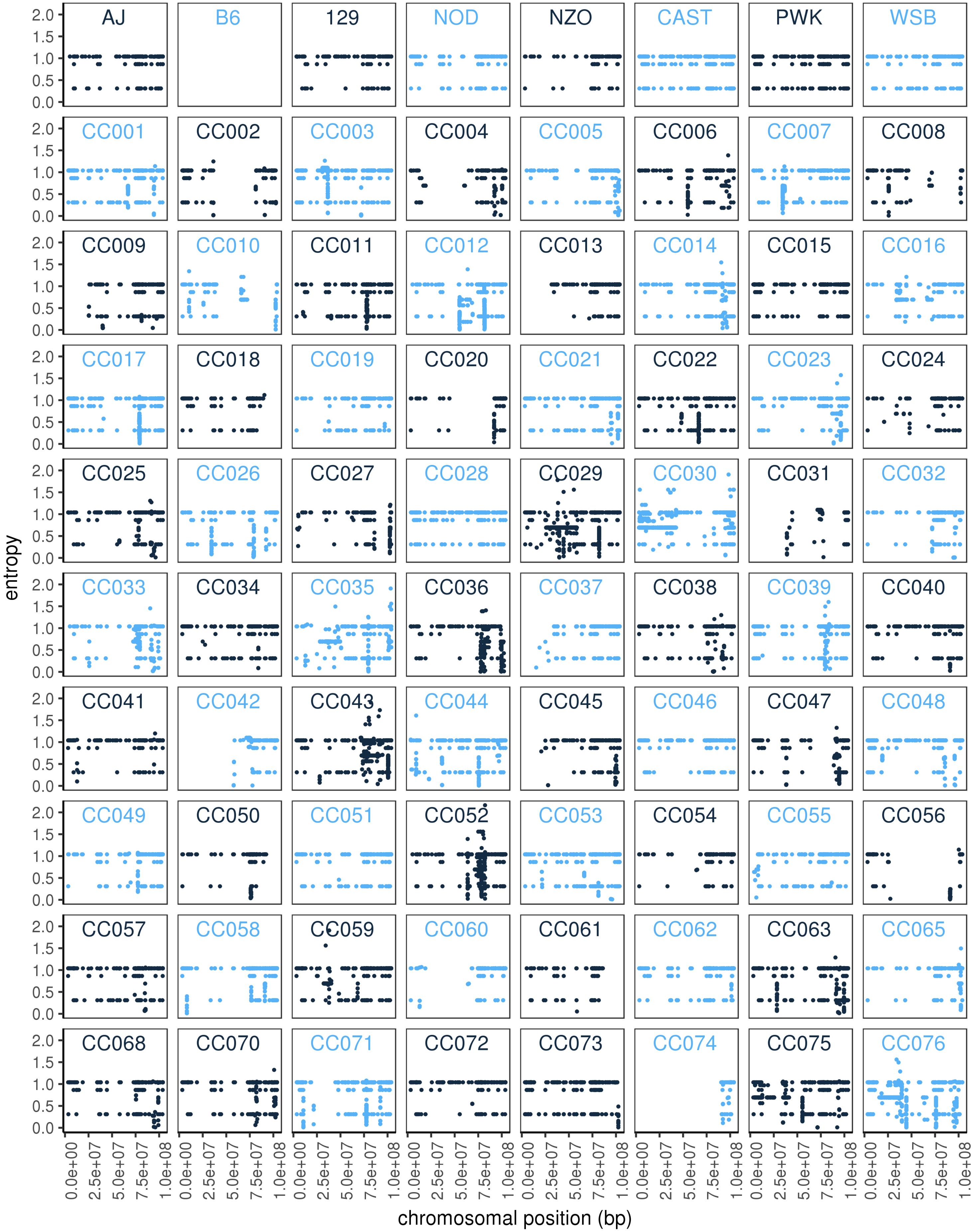

Figure S16 chr 15, non-zero entropies in exons (+/-100bp) in all strains. Each point corresponds to the entropy of a variant at that position along the chromosome

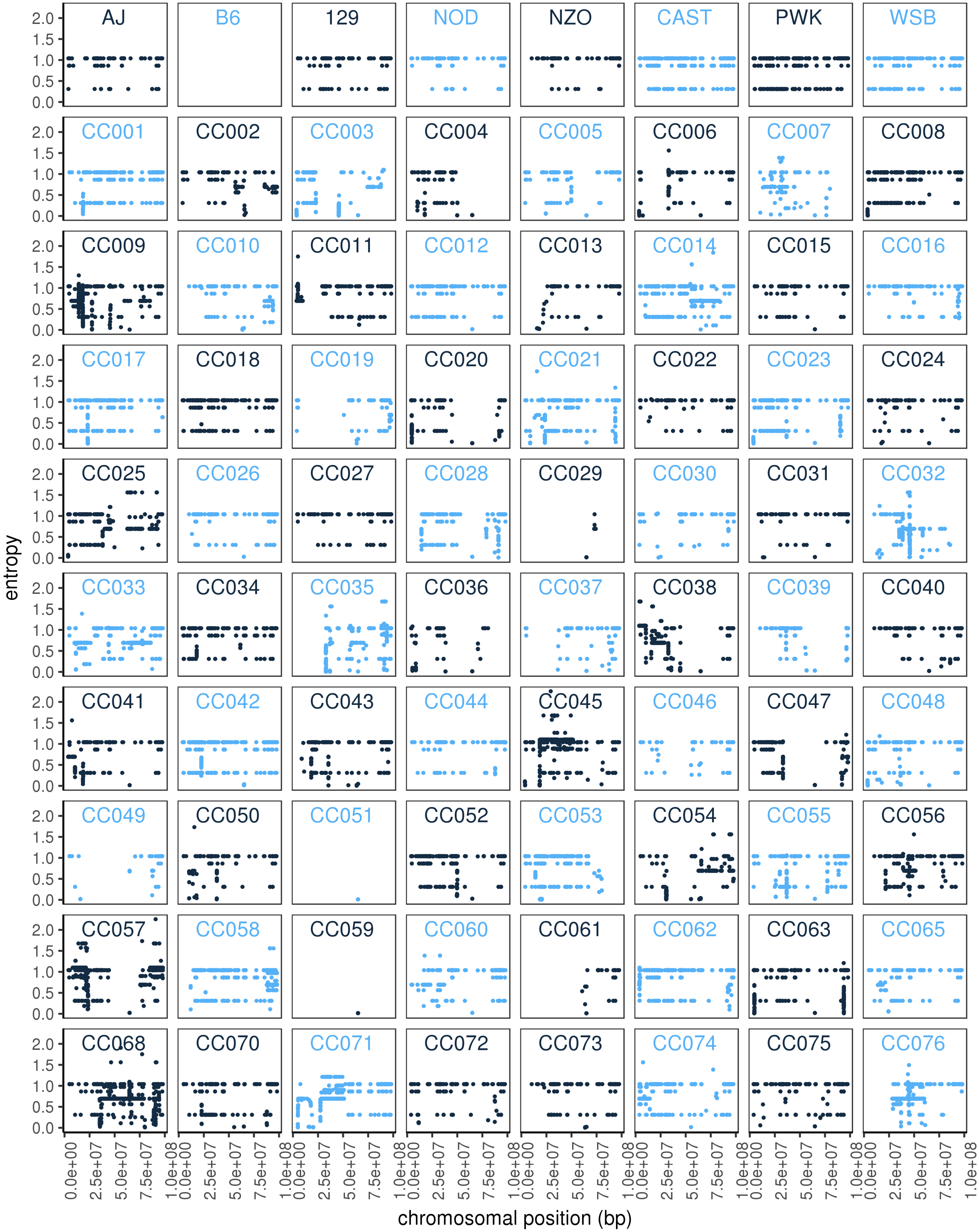

Figure S17 chr 16, non-zero entropies in exons (+/-100bp) in all strains. Each point corresponds to the entropy of a variant at that position along the chromosome

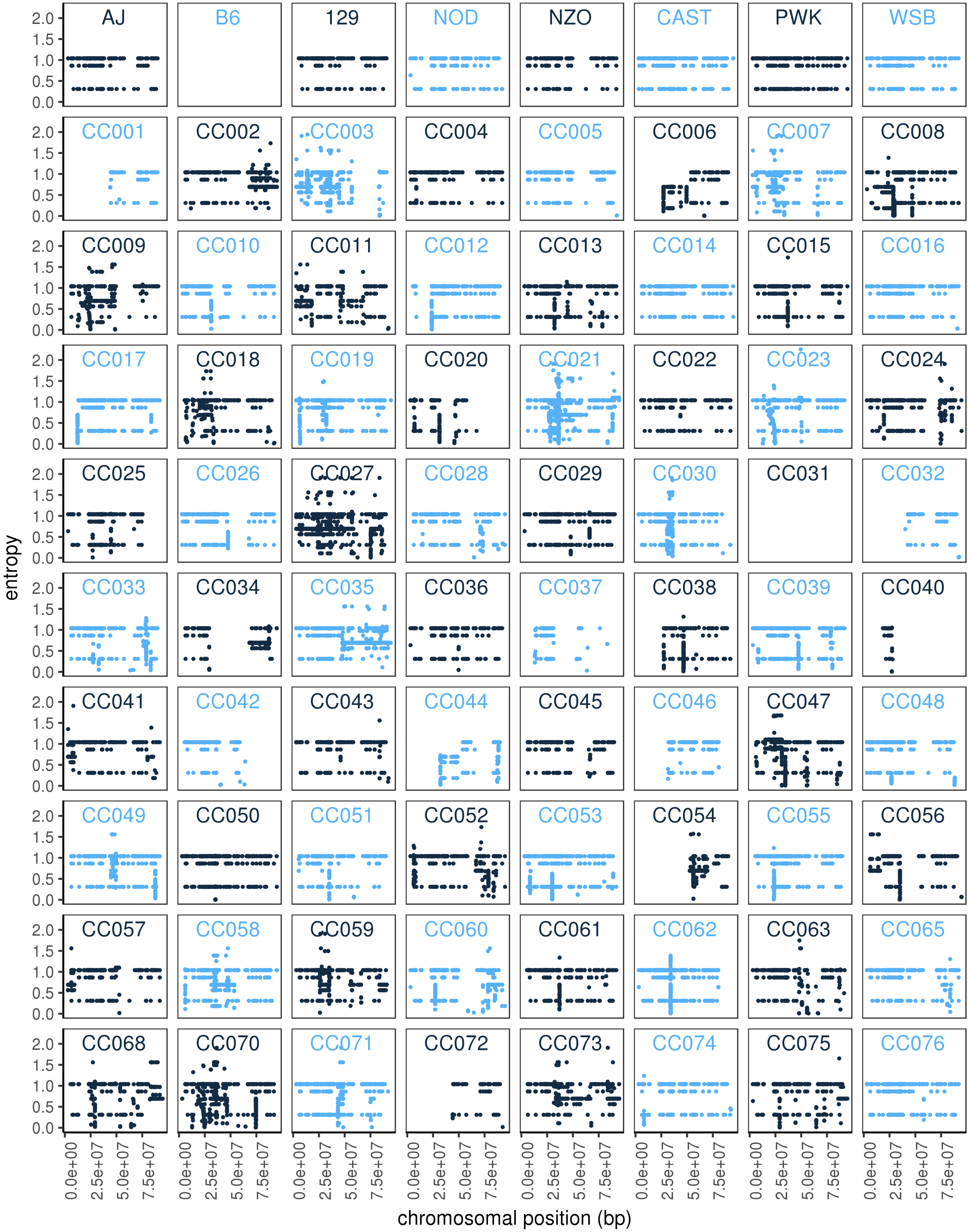

Figure S18 chr 17, non-zero entropies in exons (+/-100bp) in all strains. Each point corresponds to the entropy of a variant at that position along the chromosome

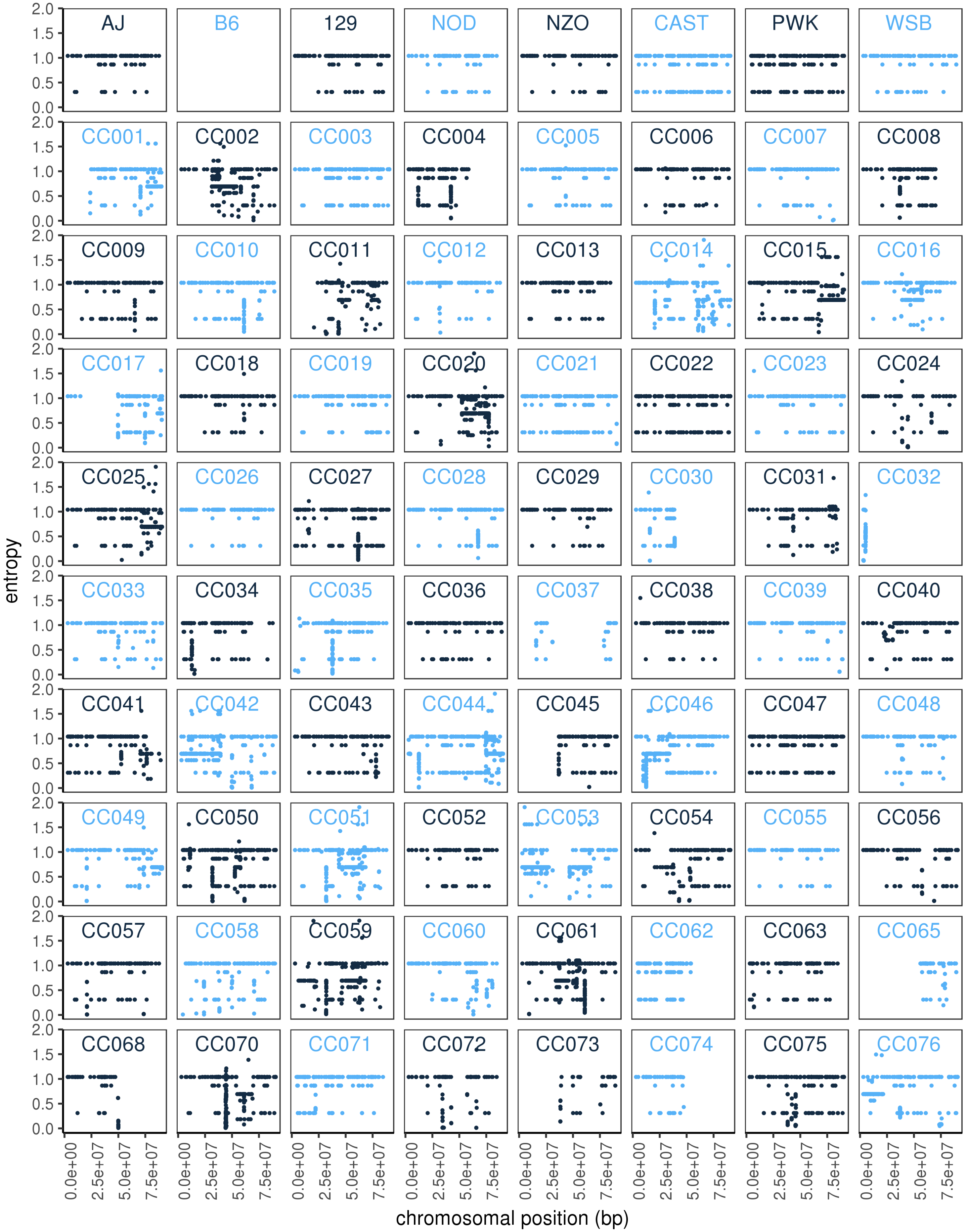

Figure S19 chr 18, non-zero entropies in exons (+/-100bp) in all strains. Each point corresponds to the entropy of a variant at that position along the chromosome

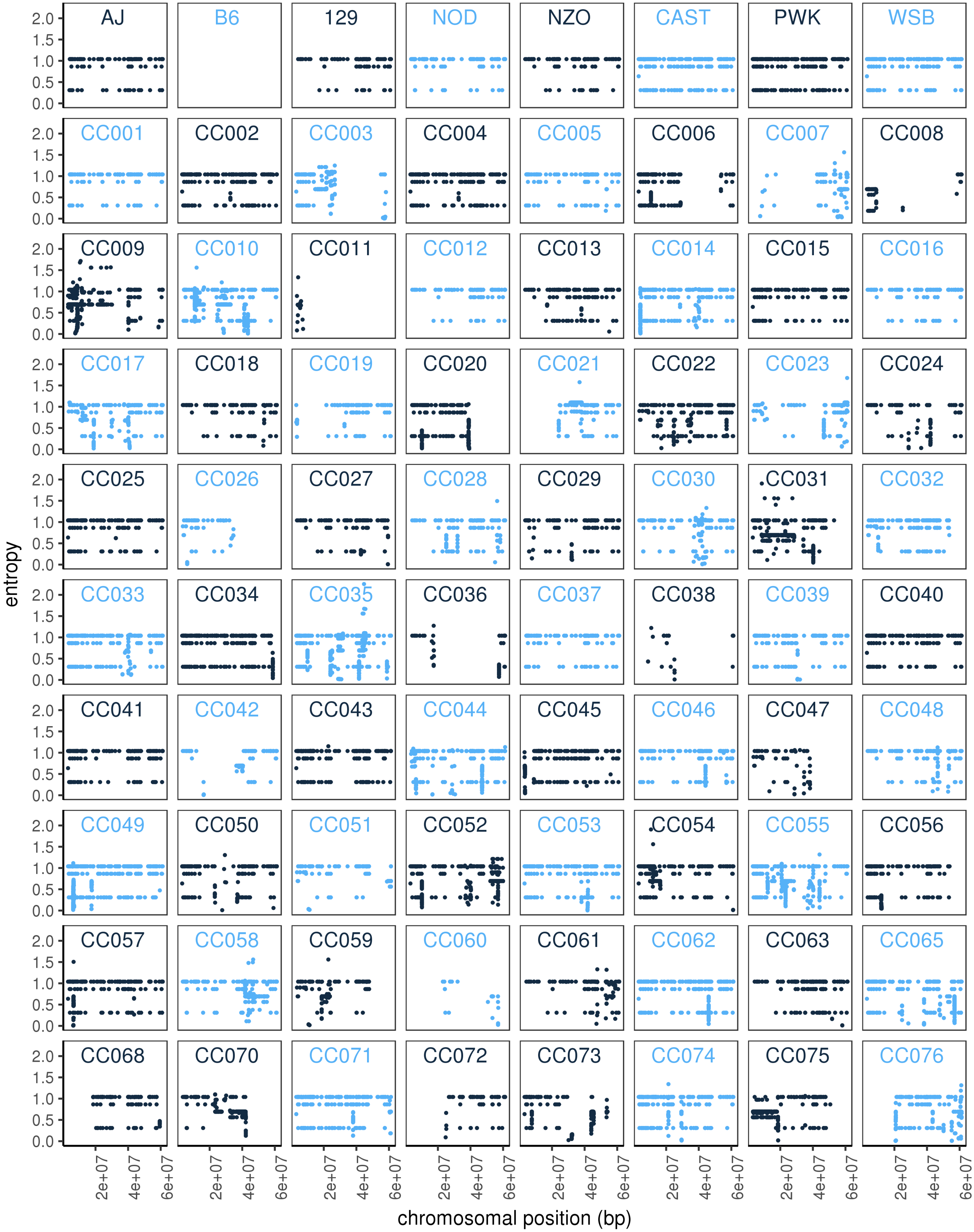

Figure S20 chr 19, non-zero entropies in exons (+/-100bp) in all strains. Each point corresponds to the entropy of a variant at that position along the chromosome

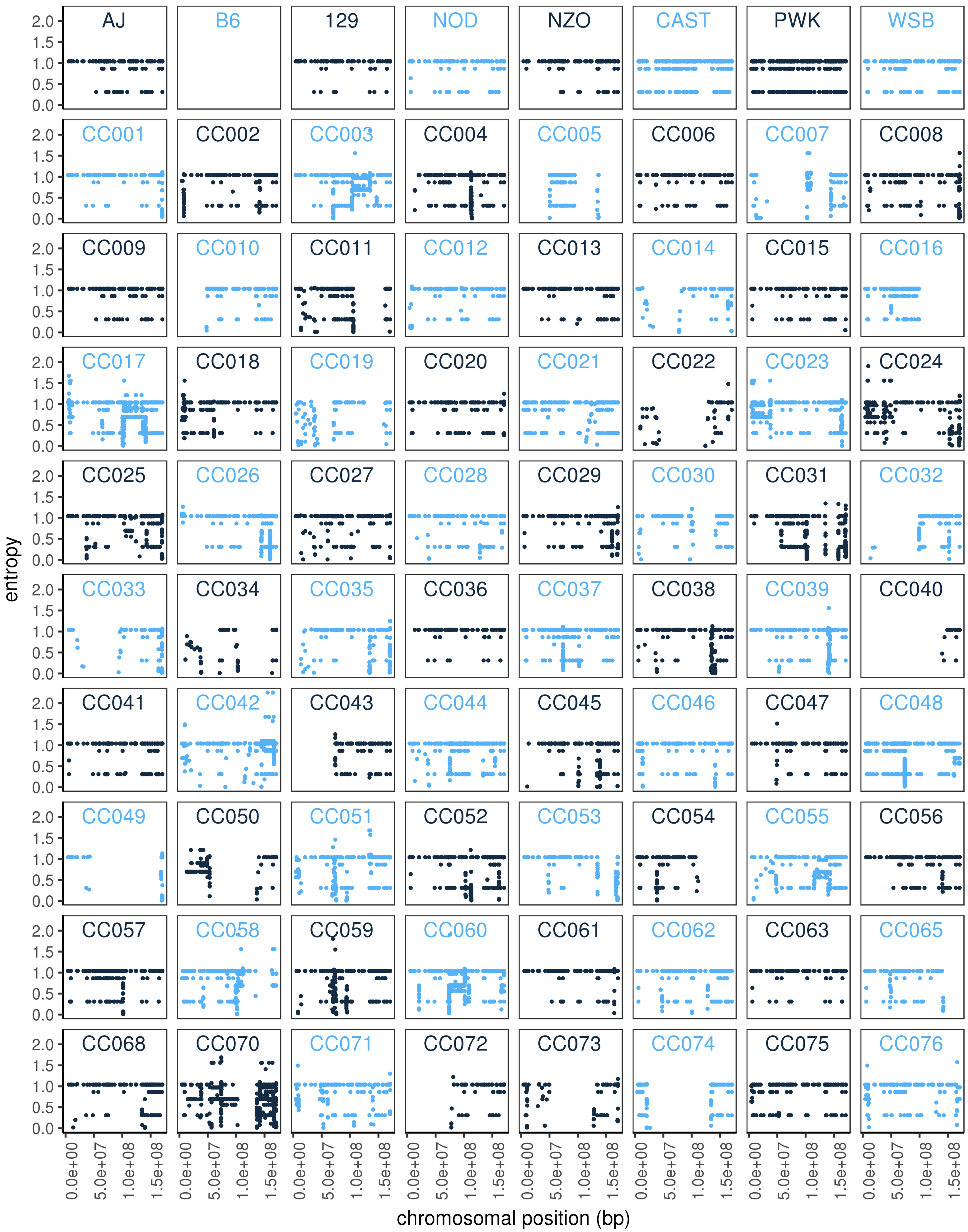

Figure S21 chr X, non-zero entropies in exons (+/-100bp) in all strains. Each point corresponds to the entropy of a variant at that position along the chromosome

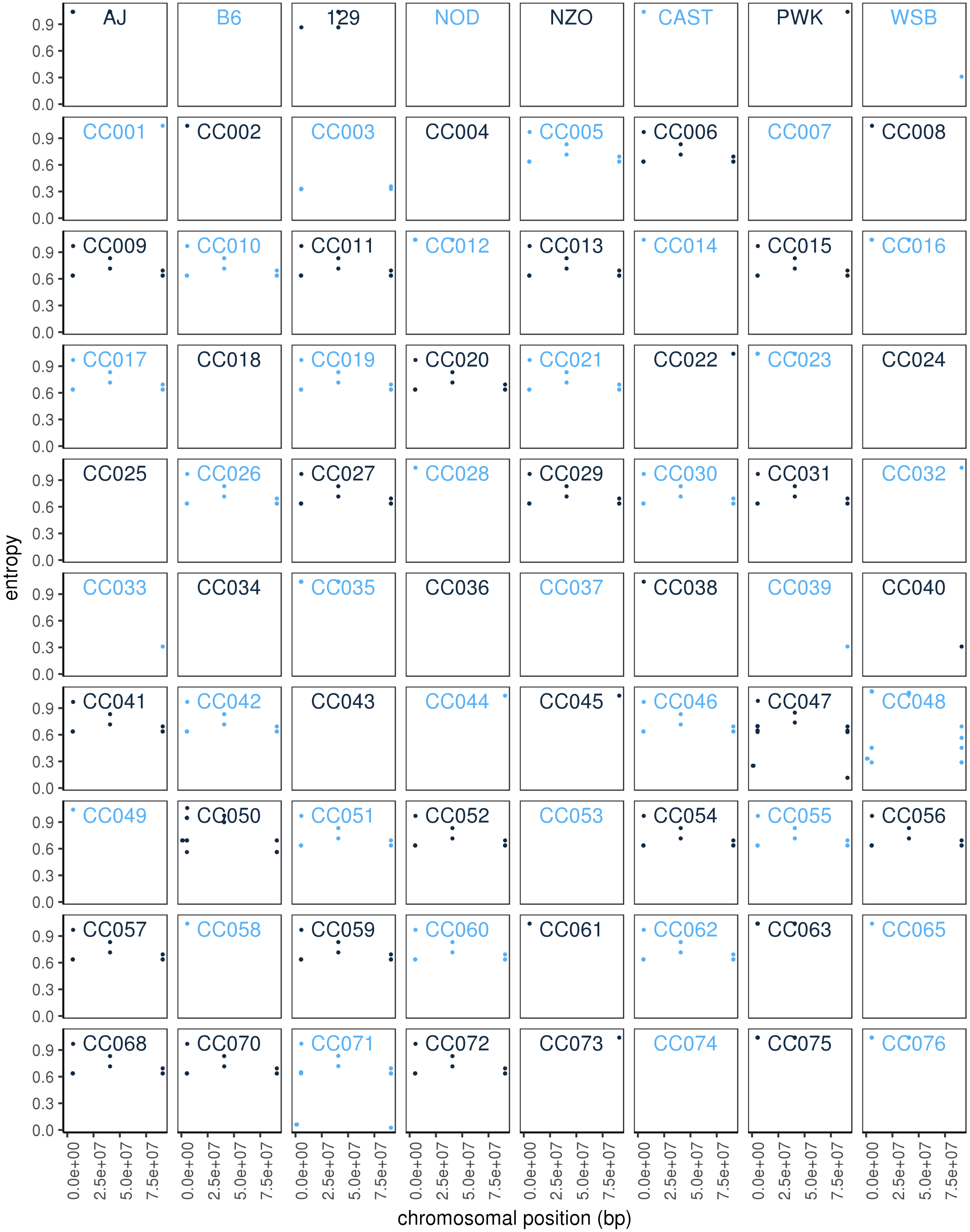

Figure S22 chr Y, non-zero entropies in exons (+/-100bp) in all strains. Each point corresponds to the entropy of a variant at that position along the chromosome

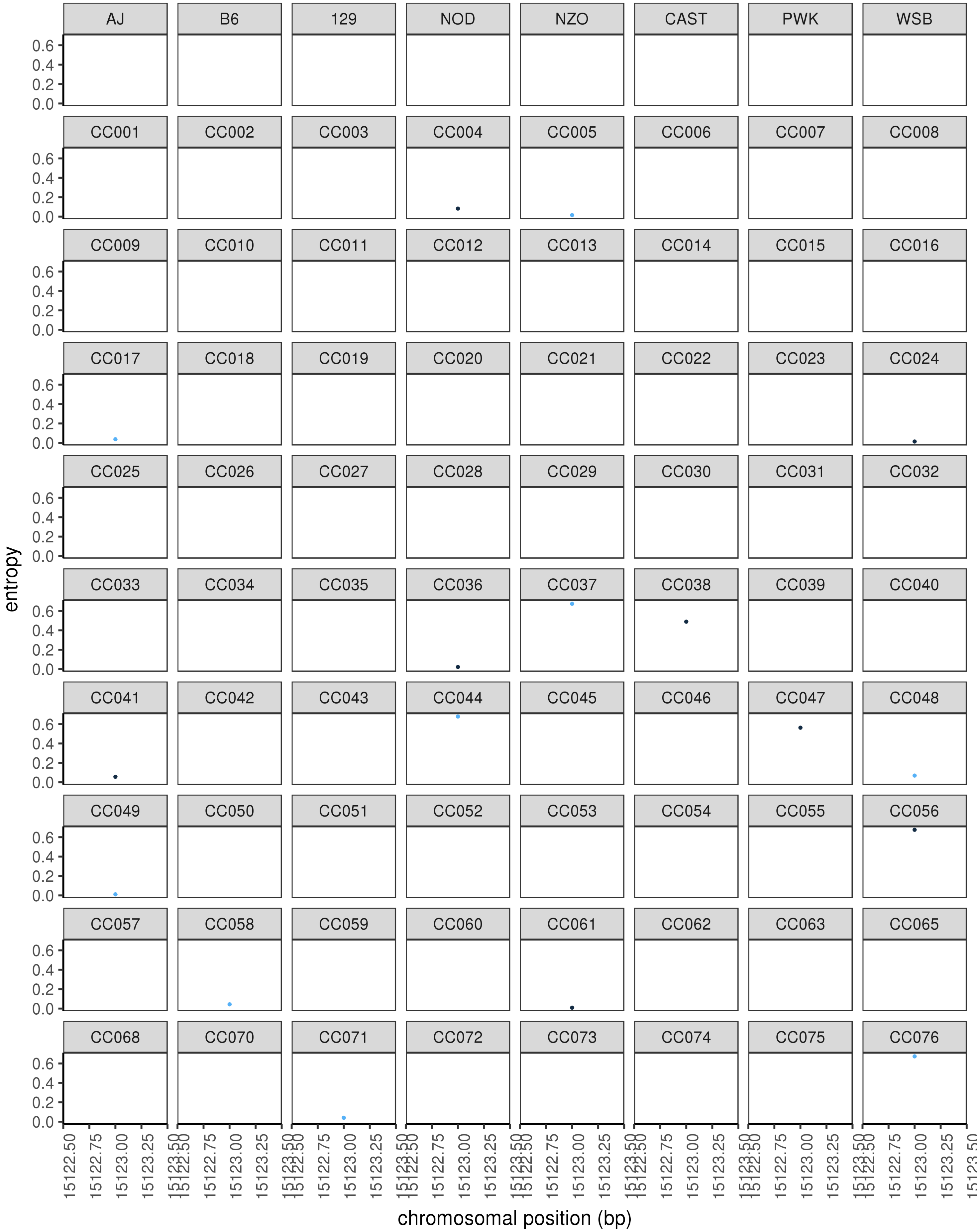

Figure S23 chr MT, non-zero entropies in exons (+/-100bp) in all strains. Each point corresponds to the entropy of a variant at that position along the chromosome

2.2 Entropies per strain

Each figure corresponds to a strain. Each panel depicts the non-zero entropies in one of that strain's chromosomes.

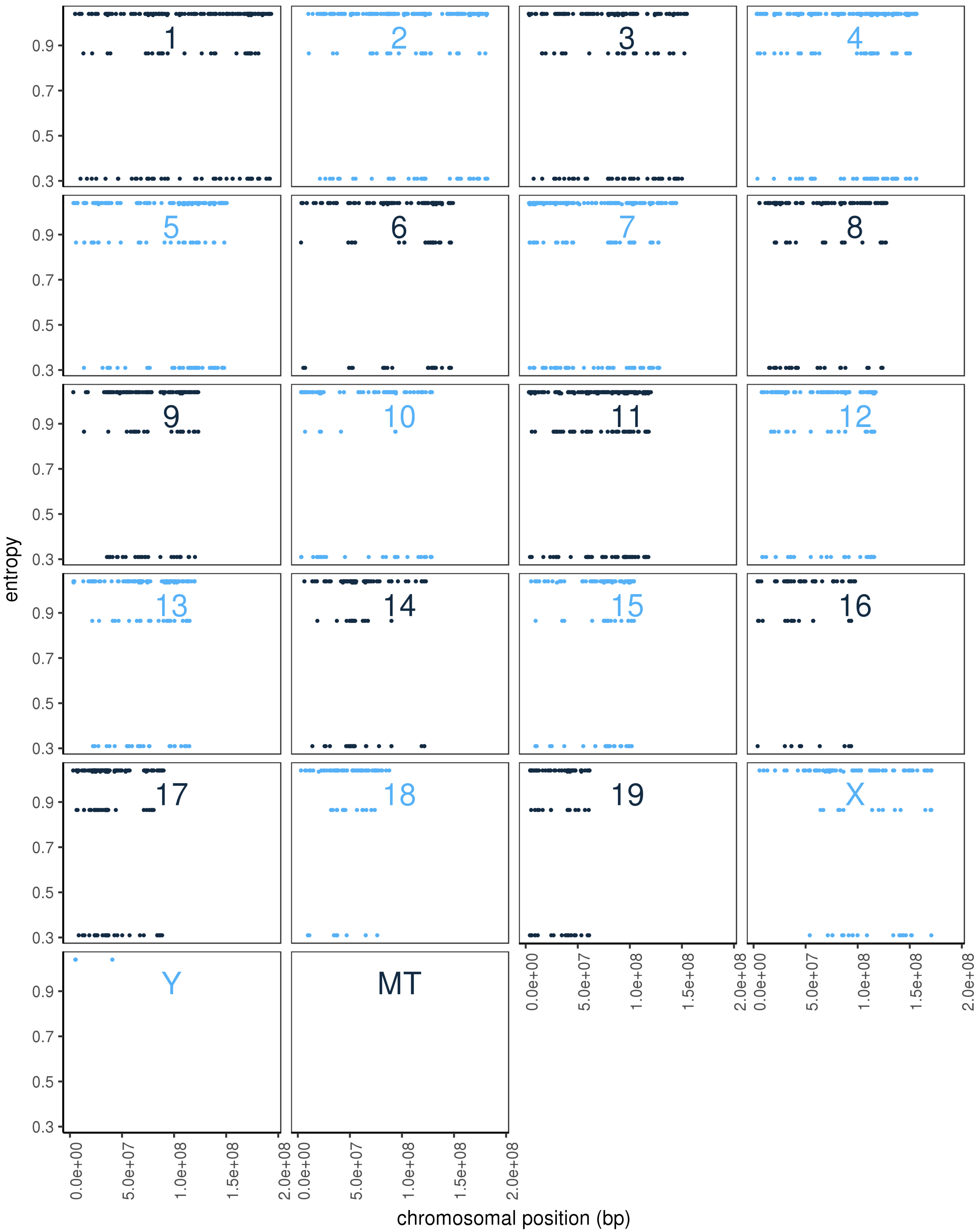

Figure S24 strain AJ, non-zero entropies in exons (+/-100 bp) in all chromosomes. Each point corresponds to the entropy of a variant at that position along the chromosome

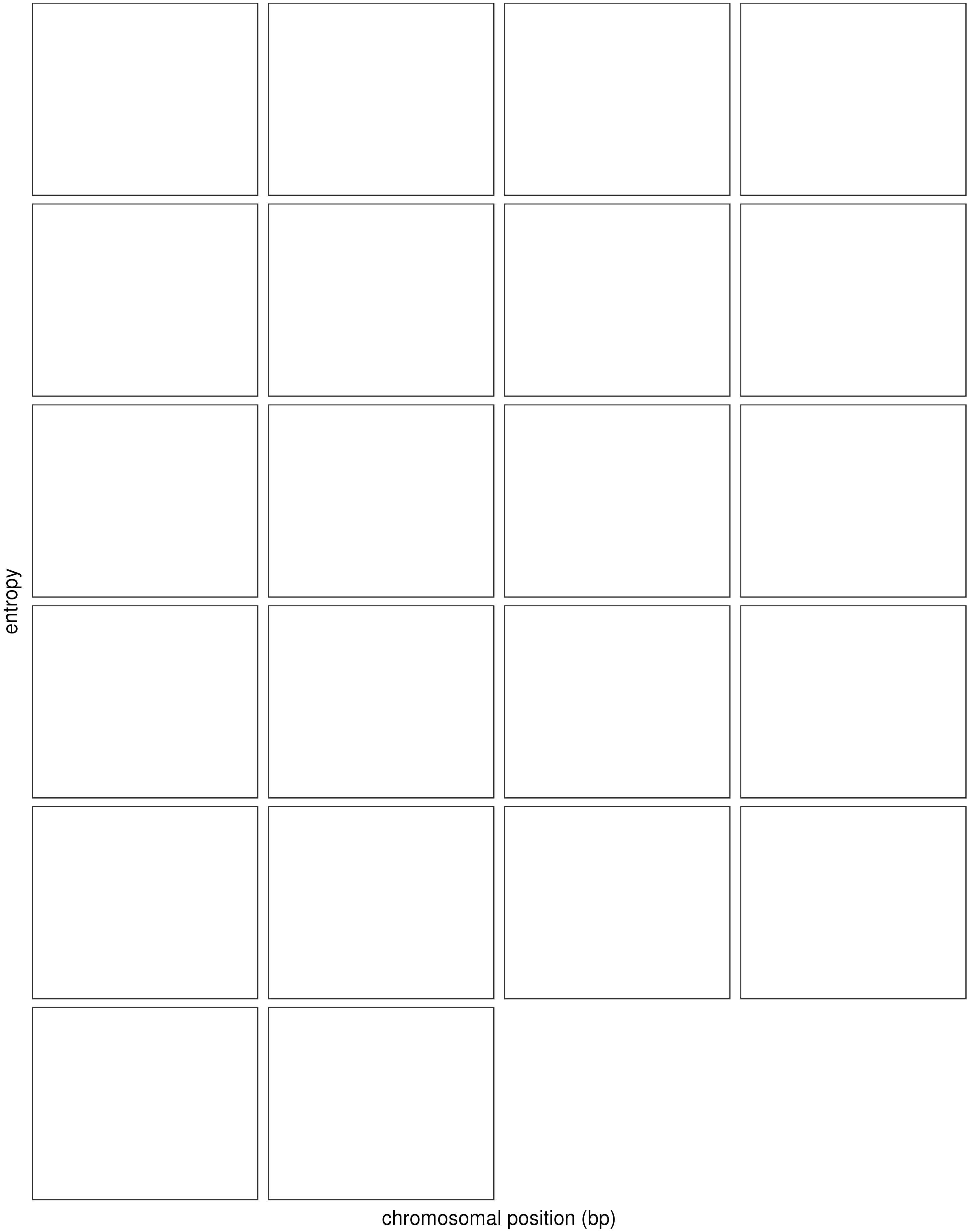

Figure S25 strain B6, non-zero entropies in exons (+/-100 bp) in all chromosomes. Each point corresponds to the entropy of a variant at that position along the chromosome

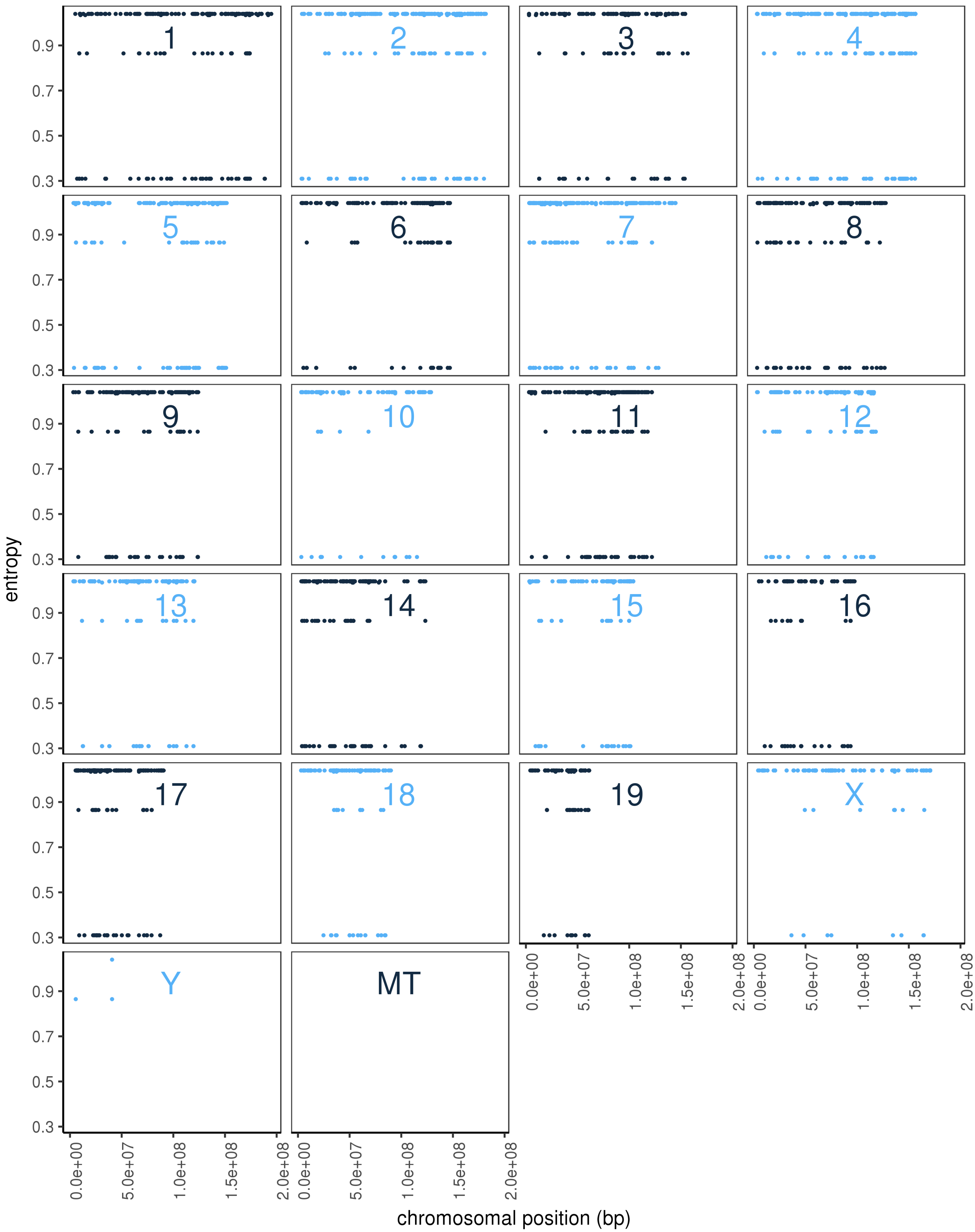

Figure S26 strain 129, non-zero entropies in exons (+/-100 bp) in all chromosomes. Each point corresponds to the entropy of a variant at that position along the chromosome

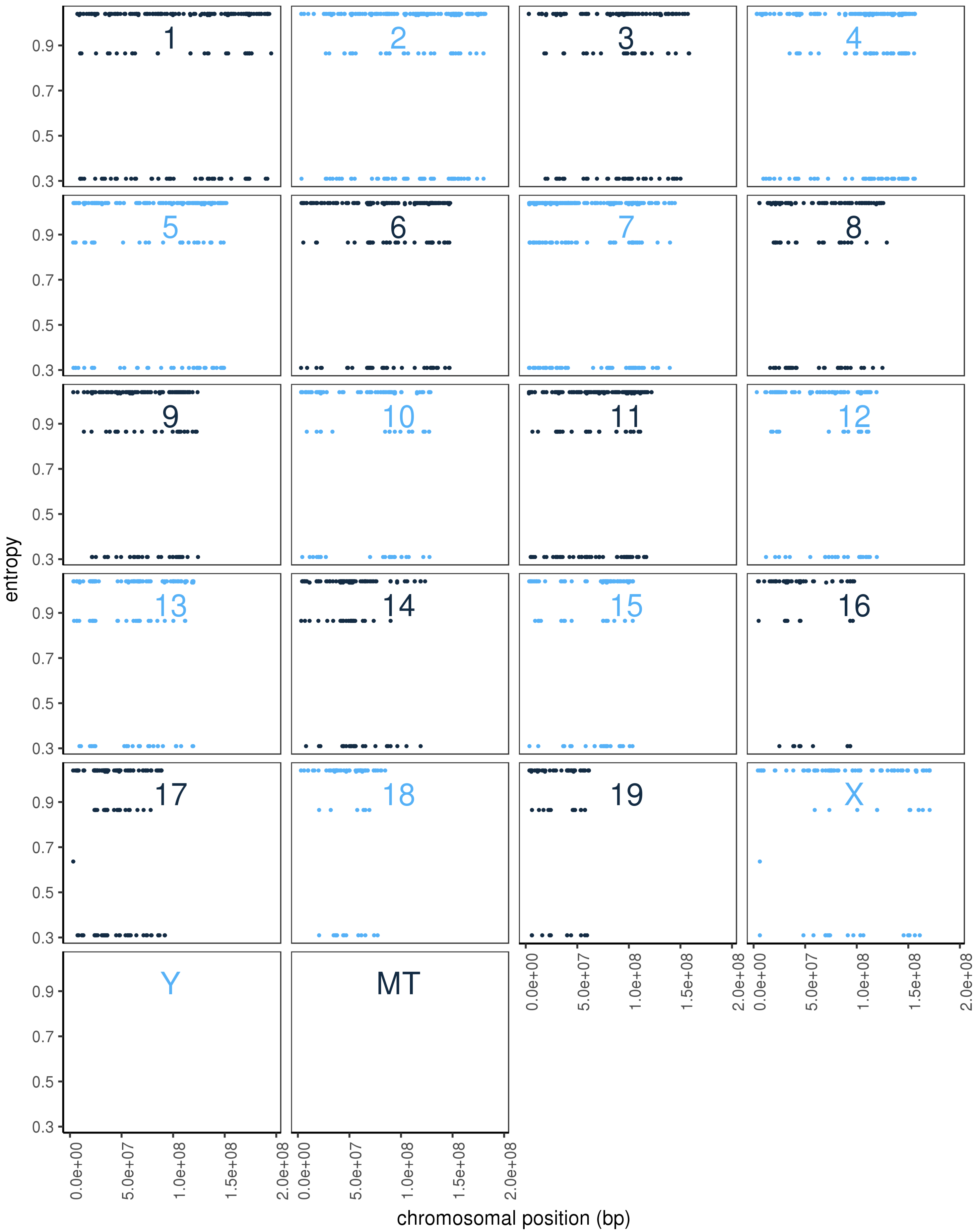

Figure S27 strain NOD, non-zero entropies in exons (+/-100 bp) in all chromosomes. Each point corresponds to the entropy of a variant at that position along the chromosome

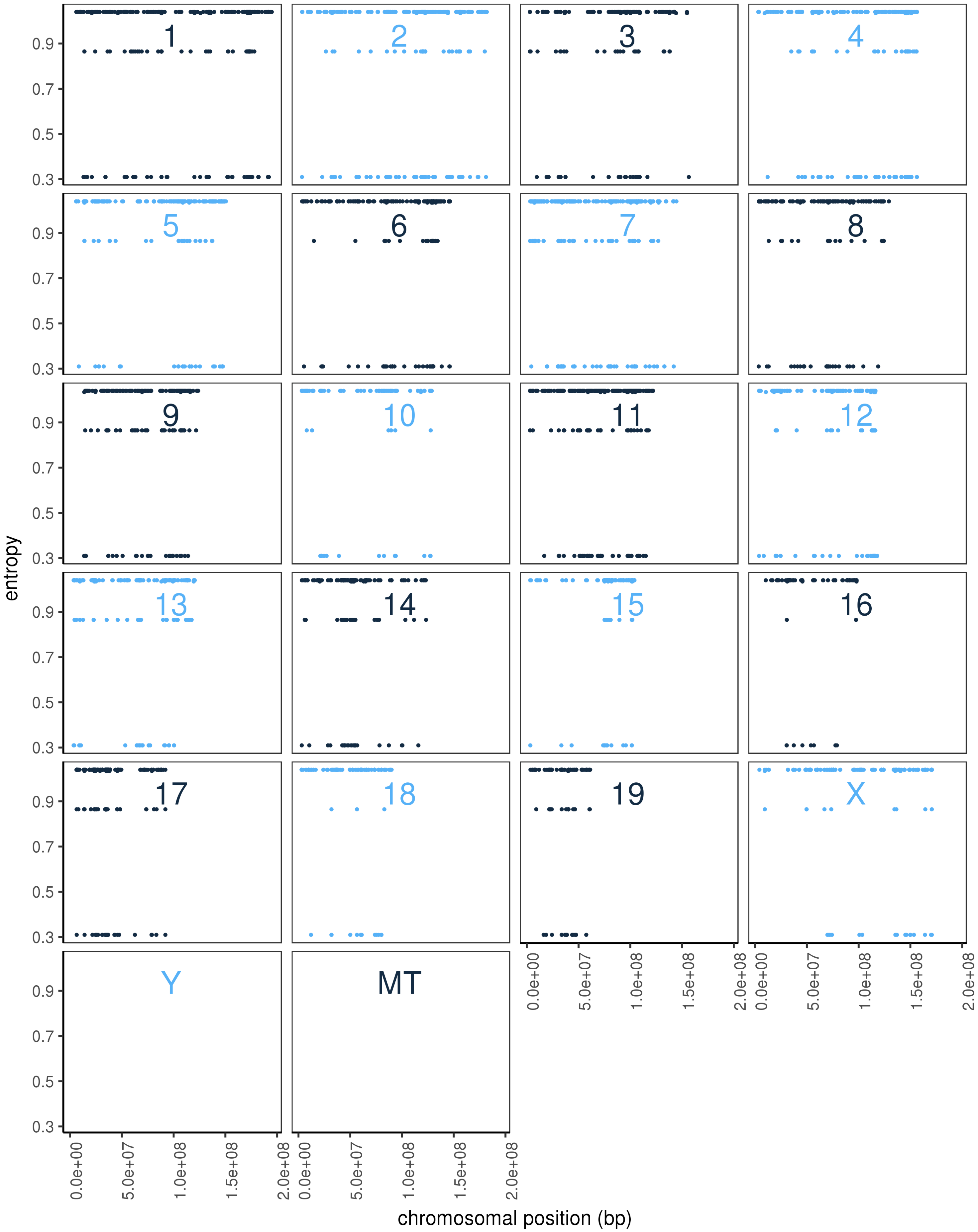

Figure S28 strain NZO, non-zero entropies in exons (+/-100 bp) in all chromosomes. Each point corresponds to the entropy of a variant at that position along the chromosome

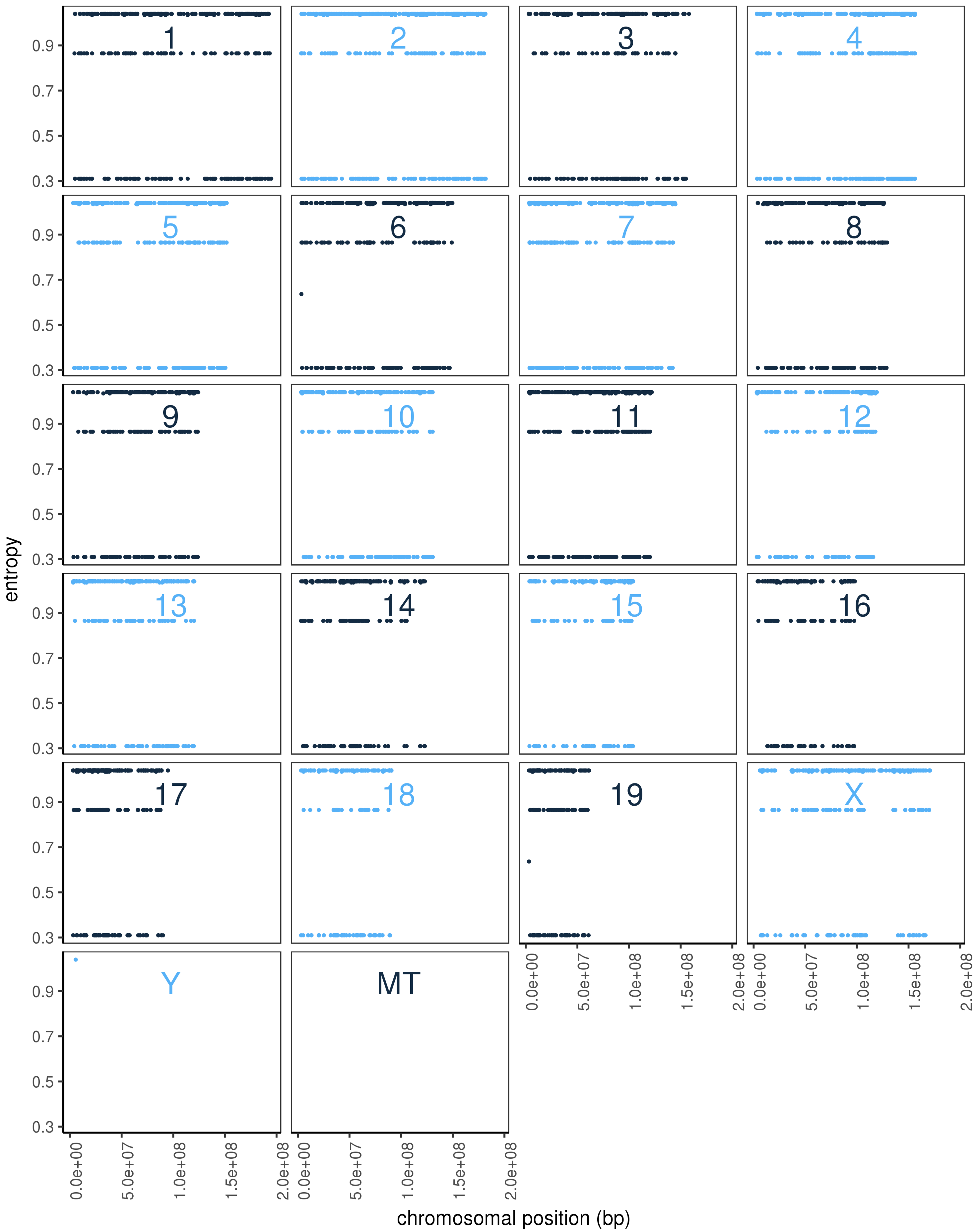

Figure S29 strain CAST, non-zero entropies in exons (+/-100 bp) in all chromosomes. Each point corresponds to the entropy of a variant at that position along the chromosome

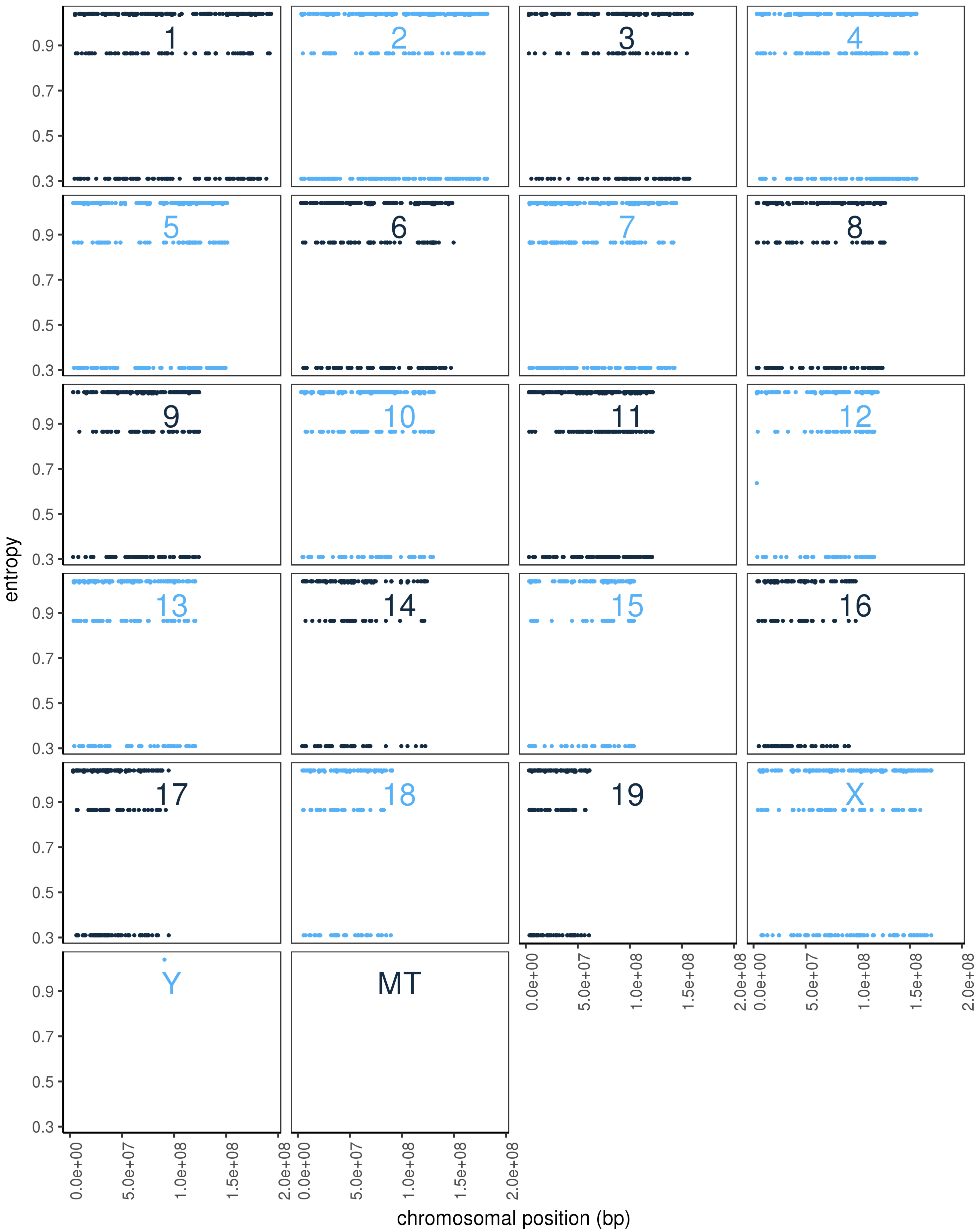

Figure S30 strain PWK, non-zero entropies in exons (+/-100 bp) in all chromosomes. Each point corresponds to the entropy of a variant at that position along the chromosome

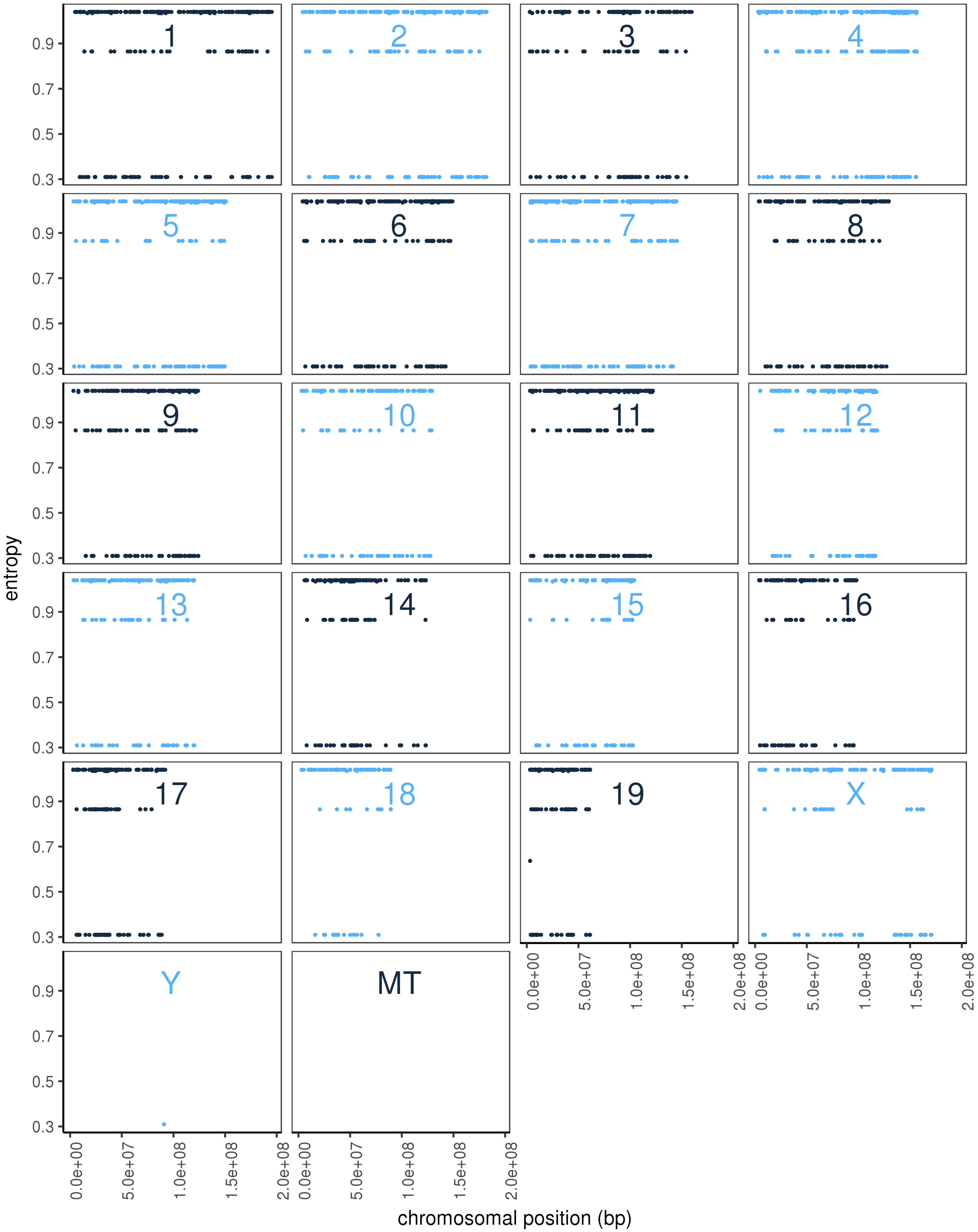

Figure S31 strain WSB, non-zero entropies in exons (+/-100 bp) in all chromosomes. Each point corresponds to the entropy of a variant at that position along the chromosome

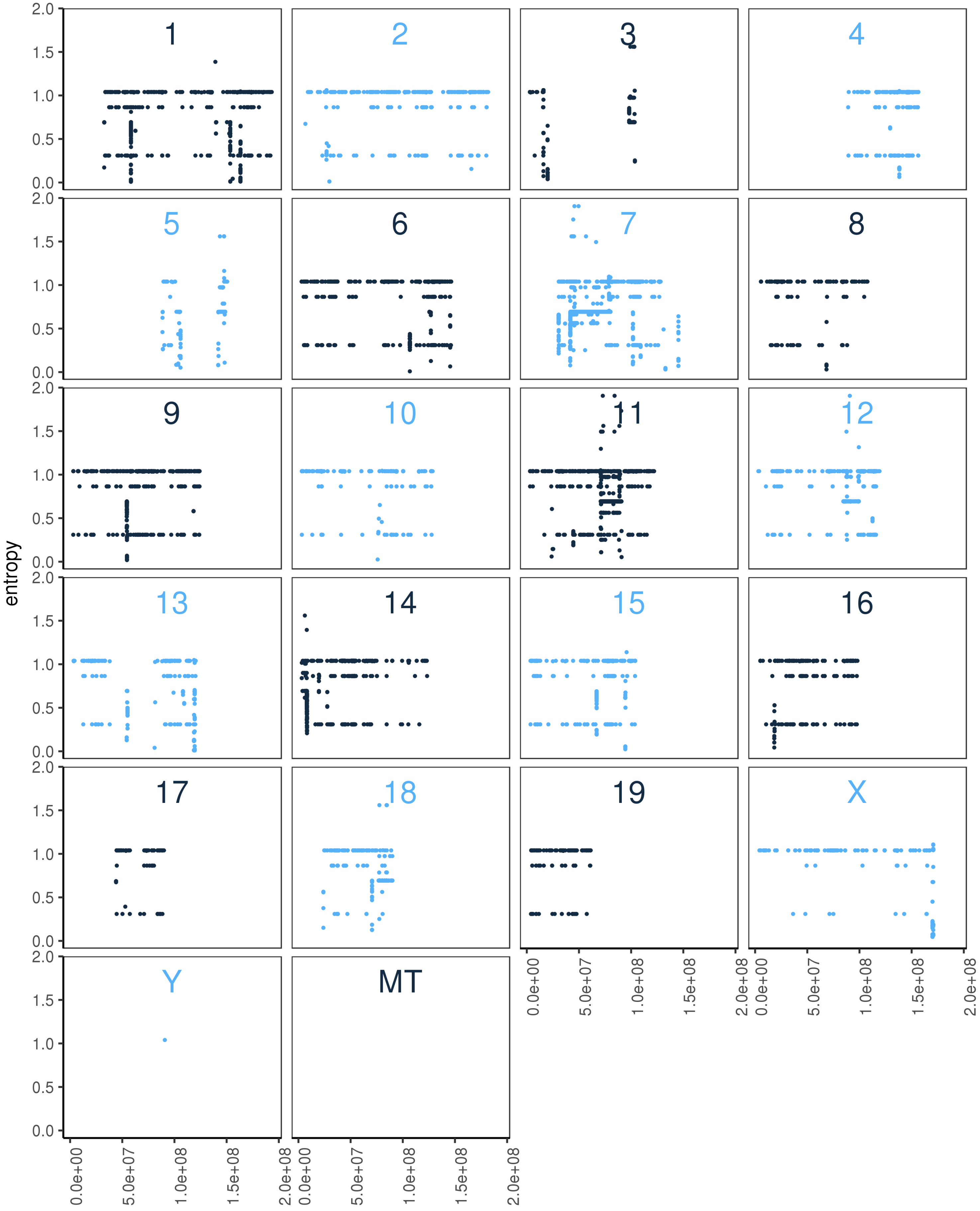

Figure S32 strain CC001, non-zero entropies in exons (+/-100 bp) in all chromosomes. Each point corresponds to the entropy of a variant at that position along the chromosome

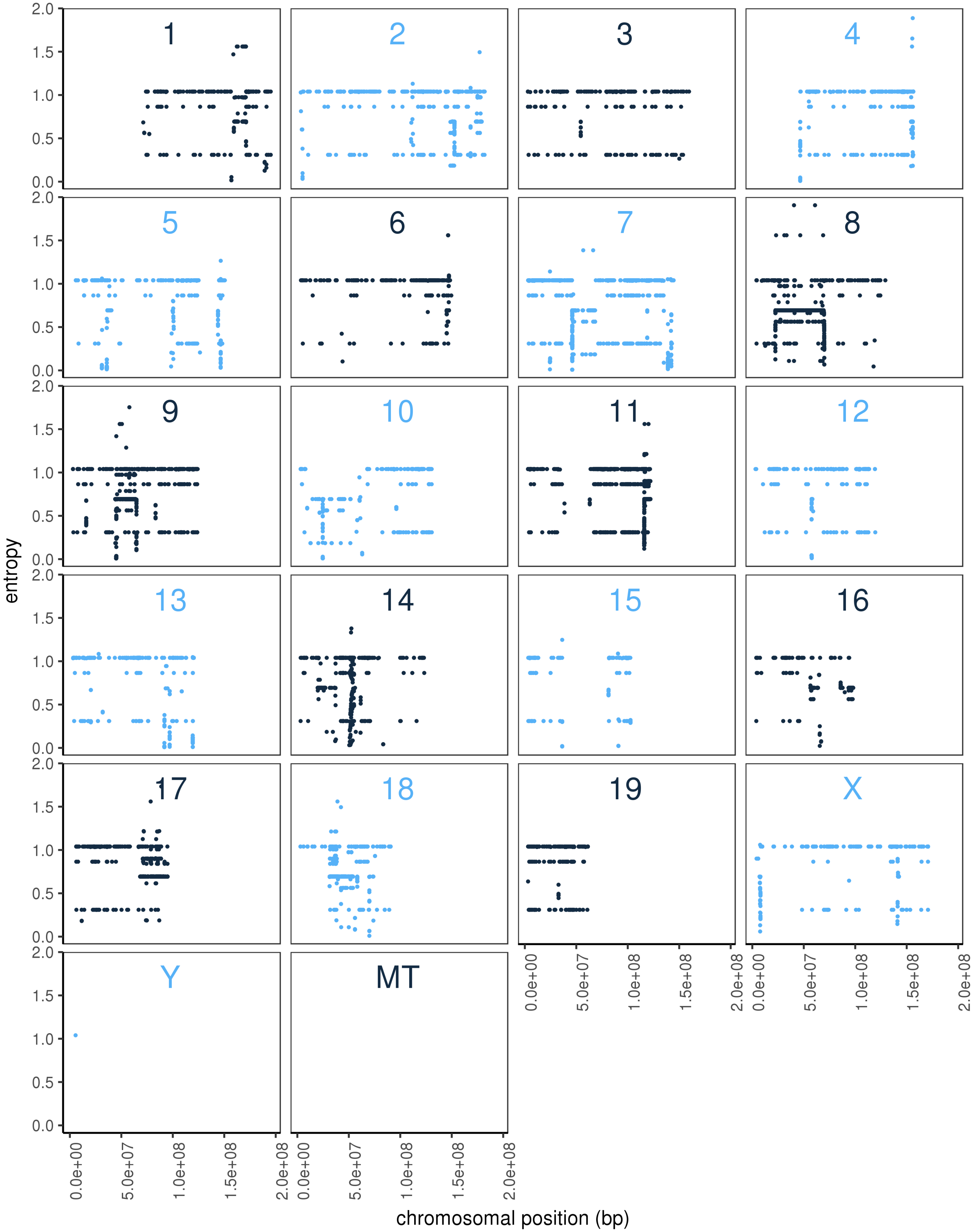

Figure S33 strain CC002, non-zero entropies in exons (+/-100 bp) in all chromosomes. Each point corresponds to the entropy of a variant at that position along the chromosome

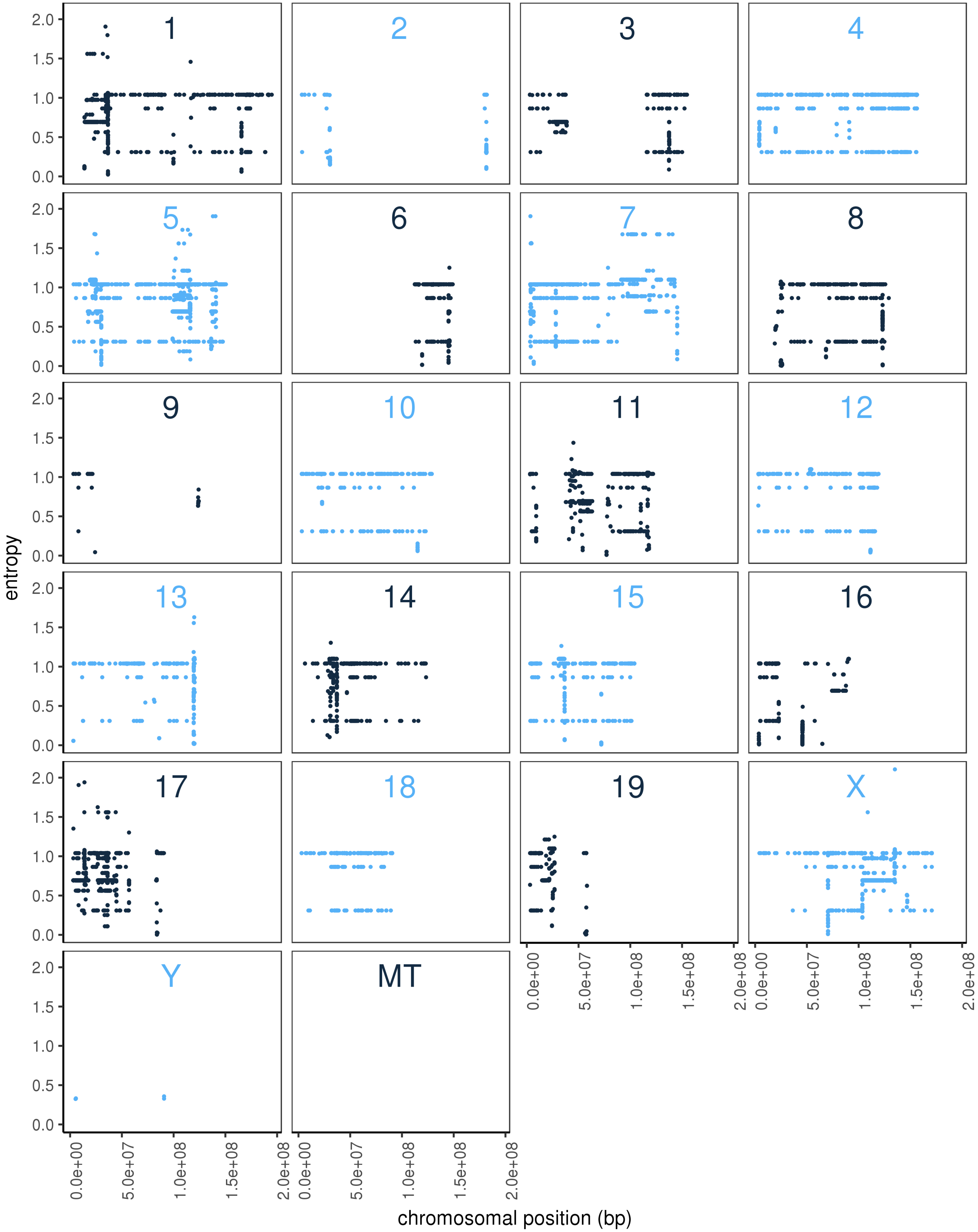

Figure S34 strain CC003, non-zero entropies in exons (+/-100 bp) in all chromosomes. Each point corresponds to the entropy of a variant at that position along the chromosome

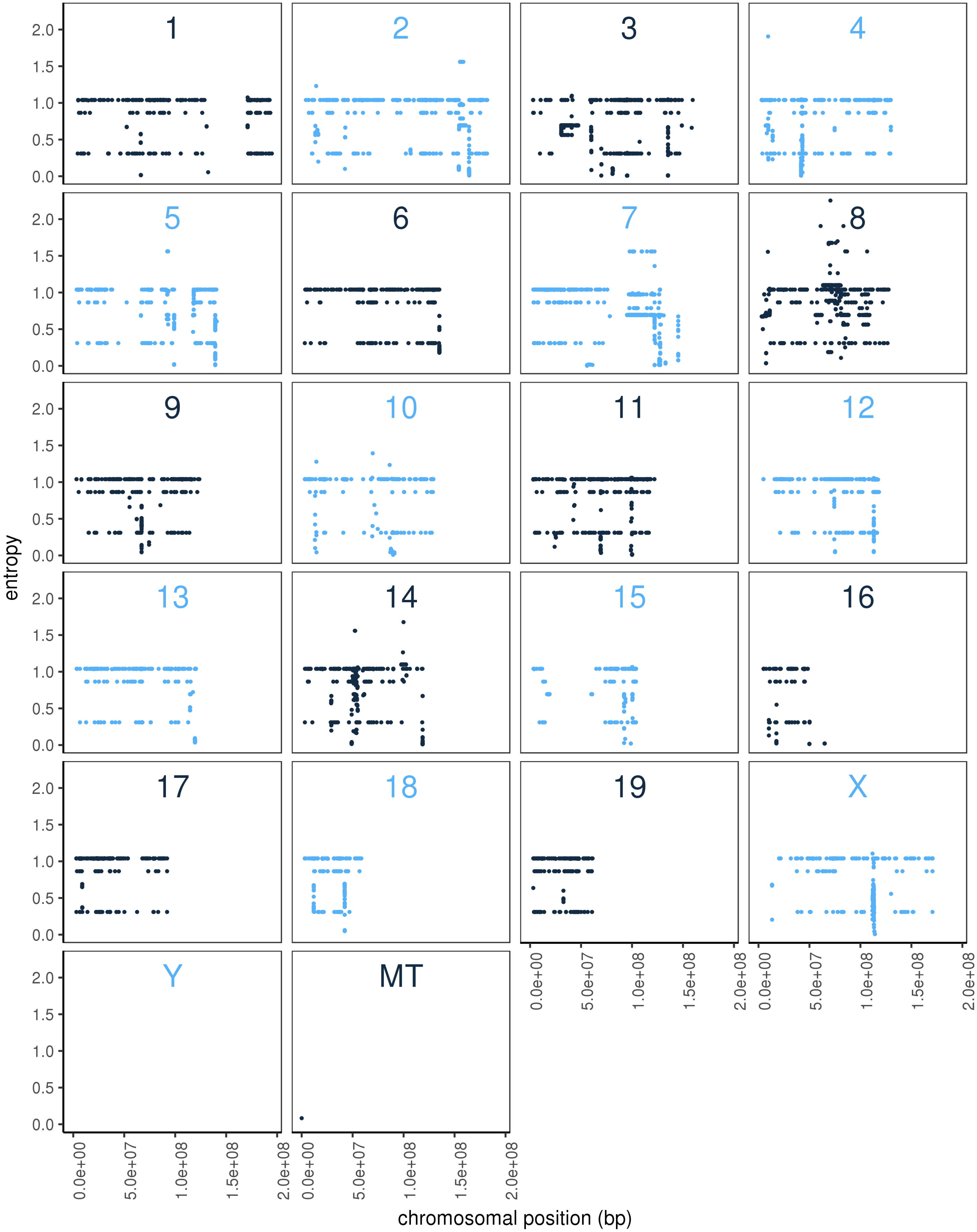

Figure S35 strain CC004, non-zero entropies in exons (+/-100 bp) in all chromosomes. Each point corresponds to the entropy of a variant at that position along the chromosome

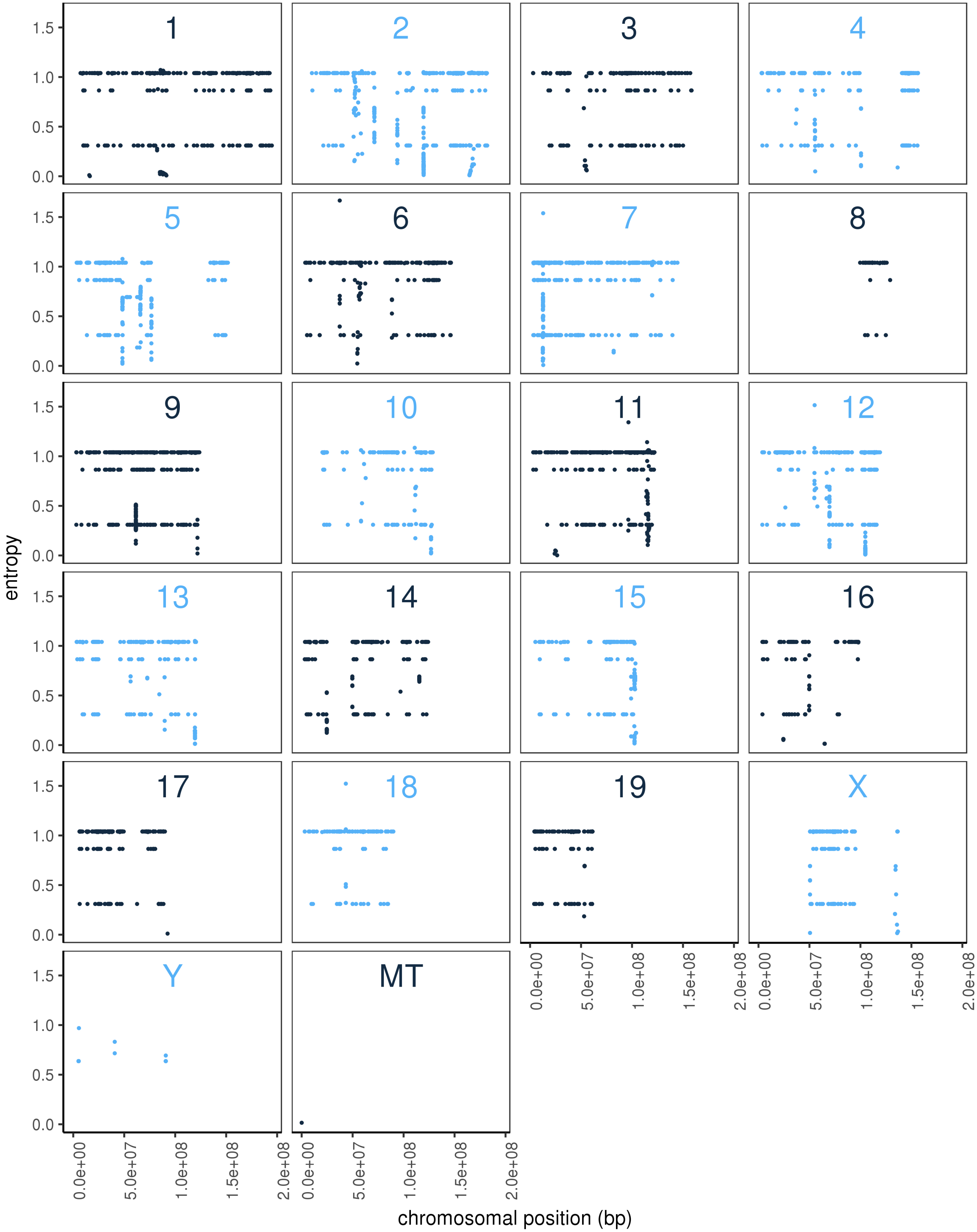

Figure S36 strain CC005, non-zero entropies in exons (+/-100 bp) in all chromosomes. Each point corresponds to the entropy of a variant at that position along the chromosome

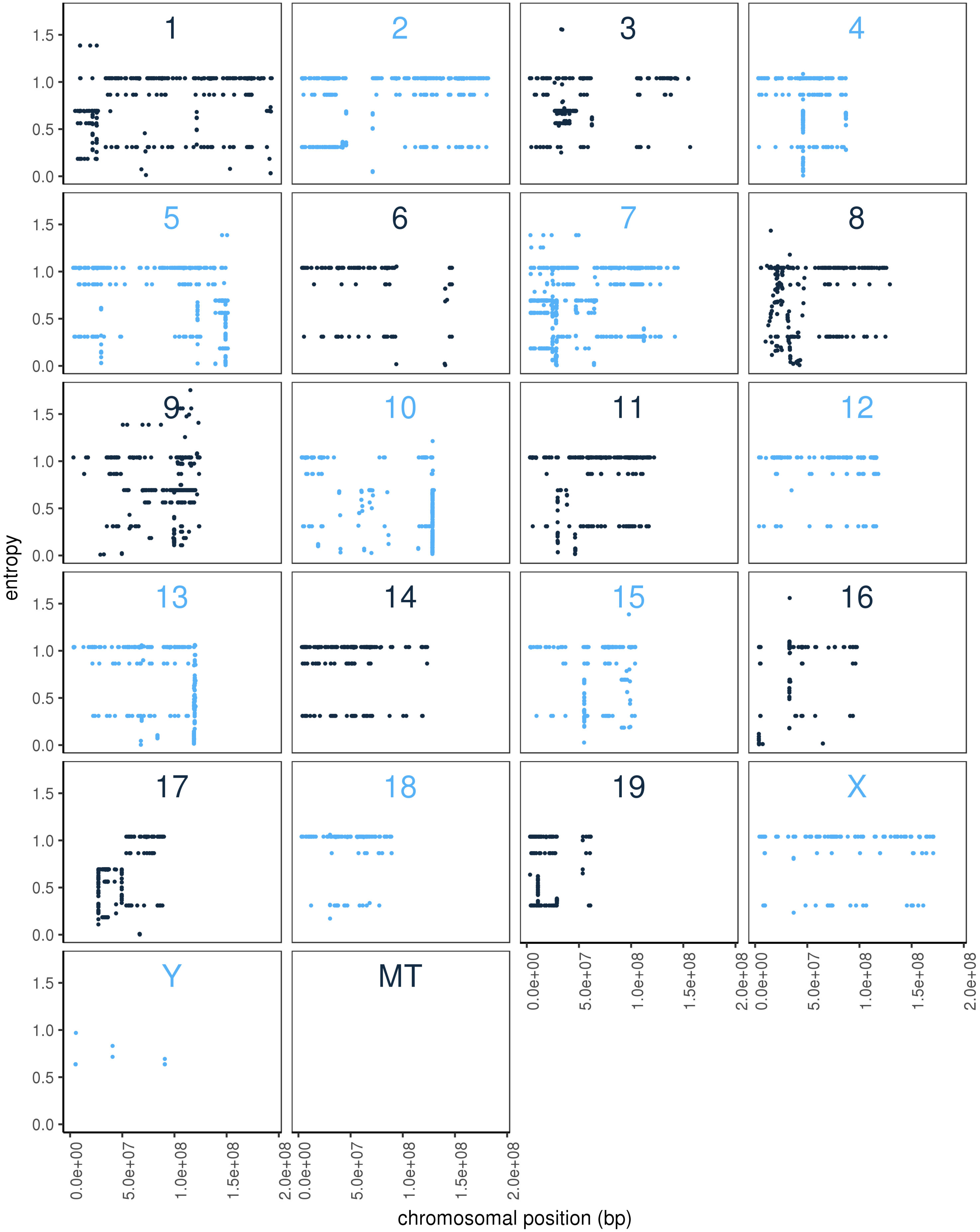

Figure S37 strain CC006, non-zero entropies in exons (+/-100 bp) in all chromosomes. Each point corresponds to the entropy of a variant at that position along the chromosome

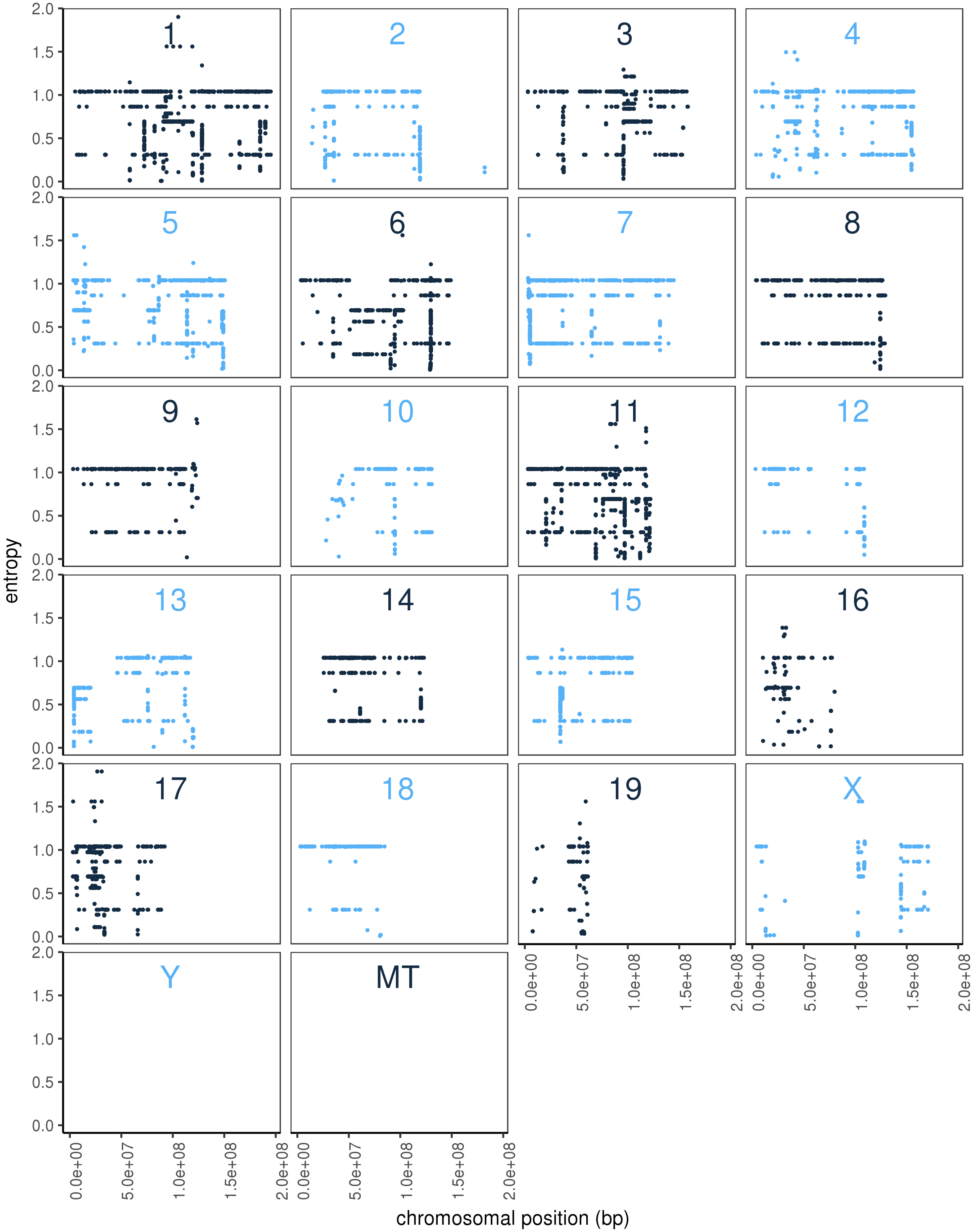

Figure S38 strain CC007, non-zero entropies in exons (+/-100 bp) in all chromosomes. Each point corresponds to the entropy of a variant at that position along the chromosome

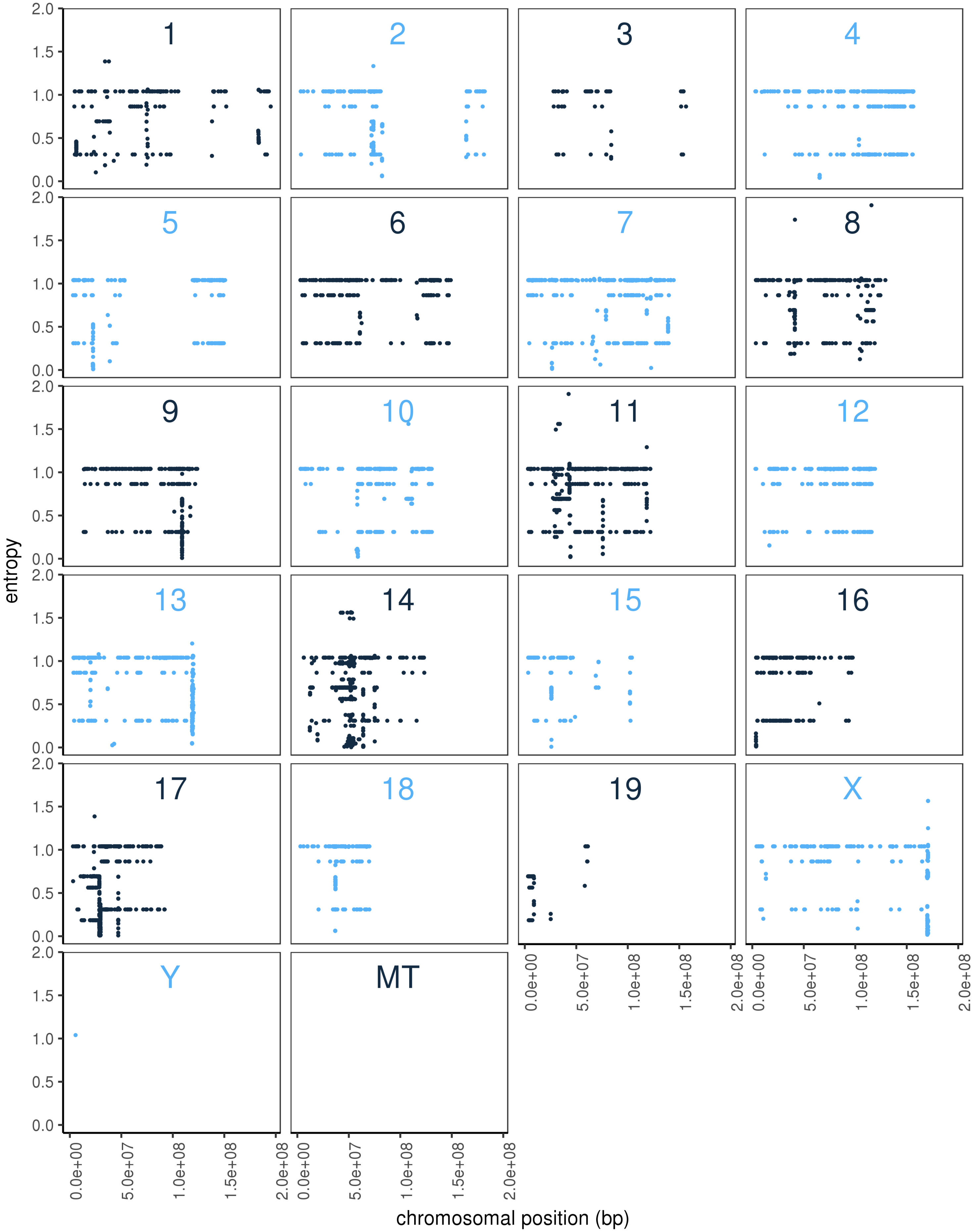

Figure S39 strain CC008, non-zero entropies in exons (+/-100 bp) in all chromosomes. Each point corresponds to the entropy of a variant at that position along the chromosome

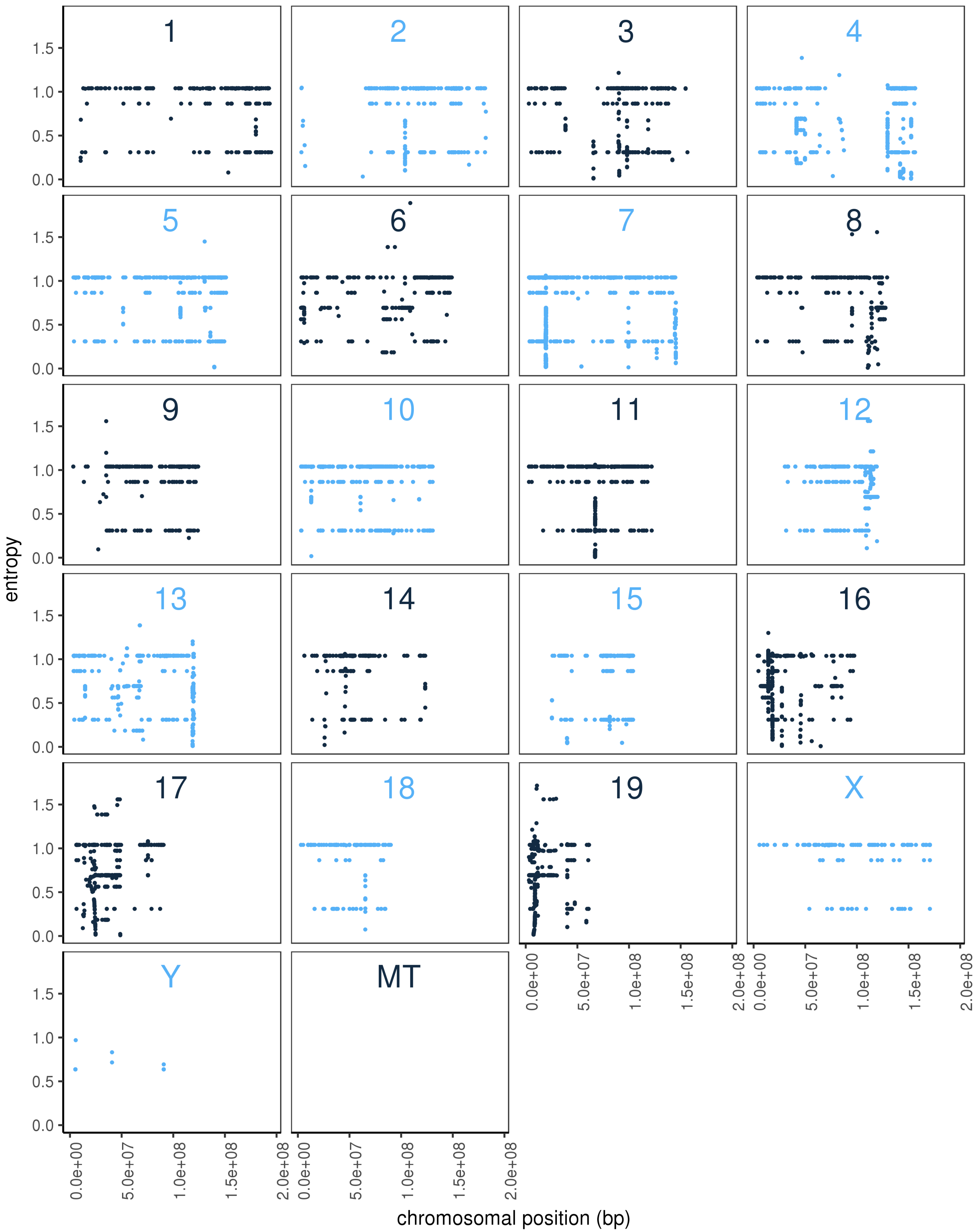

Figure S40 strain CC009, non-zero entropies in exons (+/-100 bp) in all chromosomes. Each point corresponds to the entropy of a variant at that position along the chromosome

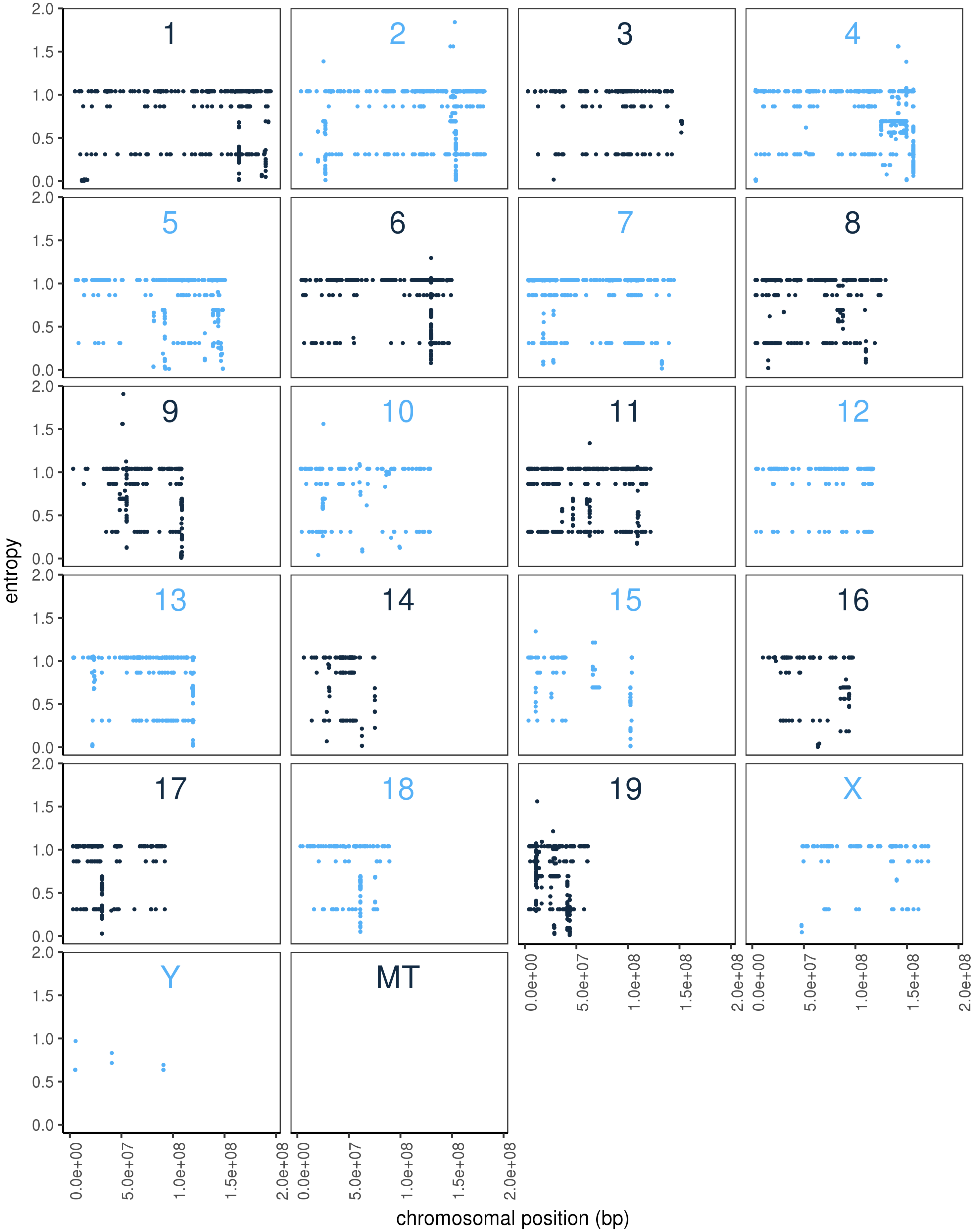

Figure S41 strain CC010, non-zero entropies in exons (+/-100 bp) in all chromosomes. Each point corresponds to the entropy of a variant at that position along the chromosome

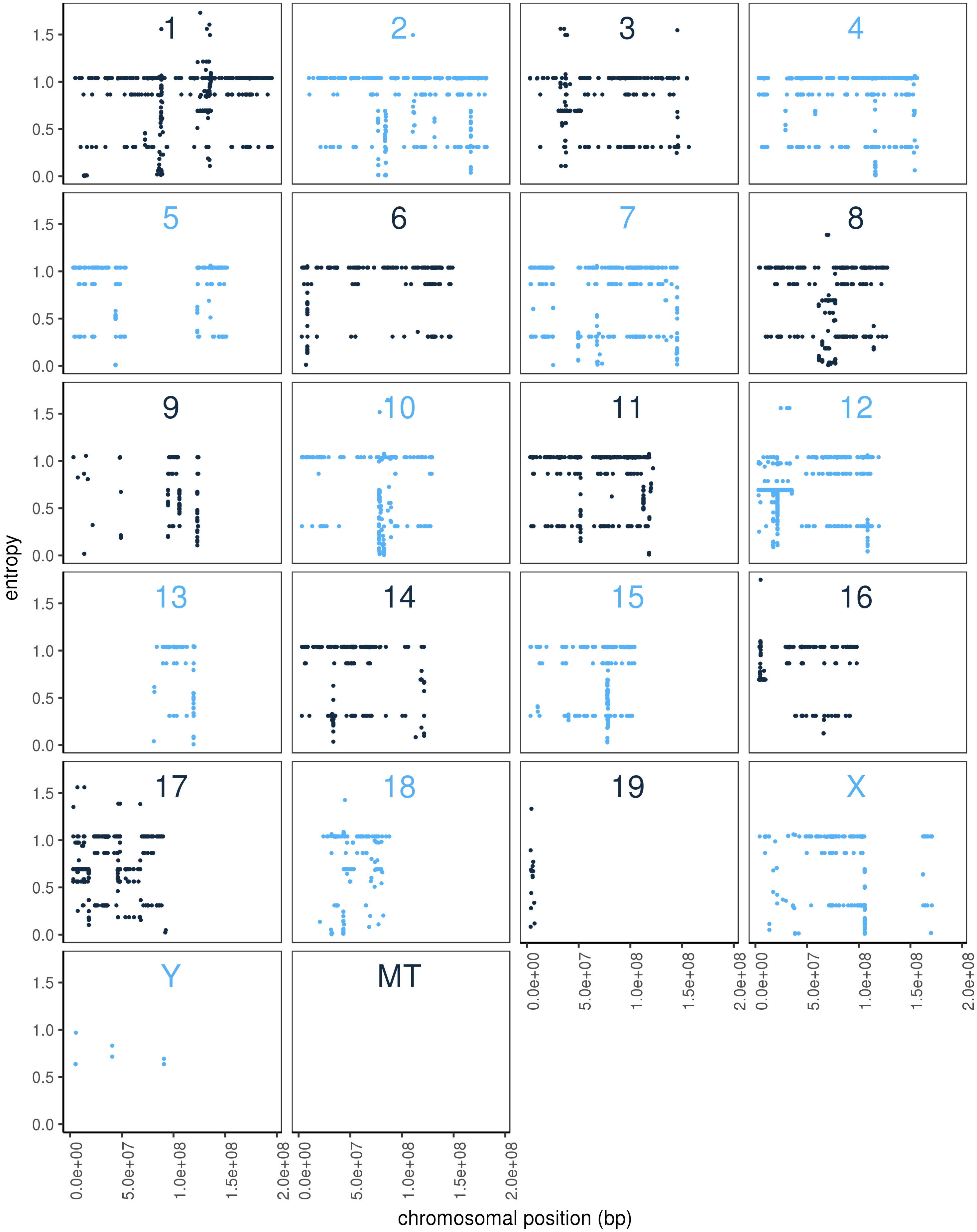

Figure S42 strain CC011, non-zero entropies in exons (+/-100 bp) in all chromosomes. Each point corresponds to the entropy of a variant at that position along the chromosome

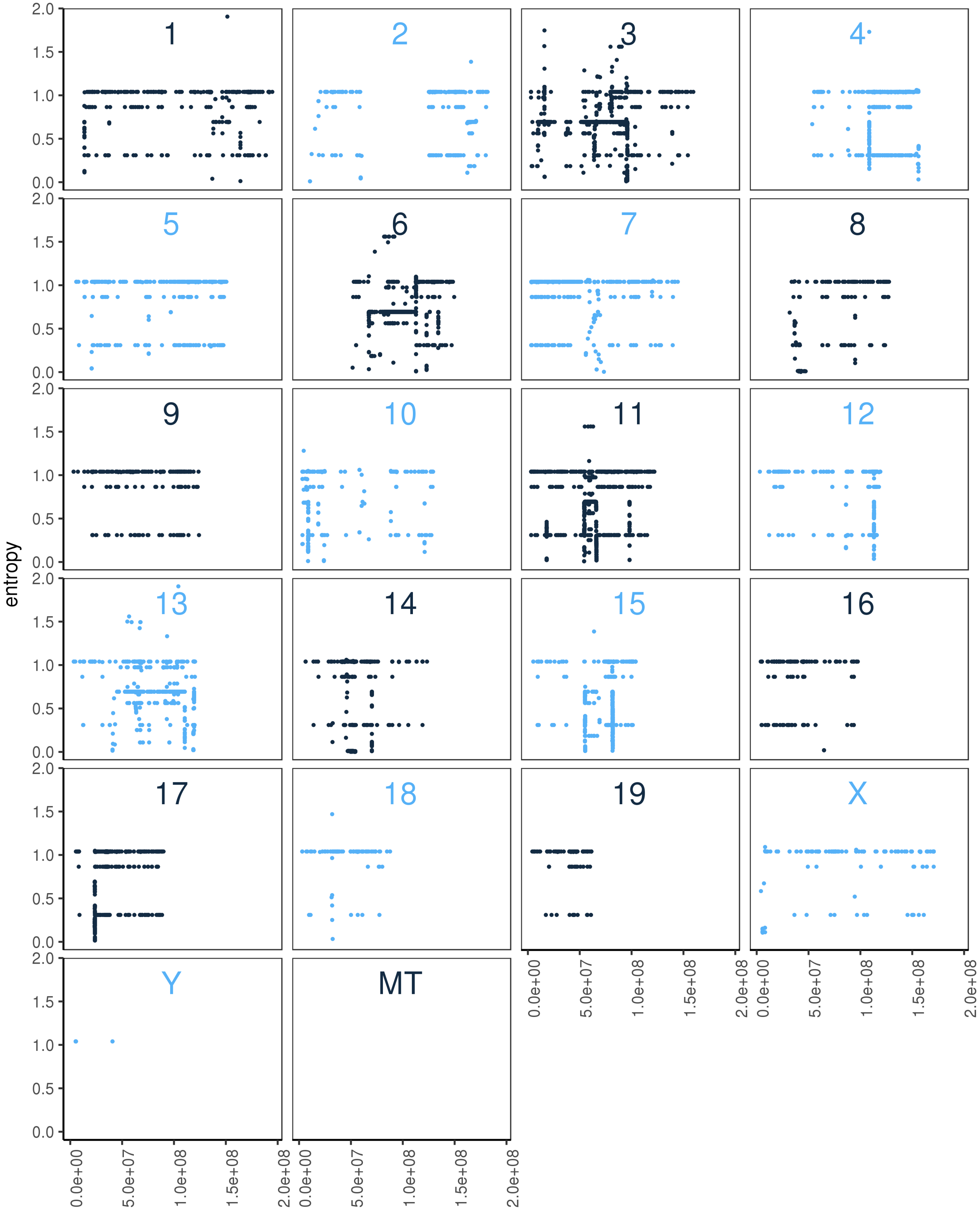

Figure S43 strain CC012, non-zero entropies in exons (+/-100 bp) in all chromosomes. Each point corresponds to the entropy of a variant at that position along the chromosome

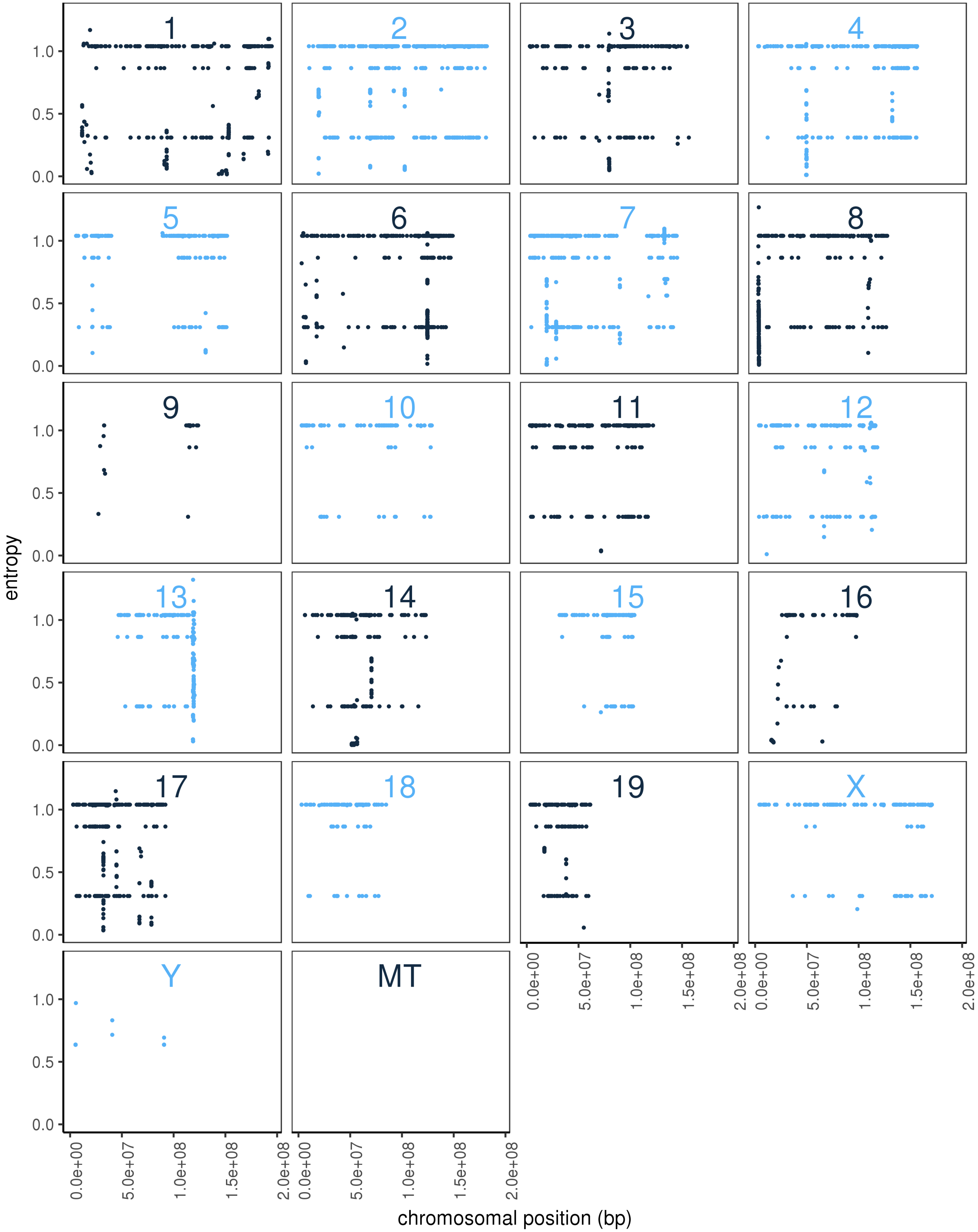

Figure S44 strain CC013, non-zero entropies in exons (+/-100 bp) in all chromosomes. Each point corresponds to the entropy of a variant at that position along the chromosome

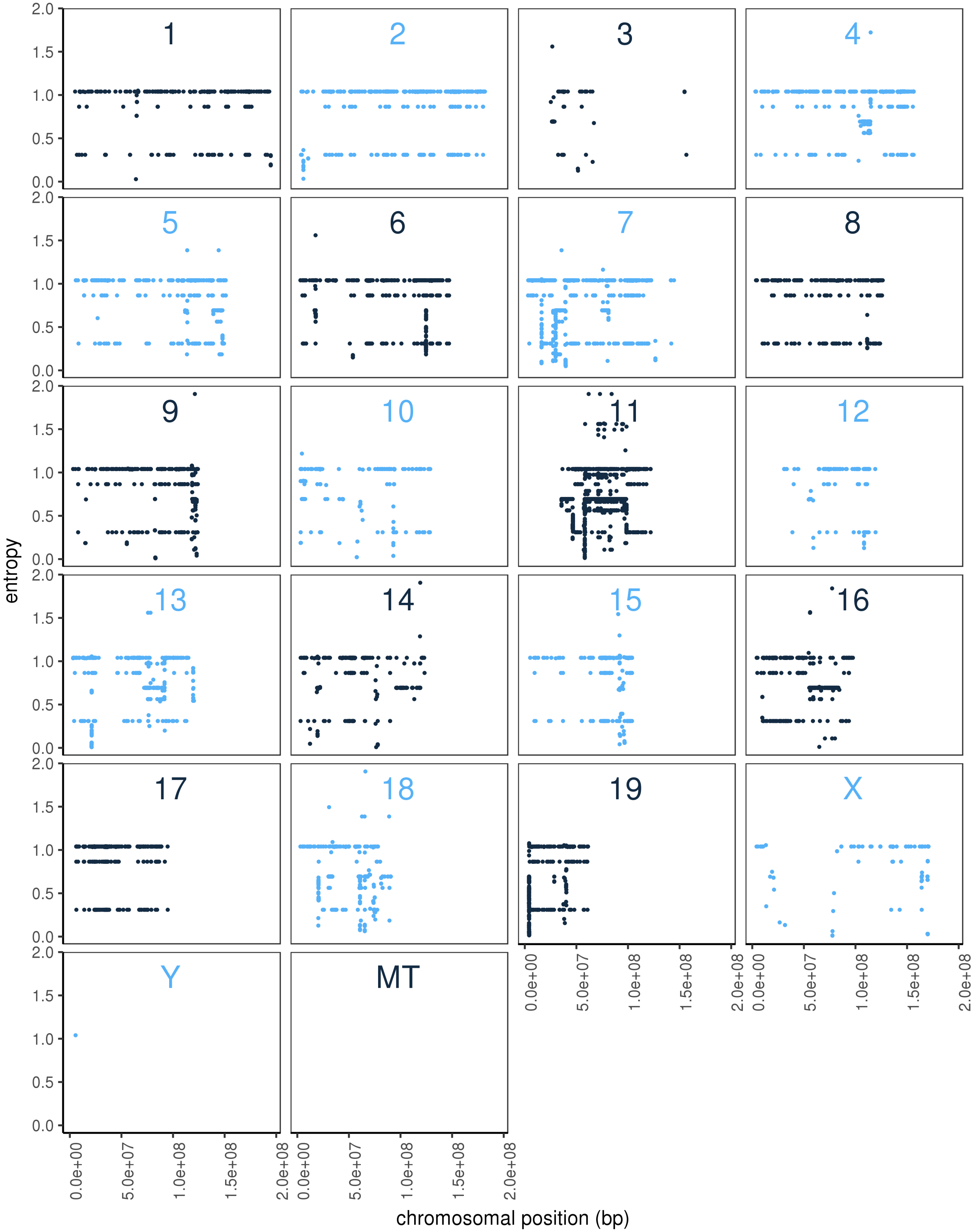

Figure S45 strain CC014, non-zero entropies in exons (+/-100 bp) in all chromosomes. Each point corresponds to the entropy of a variant at that position along the chromosome

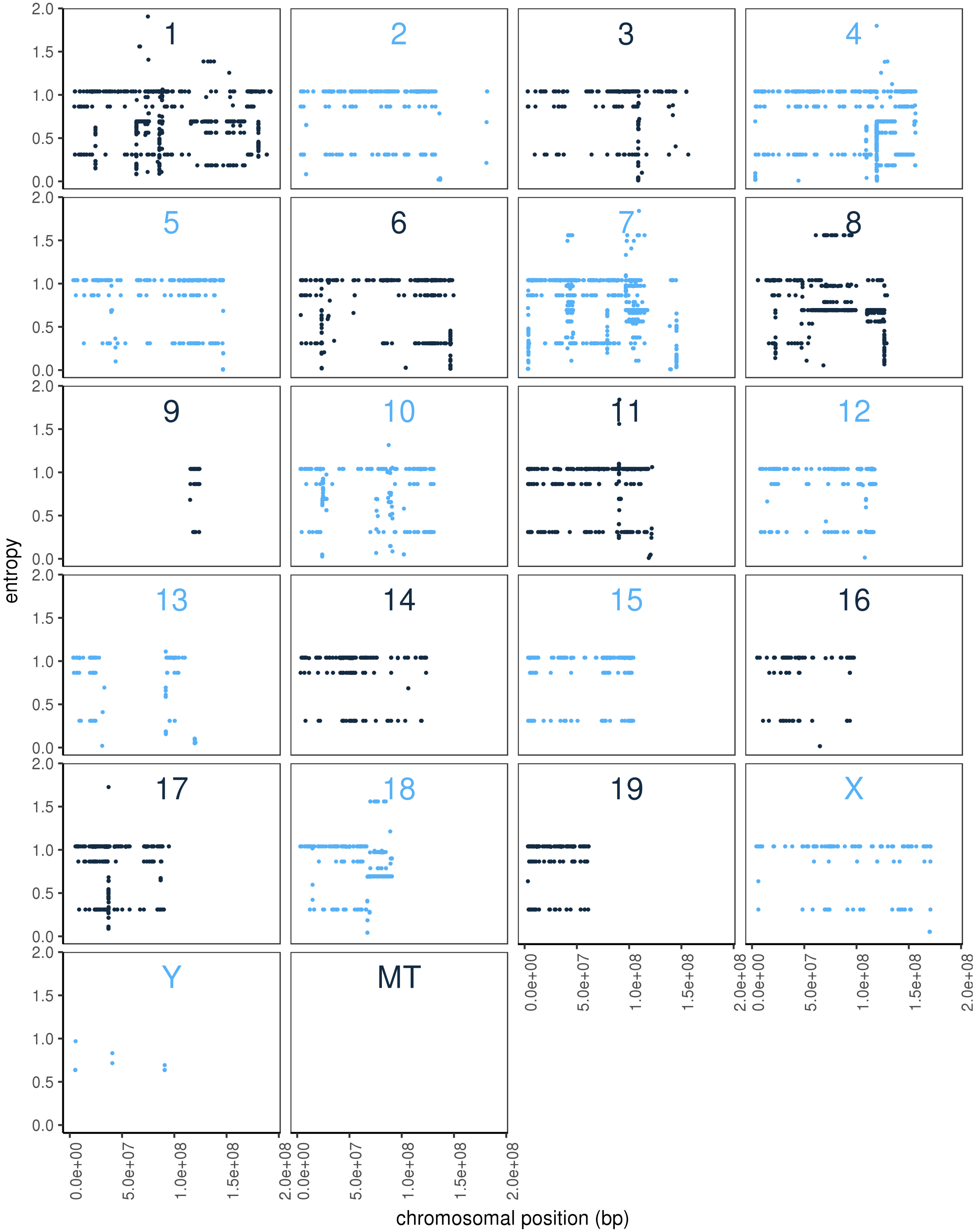

Figure S46 strain CC015, non-zero entropies in exons (+/-100 bp) in all chromosomes. Each point corresponds to the entropy of a variant at that position along the chromosome

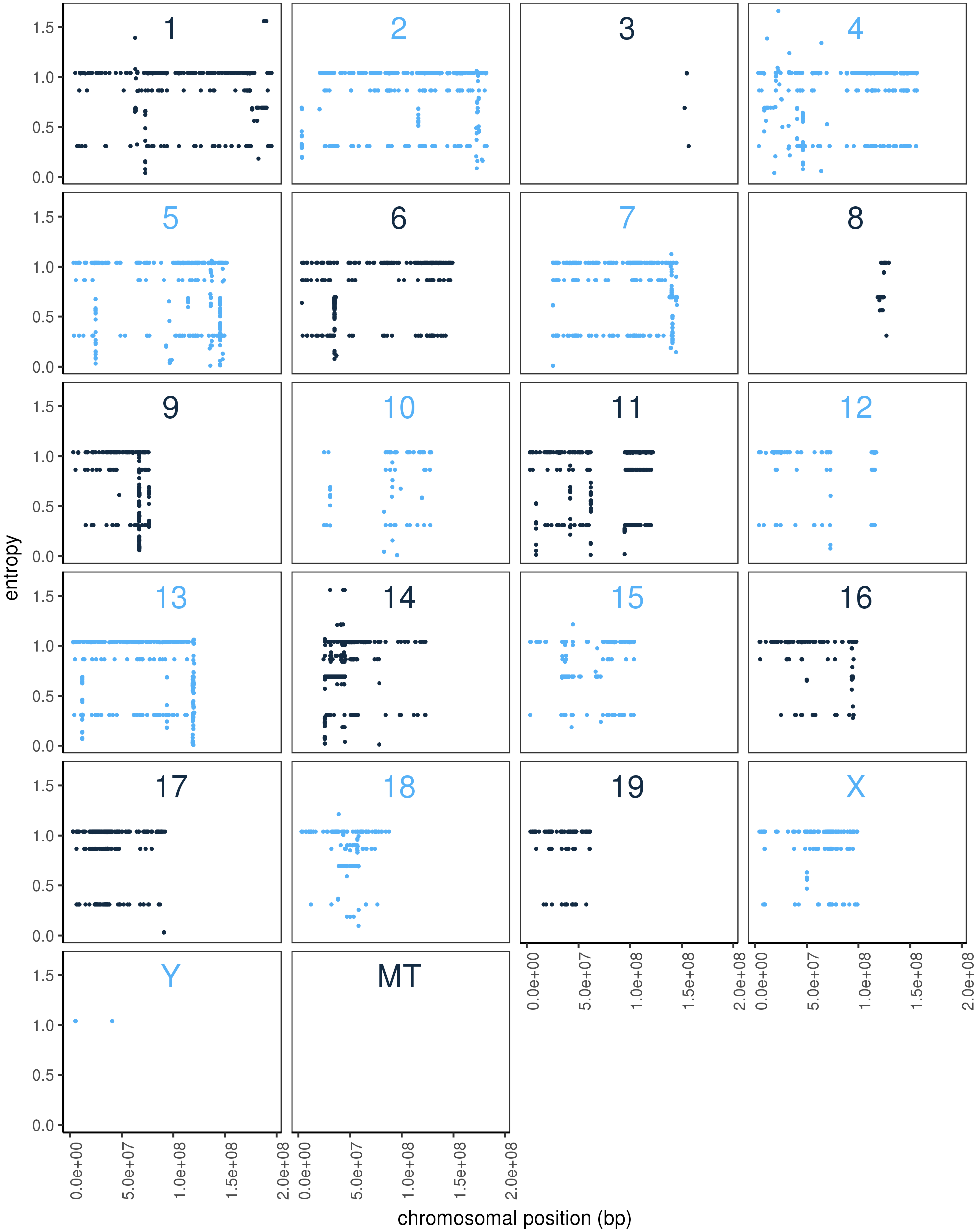

Figure S47 strain CC016, non-zero entropies in exons (+/-100 bp) in all chromosomes. Each point corresponds to the entropy of a variant at that position along the chromosome

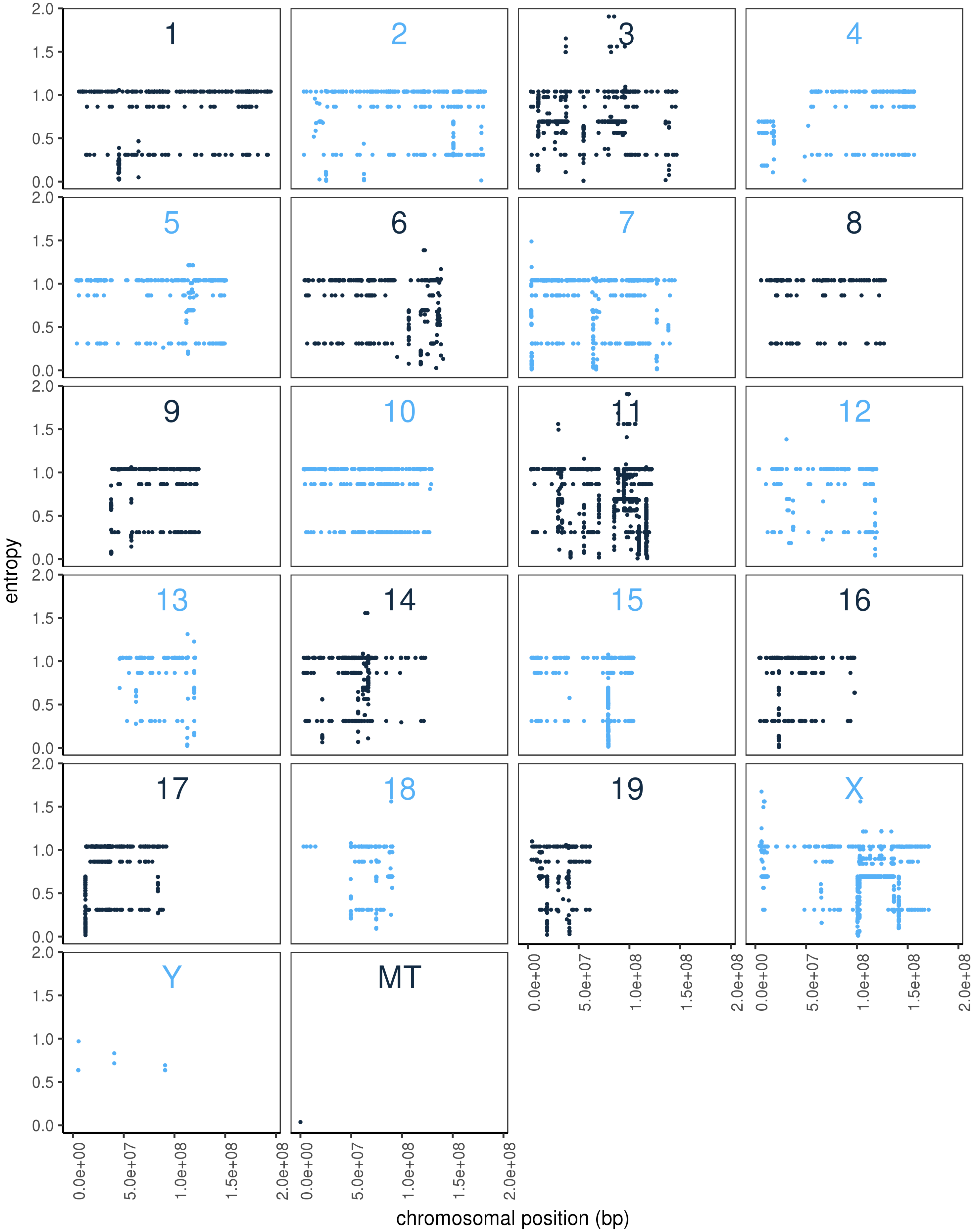

Figure S48 strain CC017, non-zero entropies in exons (+/-100 bp) in all chromosomes. Each point corresponds to the entropy of a variant at that position along the chromosome

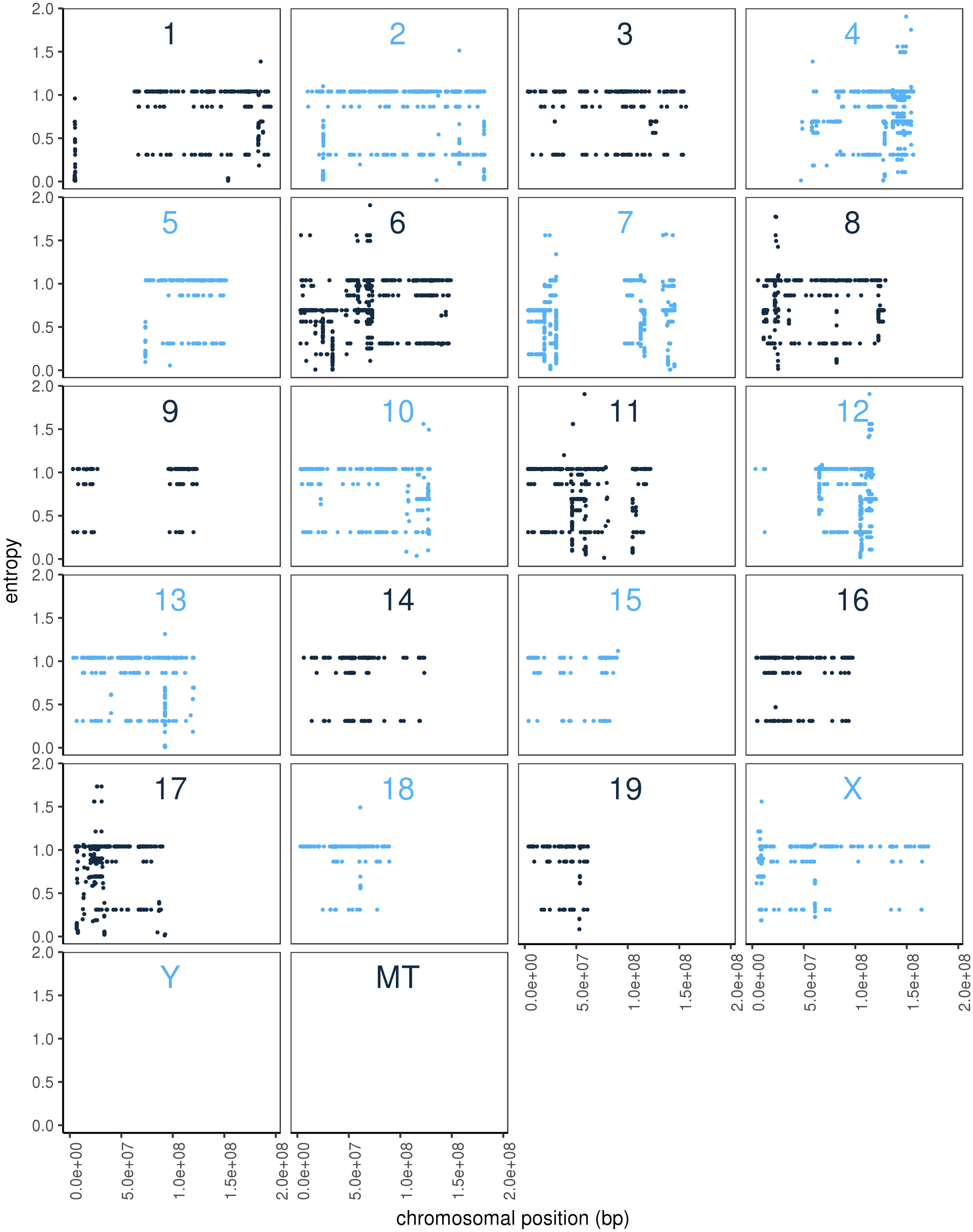

Figure S49 strain CC018, non-zero entropies in exons (+/-100 bp) in all chromosomes. Each point corresponds to the entropy of a variant at that position along the chromosome

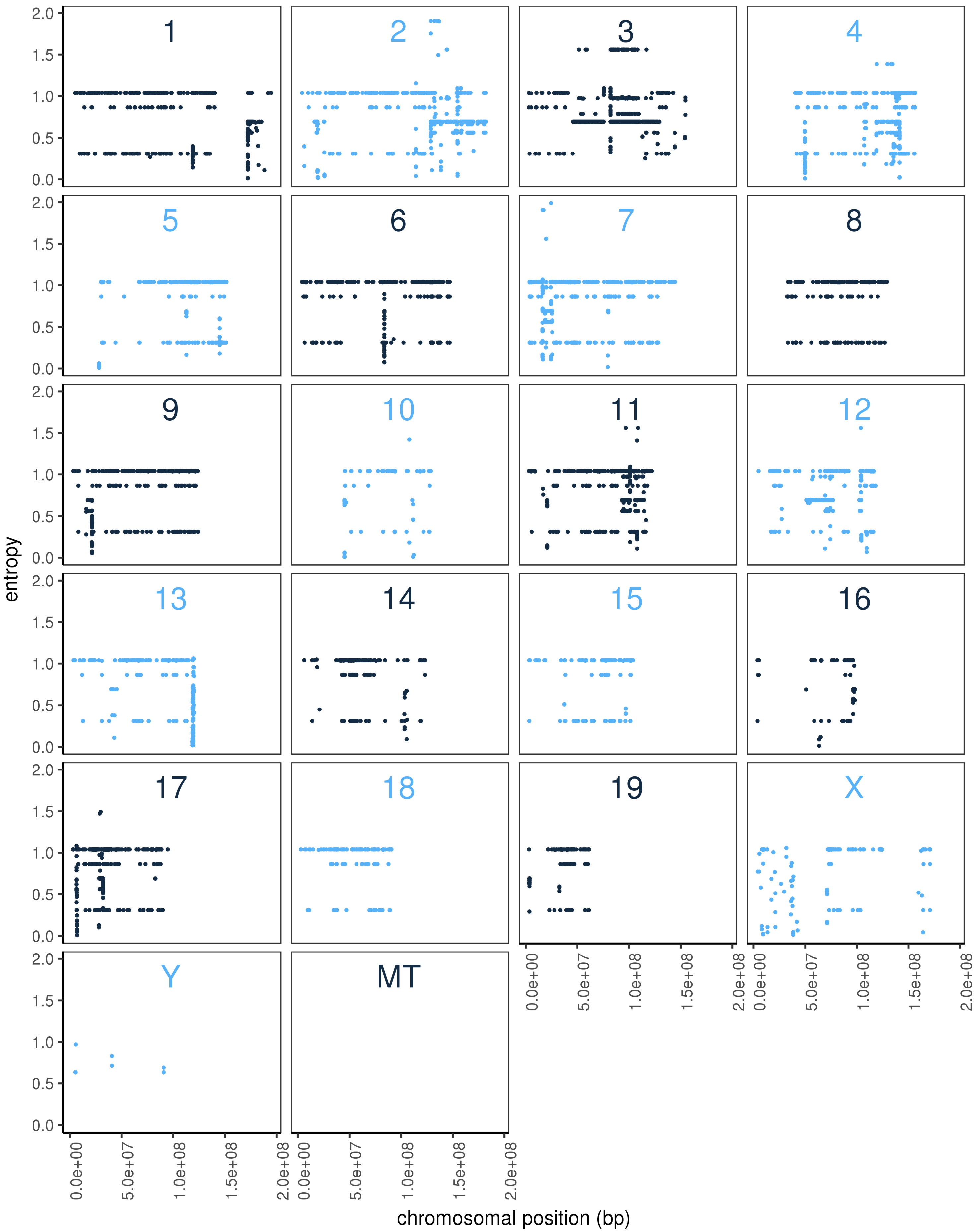

Figure S50 strain CC019, non-zero entropies in exons (+/-100 bp) in all chromosomes. Each point corresponds to the entropy of a variant at that position along the chromosome

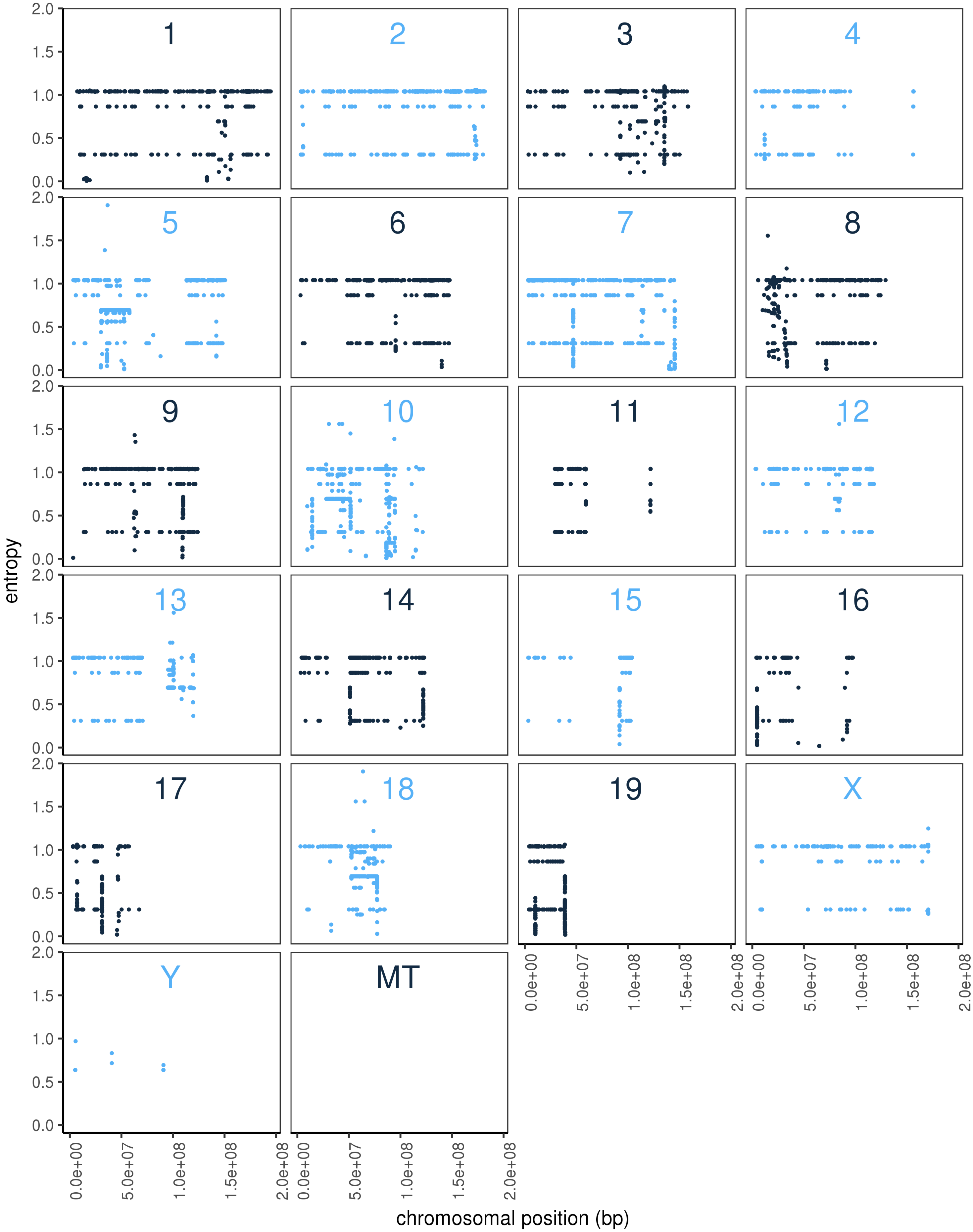

Figure S51 strain CC020, non-zero entropies in exons (+/-100 bp) in all chromosomes. Each point corresponds to the entropy of a variant at that position along the chromosome

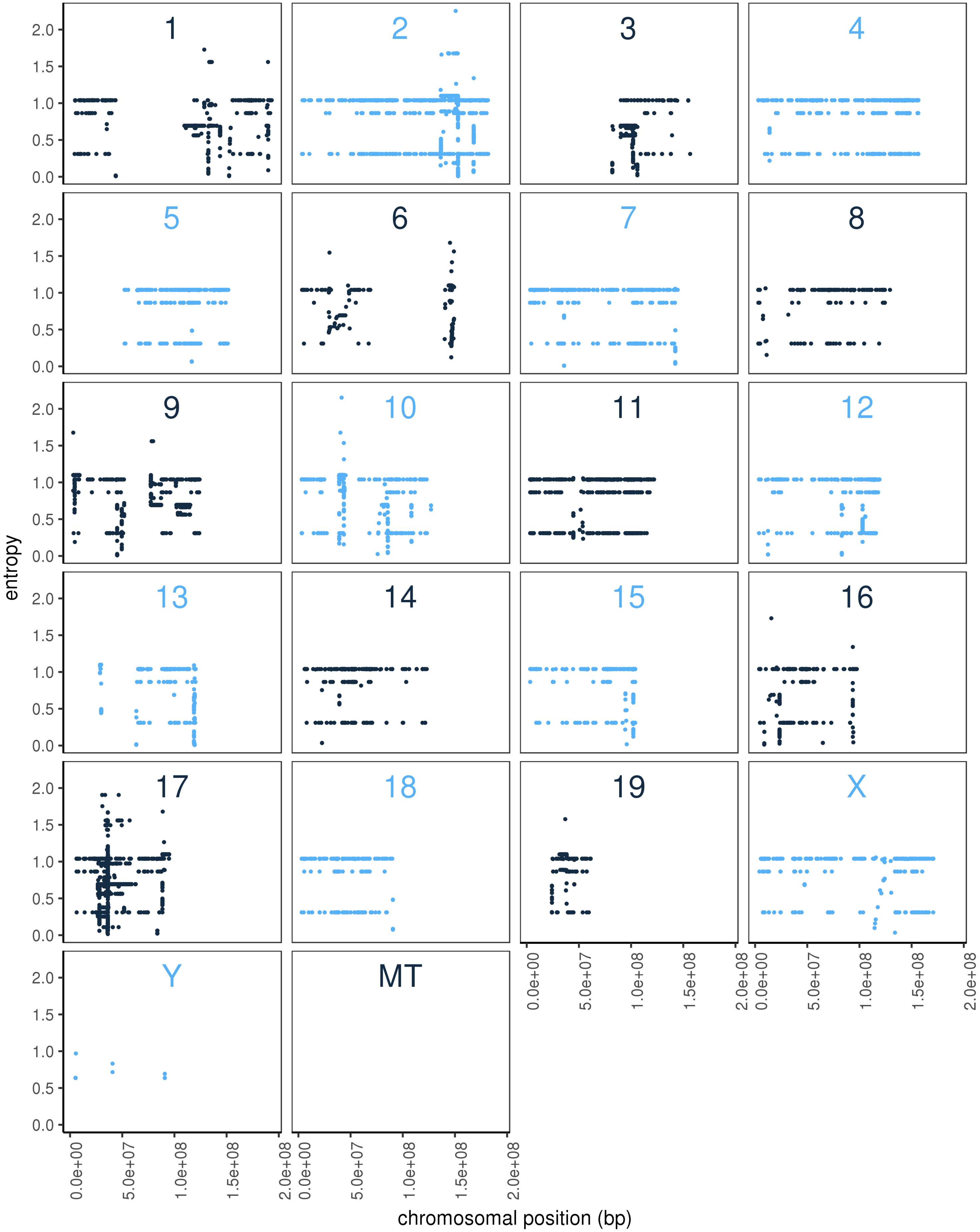

Figure S52 strain CC021, non-zero entropies in exons (+/-100 bp) in all chromosomes. Each point corresponds to the entropy of a variant at that position along the chromosome

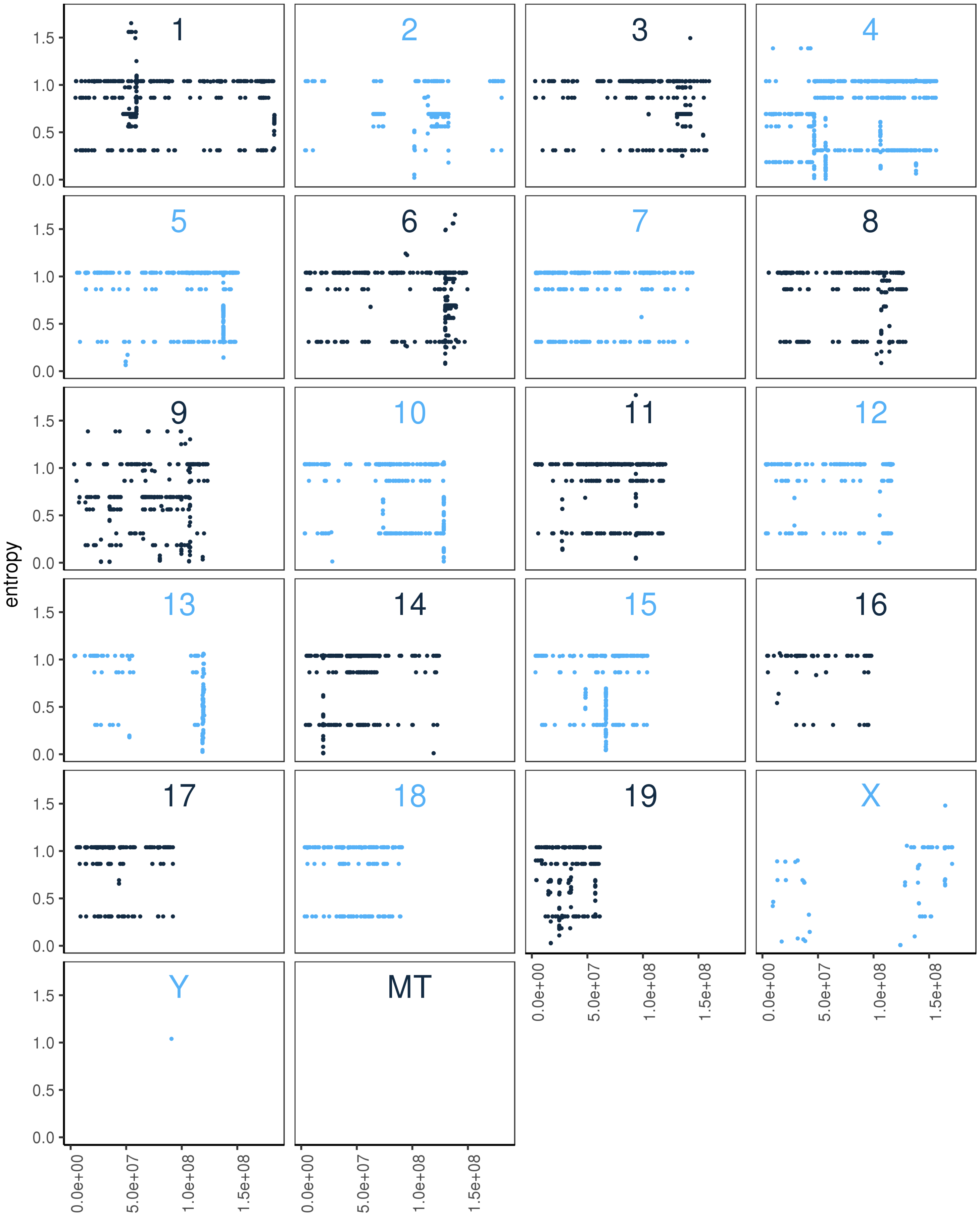

Figure S53 strain CC022, non-zero entropies in exons (+/-100 bp) in all chromosomes. Each point corresponds to the entropy of a variant at that position along the chromosome

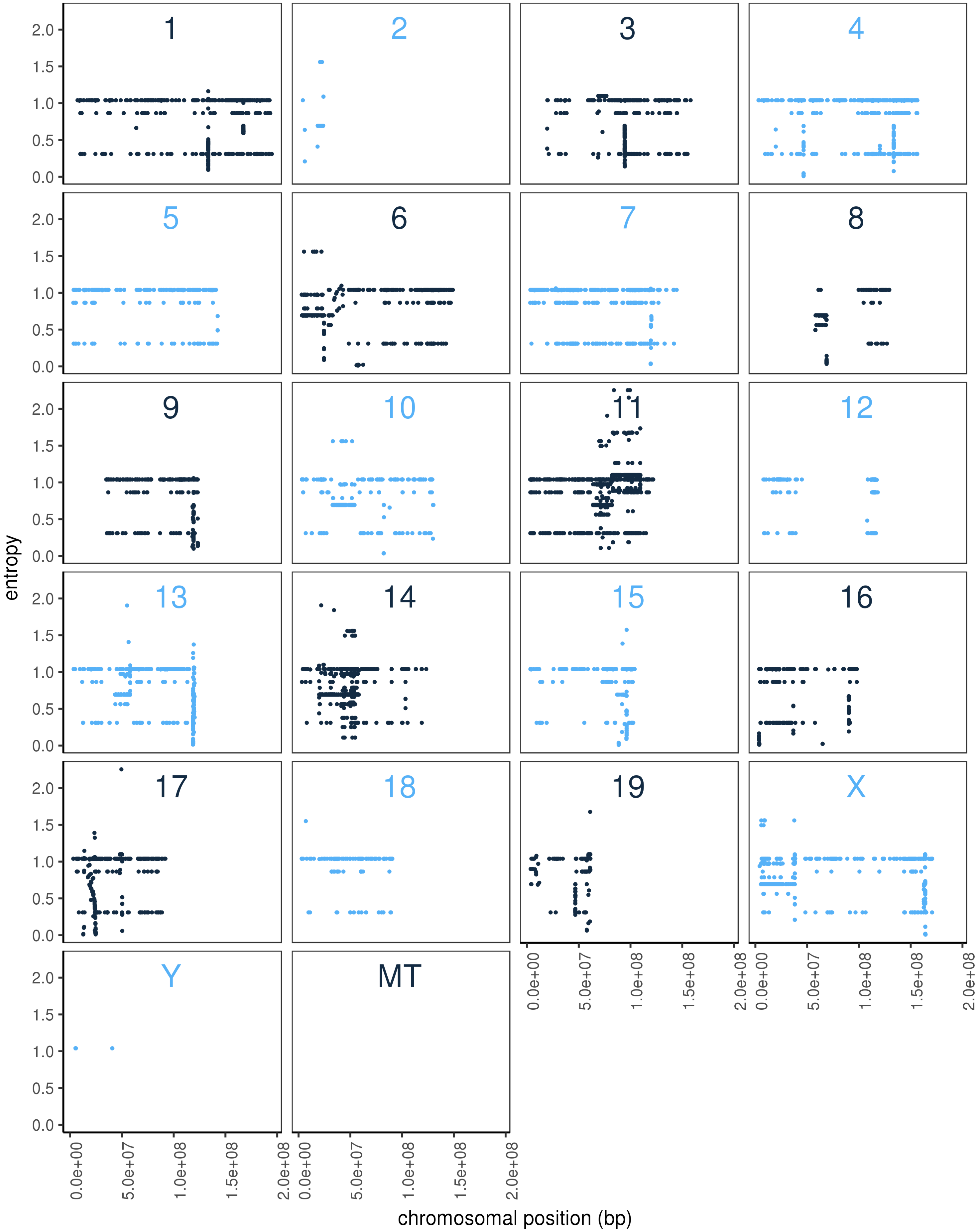

Figure S54 strain CC023, non-zero entropies in exons (+/-100 bp) in all chromosomes. Each point corresponds to the entropy of a variant at that position along the chromosome

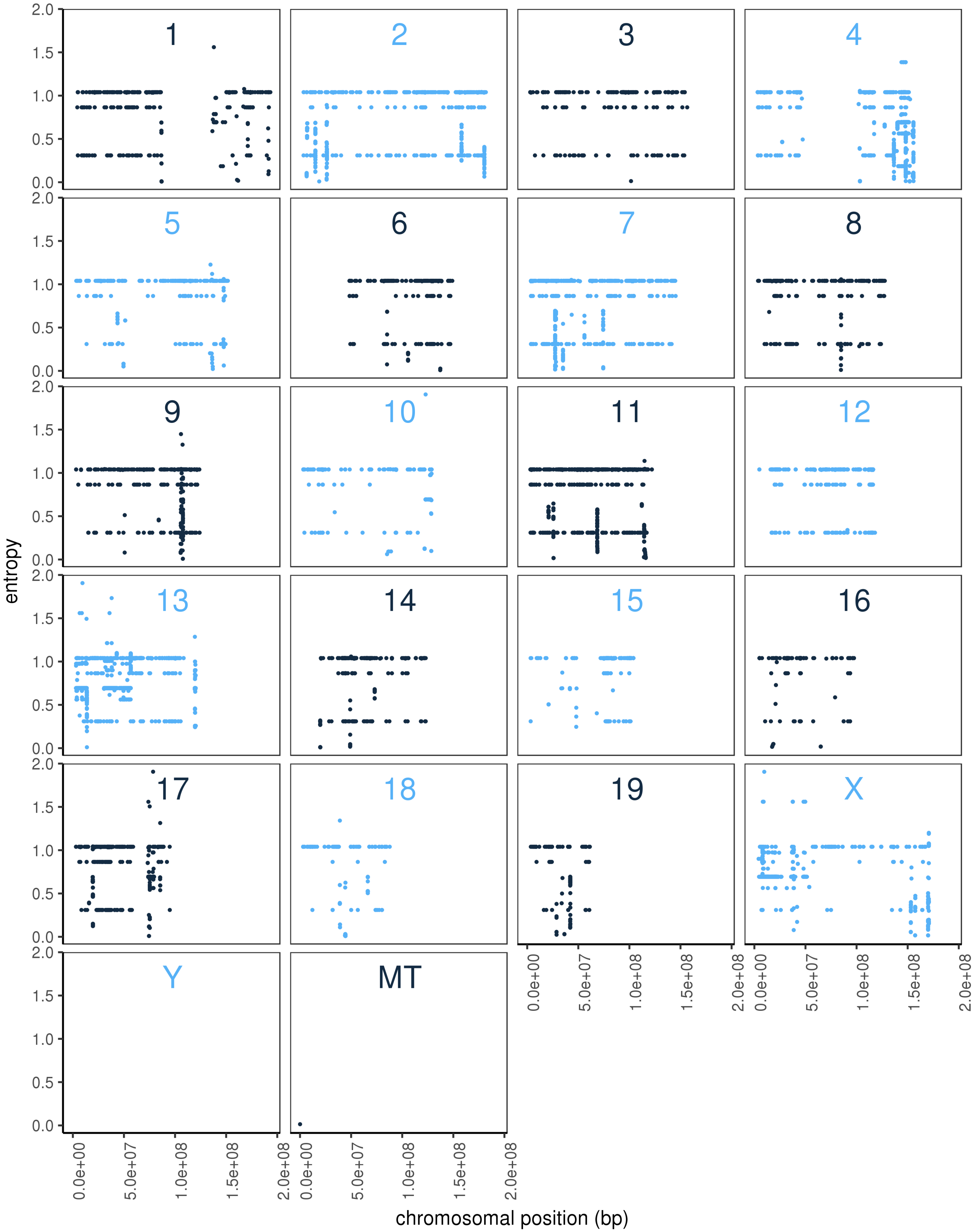

Figure S55 strain CC024, non-zero entropies in exons (+/-100 bp) in all chromosomes. Each point corresponds to the entropy of a variant at that position along the chromosome

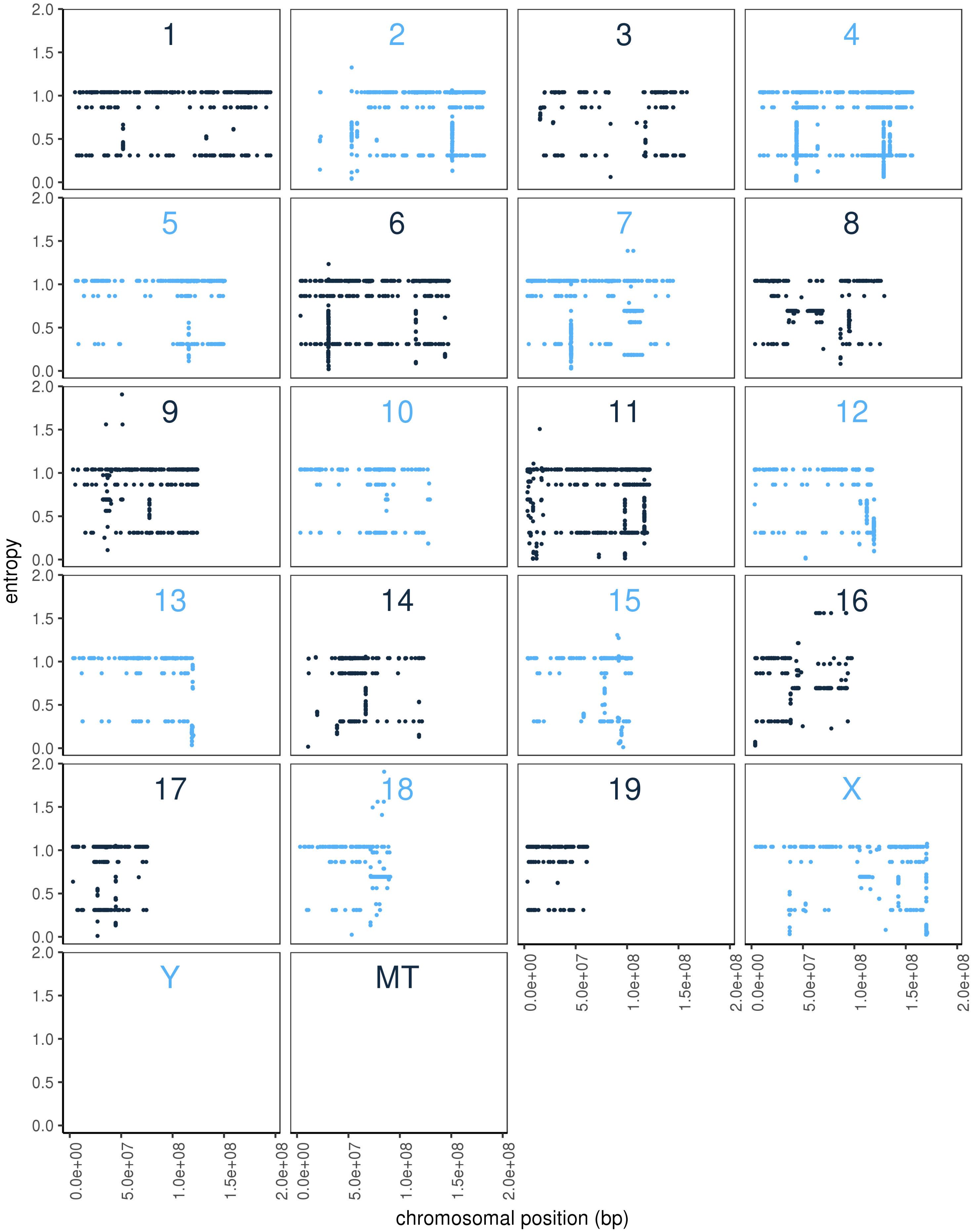

Figure S56 strain CC025, non-zero entropies in exons (+/-100 bp) in all chromosomes. Each point corresponds to the entropy of a variant at that position along the chromosome

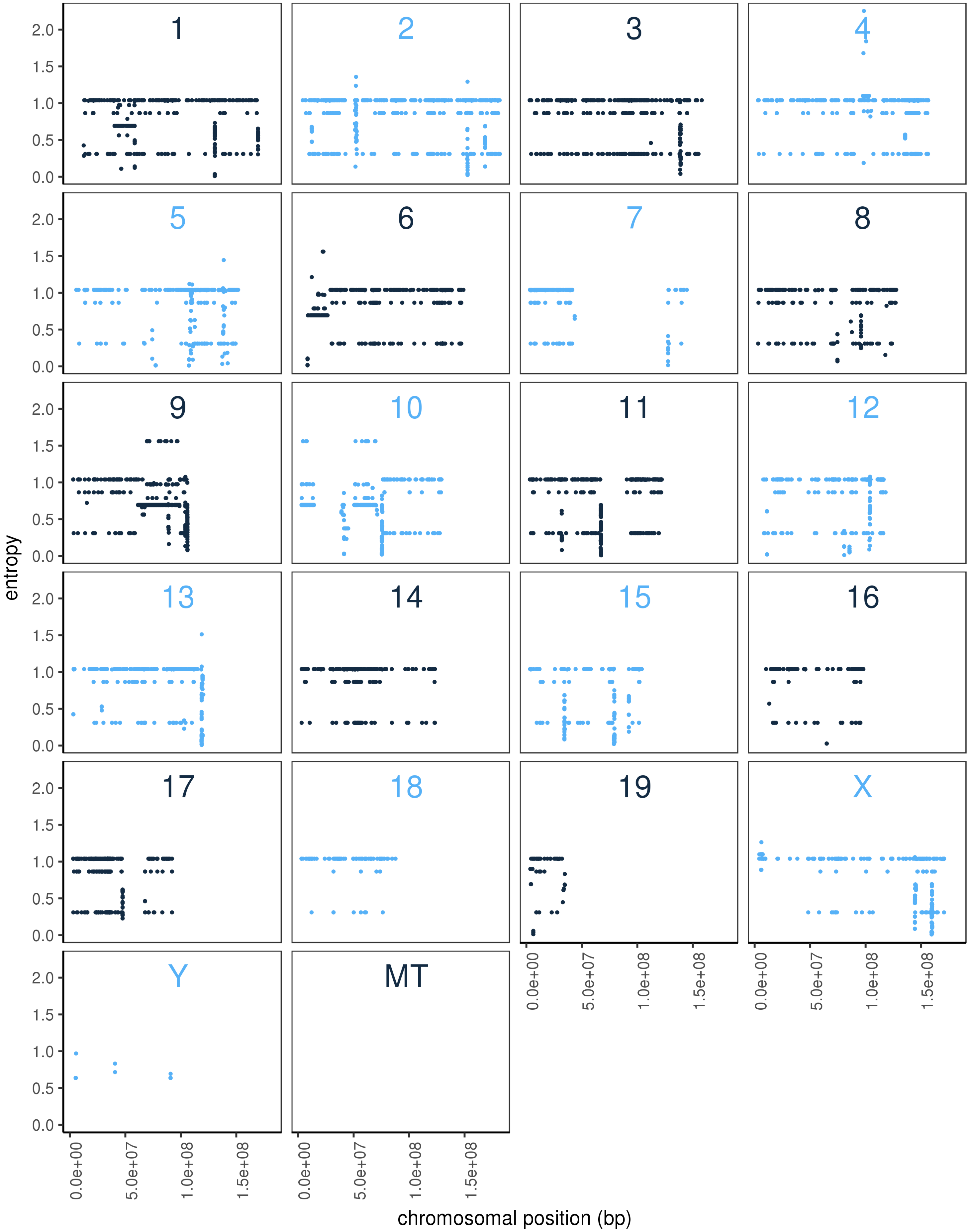

Figure S57 strain CC026, non-zero entropies in exons (+/-100 bp) in all chromosomes. Each point corresponds to the entropy of a variant at that position along the chromosome

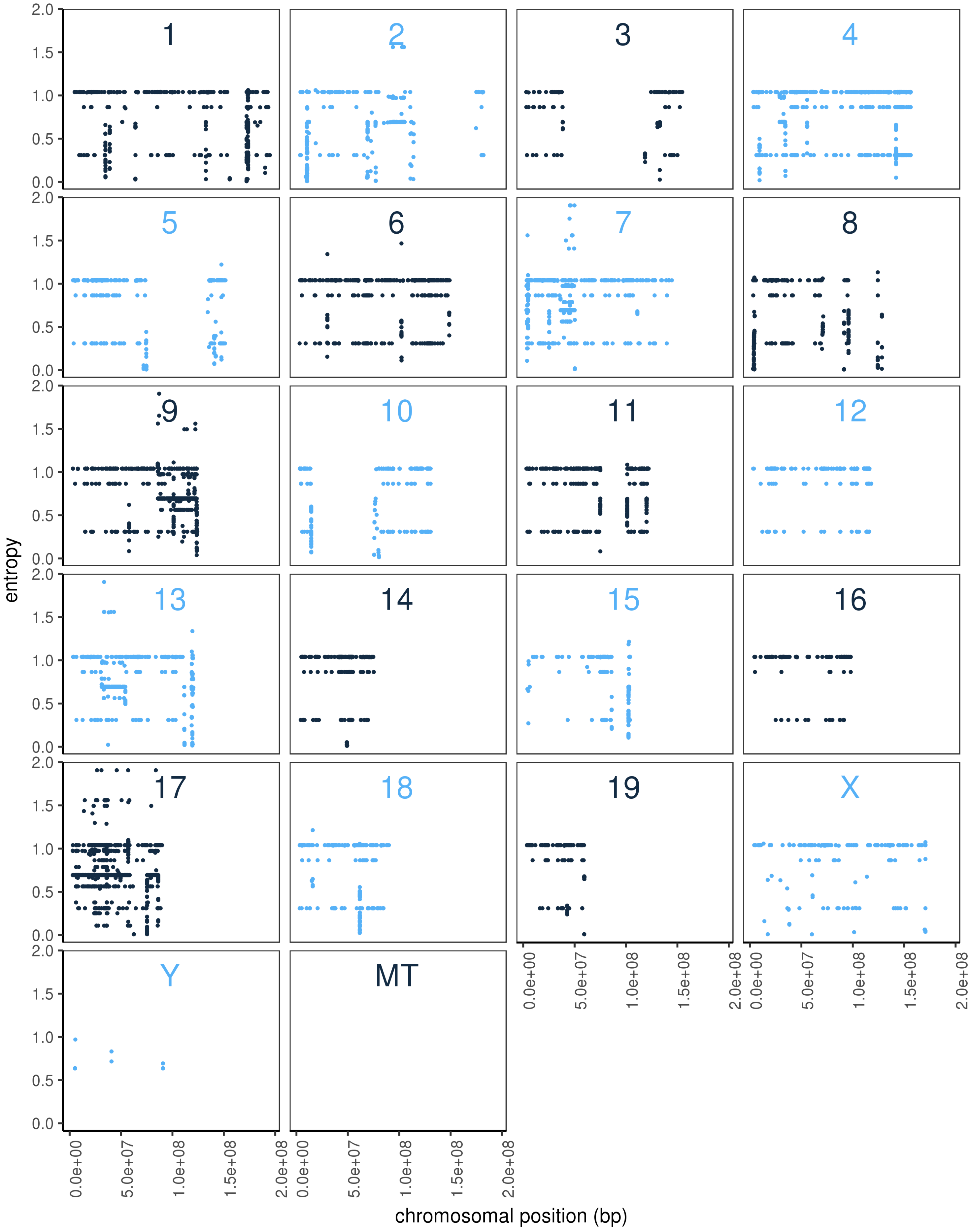

Figure S58 strain CC027, non-zero entropies in exons (+/-100 bp) in all chromosomes. Each point corresponds to the entropy of a variant at that position along the chromosome

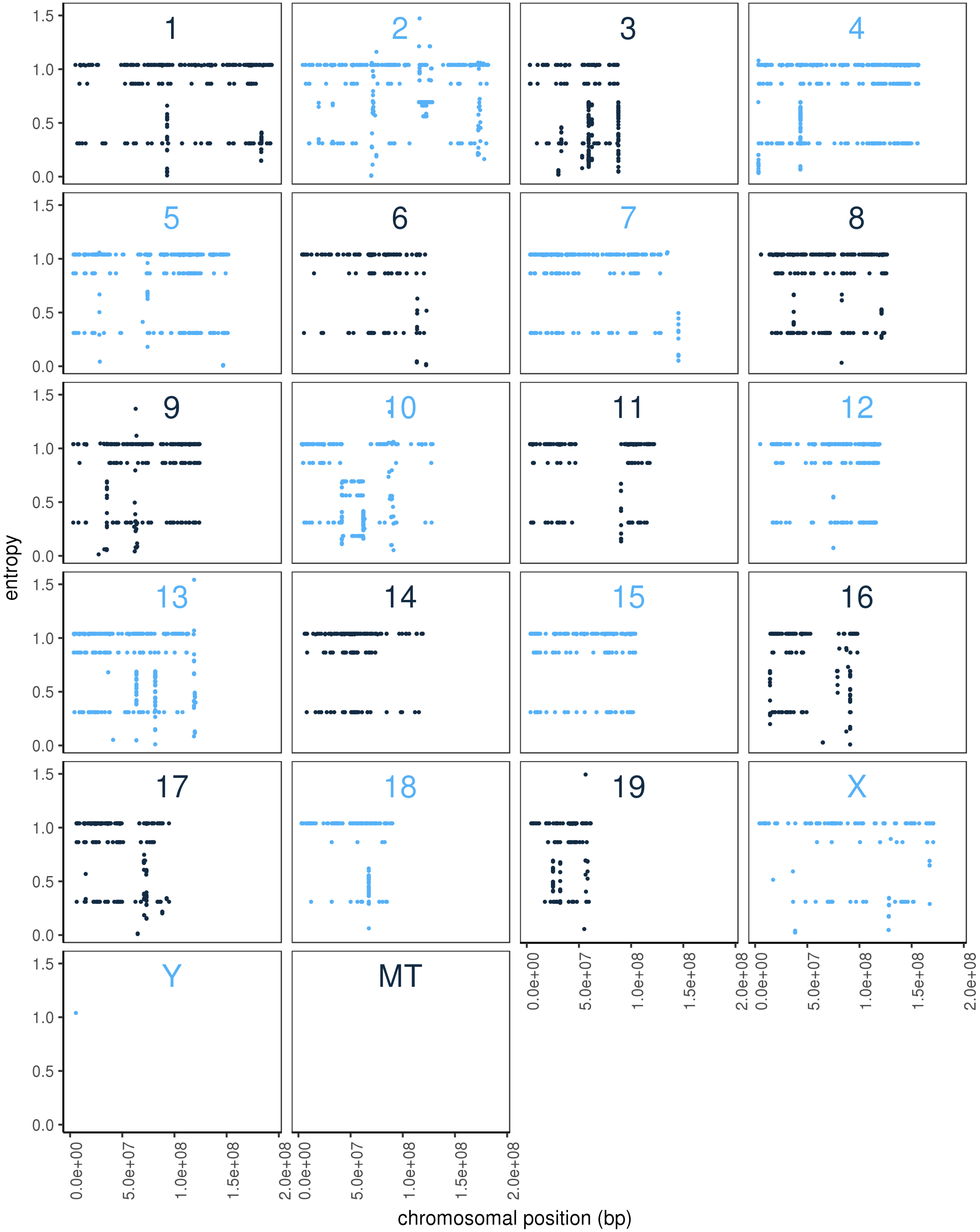

Figure S59 strain CC028, non-zero entropies in exons (+/-100 bp) in all chromosomes. Each point corresponds to the entropy of a variant at that position along the chromosome

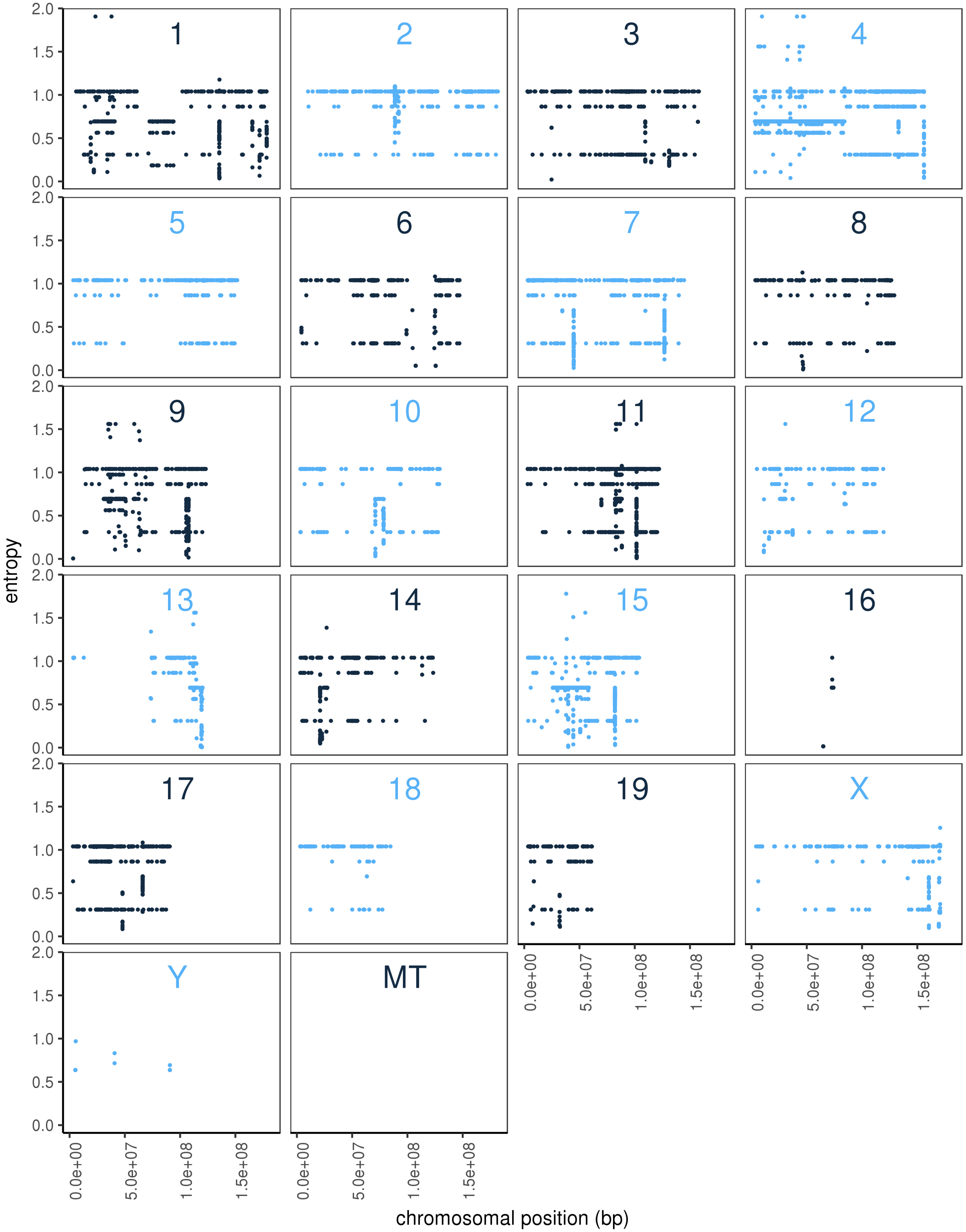

Figure S60 strain CC029, non-zero entropies in exons (+/-100 bp) in all chromosomes. Each point corresponds to the entropy of a variant at that position along the chromosome

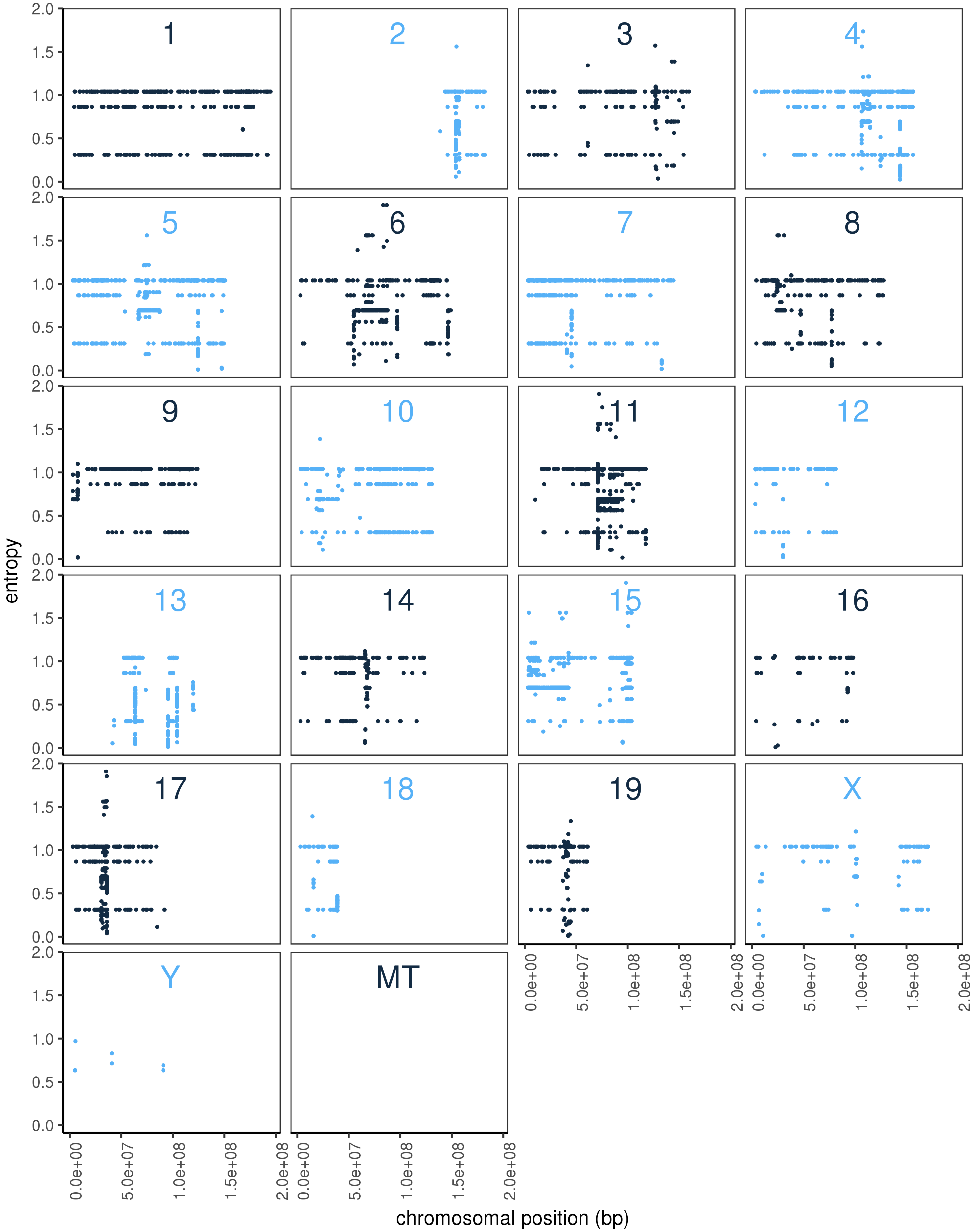

Figure S61 strain CC030, non-zero entropies in exons (+/-100 bp) in all chromosomes. Each point corresponds to the entropy of a variant at that position along the chromosome

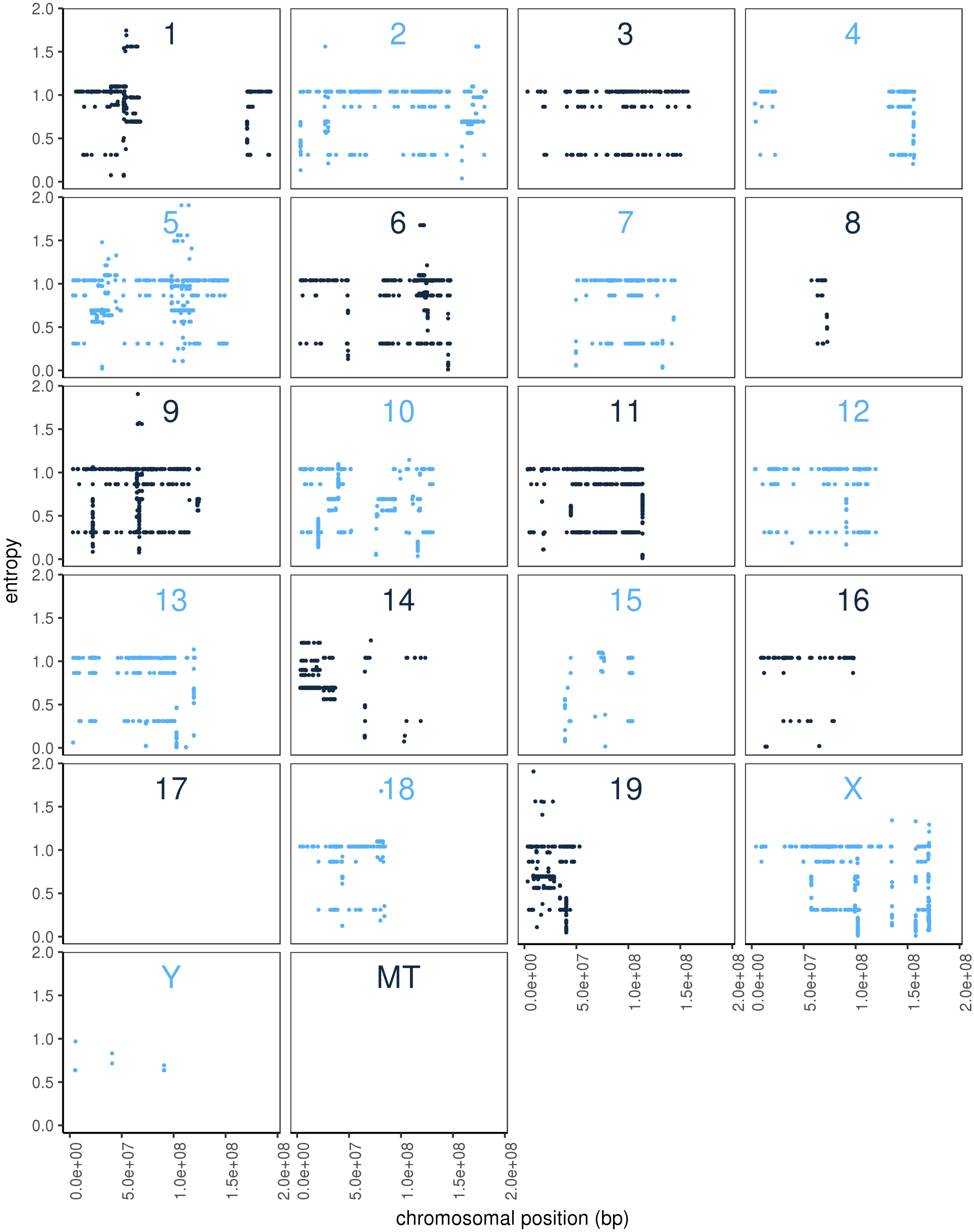

Figure S62 strain CC031, non-zero entropies in exons (+/-100 bp) in all chromosomes. Each point corresponds to the entropy of a variant at that position along the chromosome

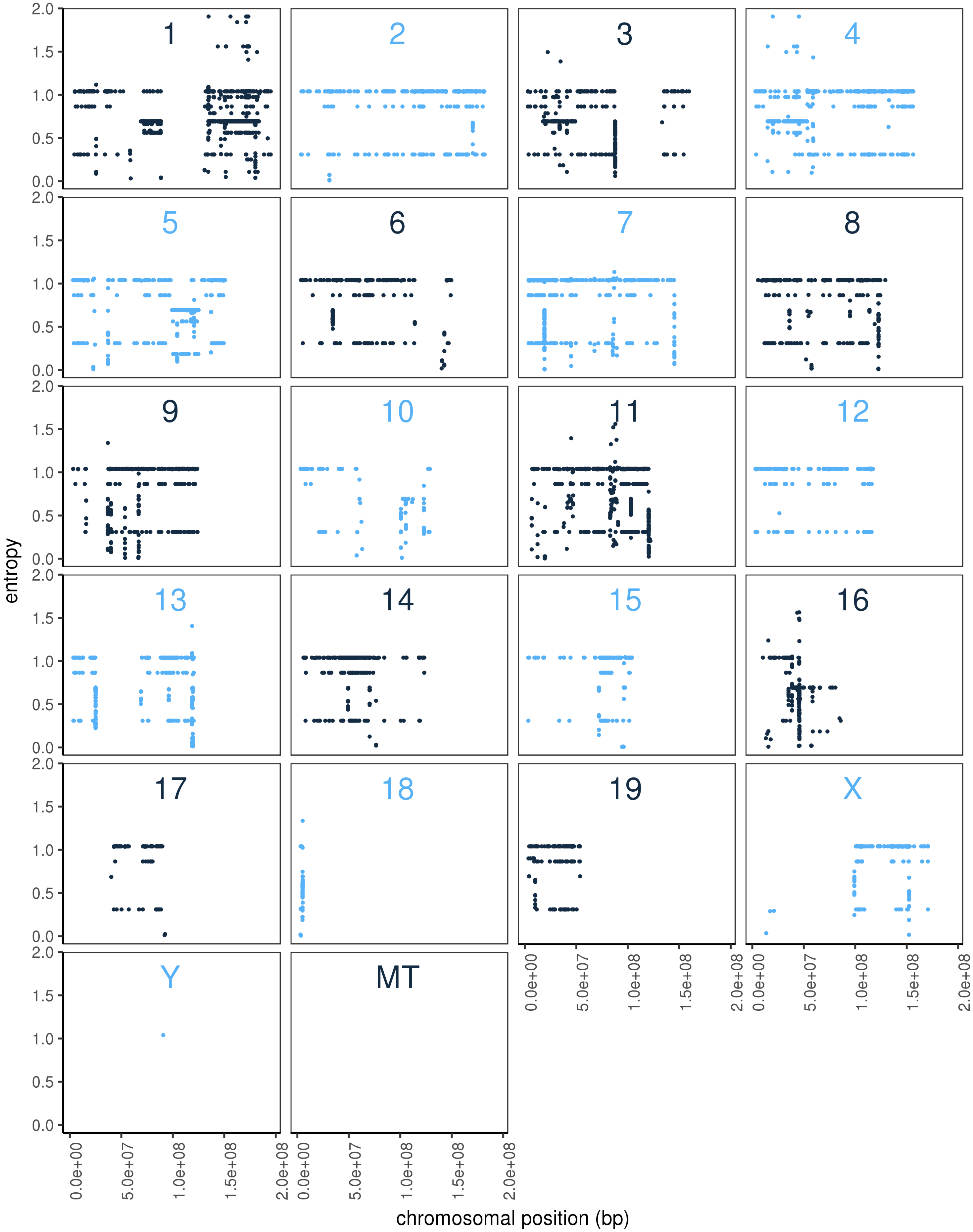

Figure S63 strain CC032, non-zero entropies in exons (+/-100 bp) in all chromosomes. Each point corresponds to the entropy of a variant at that position along the chromosome

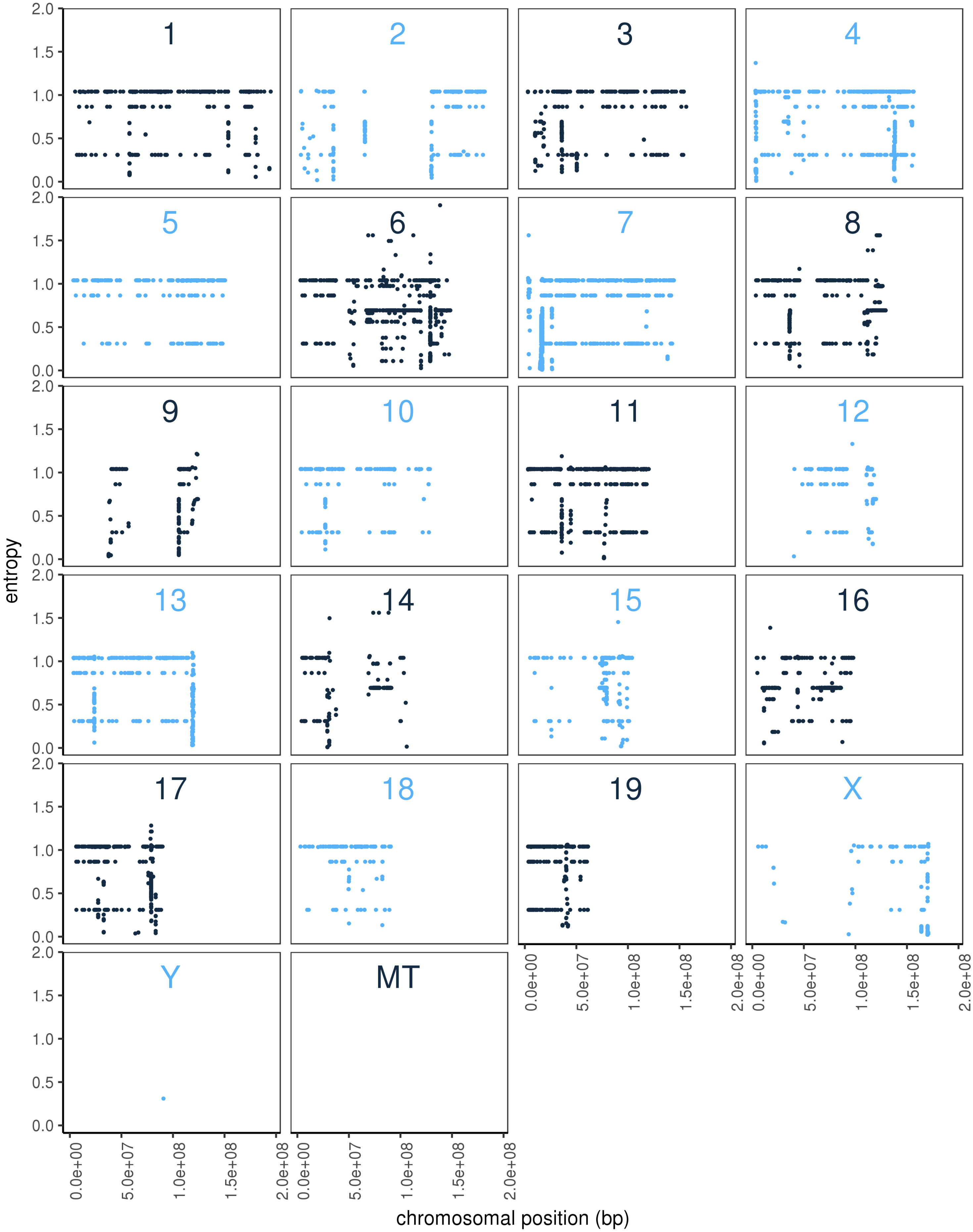

Figure S64 strain CC033, non-zero entropies in exons (+/-100 bp) in all chromosomes. Each point corresponds to the entropy of a variant at that position along the chromosome

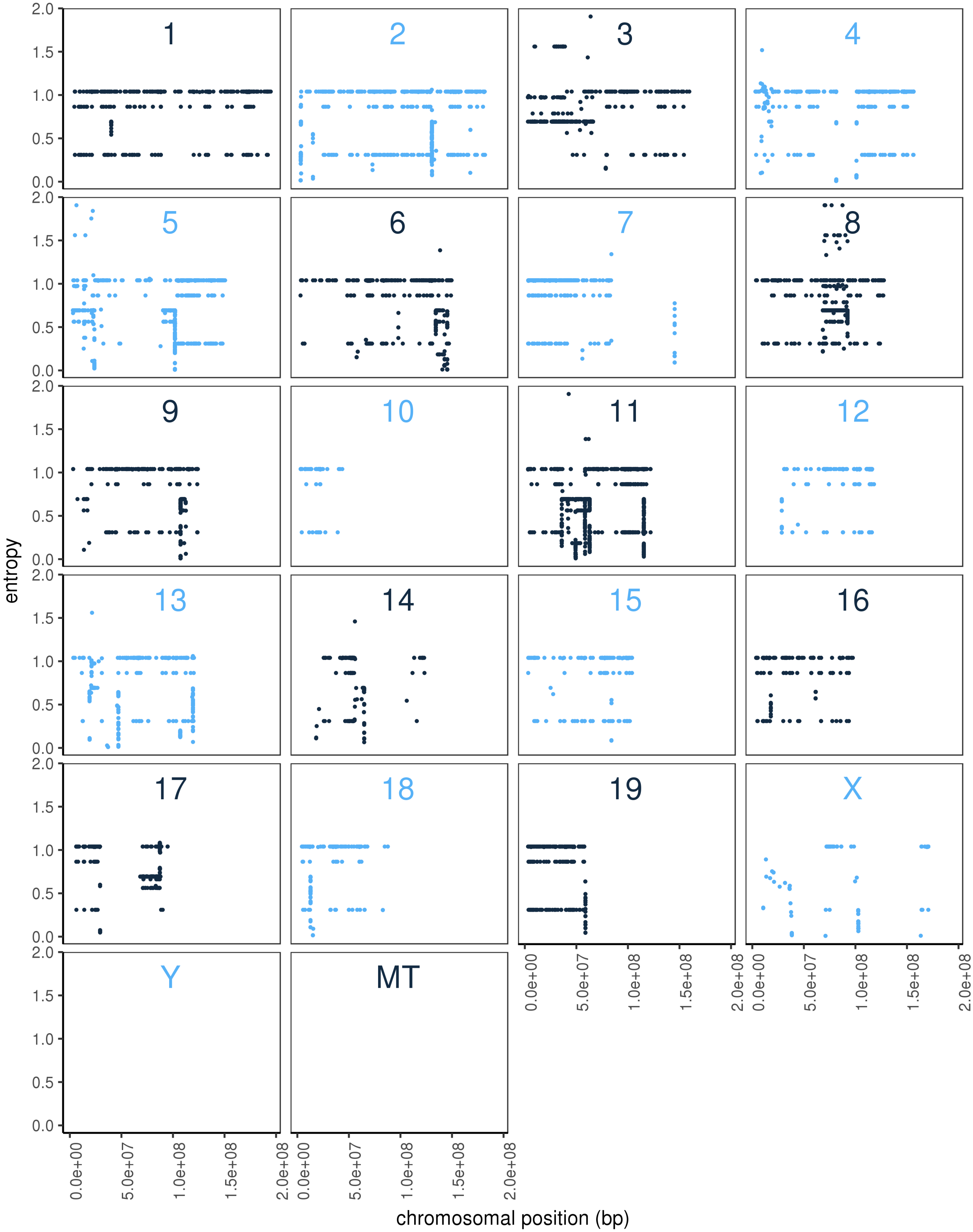

Figure S65 strain CC034, non-zero entropies in exons (+/-100 bp) in all chromosomes. Each point corresponds to the entropy of a variant at that position along the chromosome

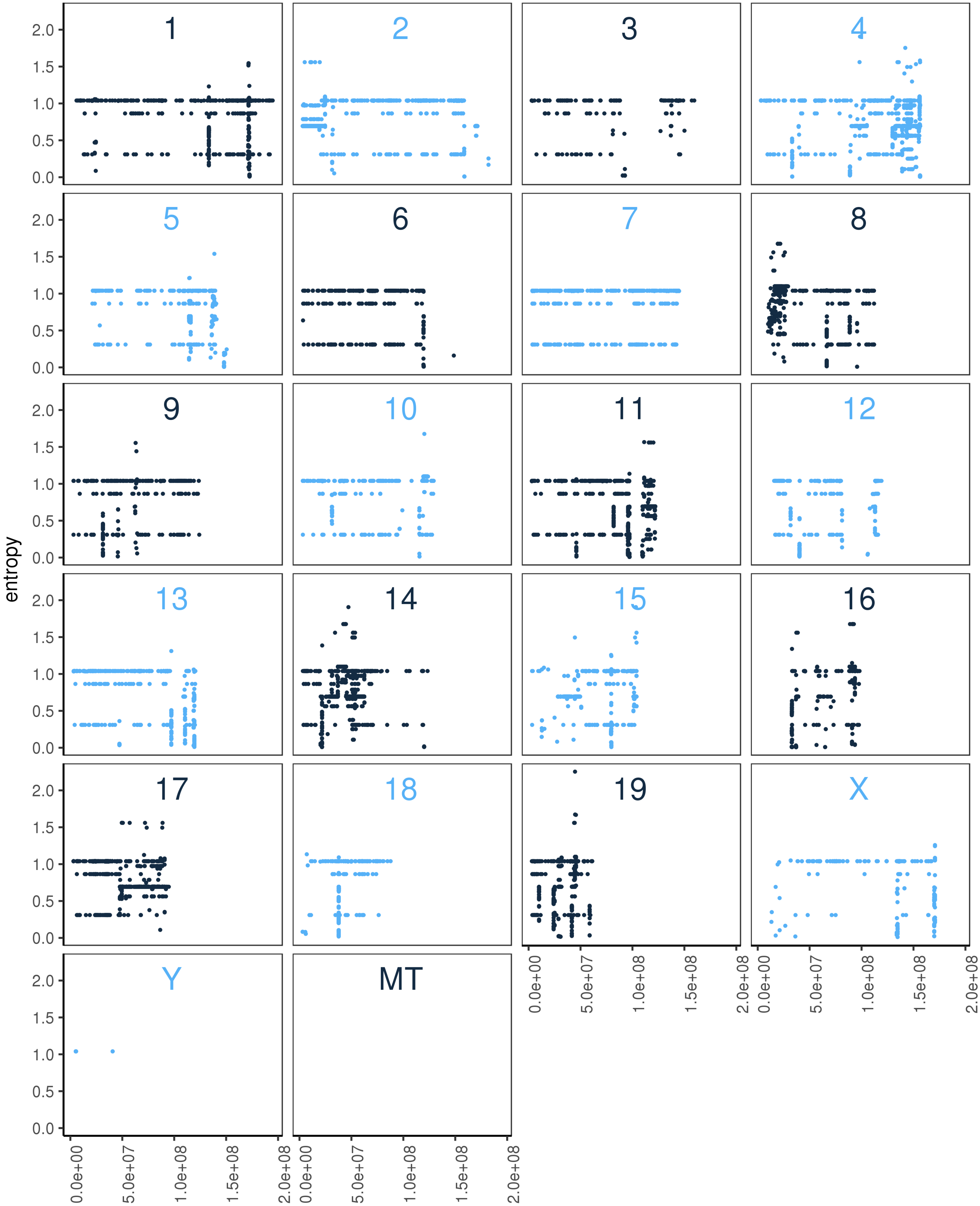

Figure S66 strain CC035, non-zero entropies in exons (+/-100 bp) in all chromosomes. Each point corresponds to the entropy of a variant at that position along the chromosome

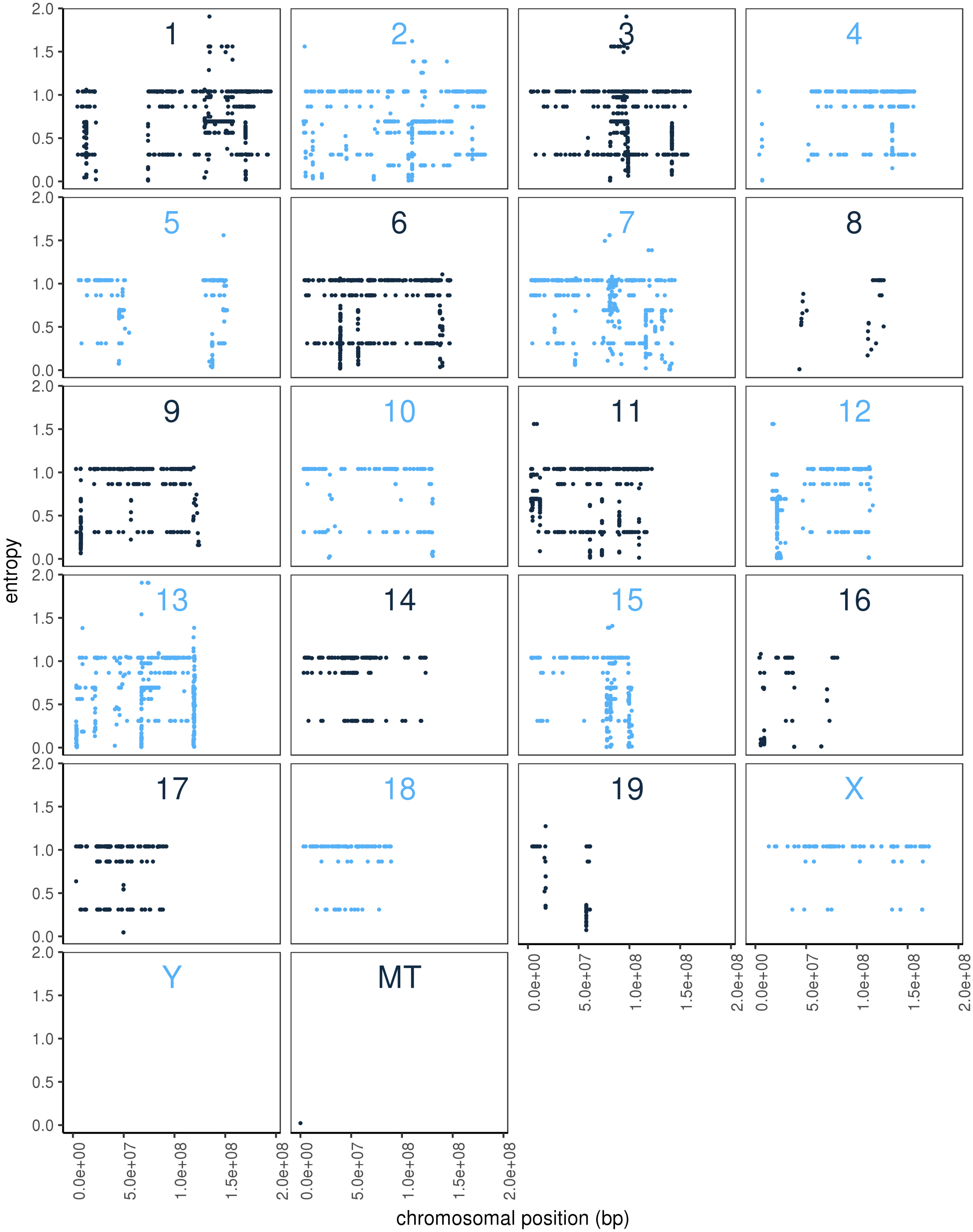

Figure S67 strain CC036, non-zero entropies in exons (+/-100 bp) in all chromosomes. Each point corresponds to the entropy of a variant at that position along the chromosome

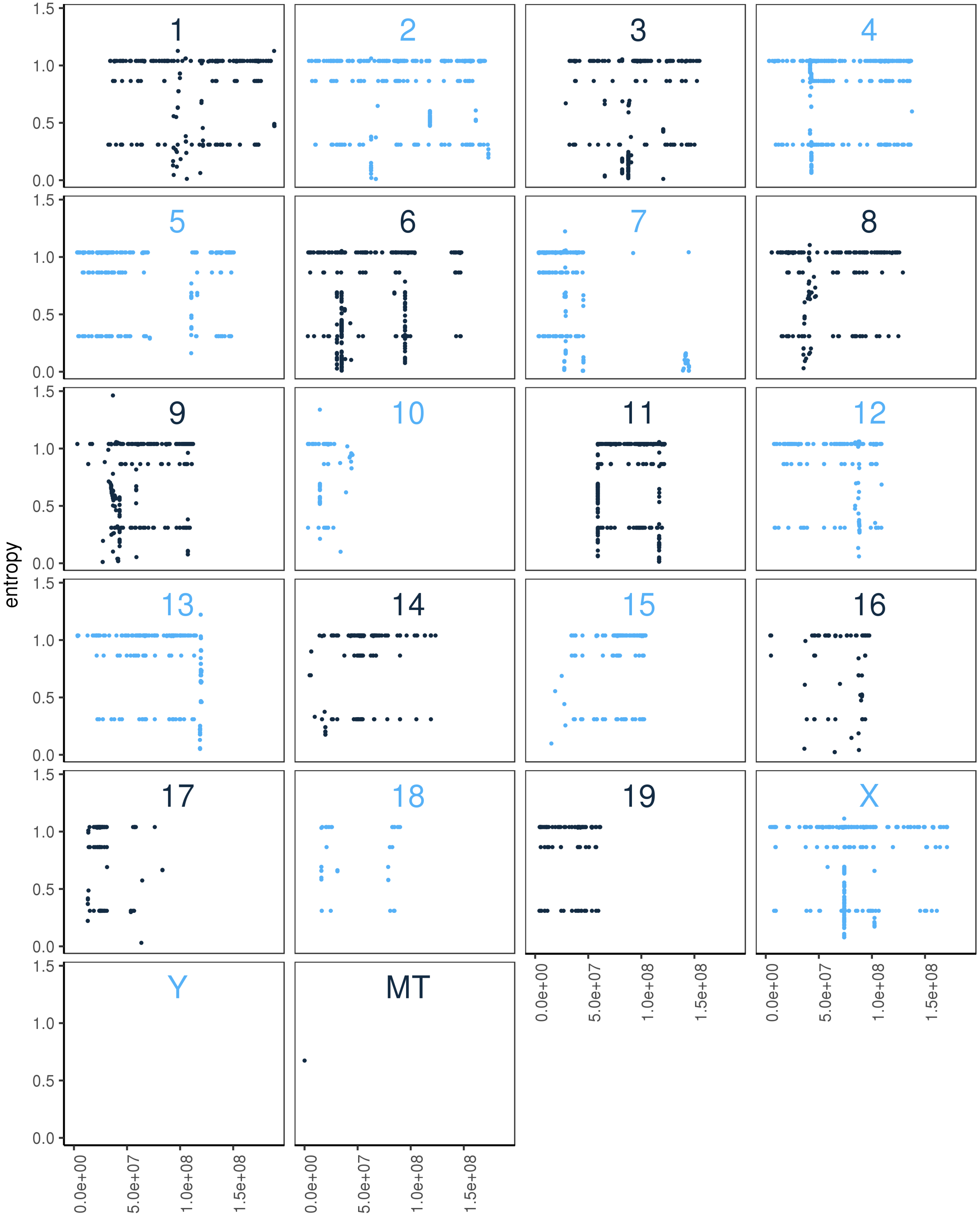

Figure S68 strain CC037, non-zero entropies in exons (+/-100 bp) in all chromosomes. Each point corresponds to the entropy of a variant at that position along the chromosome

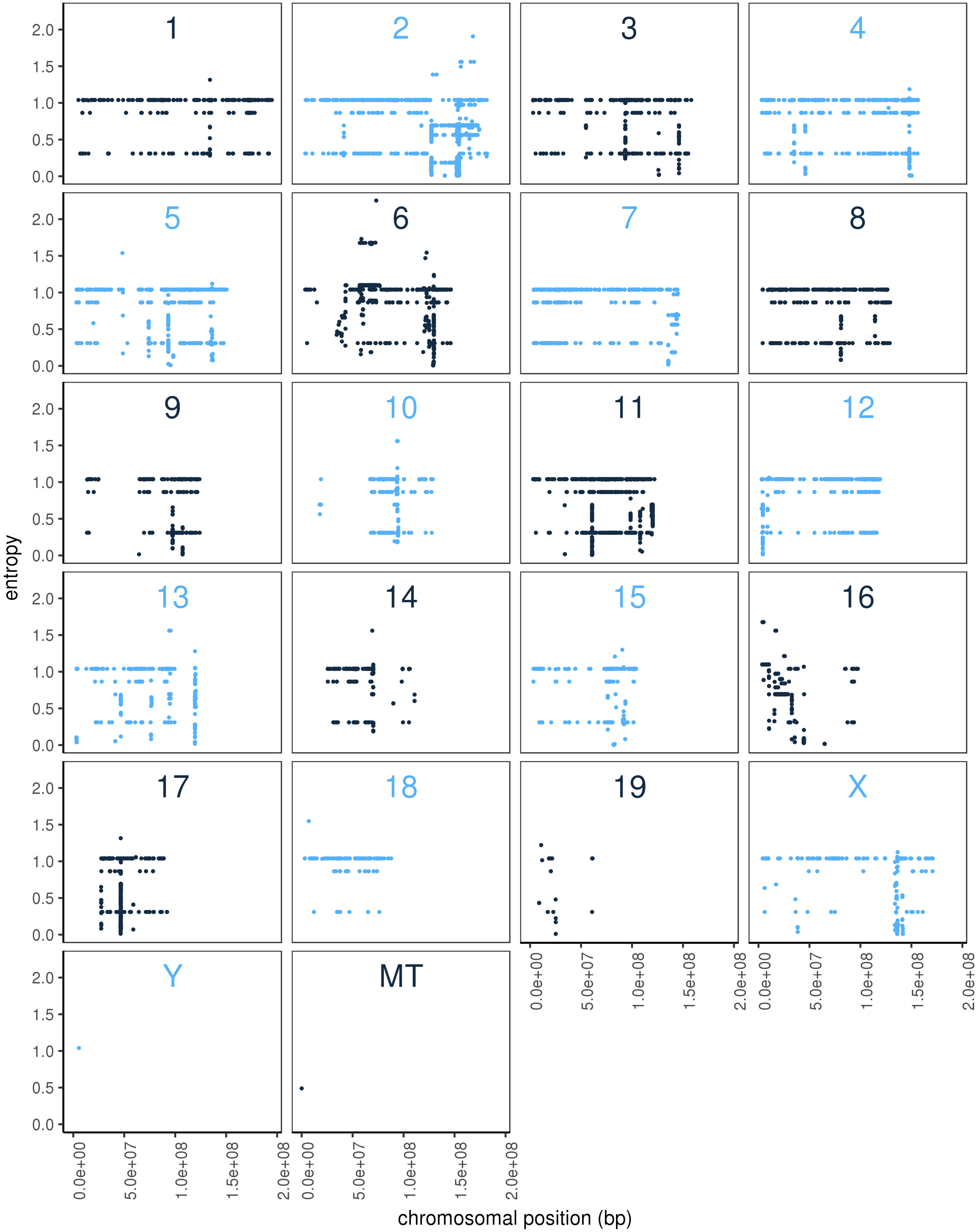

Figure S69 strain CC038, non-zero entropies in exons (+/-100 bp) in all chromosomes. Each point corresponds to the entropy of a variant at that position along the chromosome

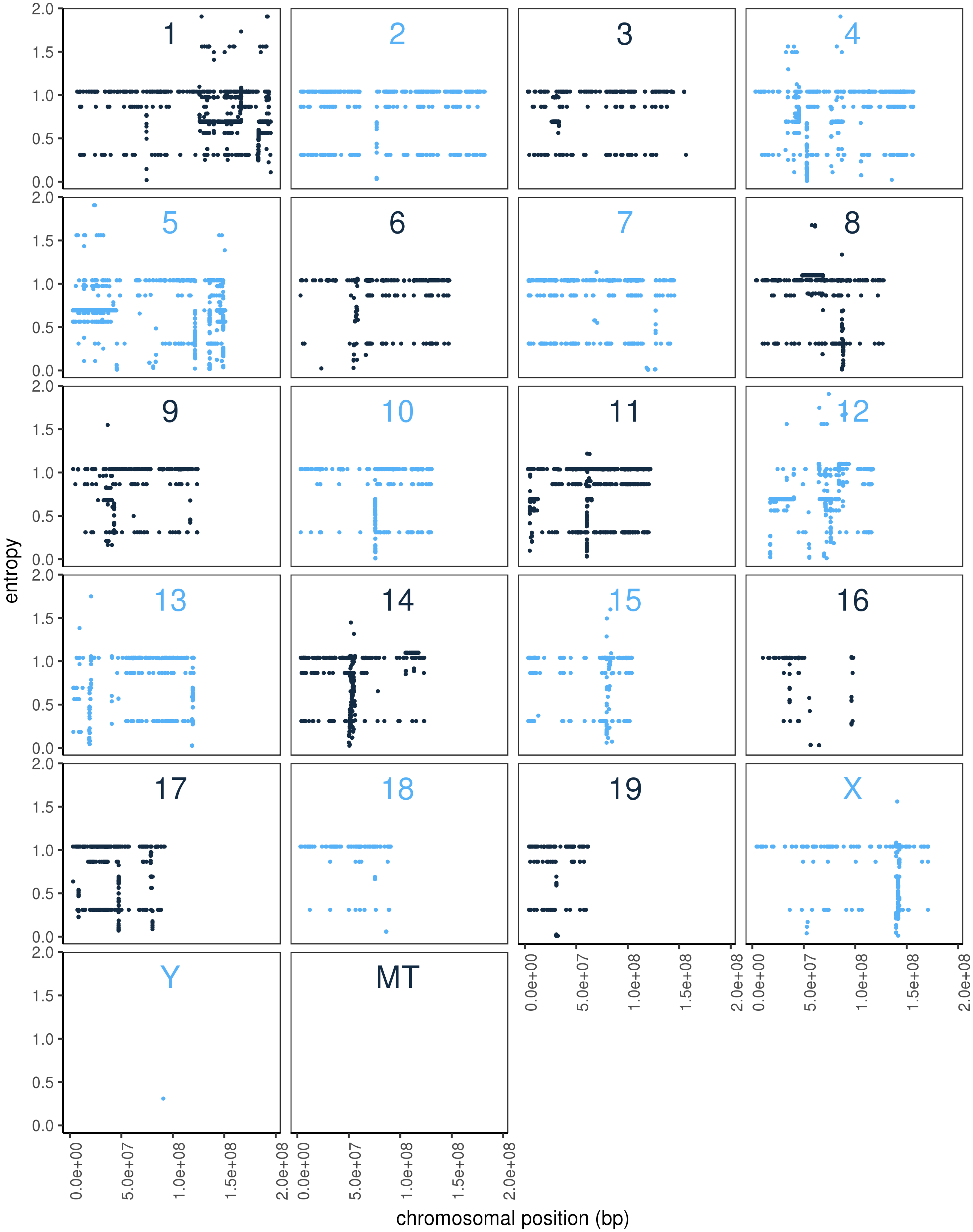

Figure S70 strain CC039, non-zero entropies in exons (+/-100 bp) in all chromosomes. Each point corresponds to the entropy of a variant at that position along the chromosome

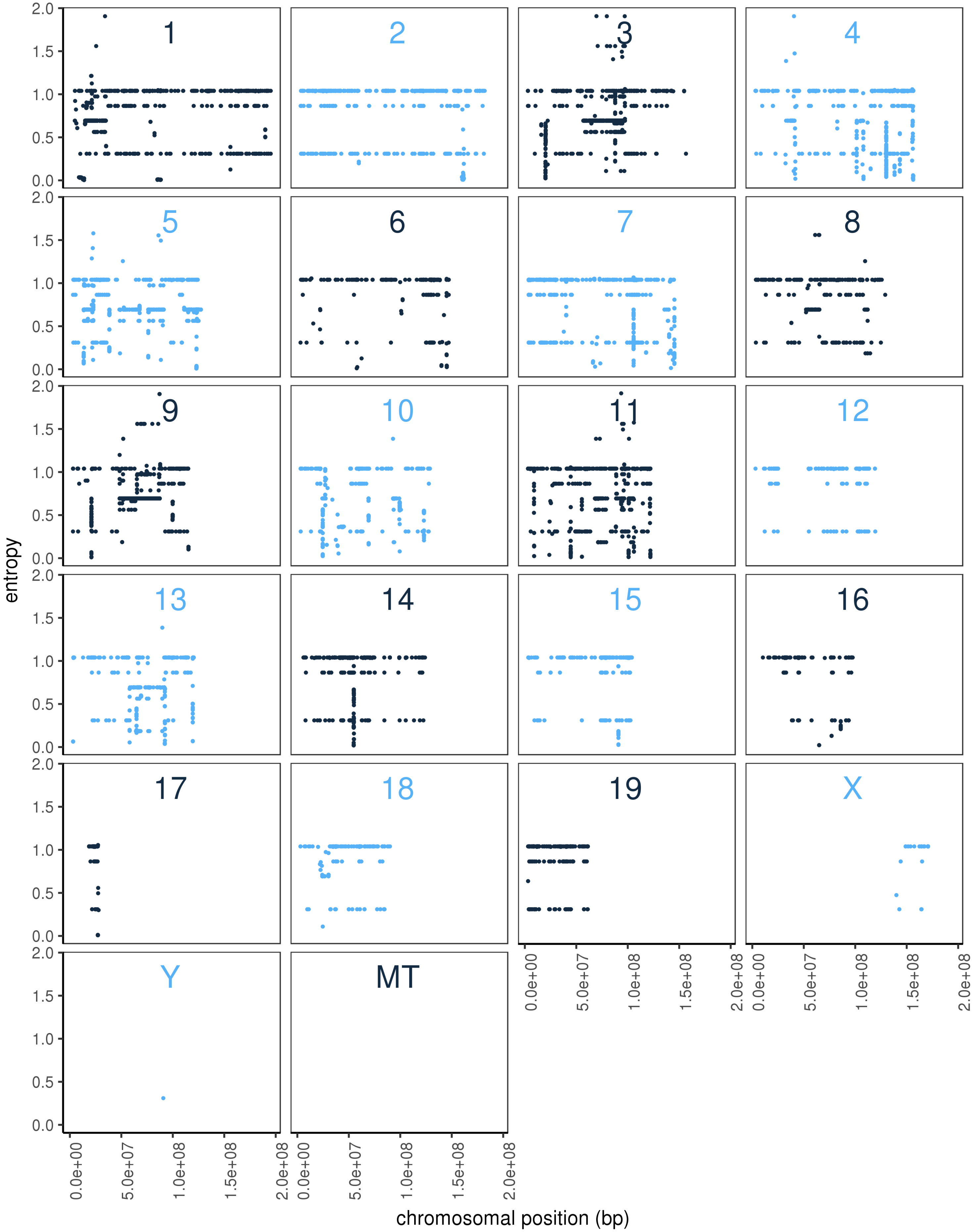

Figure S71 strain CC040, non-zero entropies in exons (+/-100 bp) in all chromosomes. Each point corresponds to the entropy of a variant at that position along the chromosome

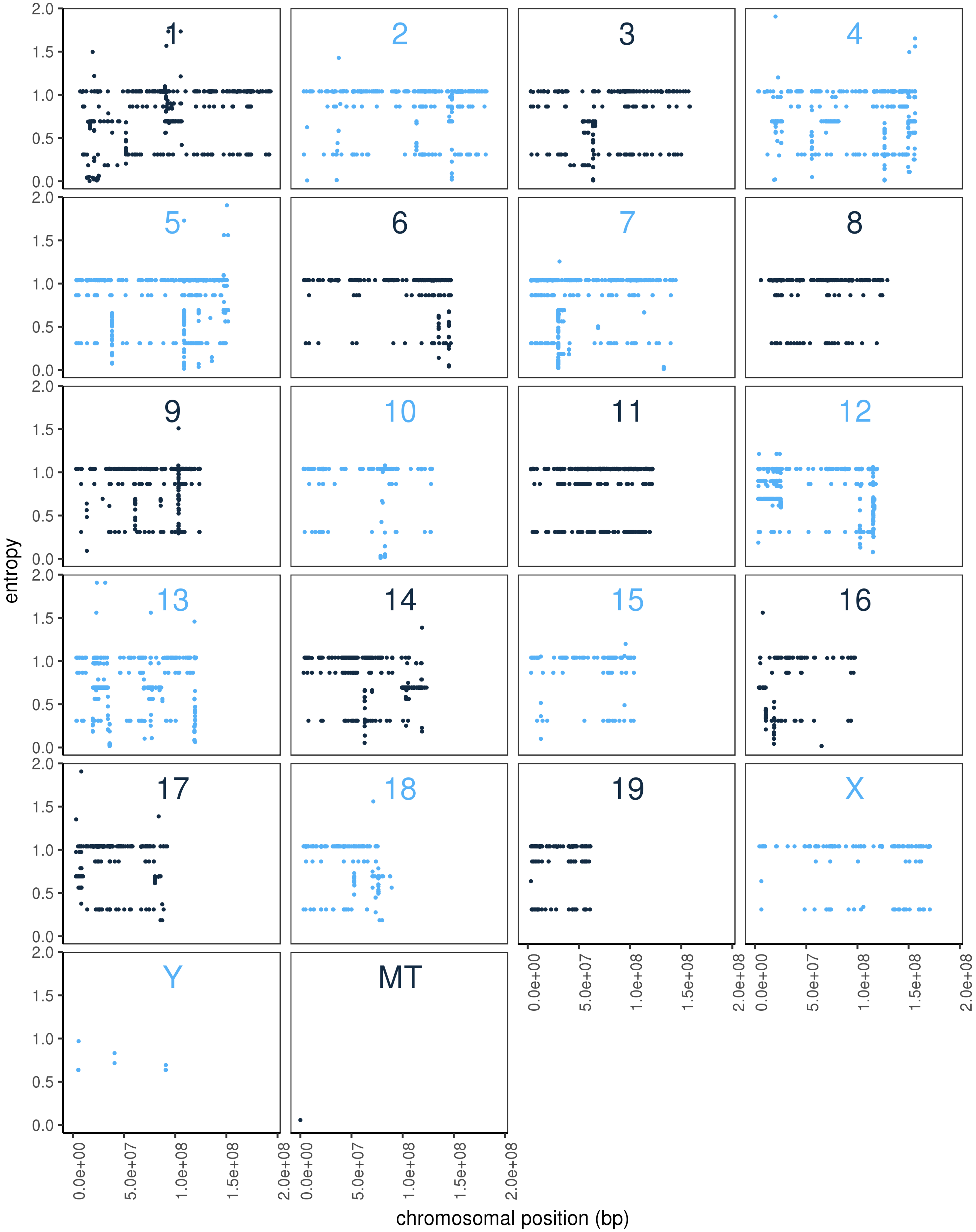

Figure S72 strain CC041, non-zero entropies in exons (+/-100 bp) in all chromosomes. Each point corresponds to the entropy of a variant at that position along the chromosome

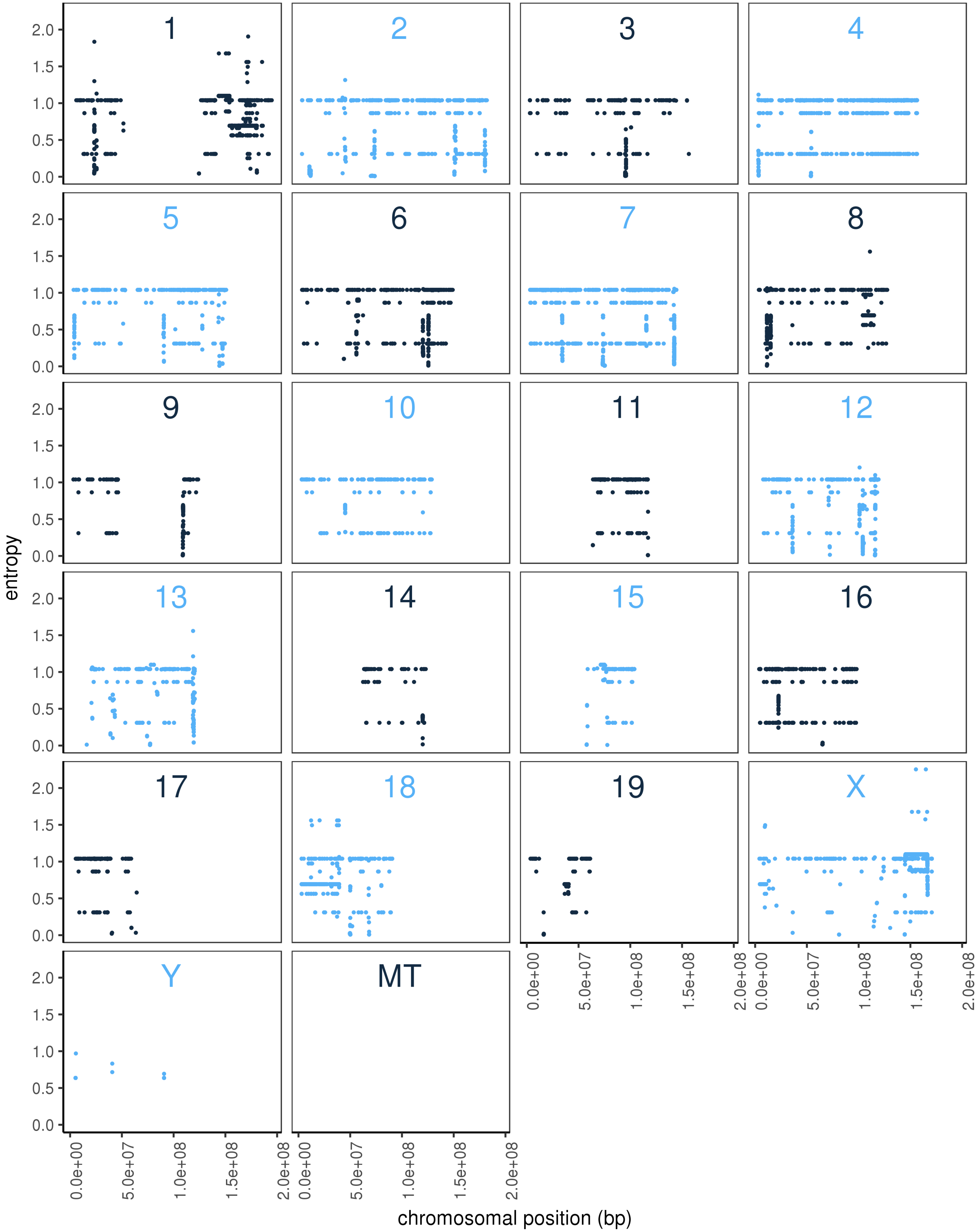

Figure S73 strain CC042, non-zero entropies in exons (+/-100 bp) in all chromosomes. Each point corresponds to the entropy of a variant at that position along the chromosome

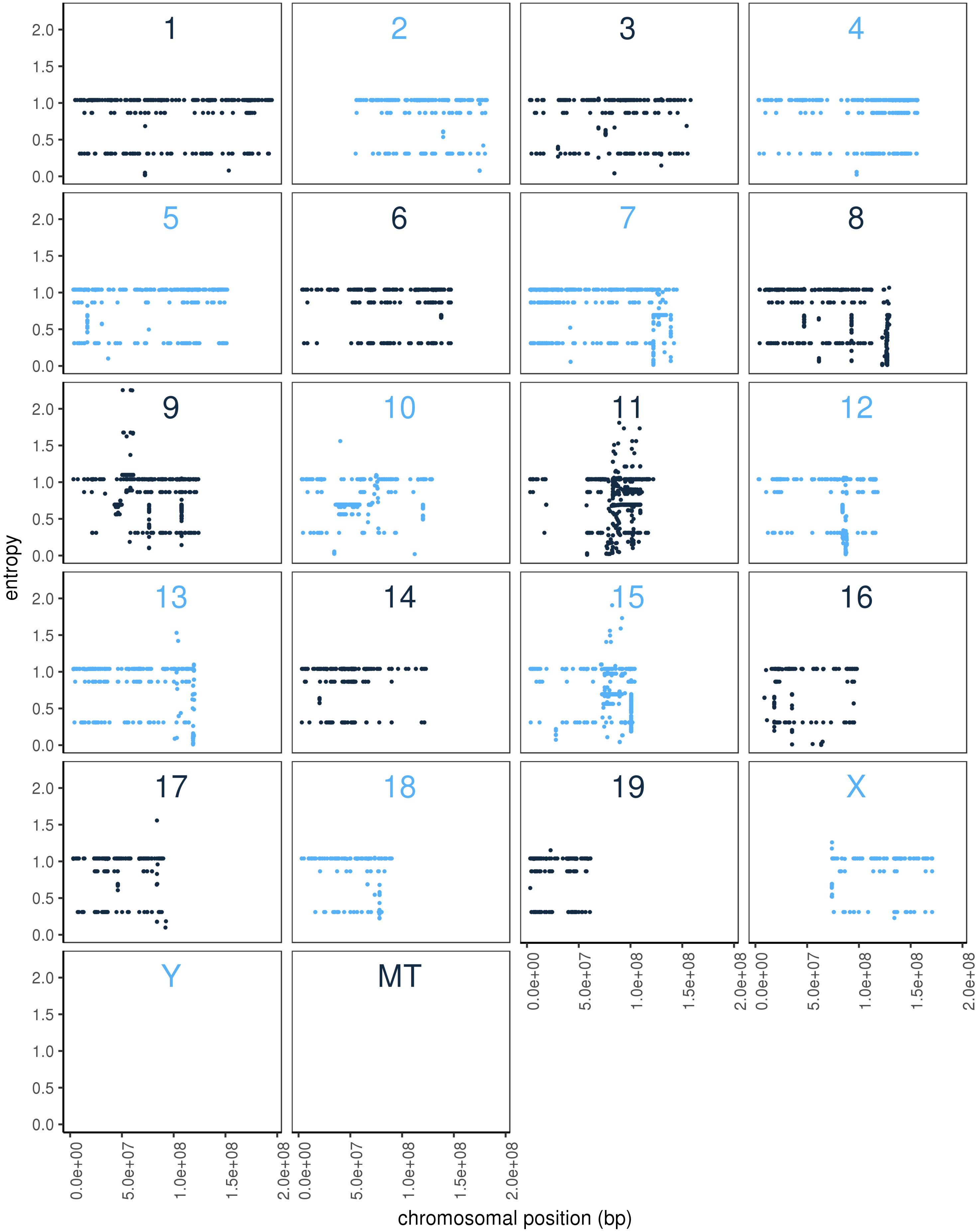

Figure S74 strain CC043, non-zero entropies in exons (+/-100 bp) in all chromosomes. Each point corresponds to the entropy of a variant at that position along the chromosome

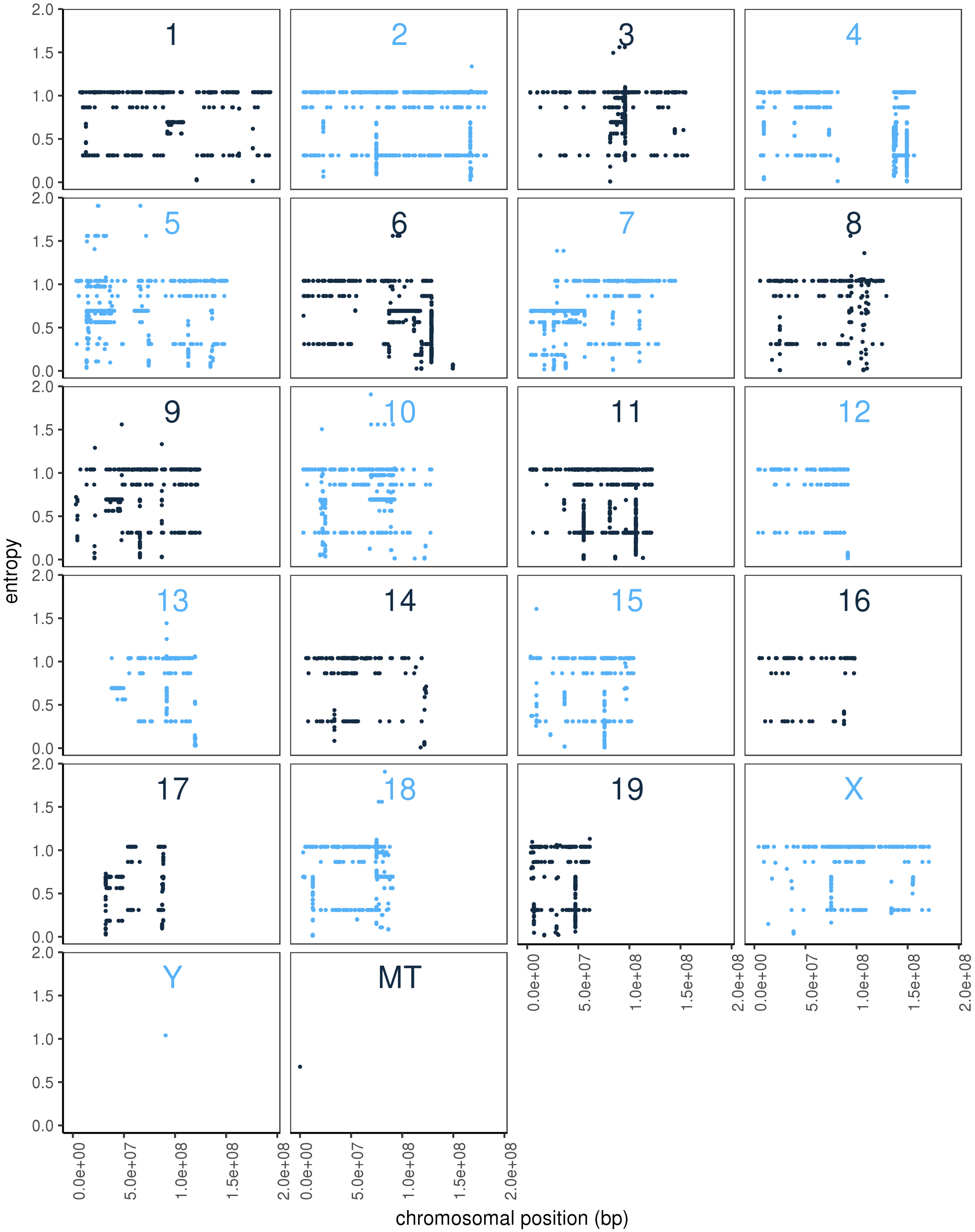

Figure S75 strain CC044, non-zero entropies in exons (+/-100 bp) in all chromosomes. Each point corresponds to the entropy of a variant at that position along the chromosome

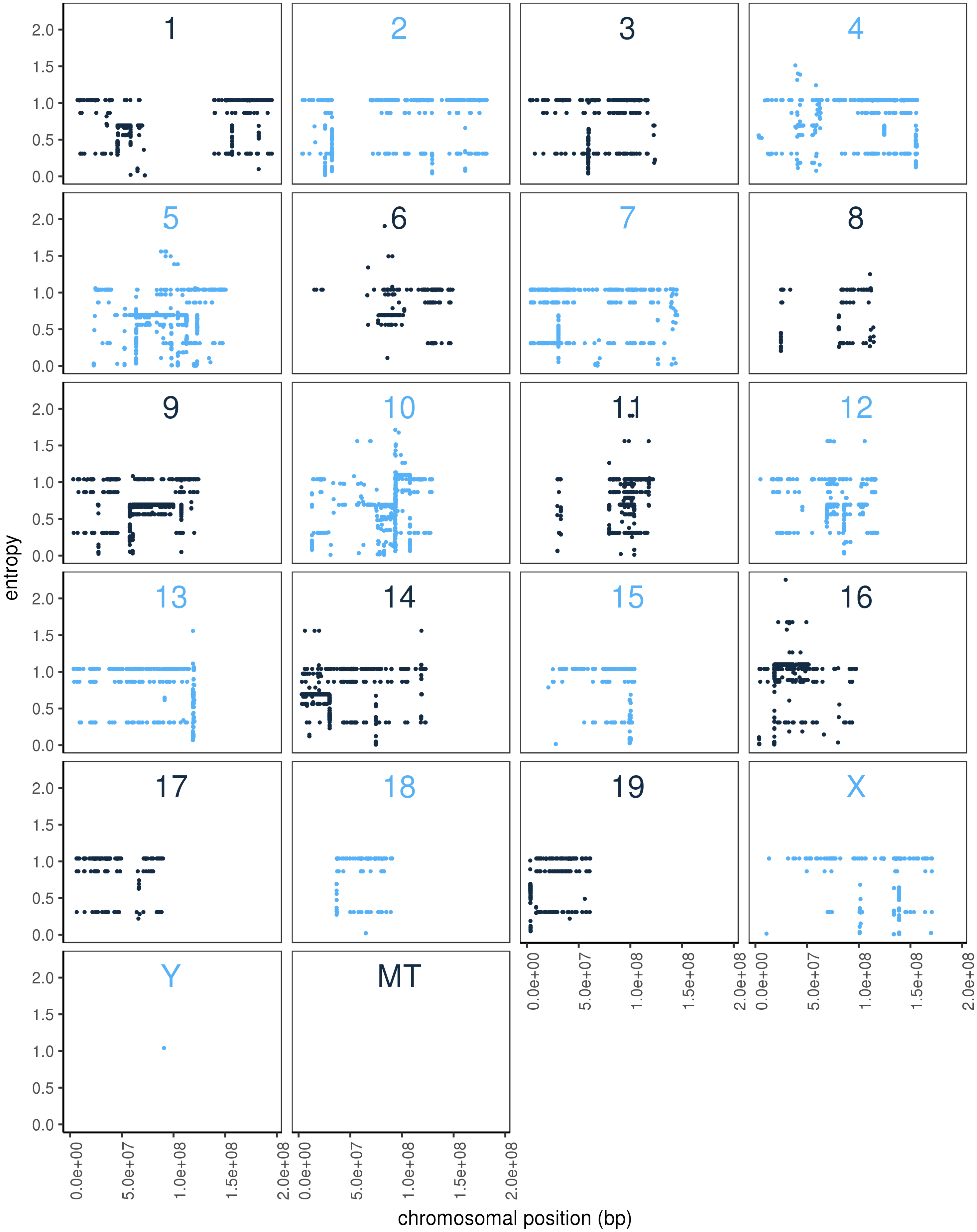

Figure S76 strain CC045, non-zero entropies in exons (+/-100 bp) in all chromosomes. Each point corresponds to the entropy of a variant at that position along the chromosome

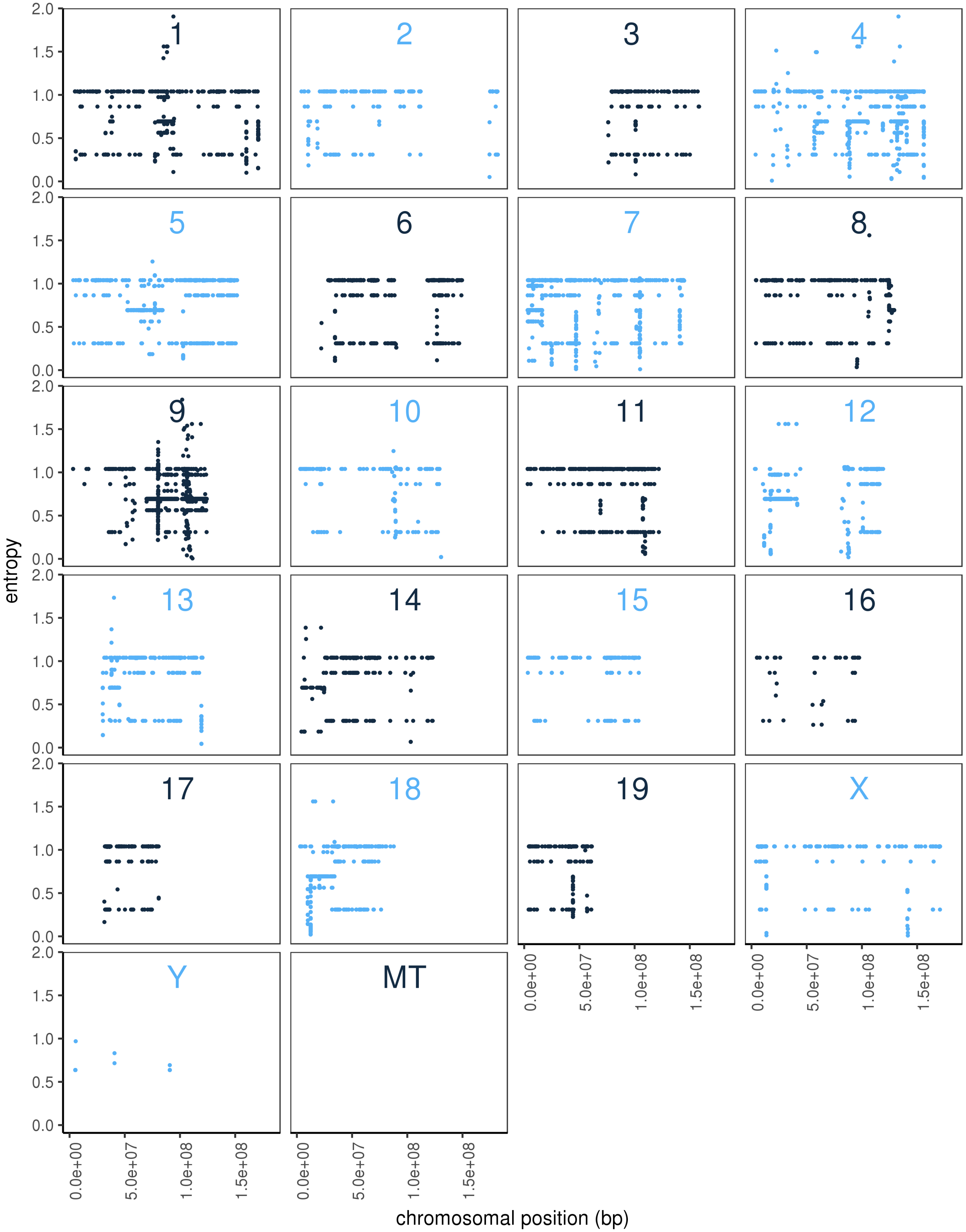

Figure S77 strain CC046, non-zero entropies in exons (+/-100 bp) in all chromosomes. Each point corresponds to the entropy of a variant at that position along the chromosome

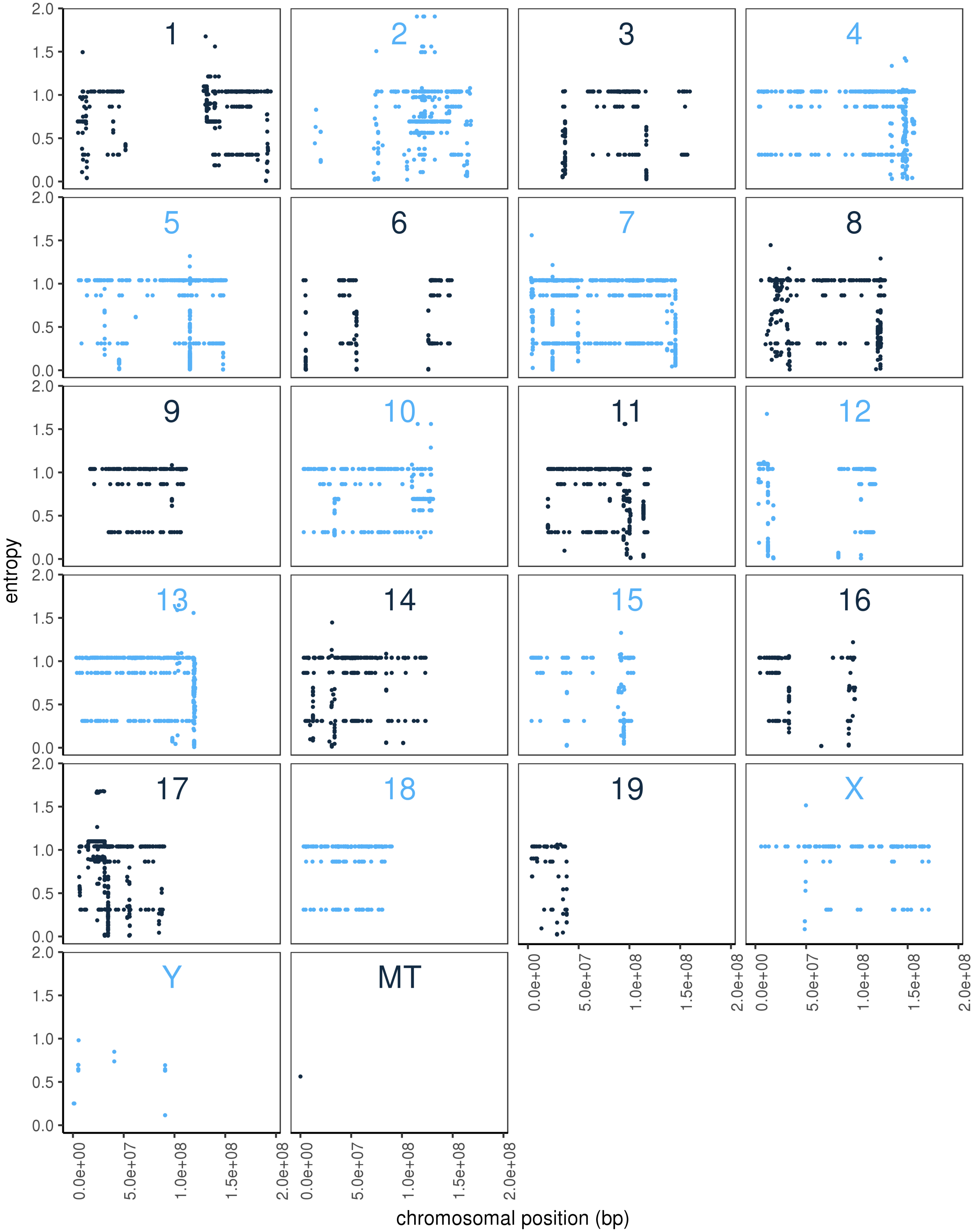

Figure S78 strain CC047, non-zero entropies in exons (+/-100 bp) in all chromosomes. Each point corresponds to the entropy of a variant at that position along the chromosome

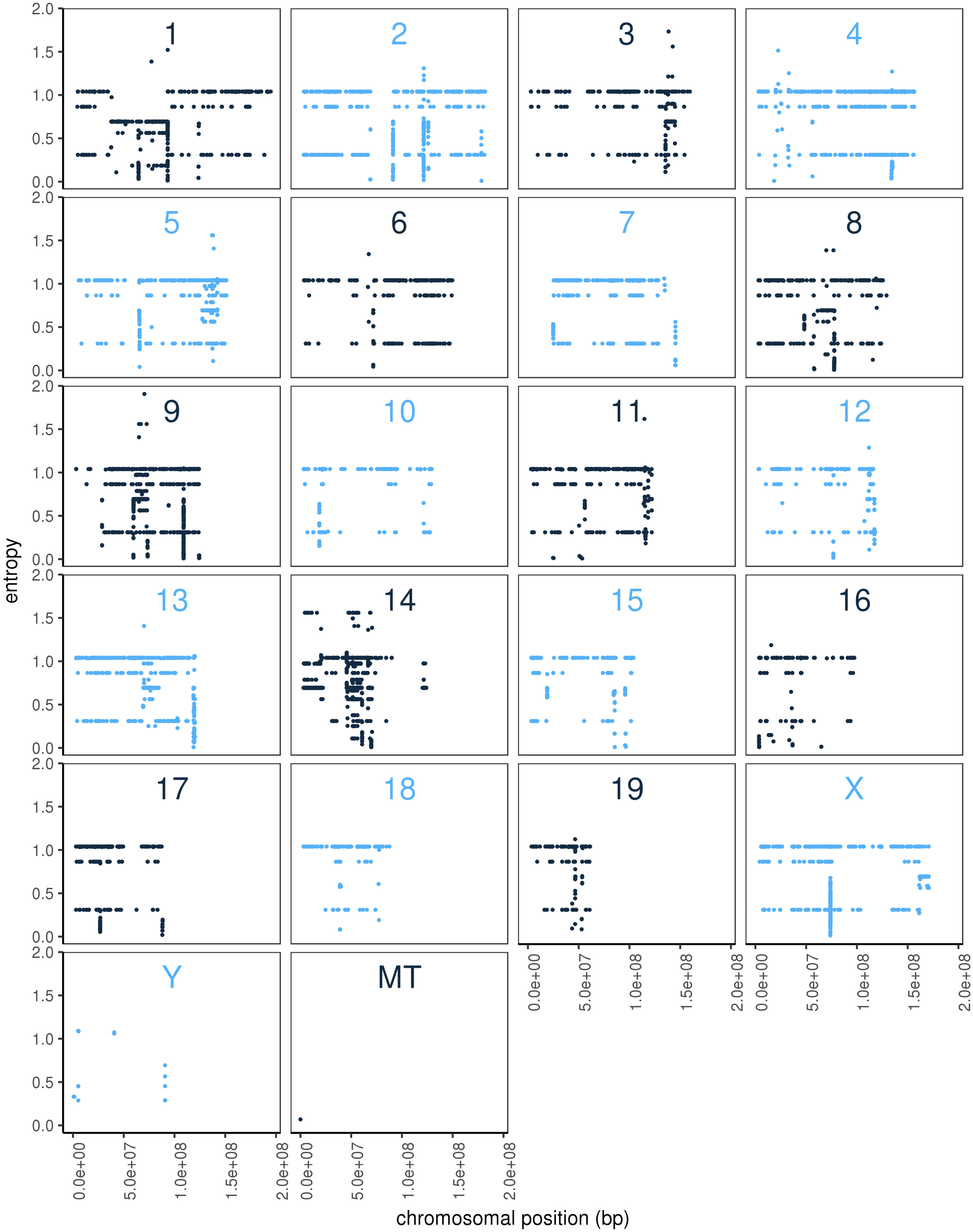

Figure S79 strain CC048, non-zero entropies in exons (+/-100 bp) in all chromosomes. Each point corresponds to the entropy of a variant at that position along the chromosome

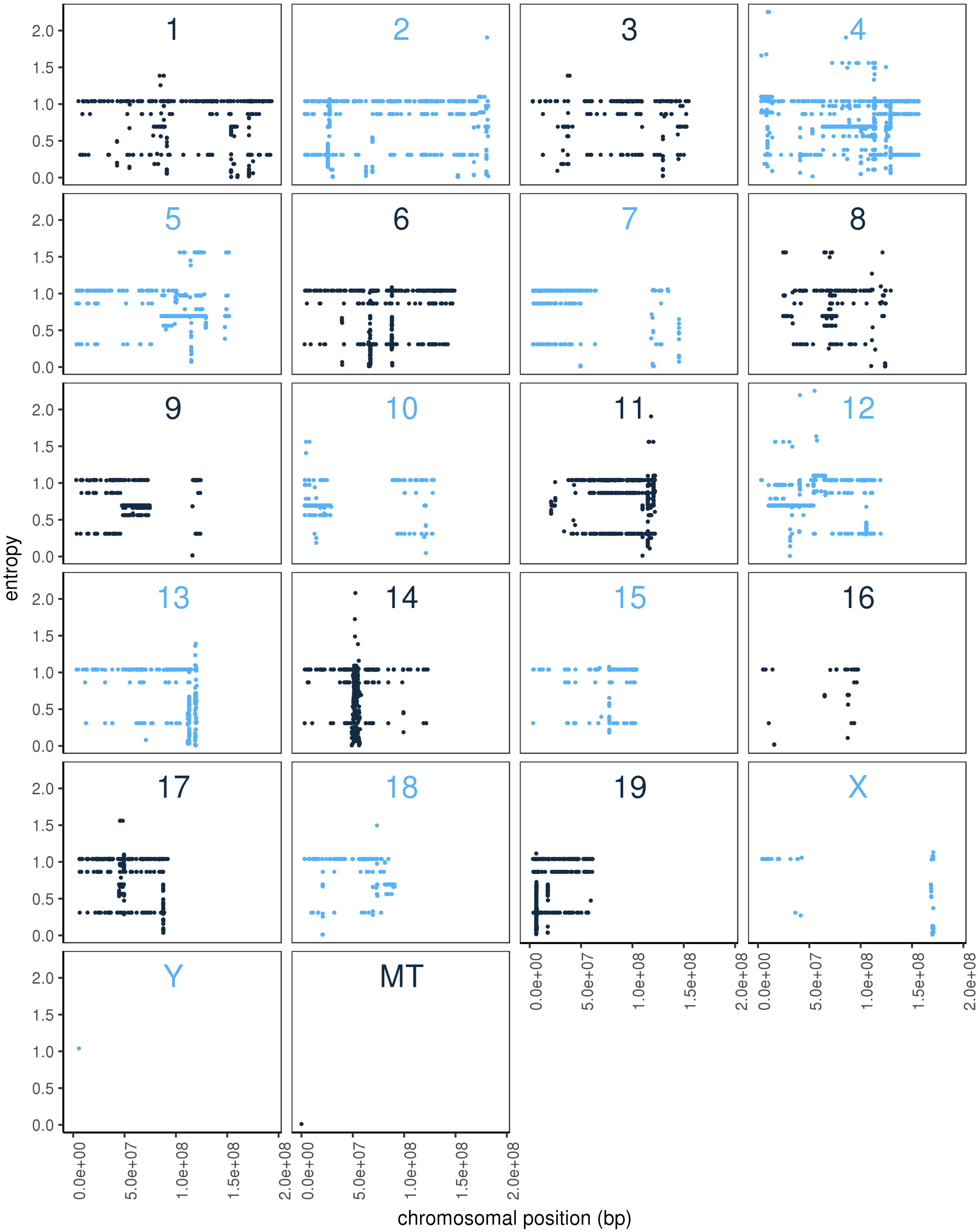

Figure S80 strain CC049, non-zero entropies in exons (+/-100 bp) in all chromosomes. Each point corresponds to the entropy of a variant at that position along the chromosome

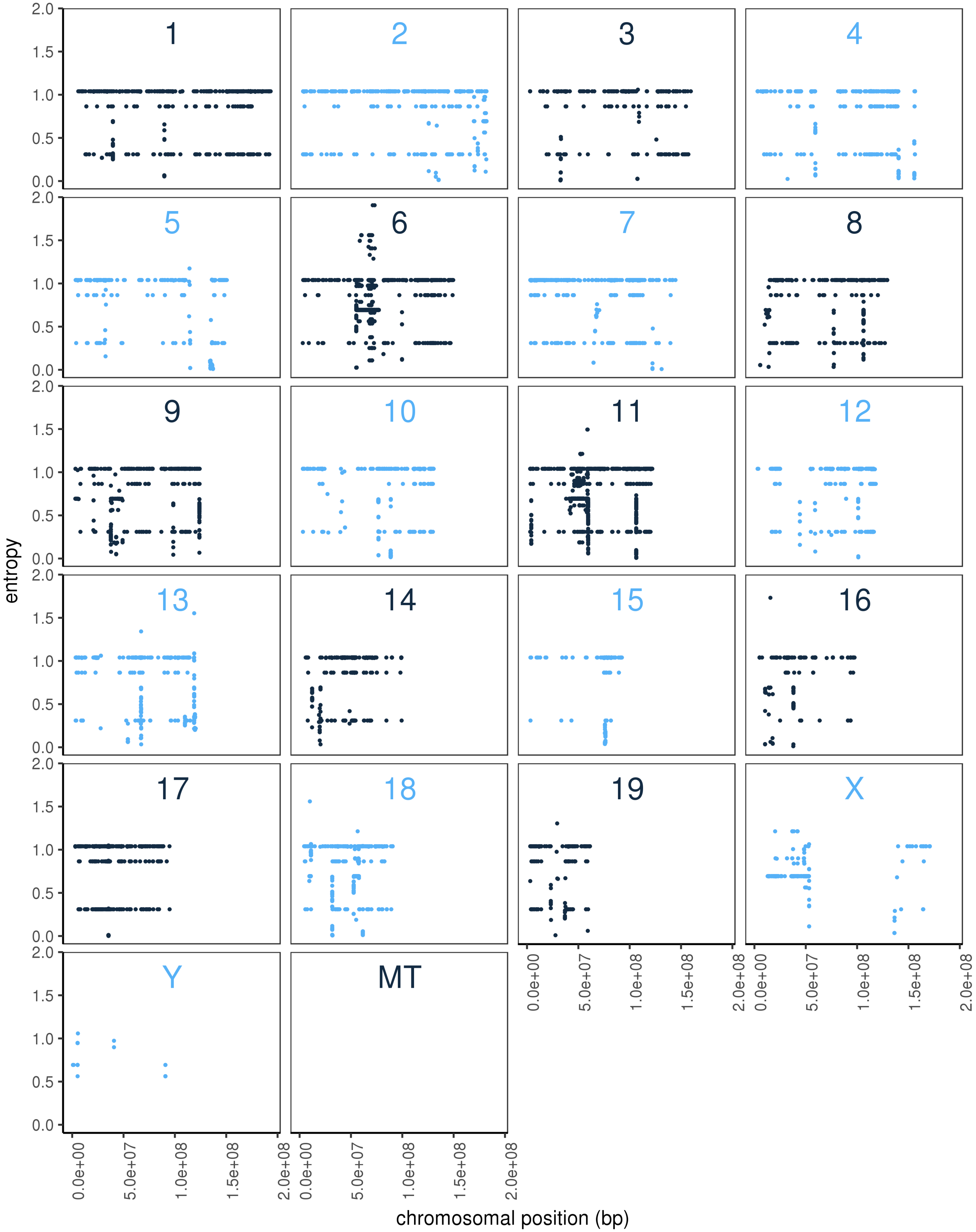

Figure S81 strain CC050, non-zero entropies in exons (+/-100 bp) in all chromosomes. Each point corresponds to the entropy of a variant at that position along the chromosome

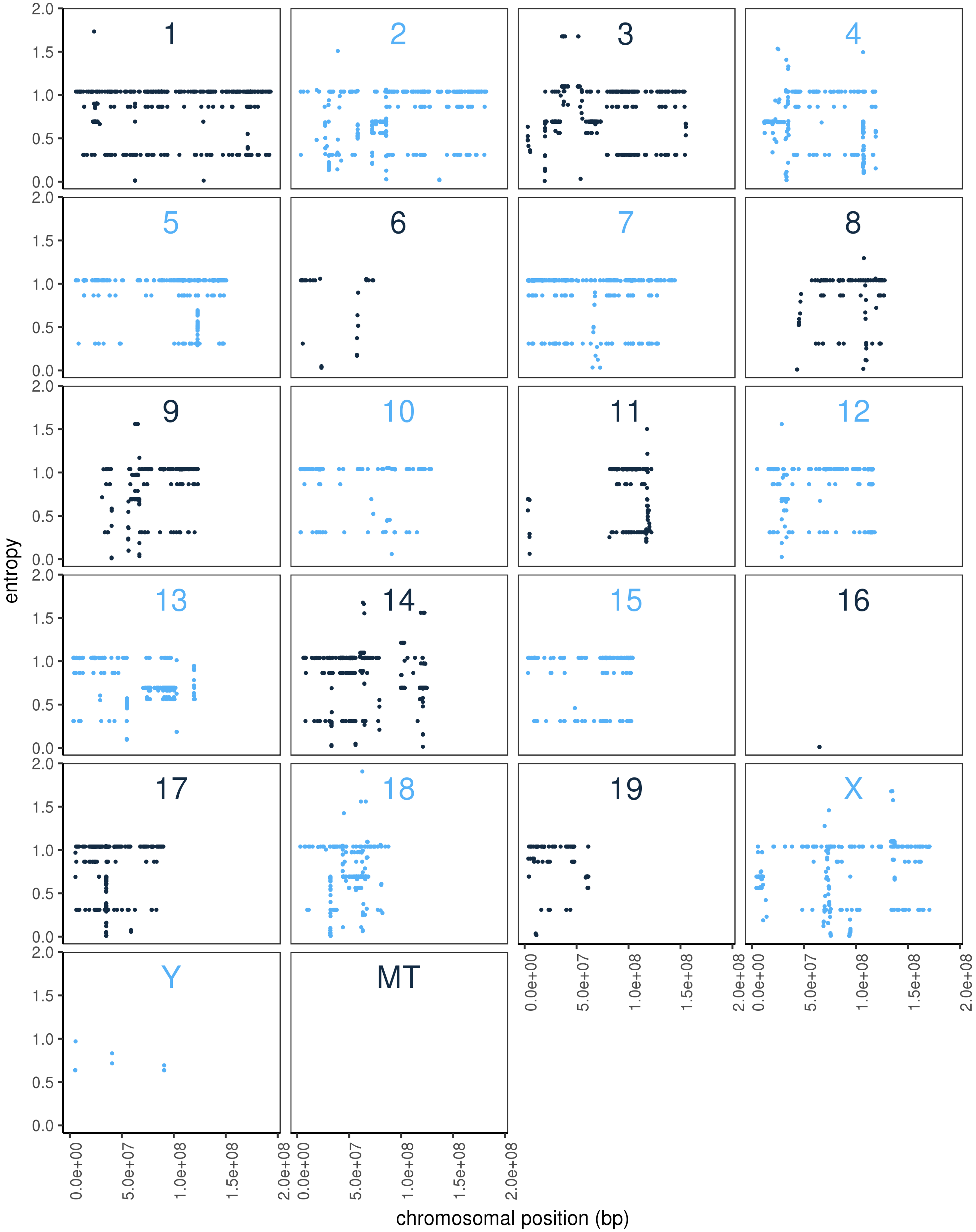

Figure S82 strain CC051, non-zero entropies in exons (+/-100 bp) in all chromosomes. Each point corresponds to the entropy of a variant at that position along the chromosome

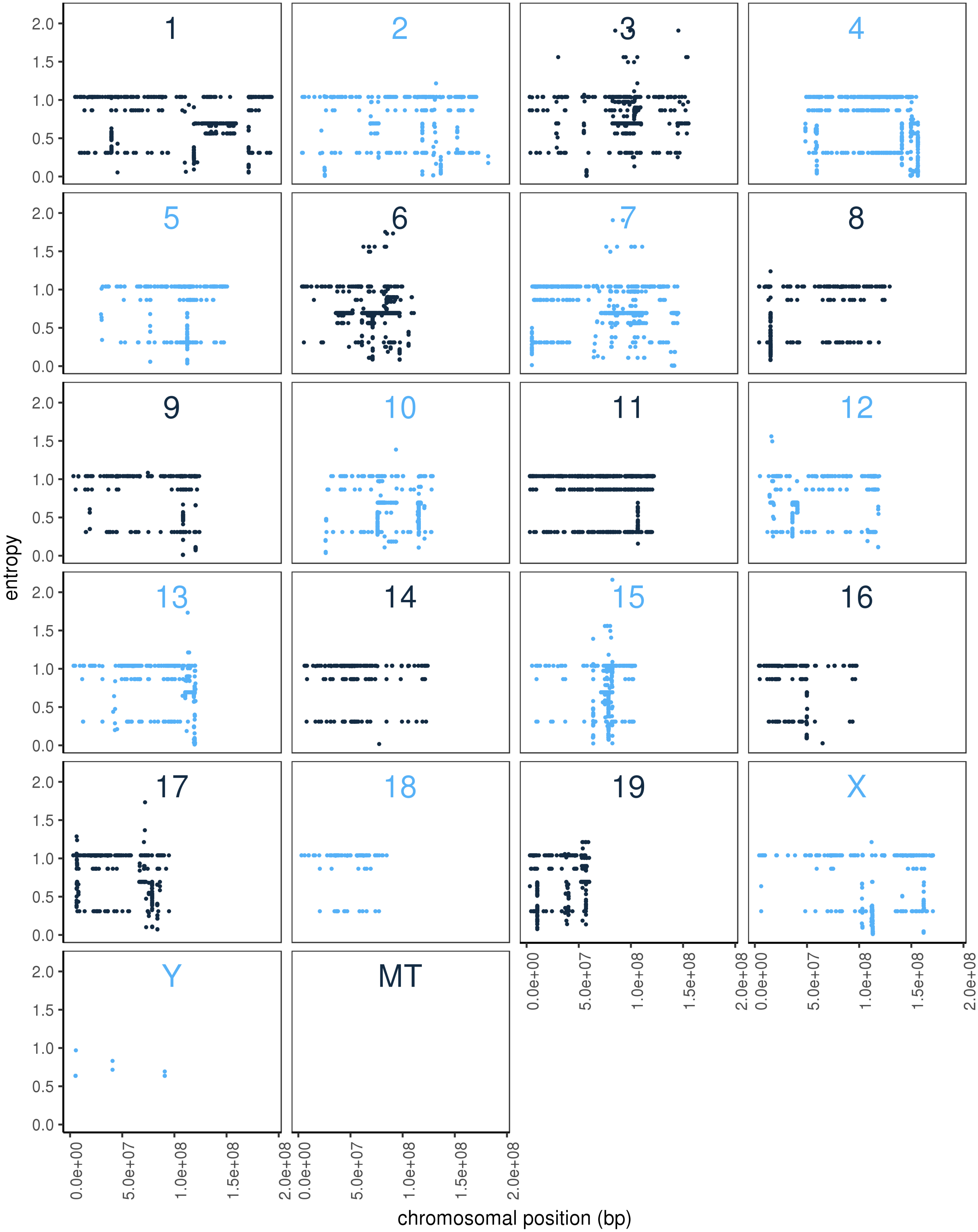

Figure S83 strain CC052, non-zero entropies in exons (+/-100 bp) in all chromosomes. Each point corresponds to the entropy of a variant at that position along the chromosome

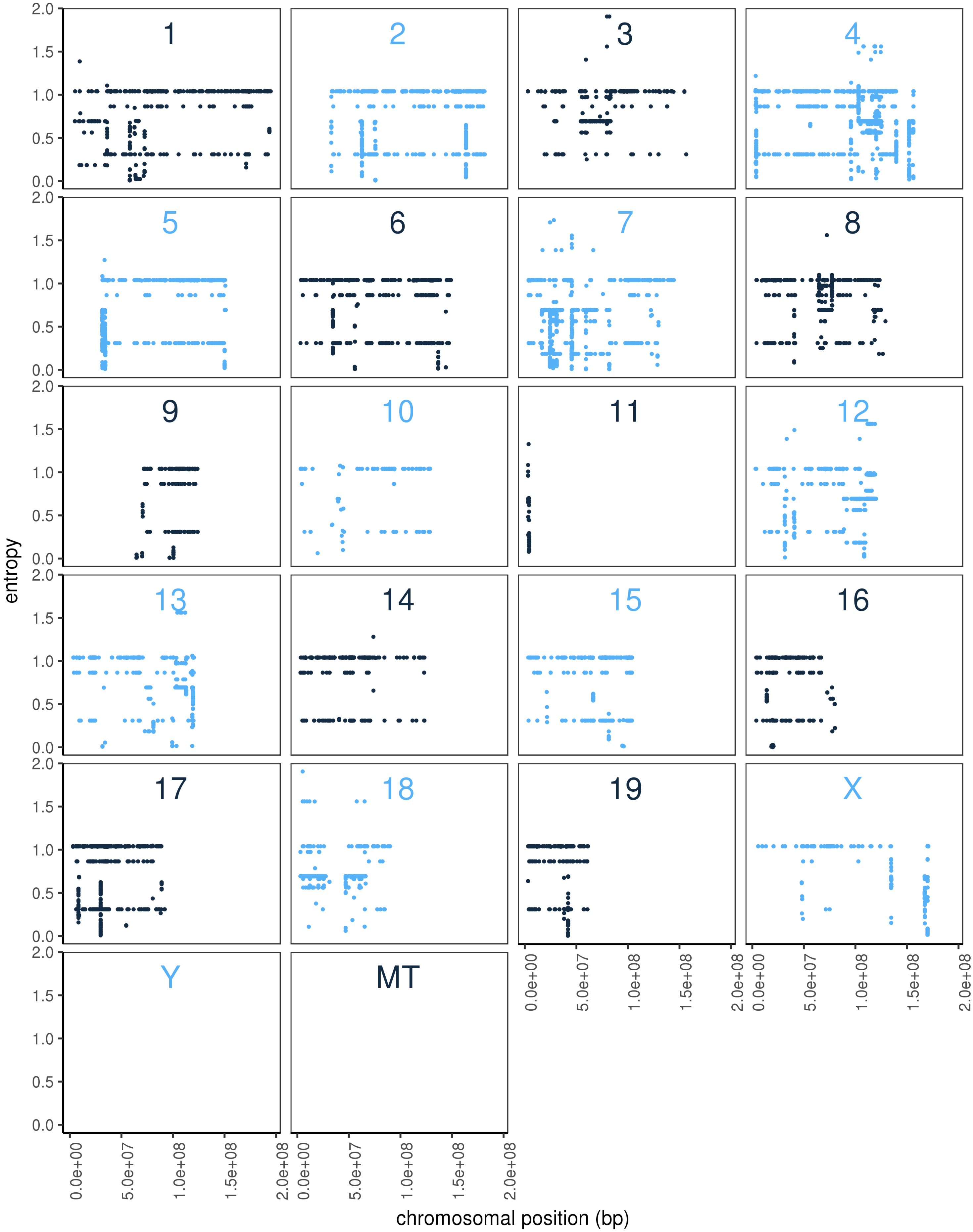

Figure S84 strain CC053, non-zero entropies in exons (+/-100 bp) in all chromosomes. Each point corresponds to the entropy of a variant at that position along the chromosome

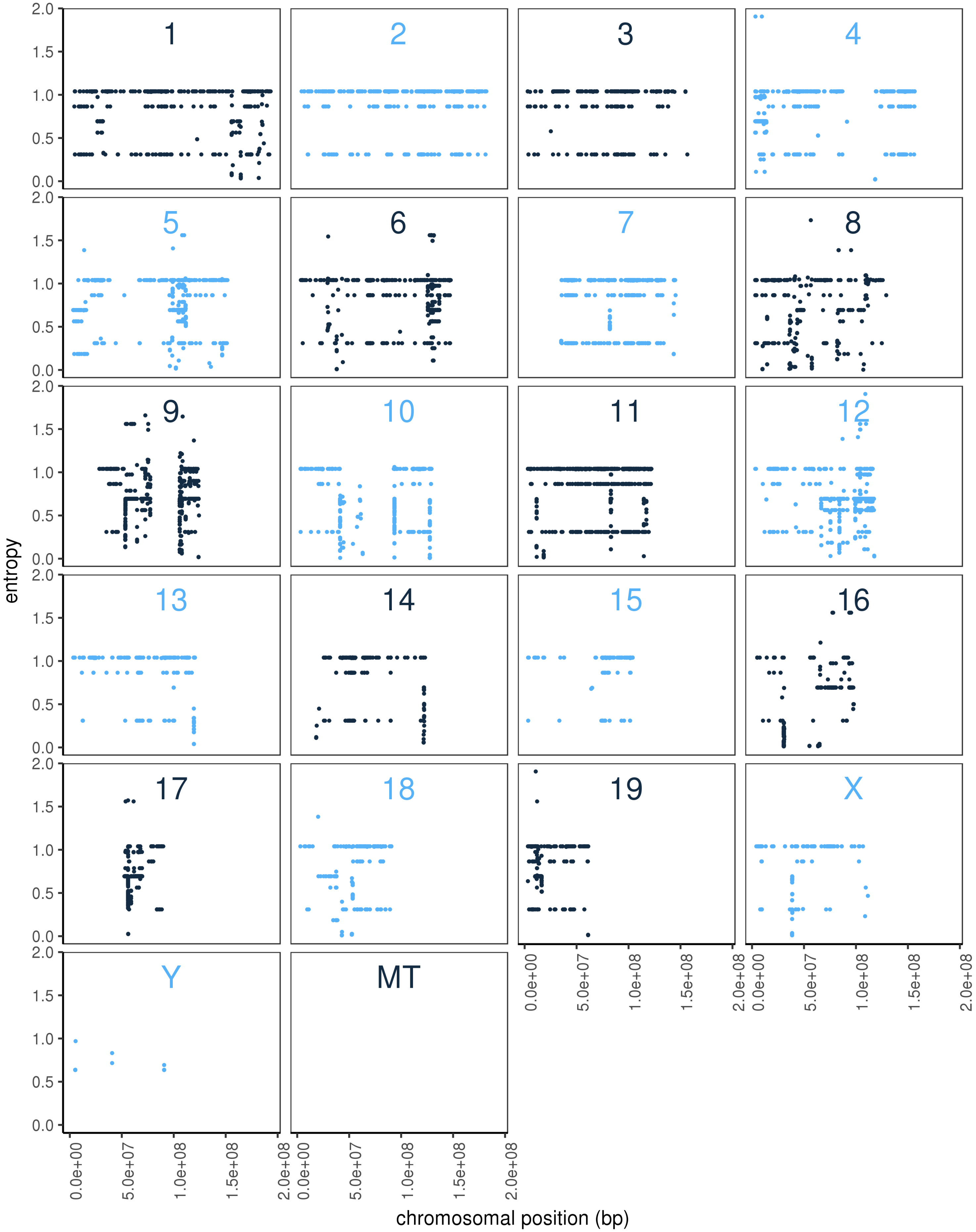

Figure S85 strain CC054, non-zero entropies in exons (+/-100 bp) in all chromosomes. Each point corresponds to the entropy of a variant at that position along the chromosome

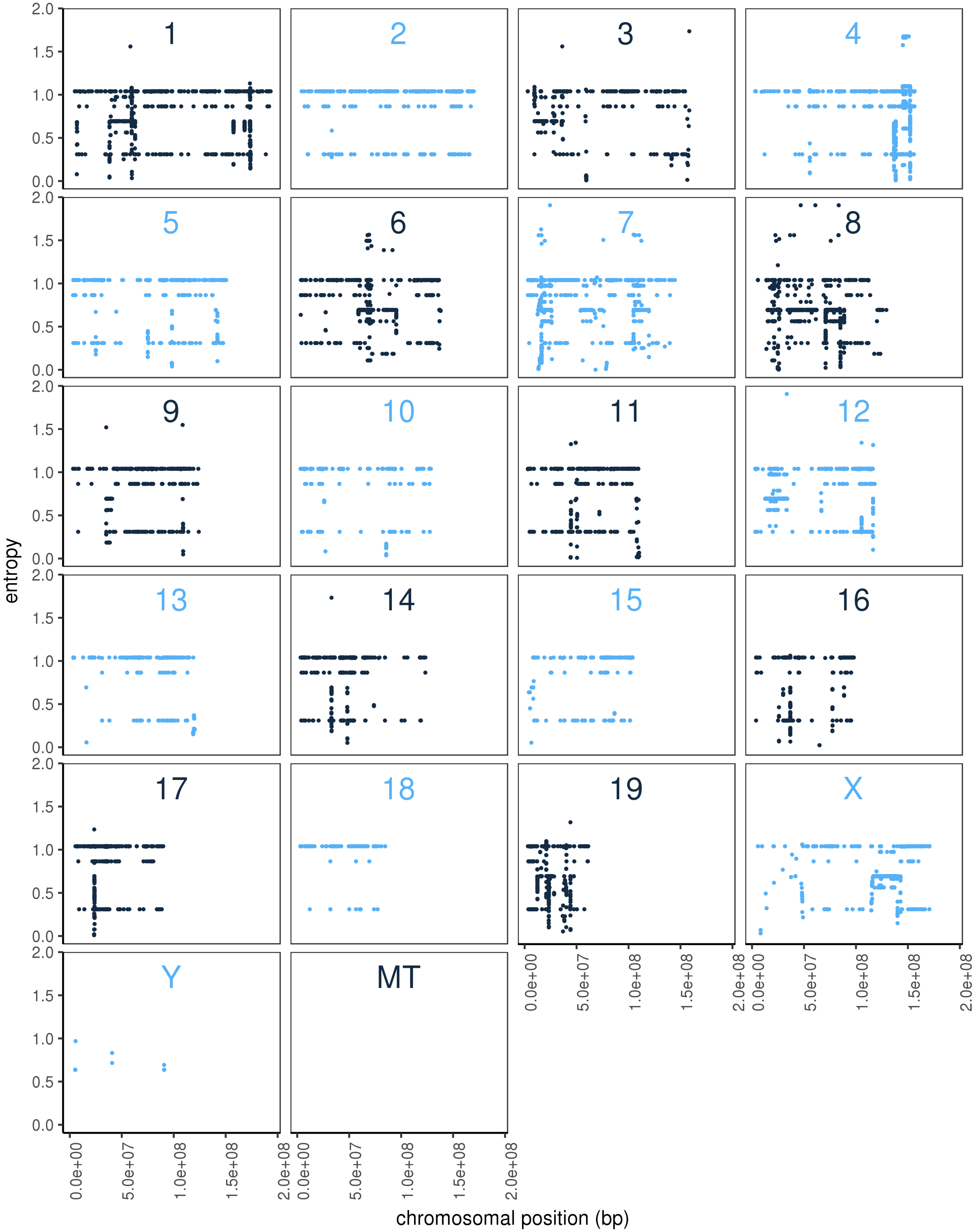

Figure S86 strain CC055, non-zero entropies in exons (+/-100 bp) in all chromosomes. Each point corresponds to the entropy of a variant at that position along the chromosome

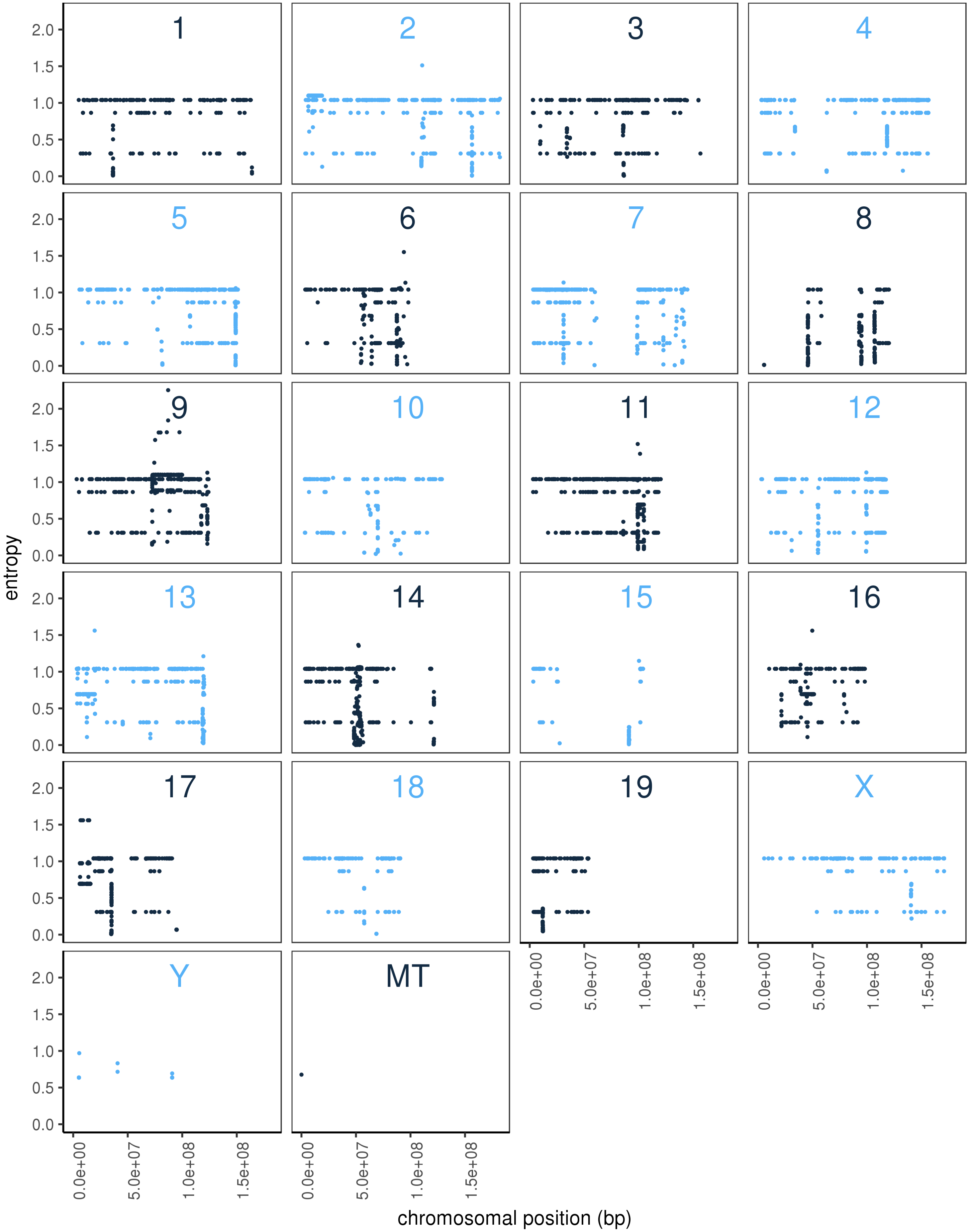

Figure S87 strain CC056, non-zero entropies in exons (+/-100 bp) in all chromosomes. Each point corresponds to the entropy of a variant at that position along the chromosome

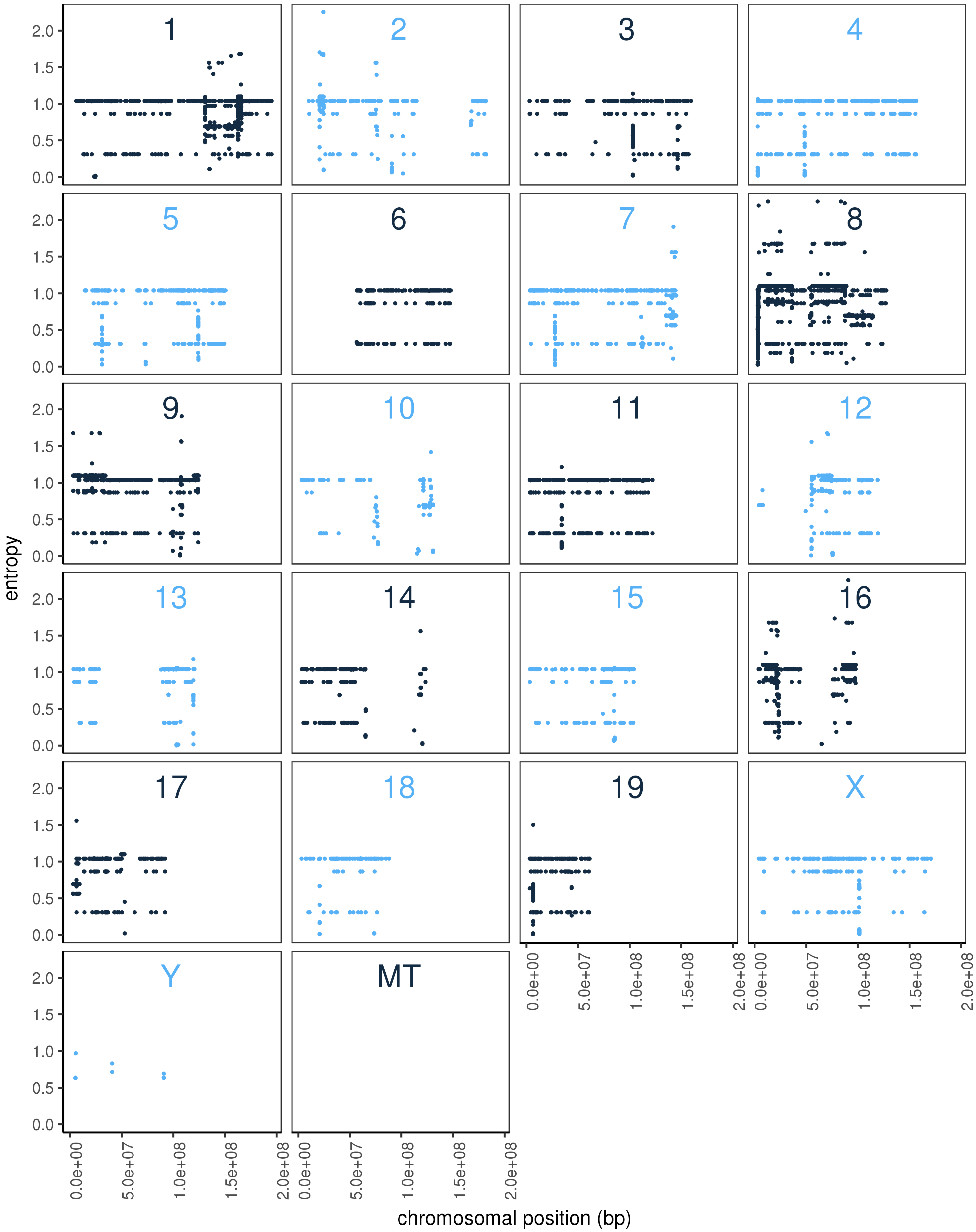

Figure S88 strain CC057, non-zero entropies in exons (+/-100 bp) in all chromosomes. Each point corresponds to the entropy of a variant at that position along the chromosome

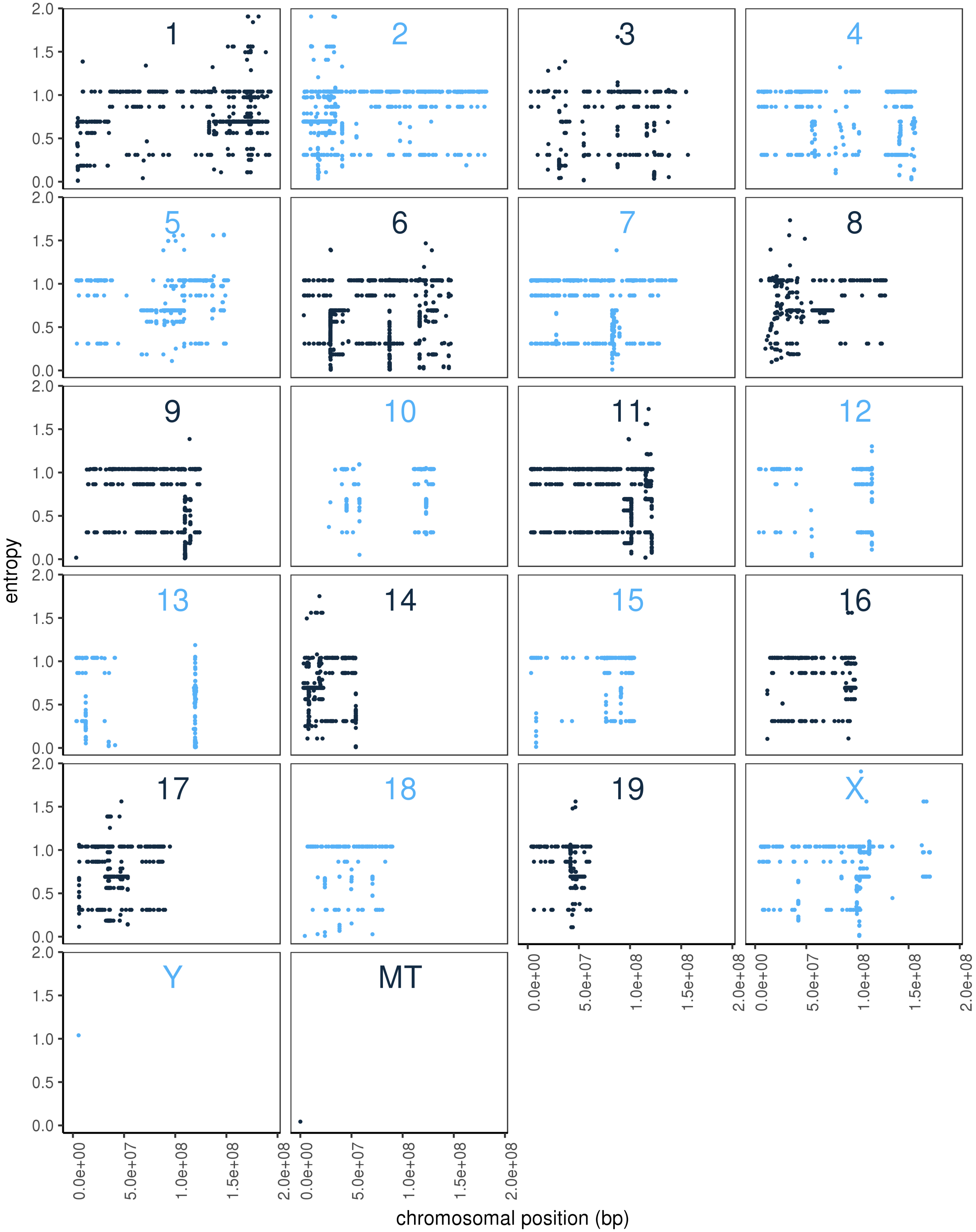

Figure S89 strain CC058, non-zero entropies in exons (+/-100 bp) in all chromosomes. Each point corresponds to the entropy of a variant at that position along the chromosome

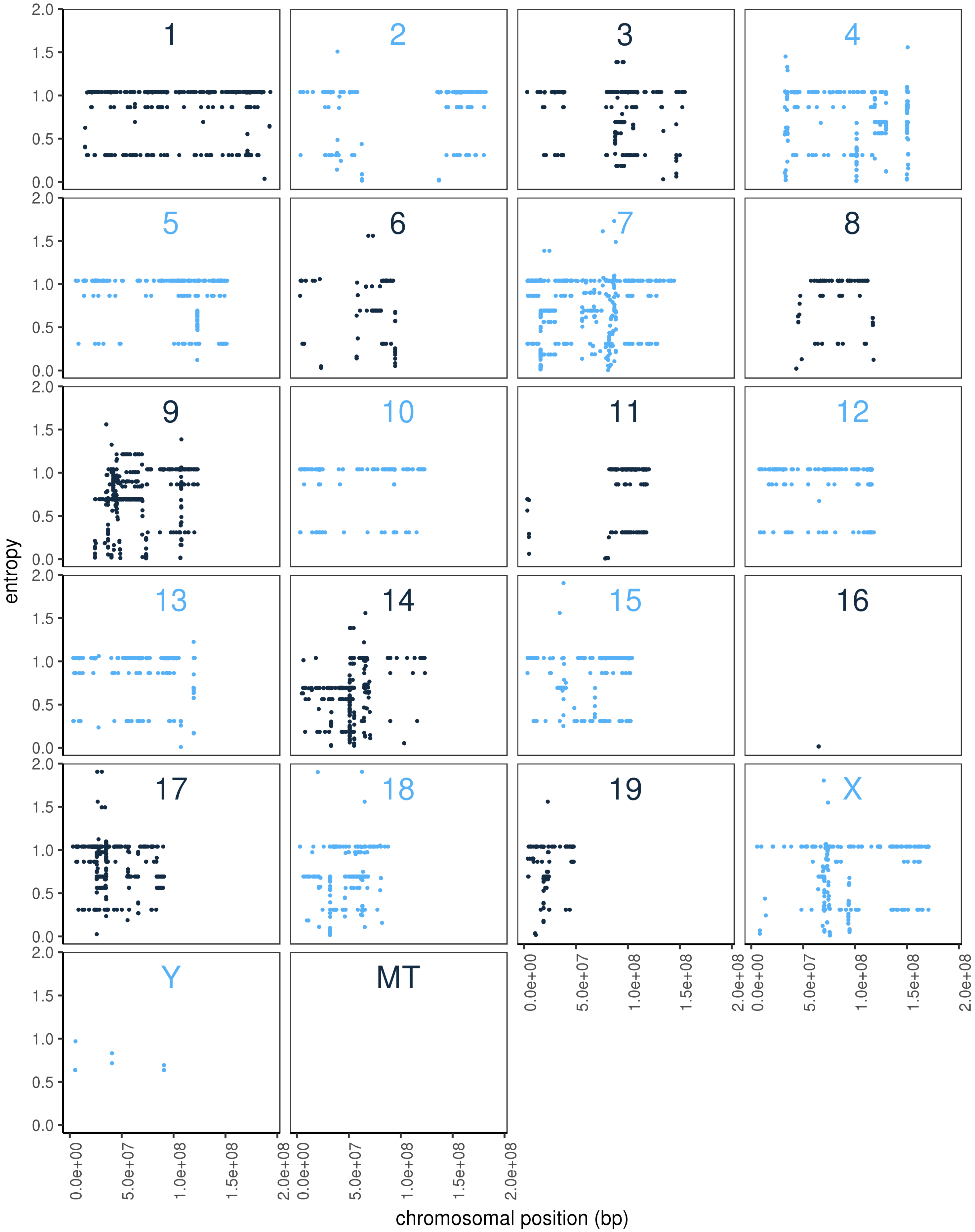

Figure S90 strain CC059, non-zero entropies in exons (+/-100 bp) in all chromosomes. Each point corresponds to the entropy of a variant at that position along the chromosome

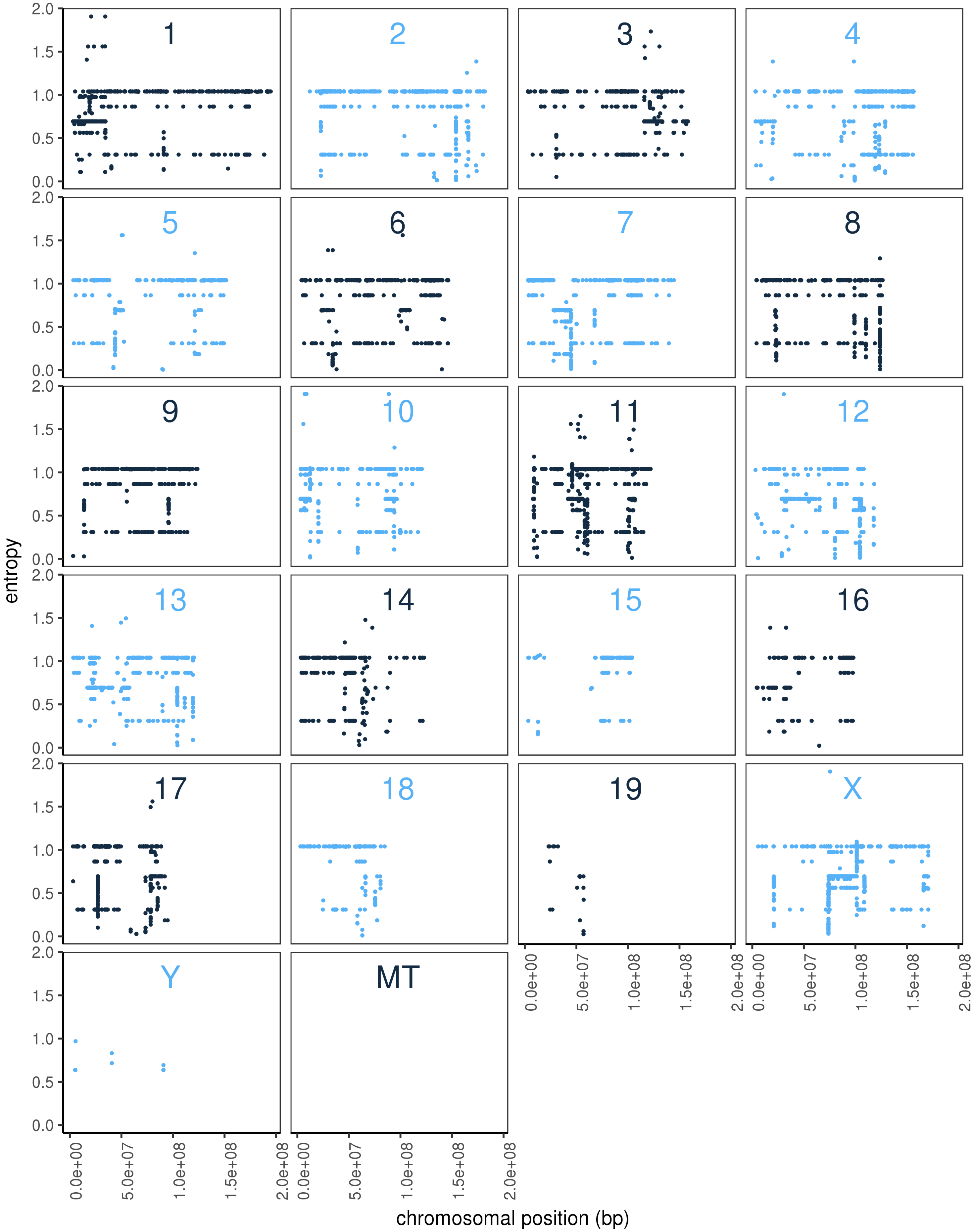

Figure S91 strain CC060, non-zero entropies in exons (+/-100 bp) in all chromosomes. Each point corresponds to the entropy of a variant at that position along the chromosome

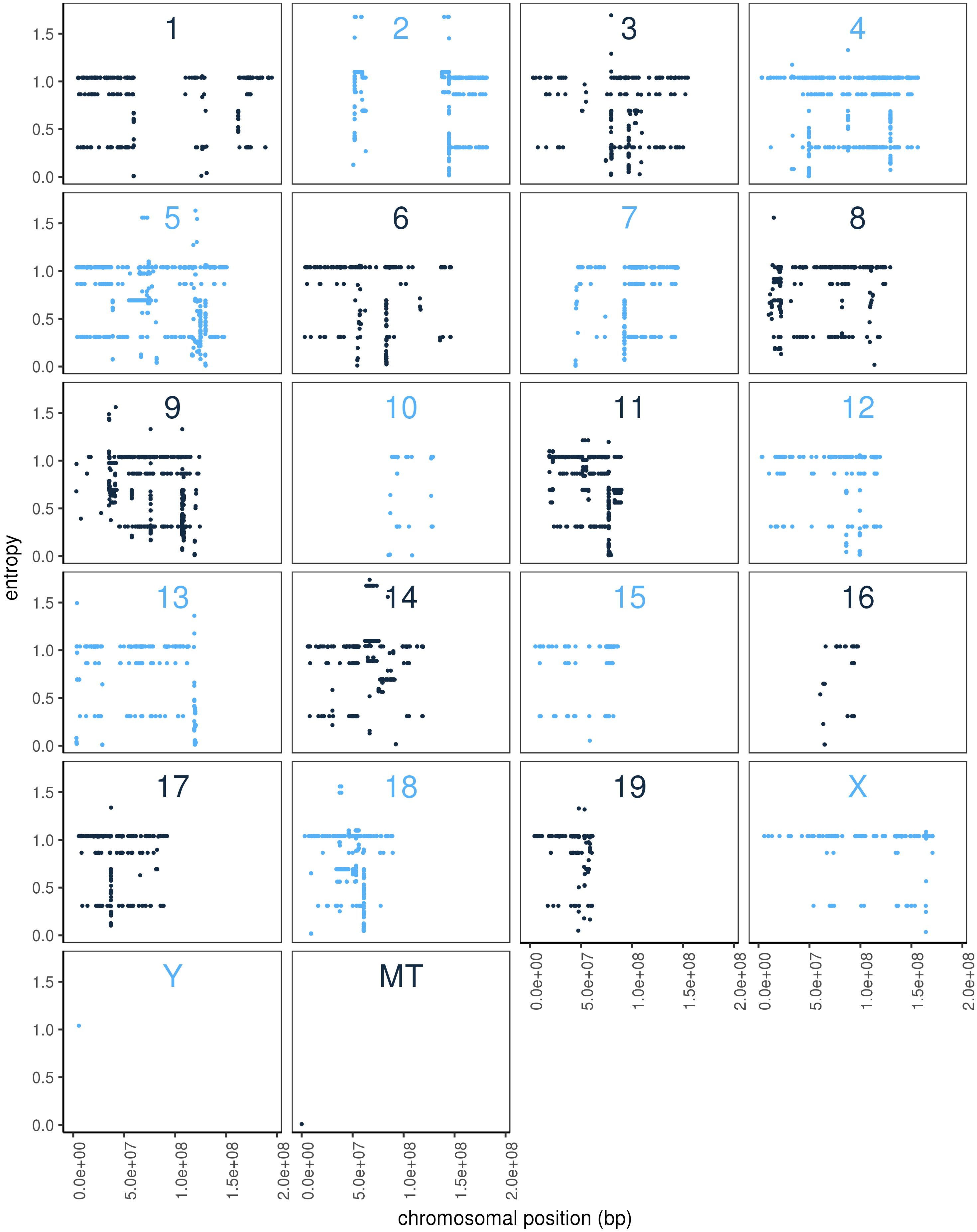

Figure S92 strain CC061, non-zero entropies in exons (+/-100 bp) in all chromosomes. Each point corresponds to the entropy of a variant at that position along the chromosome

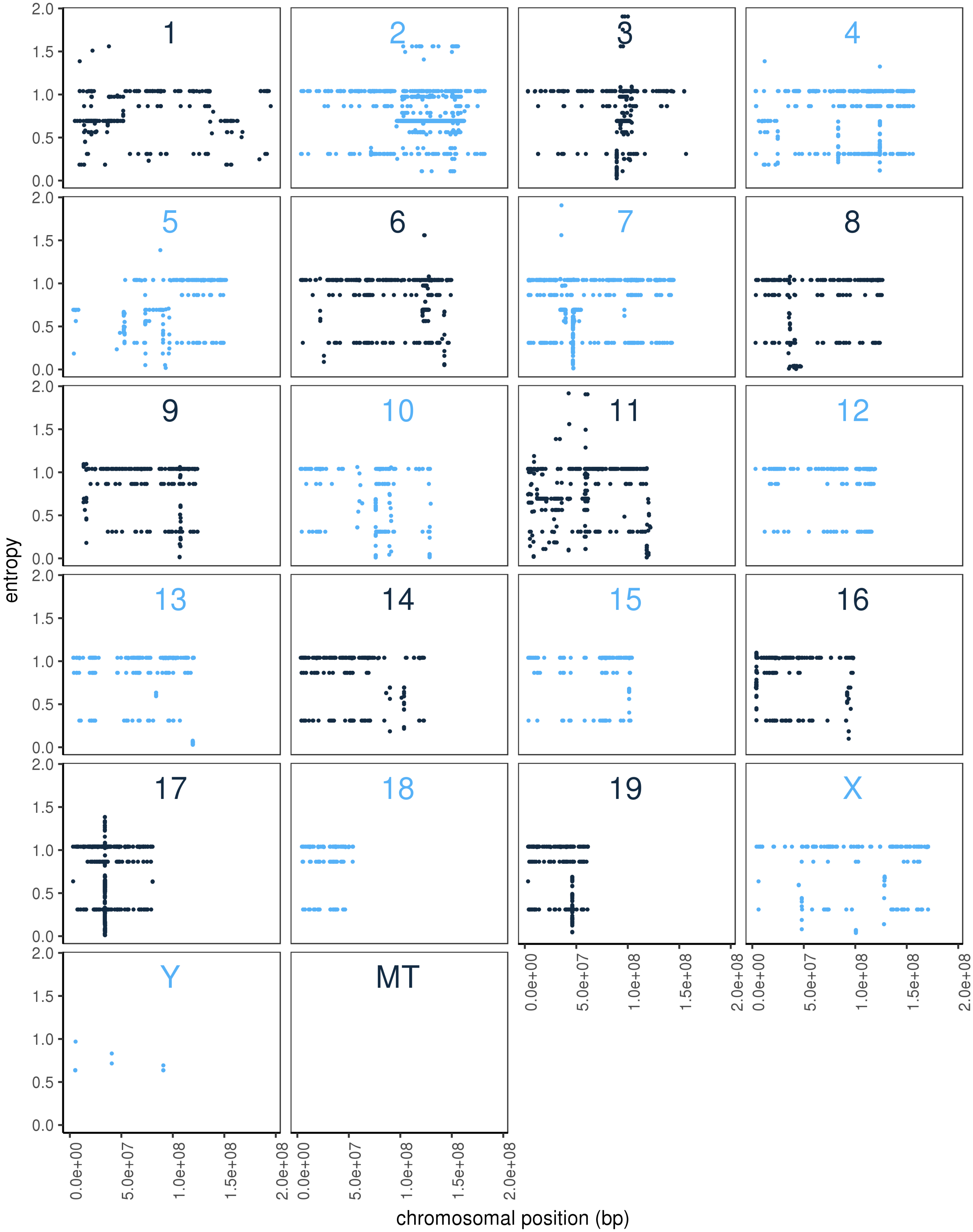

Figure S93 strain CC062, non-zero entropies in exons (+/-100 bp) in all chromosomes. Each point corresponds to the entropy of a variant at that position along the chromosome

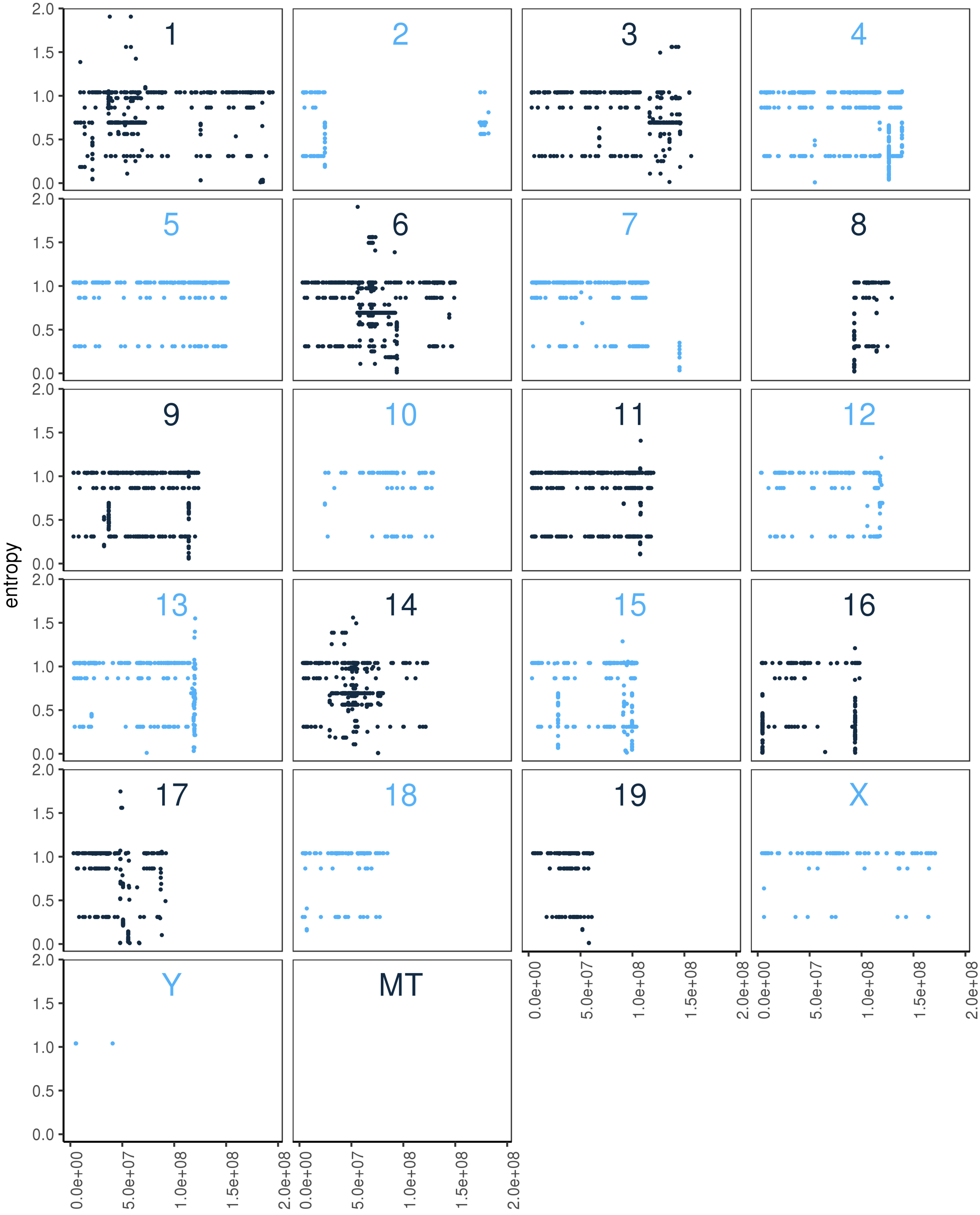

Figure S94 strain CC063, non-zero entropies in exons (+/-100 bp) in all chromosomes. Each point corresponds to the entropy of a variant at that position along the chromosome

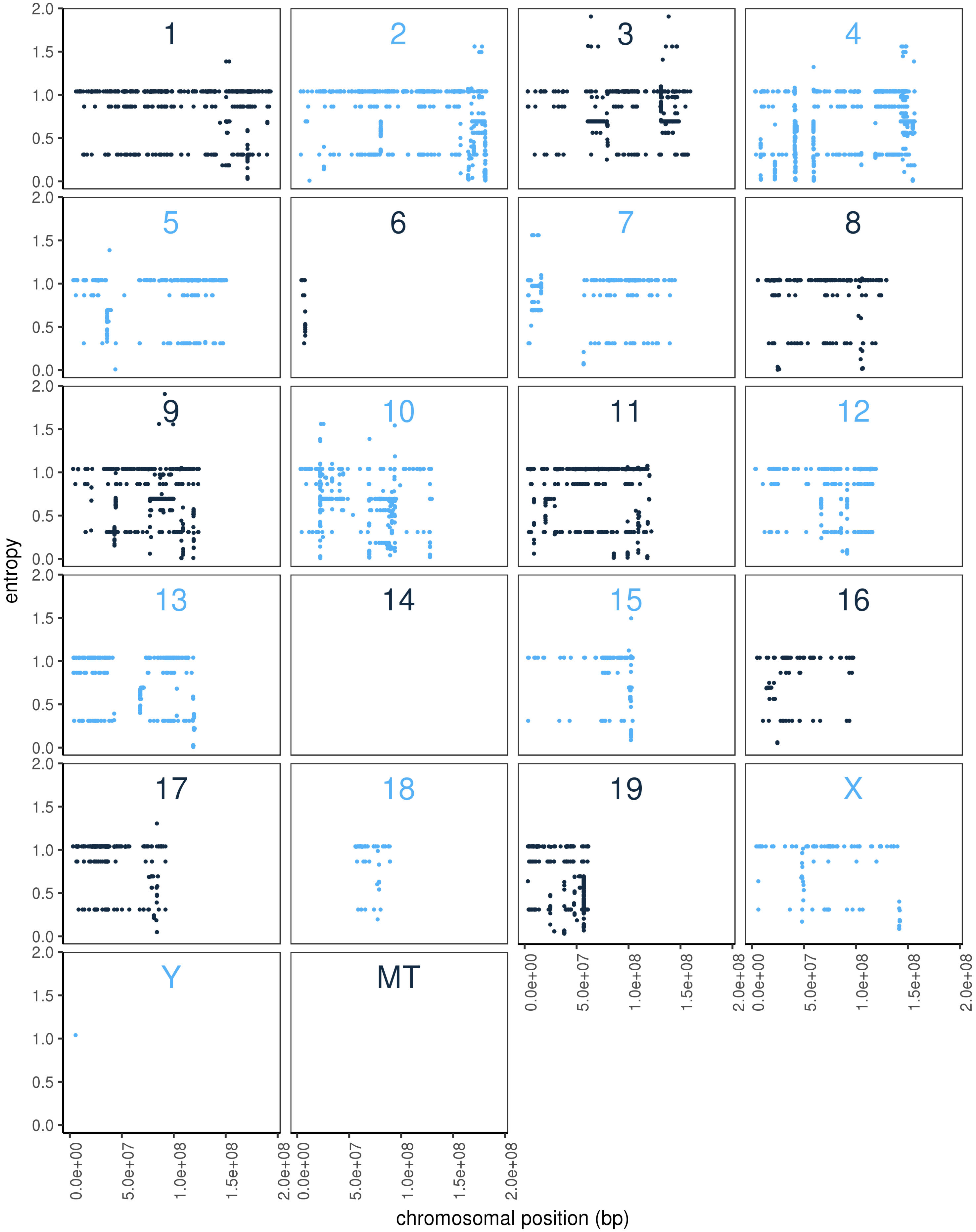

Figure S95 strain CC065, non-zero entropies in exons (+/-100 bp) in all chromosomes. Each point corresponds to the entropy of a variant at that position along the chromosome

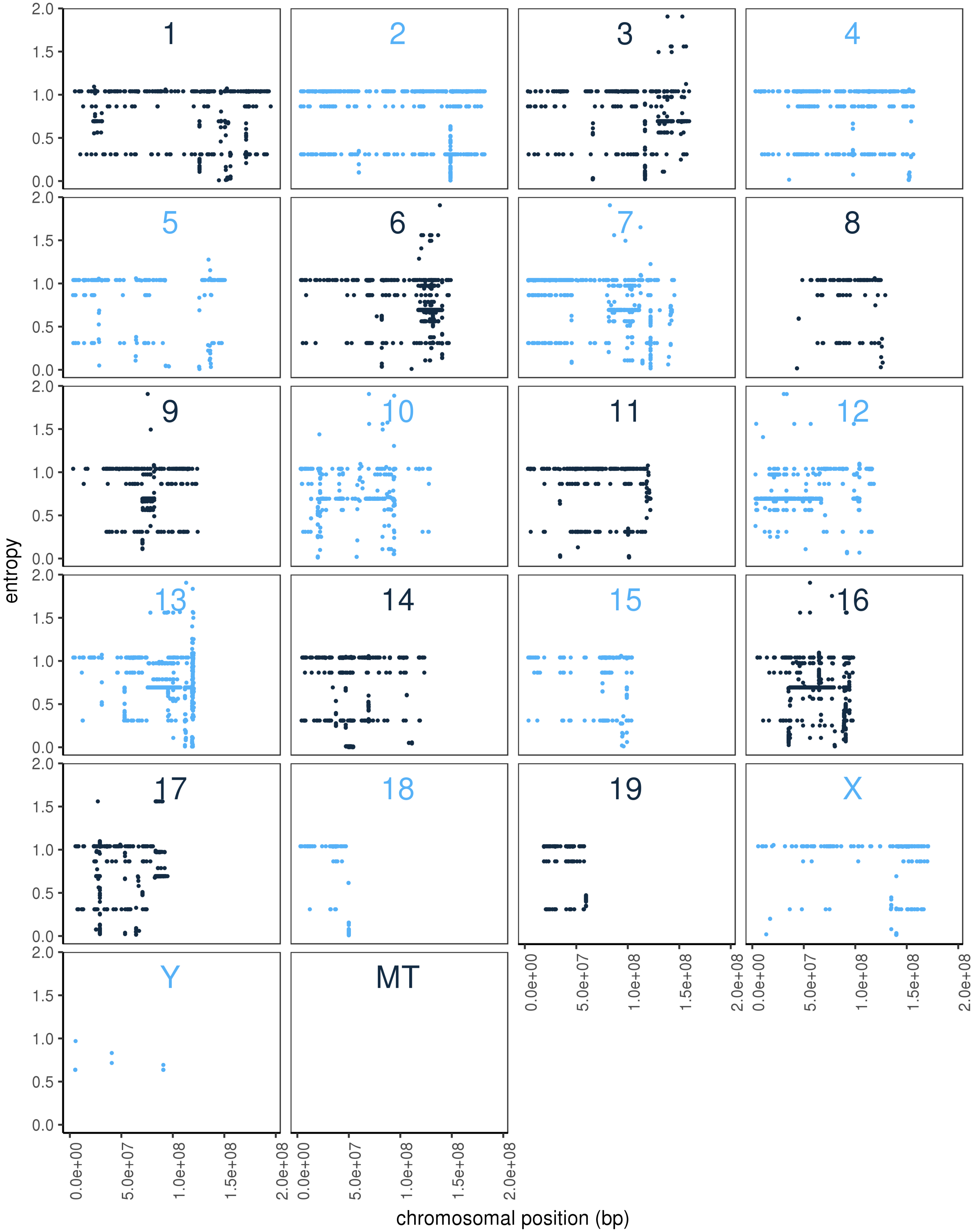

Figure S96 strain CC068, non-zero entropies in exons (+/-100 bp) in all chromosomes. Each point corresponds to the entropy of a variant at that position along the chromosome

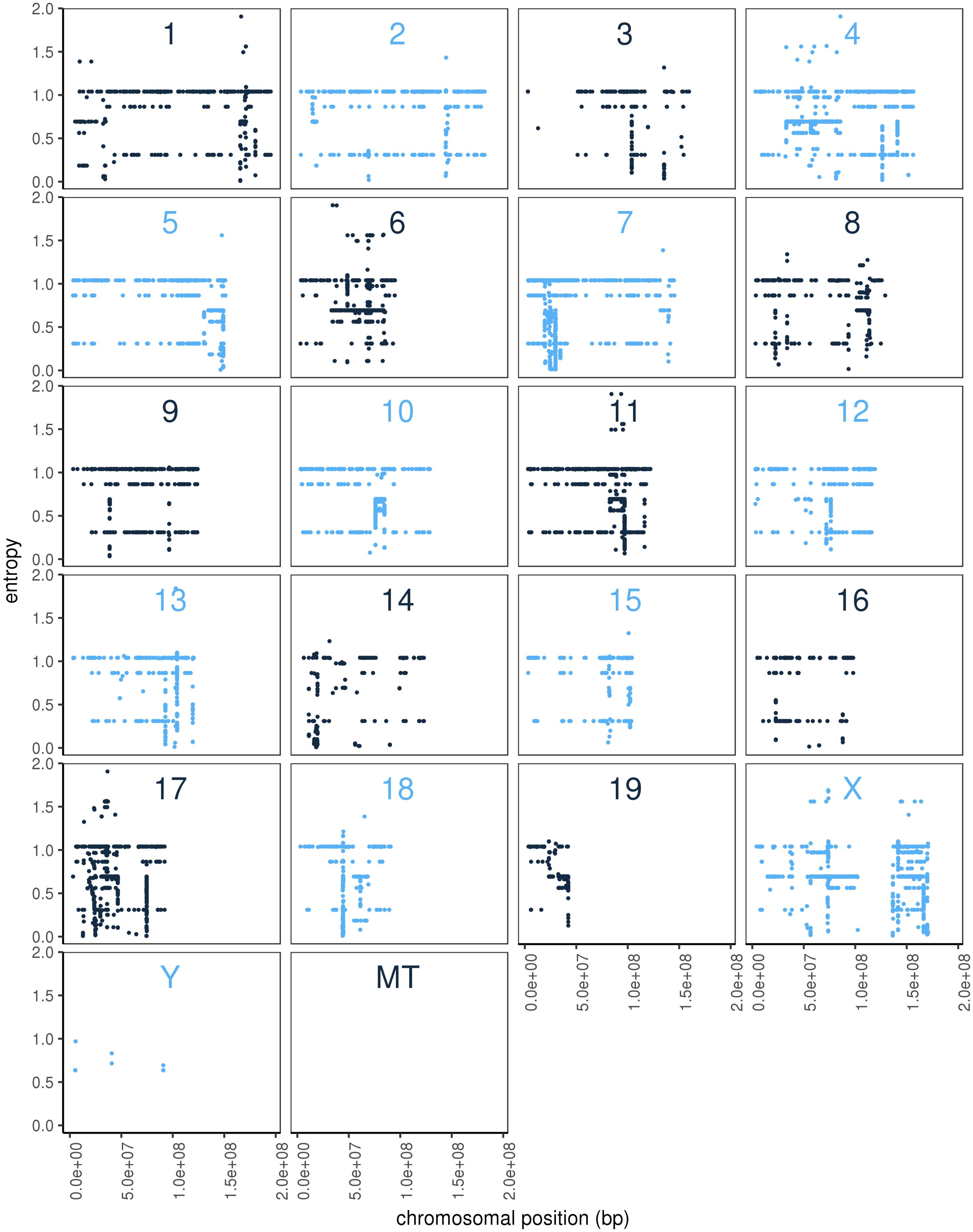

Figure S97 strain CC070, non-zero entropies in exons (+/-100 bp) in all chromosomes. Each point corresponds to the entropy of a variant at that position along the chromosome

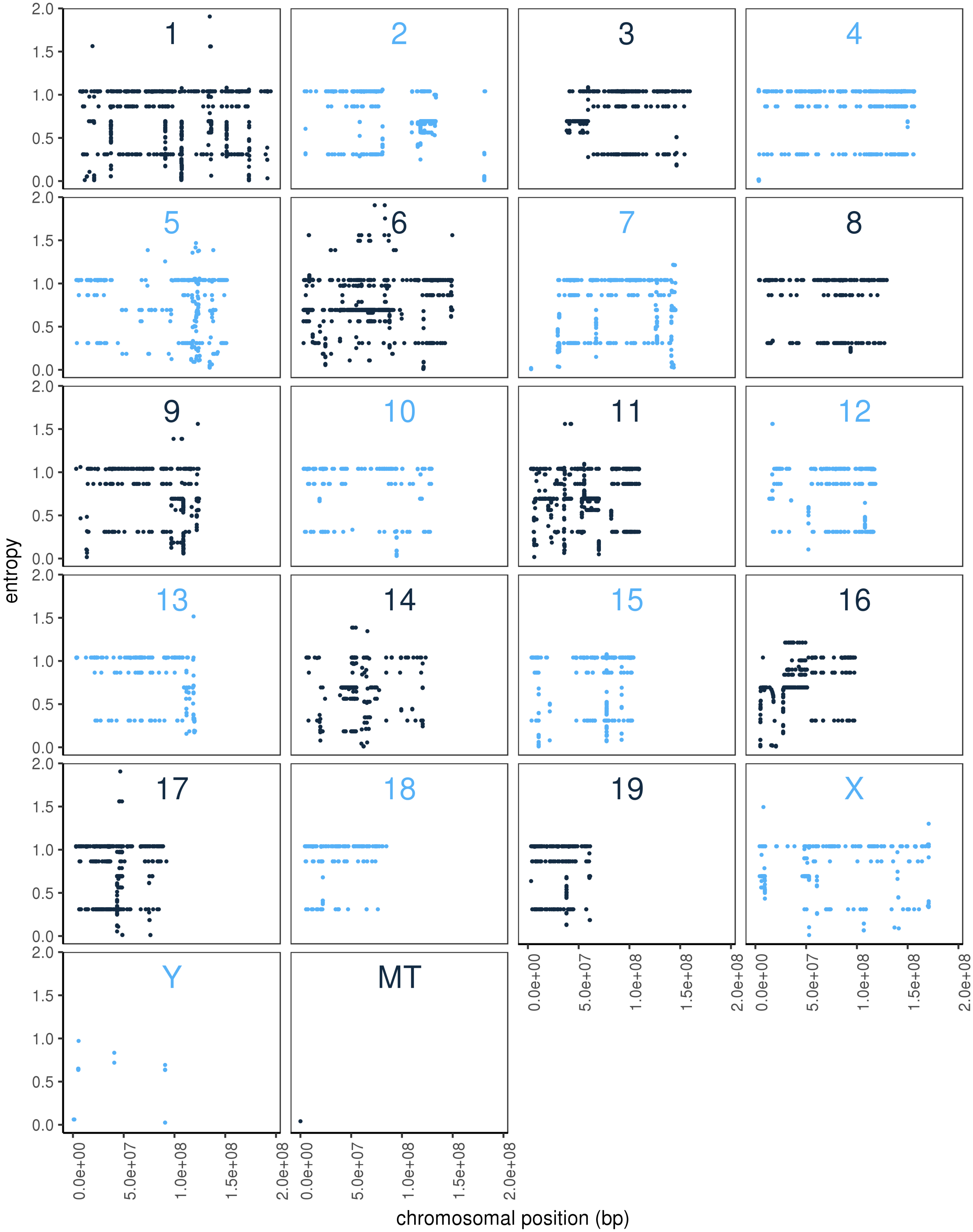

Figure S98 strain CC071, non-zero entropies in exons (+/-100 bp) in all chromosomes. Each point corresponds to the entropy of a variant at that position along the chromosome

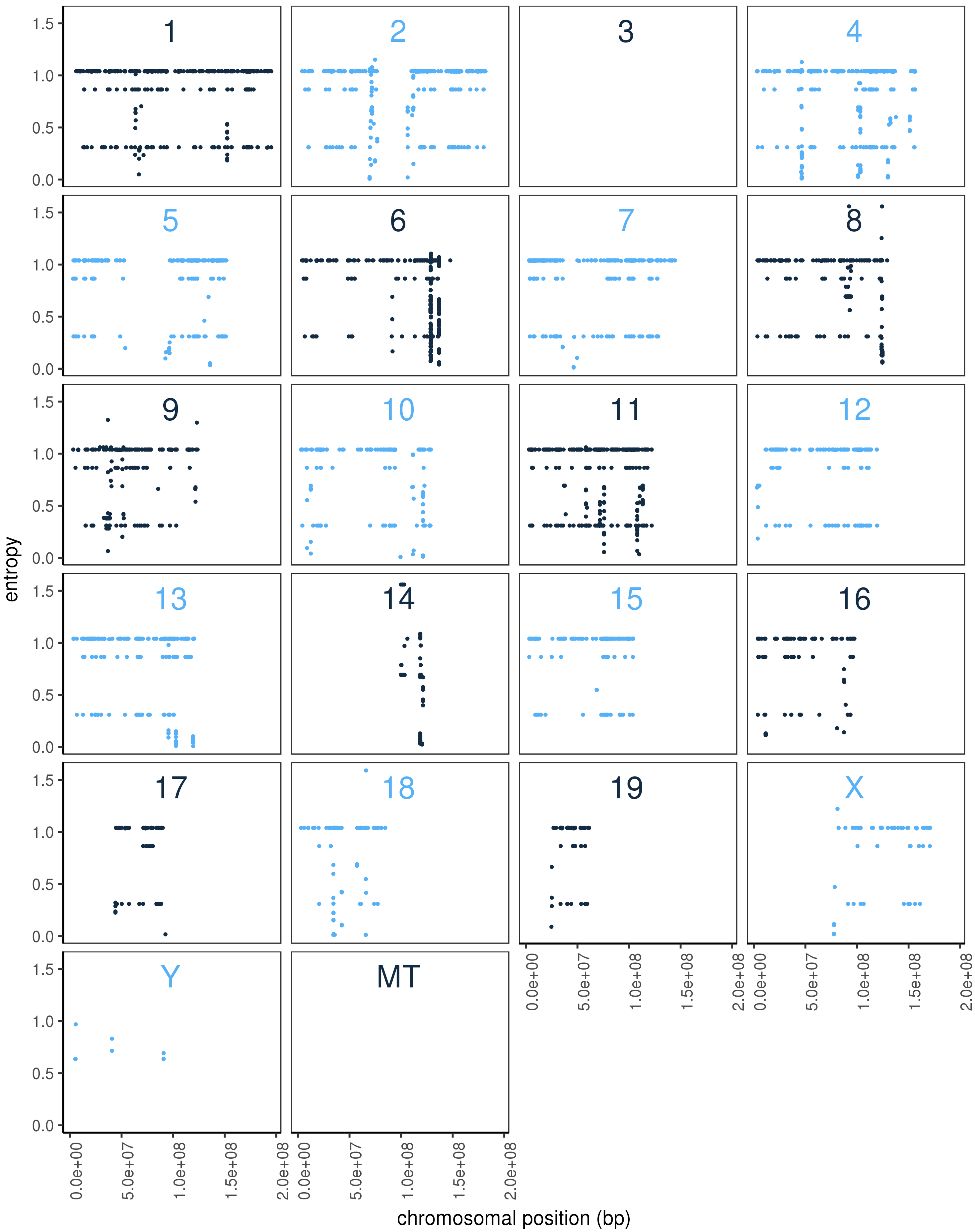

Figure S99 strain CC072, non-zero entropies in exons (+/-100 bp) in all chromosomes. Each point corresponds to the entropy of a variant at that position along the chromosome

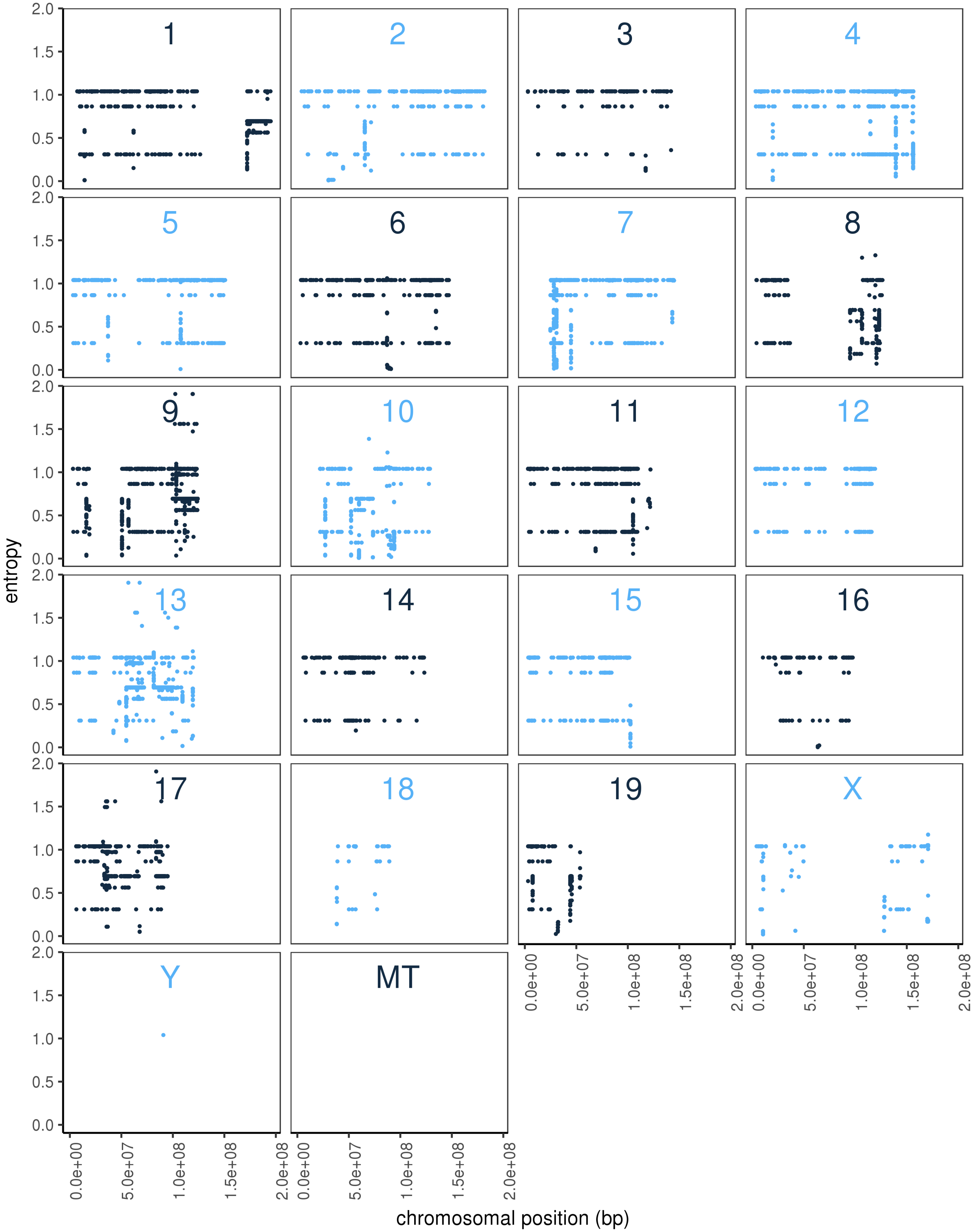

Figure S100 strain CC073, non-zero entropies in exons (+/-100 bp) in all chromosomes. Each point corresponds to the entropy of a variant at that position along the chromosome

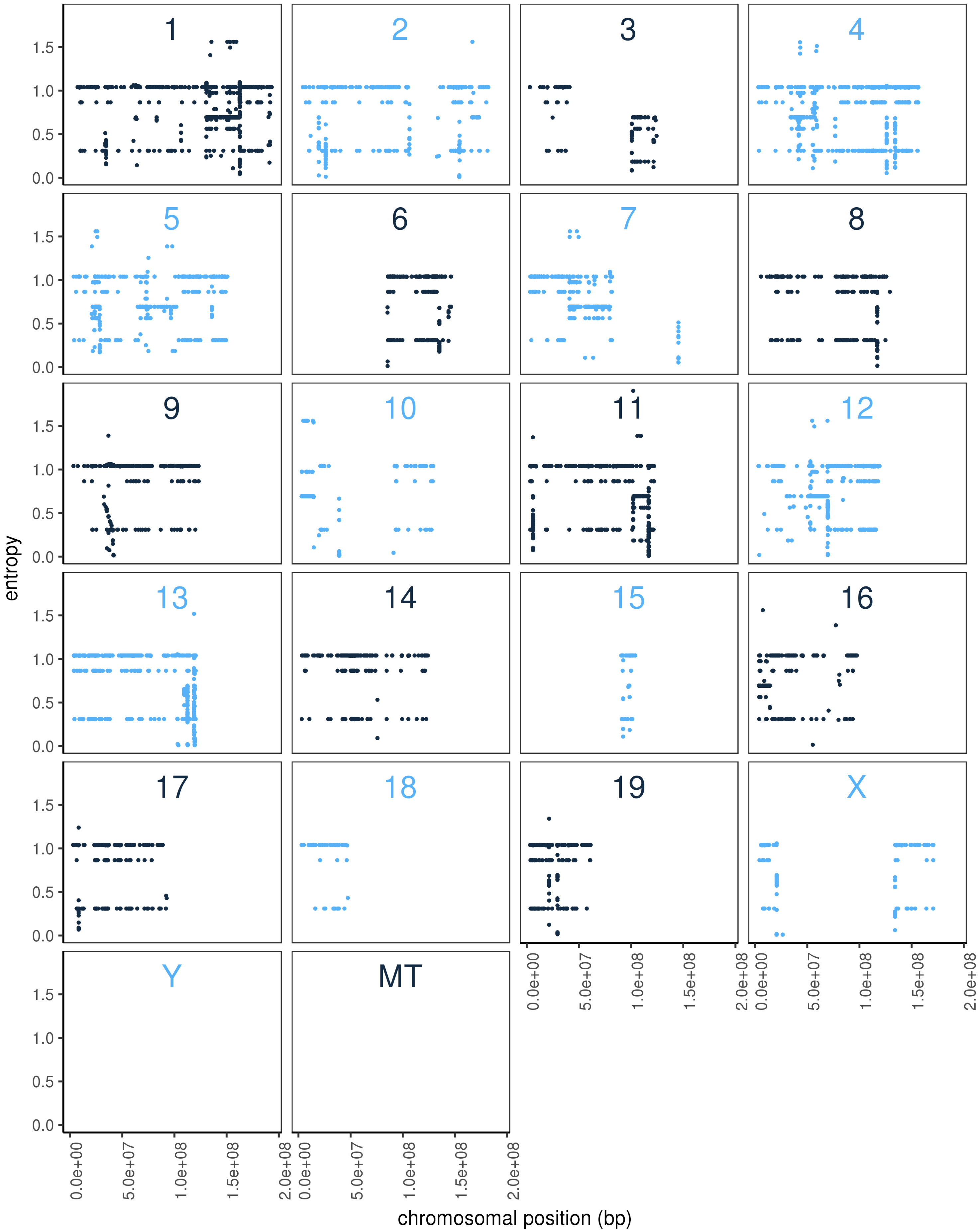

Figure S101 strain CC074, non-zero entropies in exons (+/-100 bp) in all chromosomes. Each point corresponds to the entropy of a variant at that position along the chromosome

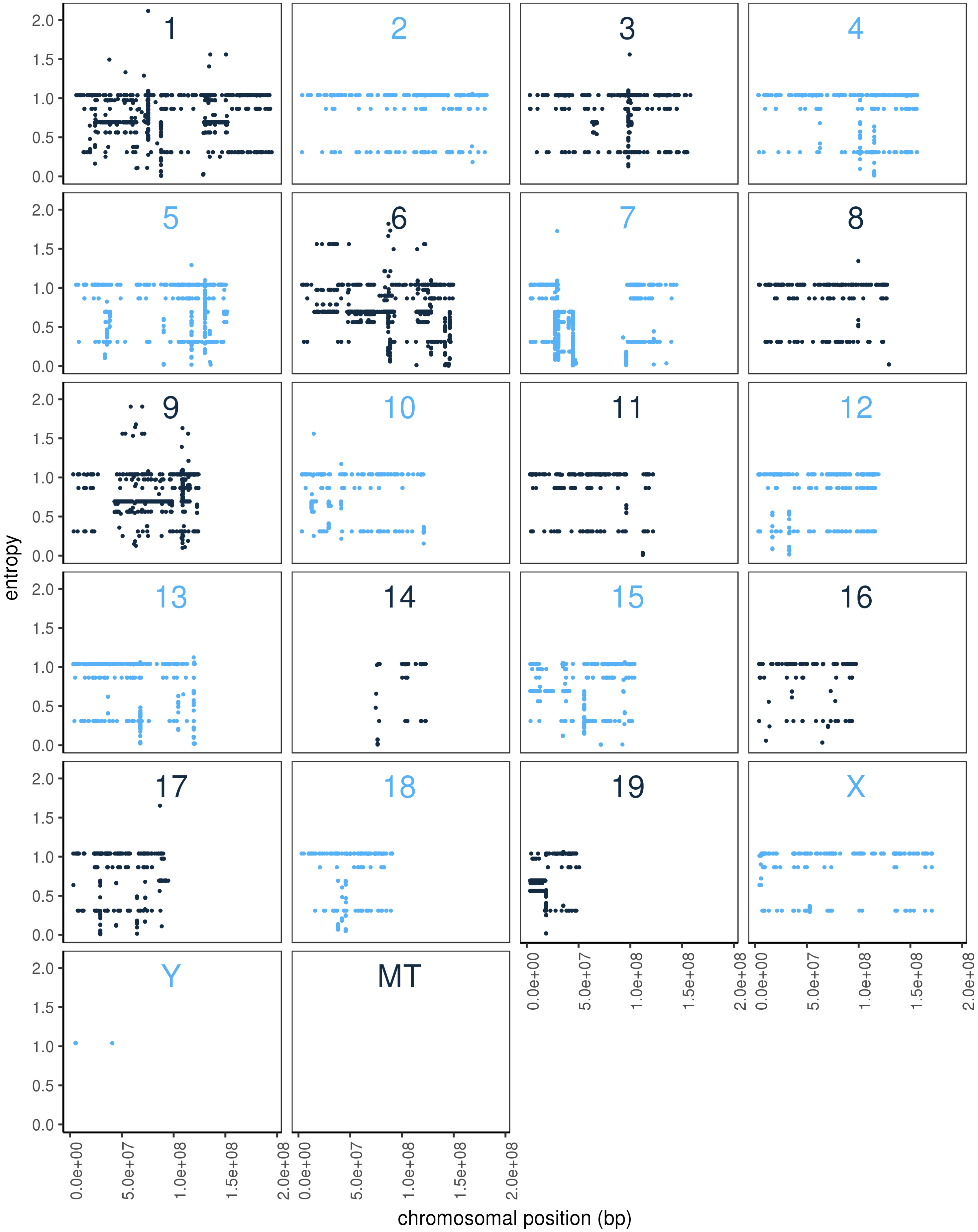

Figure S102 strain CC075, non-zero entropies in exons (+/-100 bp) in all chromosomes. Each point corresponds to the entropy of a variant at that position along the chromosome

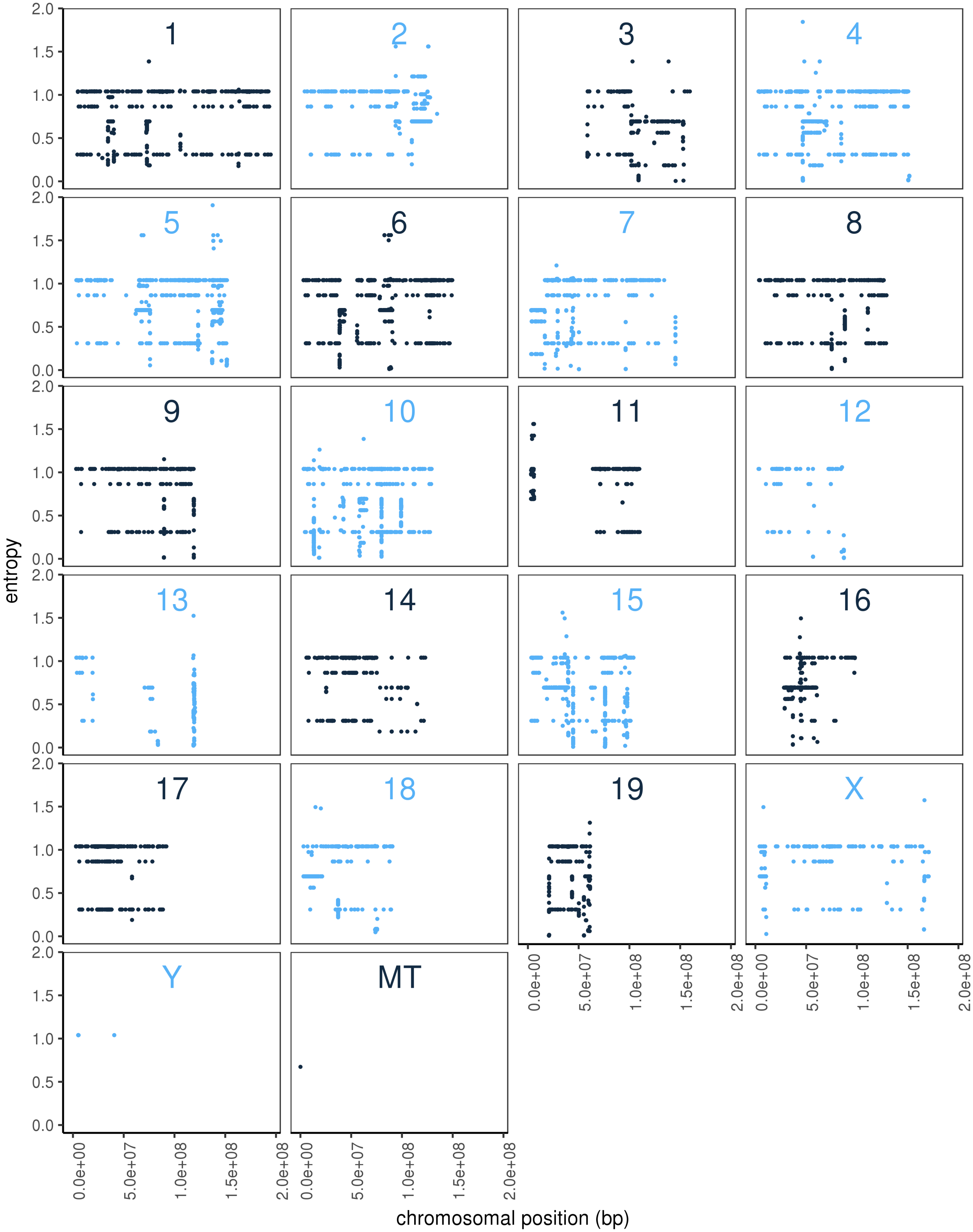

Figure S103 strain CC076, non-zero entropies in exons (+/-100 bp) in all chromosomes. Each point corresponds to the entropy of a variant at that position along the chromosome

3 Residual heterozygosity

The haplotyped most recent common ancestor (MRCA) per CC strain contained regions of residual heterozygosity. **Table S1** shows the imputed pct heterozygosity, per strain, per chromosome, as well as over all chromosomes. We define a variant as heterozygous if our imputation suggests at least a 25% chance of heterozygous genotype at that variant.

4 Predicted functional consequences counts

Predicted function consequences from founder variants are imputed for CC variants as well. **Table S2** shows the number of variants for each functional consequence, split per strain. In parentheses, is the proportion of all variants in a given strain that cause that predicted functional consequence. Note that proportions do not add up to 1 per strain, because some variants have more than one predicted consequence.
